# New sulfuryl fluoride-derived alkylating reagents for the 1,1-dihydrofluoroalkylation of thiols†
†Electronic supplementary information (ESI) available: Experimental procedures, methods, and optimization data; NMR, IR, and MS data including ^1^H, ^13^C, and ^19^F NMR spectra. See DOI: 10.1039/c9sc03570b


**DOI:** 10.1039/c9sc03570b

**Published:** 2019-09-20

**Authors:** Paul J. Foth, Frances Gu, Trevor G. Bolduc, Sahil S. Kanani, Glenn M. Sammis

**Affiliations:** a Department of Chemistry , University of British Columbia , 2036 Main Mall , Vancouver , British Columbia V6T 1Z1 , Canada . Email: gsammis@chem.ubc.ca

## Abstract

Herein, we report a new method for the one-pot synthesis of 1,1-dihydrofluoroalkyl sulfides by bubbling sulfuryl fluoride (SO_2_F_2_) through a solution of the corresponding alcohol and thiol. The reaction proceeds through a new class of bis(1,1-dihydrofluoroalkyl) sulfate reagents, to afford the desired 1,1-dihydrofluoroalkyl sulfides in 55–90% isolated yields. The bis(1,1-dihydrofluoroalkyl) sulfates are highly chemoselective for thiol alkylation, and are unreactive with competing, unprotected nucleophiles, including amines, alcohols, and carboxylic acids.

## Abstract 2

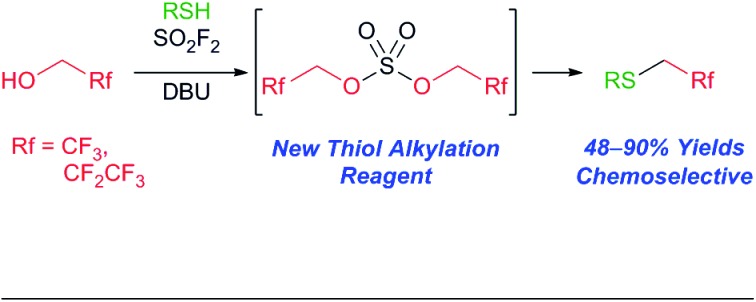
Herein, we report a new method for the one-pot synthesis of 1,1-dihydrofluoroalkyl sulfides by bubbling sulfuryl fluoride (SO_2_F_2_) through a solution of the corresponding alcohol and thiol.

## 

Sulfuryl fluoride (SO_2_F_2_) has been utilized since the 1960s as an industrial fumigant,^1^ but it has only recently attracted significant attention as a reagent for organic synthesis.1b,^2^ Studies have demonstrated that oxygen nucleophiles, such as alcohols (**1**),^3^,^4^ phenol derivatives (**2**),1b,^5^ oximes (**3**),^6^ and carboxylic acids (**4**),^7^ react with sulfuryl fluoride to form fluorosulfate derivatives (Scheme 1).^2^ The addition of a second equivalent of the oxygen nucleophile is kinetically slow, which allows fluorosulfate **5** to undergo subsequent transformations.^8^ Fluorosulfates (**5**) have been utilized as key reactants in a diverse range of reactions, including metal-catalyzed cross couplings,5c,^9^ click reactions,1b,5d deoxyfluorinations,5*b* alkylations,3a,^4^ nitrile syntheses,^6^ and the formation of amide bonds.7*a*

**Scheme 1:**
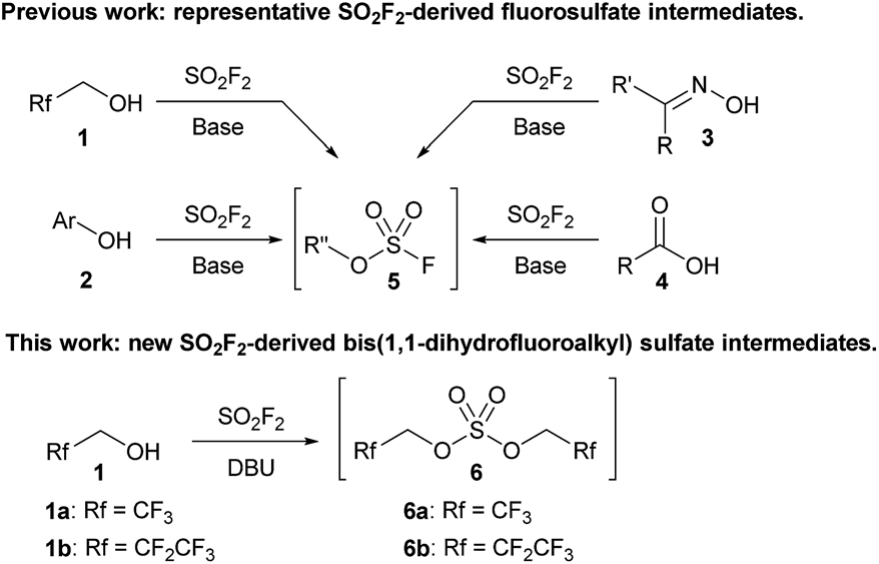
Representative examples of sulfuryl fluoride-mediated processes that utilize fluorosulfate reactive intermediates (**5**) and a new bis(trifluoroalkyl) sulfate (**6**).

All current synthetic methods rely on a very similar protocol for the formation of the fluorosulfate intermediate. Sulfuryl fluoride is bubbled through a solution of the requisite oxygen nucleophile and a base, which is usually *N*,*N*-diisopropylethylamine (DIPEA), triethylamine, or a carbonate salt.1b,^2^–^7^ Despite the expansion of the use of sulfuryl fluoride, the only reactive intermediates that have been identified are fluorosulfate derivatives (**5**), and no other sulfuryl fluoride-derived reactive intermediates have been explored.^10^

We previously reported that bubbling sulfuryl fluoride through a solution of 2,2,2-trifluoroethanol (**1a**) and an amine base, such as DIPEA or triethylamine, afforded trifluoroethyl fluorosulfate (**5a**, R′′ = CH_2_CF_3_) in >90% yield.3a,^11^ Following up on the synthesis and reactivity of fluorosulfate **5a** in new transformations, we serendipitously discovered that even moderately more basic reagents,^12^ such as 1,8-diazabicyclo[5.4.0]undec-7-ene (DBU) with a p*K*_aH_ of 12,^13^ afforded bis(trifluoroethyl) sulfate (**6a**) as the major product, and only trace amounts of fluorosulfate **5a** were detected by ^19^F NMR spectroscopy. Bis(trifluoroethyl) sulfate (**6a**) is an intriguing species as there are only two previous methods for its synthesis,^14^–^16^ and there are no studies investigating its reactivity.^17^

We elected to study the reactivity of this new bis(trifluoroethyl) sulfate intermediate (**6a**) for the 1,1-dihydrofluoroalkylation of thiols for two reasons: (1) the resulting fluoroalkyl sulfides are important fluorinated motifs in pharmaceuticals and agrochemicals,^18^–^20^ and (2) the more common sulfuryl fluoride-derived reagent, trifluoroethyl fluorosulfate **5a**, is not an effective intermediate for thiol alkylation. Previous studies by Shreeve and coworkers indicated that the reaction between methane thiol (**7**), triethylamine, and **5a** afforded the corresponding fluoroalkyl sulfide (**8**) in only 31% yield and a 2.2 : 1 preference for reactivity at carbon compared to sulfur (Scheme 2A).^14^

**Scheme 2:**
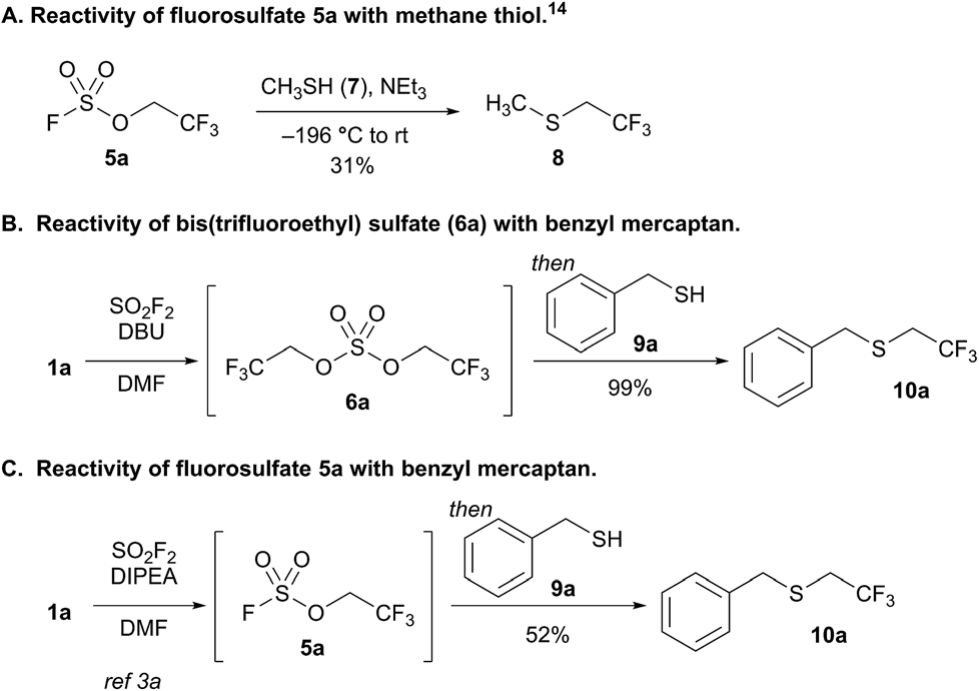
Investigations into thiol alkylation using fluorosulfate (**5a**) and bis(trifluoroethyl) sulfate (**6a**). The yields in (B) and (C) were determined by ^19^F NMR spectroscopy using trifluorotoluene as an internal standard.

To examine the viability of bis(trifluoroethyl) sulfate (**6a**) as a thiol alkylating reagent, we treated a solution of **6a** and DBU with benzyl mercaptan (**9a**), which led to a 99% ^19^F NMR yield of 1,1-dihydrofluoroalkylated product **10a** (Scheme 2B). The analogous reaction with a solution of DIPEA and fluorosulfate **5a** with **9a** afforded only 52% yield of **10a** (Scheme 2C), which is comparable to the result reported by Shreeve and coworkers.^14^ The addition of DBU and benzyl mercaptan to a solution of DIPEA and **5a** improved the yield; however, the reaction led to an increase in the amount of free trifluoroethanol in solution, presumably resulting from nucleophilic attack at sulfur.^21^

This initial result is noteworthy as it represents the first example of the direct conversion of an unactivated 1,1-dihydrofluoroalcohol to the corresponding fluoroalkyl sulfide in a one-pot process. Thiol 1,1-dihydrofluoroalkylation can be achieved through nucleophilic displacement of activated trifluoroalkyl moieties,^14^,^22^ copper-catalyzed reactions with trifluoroalkyl iodide^23^ or trifluorodiazoalkanes,^24^ or reductive trifluoroalkylthiolations.^25^ All previous work relies on activated trifluoroalkyl moieties. This is particularly problematic for select activated trifluoroethyl derivatives and longer chain 1,1-dihydrofluoroalkyl groups that are only available from the corresponding alcohols, and thus require additional synthetic steps to activate.

With an established protocol for a one-pot, sequential reaction, investigations next focused on a one-step process, where the alkylation proceeds by bubbling sulfuryl fluoride through a solution of trifluoroethanol (**1a**), benzyl mercaptan (**9a**), and DBU. At room temperature, the reaction proceeded efficiently to afford desired trifluoroethylated product **10a** in 71% yield (Table 1, entry 1). The yield increased at 40 °C (entry 2), but there was no further improvement when the reaction was run at 60 °C (entry 3). We next examined the reaction performance in different solvents (entries 4–8).^26^ Overall, the reaction was robust in a range of solvents, providing good yield in both polar aprotic (entries 4 and 5) and nonpolar solvents (entries 6 and 7). DCM was not as effective for this transformation, with product **10a** observed in only 38% yield (entry 8).^27^

**Table 1:** Optimization of the one-pot, 1,1-dihydrofluoroalkylation of benzyl mercaptan (**9a**)^*a*^

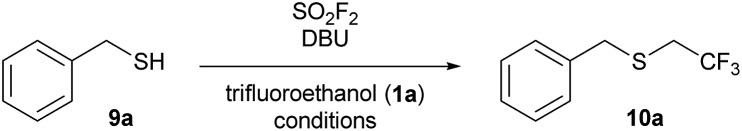			
Entry	Solvent	Temp (°C)	^19^F NMR yield^*b*^ (%)
1	DMF	25	71
2	DMF	40	86
3	DMF	60	85
4	THF	40	81
5	ACN	40	77
6	Hexane	40	75
7	Benzene	40	67
8	DCM	40	38

^*a*^All reactions were carried out following a one-pot procedure on 0.30 mmol scale of **9a** and a 1 : 1 v/v trifluoroethanol : solvent ratio.

^*b*^Yield after 20 minutes, as determined by ^19^F NMR spectroscopy using trifluorotoluene as an internal standard.

The one-pot reaction generally affords high yields of the desired thioalkylated product regardless of the steric bulk or the electronics of the thiol (Scheme 3). Benzyl mercaptan (**9a**) and furfuryl mercaptan (**9b**) were both efficiently trifluoroethylated to form the corresponding sulfides **10a** and **10b** in good yields. 1-Decanethiol (**9c**), 2-phenylethanethiol (**9d**), methyl thiolglycolate (**9e**), and 1,9-nonanedithiol (**9f**) were effective substrates for this transformation, affording mono- and dialkylated products **10c–10f** in 58% to 73% isolated yields. The reaction was insensitive to steric bulk alpha to the thiol, with both cyclohexyl mercaptan (**9g**) and triphenylmethanethiol (**9h**) alkylated in comparable yields (**10g** and **10h**, respectively). Electron rich and electron poor thiophenol derivatives were well tolerated, regardless of the position of the substituents (**10i-q**). Importantly, longer chain 1,1-dihydrofluoroalcohols, such as 2,2,3,3,3-pentafluoropropanol (**1b**), were viable starting materials; however extended reaction times were required. The isolated yields of **11a** and **11c** were increased to 90% and 89%, respectively, by conducting the reaction in a sequential one-pot manner,^28^ where sulfuryl fluoride was first bubbled through a solution of DBU and trifluoroethanol followed by the addition of the requisite thiol.

**Scheme 3:**
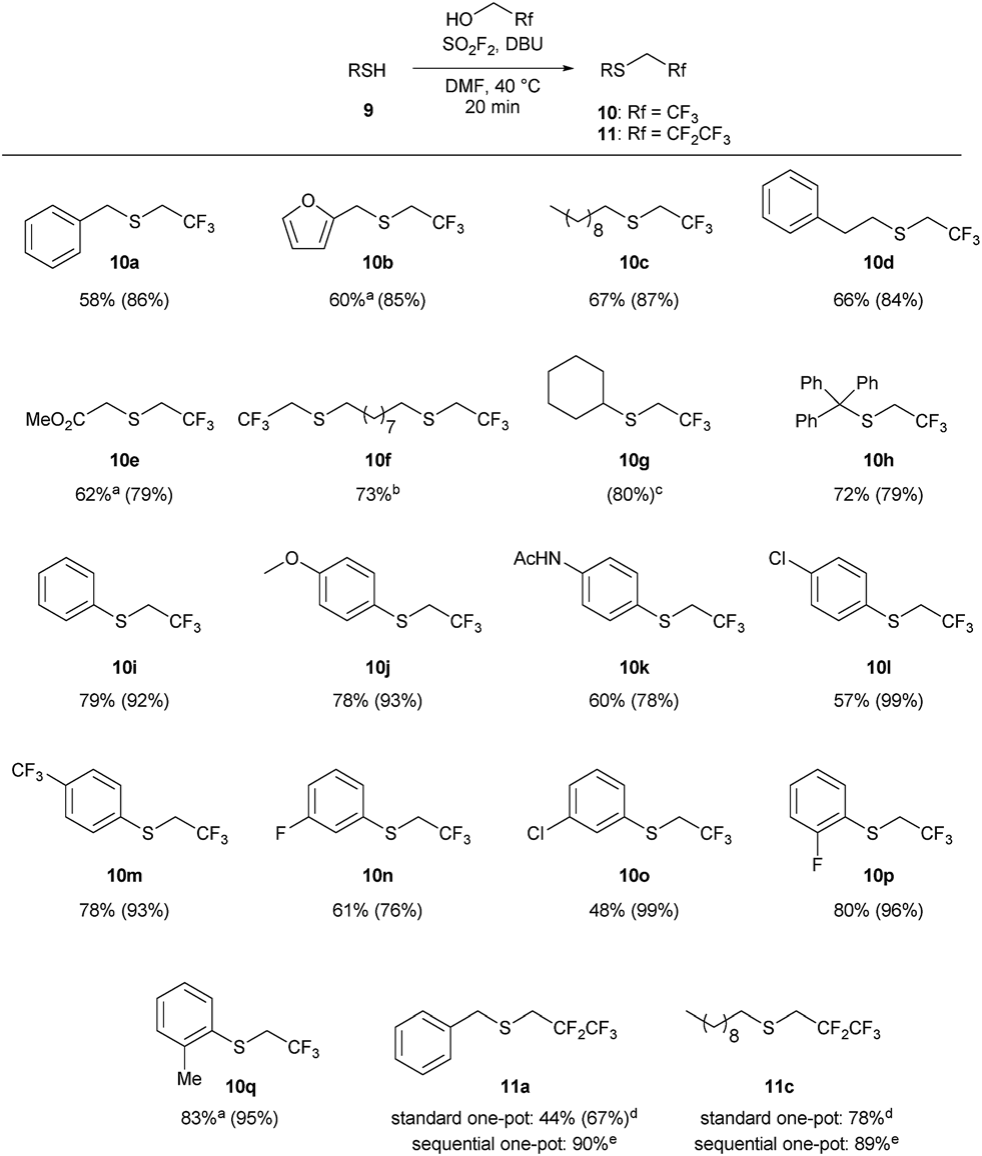
Substrate scope for the 1,1-dihydrofluoroalkylation of thiols. Reaction conditions: SO_2_F_2_ (2.9 equiv.) was bubbled through a solution of **9** (1 equiv.), DBU (5.9 equiv.) in 1 : 1 TFE/DMF (v/v), at 40 °C for 3 minutes, and then the reaction was stirred for an additional 17 min. All reactions were run on 1 mmol scale of thiol unless otherwise indicated. Isolated yields for the one-pot reaction are reported, with ^19^F NMR yields (using trifluorotoluene as the internal standard) provided in parentheses. ^a^The isolated yield has been corrected to account for disulfide or solvent impurities. See the ESI† for details. ^b^The reaction was conducted on 0.5 mmol scale. ^c^The product was not isolated due to volatility. ^d^The reaction was stirred for 2 hours. ^e^Pentafluoropropanol : DMF (1 : 2 v/v) was used to form the reagent, and then the thiol was added to the reaction mixture. The reaction was stirred for 30 minutes.

We next examined the functional group tolerance of this new thiol 1,1-dihydrofluoroalkylation (Scheme 4). In substrates in which there is competition between alcohol and thiol alkylation, the reaction cleanly afforded good yields of the desired thiol 1,1-dihydrofluoroalkylation products (**10r** and **11r**). Carboxylic acids were also tolerated, with good isolated yields of thiol alkylated product **10s** using either the standard one-pot or the sequential one-pot protocols. The reaction was selective for the thiol over potential competing reactivity at the nitrogen atom of aniline and pyridine derivatives (**10t**, **11t**, and **10u**). As primary nitrogen derivatives were competent nucleophiles in reactions with trifluoroalkyl fluorosulfate, we next examined the reaction of l-cysteine ethyl ester. Under our sequential one-pot conditions, reactivity was only observed at sulfur to give **10v** in 63% isolated yield.^29^

**Scheme 4:**
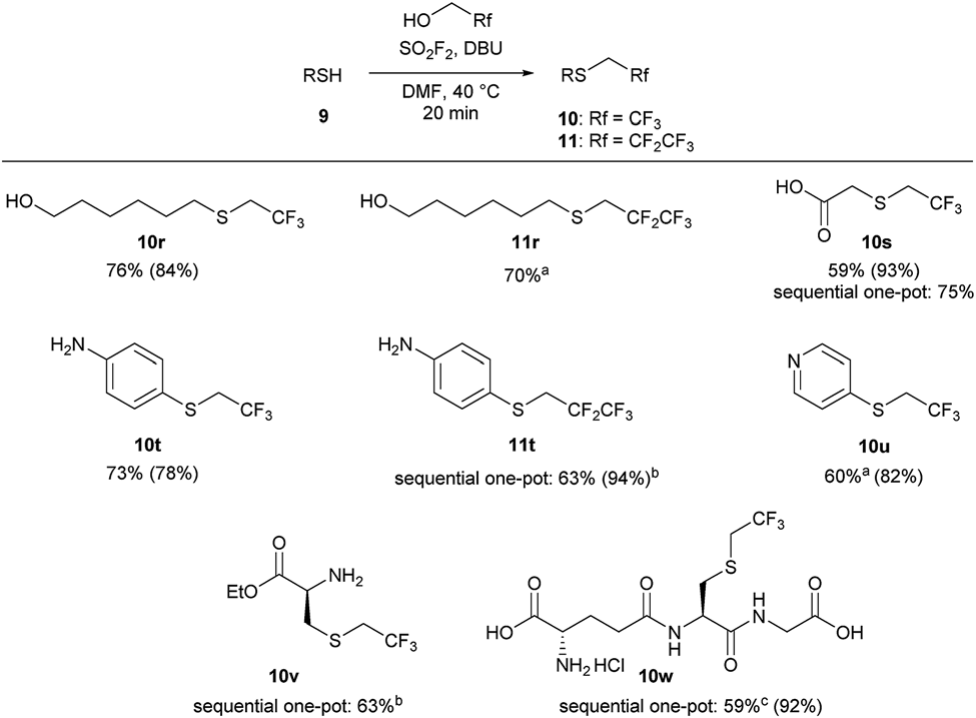
Functional group tolerance of the 1,1-dihydrofluoroalkylation reaction. Reaction conditions: SO_2_F_2_ (2.9 equiv.) was bubbled through a solution of **9** (1 equiv.), DBU (5.9 equiv.) in 1 : 1 TFE/DMF (v/v), at 40 °C for 3 minutes, and then the reaction was stirred for an additional 17 min. All reactions were run on a 1 mmol scale unless otherwise indicated. Isolated yields for the one-pot reaction are reported, with ^19^F NMR yields (using trifluorotoluene as the internal standard) provided in parentheses. ^a^The one-pot reaction was stirred for 2 hours. ^b^The sequential, one-pot reaction was stirred for 30 minutes after addition of thiol. ^c^The product was converted to an HCl salt, and the reported yield has been corrected for solvent impurities.

Finally, we investigated whether we could achieve selective 1,1-dihydrofluoroalkylation using glutathione (**9w**). Glutathione is a challenging substrate as it has two carboxylic acids, an amine, and two amides that may interfere with the desired thiol fluoroalkylation. Gratifyingly, under our sequential, one-pot reaction conditions, the thiol was selectively alkylated in 92% ^19^F NMR yield and 59% isolated yield. Under similar reaction conditions, trifluoroethyl triflate only afforded moderate yields of **10w**.

Intrigued by the chemoselectivity of the bis(trifluoroethyl) sulfate reagent (**6a**), we next investigated its selectivity compared to trifluoroethyl fluorosulfate (**5a**)^30^ in a competition experiment between benzyl mercaptan (**9a**) and piperidine (**12**)^31^ (Scheme 5).^32^ Addition of **9a** and **12** to a preformed solution of trifluoroethyl fluorosulfate and DIPEA afforded only a slight preference for thiol alkylation (Scheme 5A). Trifluoroethanol (**1a**) was liberated in the course of the reaction, which is likely the result of the addition of thiol to the sulfur center of the fluorosulfate reagent.^33^,^34^ Better selectivity for thiol *versus* amine alkylation could be achieved by adding DBU with **9a** and **12**;^35^ however, there was also a concomitant increase in the amount of **1a** (Scheme 5B). Increasing the amount of fluorosulfate reagent **5a** resulted in more amine alkylated product (**13**), but did not lead to a significantly better yield of **10a**. In contrast, formation of bis(trifluoroethyl) sulfate (**6a**) followed by addition of **9a** and **12** led to 90% yield of thiol alkylated product **10a**, and only trace amounts of **13** and trifluoroethanol (Scheme 5C). Further increasing the equivalents of the alkylating reagent led to near quantitative yield of **10a** (>97%). Even when pyrrolidine (**14**), a more nucleophilic amine,^36^ was used in a competition experiment, trifluoroethyl sulfide **10a** was obtained almost exclusively (Scheme 5D).^37^

**Scheme 5:**
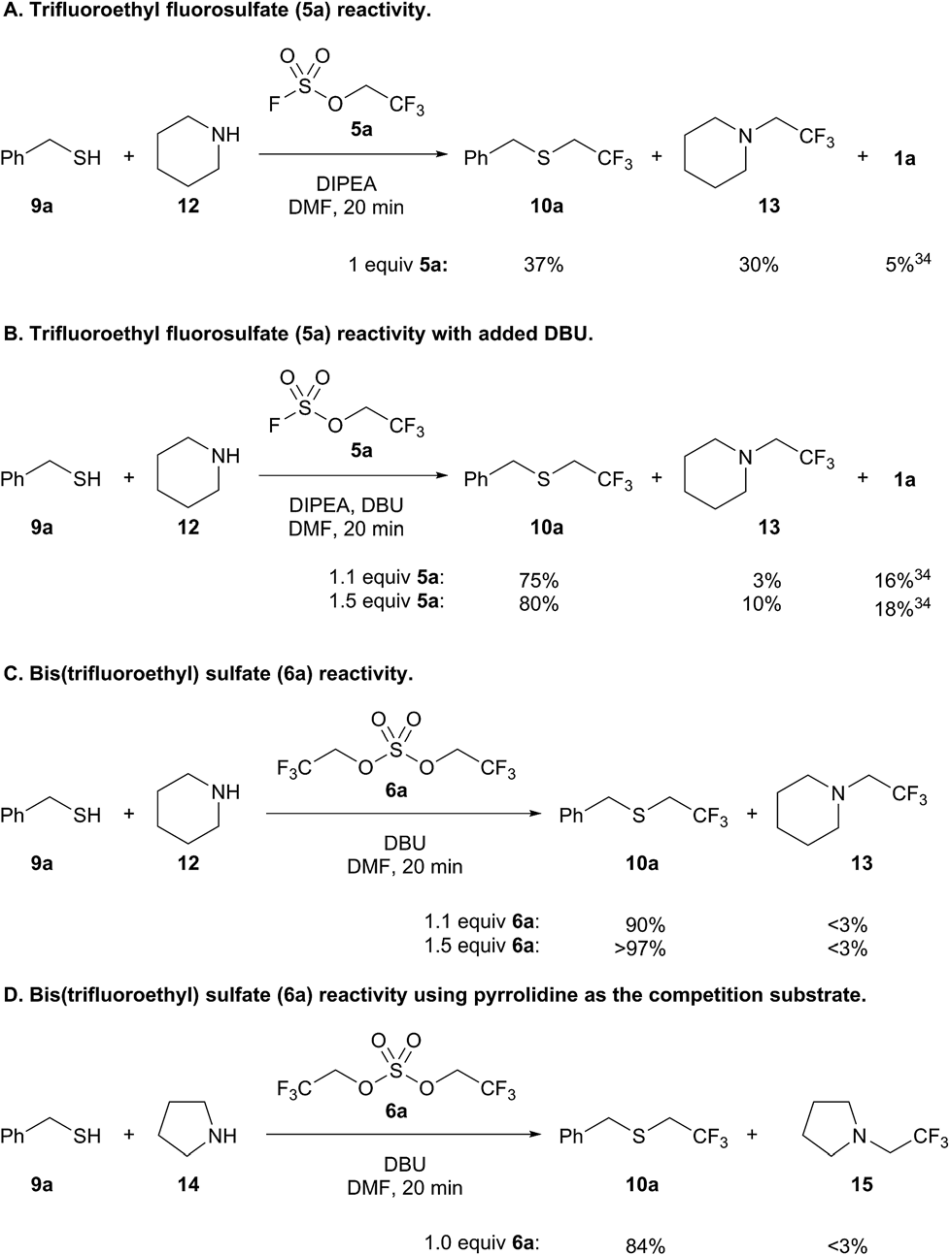
Competition experiments with sulfur and nitrogen nucleophiles.

Overall, we have developed a new method for the 1,1-dihydrofluoroalkylation of thiols using a previously unexplored, sulfuryl fluoride derived bis(trifluoroethyl) sulfate reagent (**6a**). This protocol enables the one-pot activation and thiolation of 1,1-dihydrofluoroalcohols to afford industrially relevant moieties in high yields, regardless of the sterics or electronics of the starting thiol. *In situ* generated bis(trifluoroethyl) sulfate (**6a**) is highly selective for thiols, even in the presence of unprotected alcohols, carboxylic acids, or amines, allowing for possible late-stage functionalization. Compared to trifluoro-ethyl fluorosulfate, the new bis(trifluoroethyl) sulfate reagent displays superior thiol alkylation chemoselectivity over both competing amine alkylation and reactivity at the sulfate center. Efforts to further explore this new class of bis(1,1-dihydrofluoroalkyl) reagents in the context of other reactions are currently underway.

## Conflicts of interest

There are no conflicts to declare.

## Supplementary Material

Supplementary information
