# Supplementary material for: New sulfuryl fluoride-derived alkylating reagents for the 1,1-dihydrofluoroalkylation of thiols
Source: Chem Sci. 2019 Sep 20;10(44):10331–5. doi: 10.1039/c9sc03570b (PMC6984387; doi:10.1039/c9sc03570b)

Electronic Supplementary Material (ESI) for Chemical Science.
This journal is © The Royal Society of Chemistry 2019

Supplementary Information for:

New Sulfuryl Fluoride-Derived Alkylating Reagents for the 1,1-Dihydrofluoroalkylation of Thiols

*Paul J. Foth, Frances Gu, Trevor G. Bolduc, Sahil S. Kanani, Glenn M. Sammis**

Table of contents

**General methods and instrumentation**	**S2**
**Optimization of one-pot conditions for the trifluoroethylation of benzyl mercaptan (9a)**	**S3**
**Representative example for the ^{19}F NMR yield determination**	**S4**
**Synthesis and characterization of bis(trifluoroethyl) sulfate (6a) and bis(pentafluoropropyl) sulfate (6b)**	**S5**
**Studies exploring the effect of base on the reactions between benzyl mercaptan (9a) and trifluoroethyl fluorosulfate (5a)/bis(trifluoroethyl) sulfate (6a)**	**S6**
**Reactivity studies with benzyl mercaptan (9a) and piperidine (12)**	**S7**
**Reactivity studies with benzyl mercaptan (9a) and pyrrolidine (14)**	**S9**
**Reactivity studies with glutathione and trifluoroethyl triflate (S1)**	**S10**
**General procedure for the one-pot trifluoroethylation of thiols**	**S11**
**General procedure for the trifluoroethylation of thiols by sequential addition**	**S12**
**General procedure for the synthesis of 2,2,3,3,3-pentafluoropropyl sulfides**	**S12**
**Thiobenzoic acid experiments**	**S24**
**References**	**S25**
**^{1}H, ^{13}C and ^{19}F NMR Spectra**	**S26**

General methods and instrumentation

All reactions were performed in 4 or 20 mL glass vials under nitrogen atmosphere unless otherwise noted. All chemicals were purchased from commercial sources and used as received unless otherwise noted. Sulfuryl diimidazole (SDI) was prepared and used to generate sulfuryl fluoride (SO_2F_2) following a modified procedure from *Org. Lett.* 2017, **19**, 5244–5247. Screw caps and PTFE/Silicon septa were purchased from Chemglass Life Sciences LLC. BD Intramedic™ (I.D. 1.57mm, O.D. 2.08mm) polyethylene tubing was used for the reactions. 2,2,2-Trifluoroethanol is abbreviated as TFE, and 1,8-diazabicyclo[5.4.0]undec-7-ene is abbreviated as DBU. 2,2,2-trifluoroethyl trifluoromethanesulfonate is referred to as trifluoroethyl triflate.

N-*N*-Dimethylformamide (DMF) was dried over 4 Å molecular sieves. Acetonitrile (ACN), dichloromethane (DCM), tetrahydrofuran (THF), benzene, and hexanes were obtained from a solvent purification system (SPS).

Flash column chromatography was performed using Silicycle F60 silica: 230-400 mesh (40-63 µm) silica. TLC's were run on Merck Kieselgel 60 F_{254} aluminum or glass backed plates and visualized by UV fluorescence (254 nm) then one of the following: $KMnO_4$, ninhydrin, p-anisaldehyde, bromocresol green.

Infrared (IR) spectra were obtained using a Thermo Nicolet 4700 FT-IR spectrometer or a Perkin Elmer Frontier FT-IR. The spectra are reported in cm^{-1}.

High resolution mass spectra (HRMS) were recorded on a Waters or Micromass LCT spectrometer or a JEOL AccuTOF-GC spectrometer.

Proton, carbon and fluorine nuclear magnetic resonance (^{1}H, ^{13}C and ^{19}F NMR) spectra were recorded using a Bruker AV-300 or Bruker AV-400 spectrometer. Chemical shifts (δ) are reported in parts per million (ppm) and are referenced to the centerline of $CDCl_3$ (7.26 ppm ^{1}H NMR; 77.16 ppm ^{13}C NMR), CD_2Cl_2 (5.32 ppm ^{1}H NMR; 54.00 ppm ^{13}C NMR), D_2O (4.79 ppm ^{1}H NMR). ^{19}F NMR chemical shifts were referenced to $CFCl_3$ (0.00 ppm). Coupling constants (*J*) are reported in Hz to the nearest 0.1 Hz. Peak multiplicity is indicated as follows: s (singlet), d (doublet), t (triplet), q (quartet), br s (broad singlet), m (multiplet), dd (doublet of doublets), td (triplet of doublets), tq (triplet of quartets), qd (quartet of doublets), qt (quartet of triplets). Assignment of peaks was done based on the chemical shifts, multiplicities and integrals of the peaks.

NMR yields were determined by ^{19}F NMR using a relaxation delay (or recycle delay) of 40 seconds to ensure complete relaxation of all fluorine nuclei. α,α,α-Trifluorotoluene ($PhCF_3$, δ -63.72 ppm) was used as an internal standard, unless otherwise specified.

Optimization of one-pot conditions for the trifluoroethylation of benzyl mercaptan (9a)

Table S1 – Optimization of the one-pot thiol trifluoroethylation

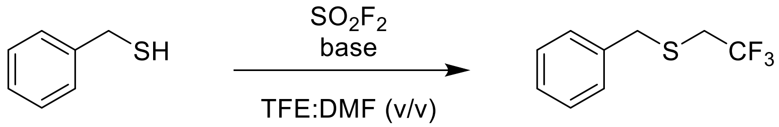

entry	base	TFE:DMF (v/v)	TFE (equiv)	time (min)	^{19}F NMR yield (%)
1	DIPEA	1:4	8	120	12
2	DIPEA	1:2	13	120	40
3	DIPEA	1:1	20	120	53
4	Et_3N	1:1	20	20	39
5	DBU	1:1	20	20	86
6	*t*BuOK	1:1	20	20	62
7	KHMDS	1:1	20	20	77
8	LHMDS	1:1	20	20	27
9	DBU	1:2	13	20	84
10	DBU	1:4	8	20	82
11	DBU	1:6	6	20	77
12	DBU	1:10	4	20	73

Conditions: The reactions were run on 0.3 mmol scale of benzyl mercaptan in 4 mL vials following the general procedure for the one-pot trifluoroethylation of thiols. The yield was determined by ^{19}F NMR spectroscopy using $PhCF_3$ as an internal standard.

Comments on the amount of trifluoroethanol utilized: While the reaction gives comparable results using 1:1, 1:2, and 1:4 TFE:DMF (v/v) ratios, we selected 1:1 for studies investigating the scope primarily for two reasons. First, when exploring the scope, we wanted to increase the rate of bis(trifluoroethyl) sulfate (**6a**) formation relative to competing thiol addition to sulfuryl fluoride. Thus, a high concentration of trifluoroethanol was utilized. Second, even though it is less atom economical, trifluoroethanol is commercially available, relatively inexpensive, easily removed from the reaction, and often used as solvent. Therefore, a 1:1 ratio was selected.

Representative example for the ^{19}F NMR yield determination

$$\left(\frac{\left(\frac{integration\ value\ of\ product}{number\ of\ nuclei\ in\ product}\right)}{\left(\frac{integration\ value\ of\ internal\ standard}{number\ of\ nuclei\ in\ internal\ standard}\right)}\right)\left(\frac{\left(\frac{mass\ internal\ standard\ (g)}{MW\ internal\ standard\ \left(\frac{g}{mol}\right)}\right)}{\left(\frac{mass\ substrate\ (g)}{MW\ substrate\ \left(\frac{g}{mol}\right)}\right)}\right) \text{x } 100\%$$

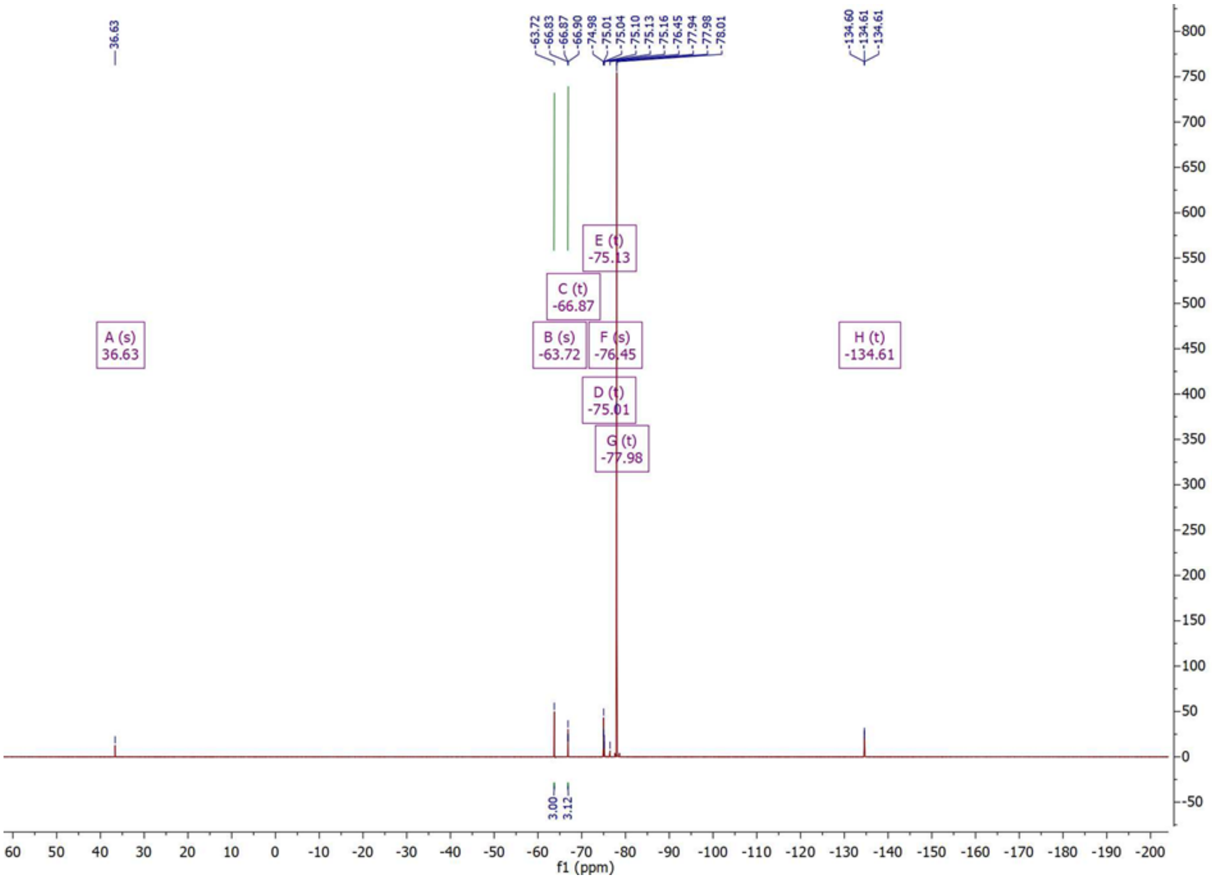

Trifluorotoluene: -63.7 ppm

Trifluoroethyl sulfide: -64 to -68 ppm (depending on the substrate)

$$\left(\frac{\left(\frac{3.12}{3\text{ F nuclei}}\right)}{\left(\frac{3.00}{3\text{ F nuclei}}\right)}\right)\left(\frac{\left(\frac{0.1202\ g\ trifluorotoluene}{146.11\ \frac{g}{mol}\ trifluorotoluene}\right)}{\left(\frac{0.124\ g\ \text{benzyl mercaptan}}{124.21\ \frac{g}{mol}\ benzyl\ mercaptan}\right)}\right) \text{x } 100\% = 85\%$$

Synthesis and characterization of bis(trifluoroethyl) sulfate (6a) and bis(pentafluoropropyl) sulfate (6b)

Scheme S1 – Synthesis and characterization of bis(trifluoroethyl) sulfate (**6a**) and bis(pentafluoropropyl) sulfate (**6b**)

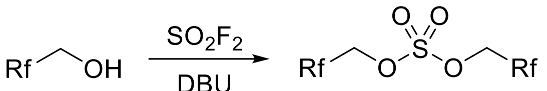

1 **6**

1a: Rf = CF_3 **6a**: Rf = CF_3

1b: Rf = CF_2CF_3 **6b**: Rf = CF_2CF_3

Procedure

Two 20 mL vials, equipped with magnetic stir-bars, were capped with septum-fitted vial caps and connected by a polyethylene tube. Vial A was charged with SDI (2.57 mmol, 0.78 equiv) and anhydrous KF (7.05 mmol, 2.1 equiv), then the system was placed under N_2 atmosphere. To vial B was added DCM (2.3 mL), DBU (4.7 mmol, 1.4 equiv), and **1** (3.3 mmol, 1 equiv). The polyethylene tube in vial B was immersed into the solution and then to vial A was added TFA (1.6 mL) in one portion. Vigorous bubbling of SO_2F_2 and fuming were observed in vial B for 2-3 minutes. When the bubbling subsided, vial B was vented via a needle for approximately 1 minute (this triggered more bubbling of SO_2F_2 through the solution). The tube and needle were then removed and the mixture in vial B was allowed to stir at room temperature for 20 min.

The reaction mixture was poured into a separatory funnel, diluted with DCM (10 mL) and then washed with aqueous HCl (1M, 3 x 10 mL), and then brine (10 mL). The organic layer was dried over anhydrous Na_2SO_4, decanted, and then concentrated *in vacuo* to a clear oil. The oil was then passed through a Pasteur pipette silica gel plug and eluted with DCM (~6 mL). The collected liquid was concentrated *in vacuo* to a clear colorless oil.

Bis(2,2,2-trifluoroethyl) sulfate (**6a**)

^{1}H NMR (300 MHz, CD_2Cl_2) δ 4.62 (q, *J* = 7.7 Hz); **^{13}C{1H} NMR** (75 MHz, CD_2Cl_2) δ 122.1 (q, *J* = 277.4 Hz), 68.3 (q, *J* = 38.8 Hz); **^{19}F NMR** (282 MHz, CD_2Cl_2) δ -75.2 (t, *J* = 7.7 Hz); **IR** (cm^{-1}) (neat): 1414, 1280, 1165, 1022, 960, ; **HRMS-EI** (*m/z*) calcd for $C_3H_4O_4F_3S$ [M – CF_3]$^+$: 192.9782. Found: 192.9776.

Bis(2,2,3,3,3-pentafluoropropyl) sulfate (**6b**)

^{1}H NMR (300 MHz, CD_2Cl_2) δ 4.68 (tq, *J* = 12.1, 1.0 Hz); **^{13}C{1H} NMR** (75 MHz, CD_2Cl_2) δ 118.8 (qt, *J* = 285.7, 34.2 Hz), 111.6 (tq, *J* = 257.2, 38.9 Hz), 67.5 (t, *J* = 28.8 Hz); **^{19}F NMR** (282 MHz, CD_2Cl_2) δ -84.6 (s, 3F), -124.7 (t, *J* = 12.0 Hz, 2F); **IR** (cm^{-1}) (neat): 1429, 1194, 1159, 1021, 998; **HRMS-EI** (*m/z*) calcd for $C_4H_4O_4F_5S$ [M – CF_2CF_3]$^+$: 242.9750. Found: 242.9753.

Studies exploring the effect of base on the reactions between benzyl mercaptan (9a) and trifluoroethyl fluorosulfate (5a)/bis(trifluoroethyl) sulfate (6a)

Scheme S2 – Reaction between trifluoroethyl fluorosulfate (**5a**) and **9a** using DIPEA.

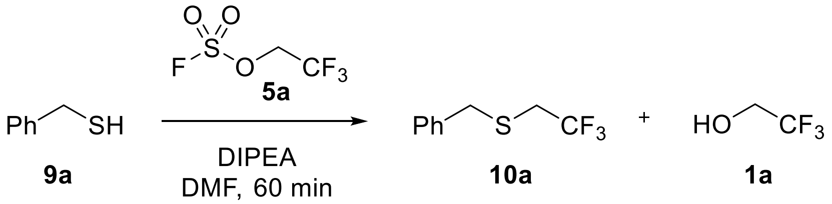

1.5 equiv **5a**: 52% 8%

For studies with trifluoroethyl fluorosulfate (**5a**) and DIPEA: Two 4 mL vials, equipped with magnetic stir-bars, were capped with septum-fitted vial caps and connected by a polyethylene tube. Vial A was charged with SDI (1.35 mmol) and anhydrous KF (3.58 mmol), then the system was placed under N_2 atmosphere. To vial B was added dry DMF (0.84 mL), 2,2,2-trifluoroethanol (0.45 mmol) and DIPEA (0.16 mL, 0.92 mmol). The polyethylene tube in vial B was immersed into the solution and then to vial A was added TFA (0.75 mL) in one portion. Vigorous bubbling of SO_2F_2 and fuming were observed in vial B for 1-2 minutes and when the bubbling subsided, vial B was vented via a needle (this triggered more bubbling of SO_2F_2 through the solution). The tube and needle were then removed and the mixture in vial B was allowed to stir at ambient temperature for 5 minutes. The reaction mixture was degassed with N_2 and the amount of reagent was quantified by ^{19}F NMR spectroscopy using $PhCF_3$ as an internal standard. To the reaction was added a solution of benzyl mercaptan (0.66 equiv) in DMF (0.15 mL). The reaction was stirred at ambient temperature for 60 min and the amount of trifluoroethylated product was 52%, as determined by ^{19}F NMR spectroscopy. The calculated percentage of trifluoroethanol (**1a**) was based on the total amount of benzyl mercaptan used.

Scheme S3 – Reaction between trifluoroethyl fluorosulfate (**5a**) and **9a** using DBU.

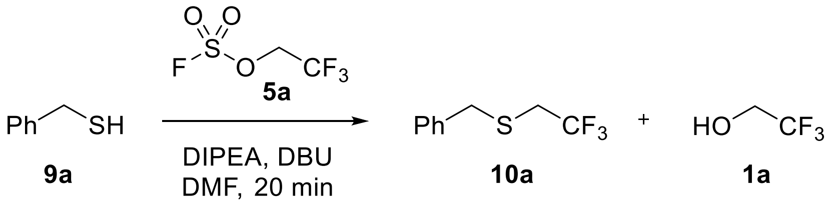

1.1 equiv **5a**: 78% 17%

For studies with trifluoroethyl fluorosulfate (**5a**) and DBU, the procedure for trifluoroethyl fluorosulfate and DIPEA was followed, however, DBU (0) was added to the reaction before benzyl mercaptan (0.89 equiv) was added. After the addition of benzyl mercaptan, the reaction was stirred at ambient temperature for 20 min and the amount of trifluoroethylated product was 78%, as determined by ^{19}F NMR spectroscopy. The calculated percentage of trifluoroethanol (**1a**) was based on the total amount of benzyl mercaptan used.

Scheme S4 – Reaction between bis(trifluoroethyl) sulfate (**6a**) and **9a** using DBU.

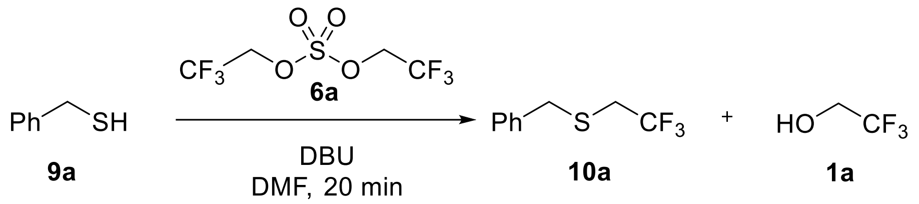

1.5 equiv **6a**: 99% <1%

For studies with bis(trifluoroethyl) sulfate (**6a**) and DBU: Two 4 mL vials, equipped with magnetic stir-bars, were capped with septum-fitted vial caps and connected by a polyethylene tube. Vial A was charged with SDI (0.87 mmol) and anhydrous KF (2.3 mmol), then the system was placed under N_2 atmosphere. To vial B was added dry DMF (0.80 mL), 2,2,2-trifluoroethanol (1.12 mmol) and DBU (1.60 mmol). The polyethylene tube in vial B was immersed into the solution and then to vial A was added TFA (0.50 mL) in one portion. Vigorous bubbling of SO_2F_2 and fuming were observed in vial B for 1-2 minutes and when the bubbling subsided, vial B was vented via a needle (this triggered more bubbling of SO_2F_2 through the solution). The tube and needle were then removed and the mixture in vial B was allowed to stir at ambient temperature for 5 minutes. The reaction mixture was degassed with N_2 and the amount of reagent was quantified by ^{19}F NMR spectroscopy, using $PhCF_3$ as an internal standard. To the reaction was added a solution of benzyl mercaptan (0.66 equiv) in DMF (0.15 mL). The reaction was stirred at ambient temperature for 20 min and the amount of trifluoroethylated product was >99%, as determined by ^{19}F NMR spectroscopy.

Reactivity studies with benzyl mercaptan (9a) and piperidine (12)

Scheme S5 – Competition experiments between benzyl mercaptan (**9a**) and piperidine (**12**) with trifluoroethyl triflate (**S1**), trifluoroethyl fluorosulfate (**5a**), or bis(trifluoroethyl) sulfate (**6a**).

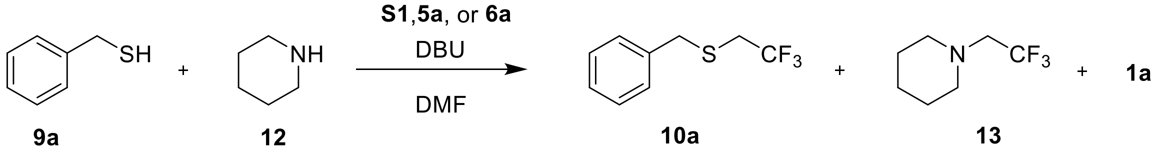

	10a	13	1a
1.1 equiv **S1**:	80%	6%	
1.5 equiv **S1**:	83%	28%	
1.1 equiv **5a**:	75%	3%	16%
1.5 equiv **5a**:	80%	10%	18%
1.1 equiv **6a**:	90%	<3%	<1%
1.5 equiv **6a**:	>97%	<3%	<1%

For studies with trifluoroethyl triflate (**S1**, 1.5 equiv): A 4 mL vial was charged with benzyl mercaptan (0.30 mmol), piperidine (0.30 mmol), and DMF (0.84 mL). To the mixture was added DBU (0.24 mL, 5.3 mmol) and then a solution of trifluoroethyl triflate (**S1**, 0.45 mmol) in DMF (0.15 mL). The reaction was stirred at ambient temperature and the amount of products formed was determined by ^{19}F NMR spectroscopy using $PhCF_3$ as an internal standard.

For studies with trifluoroethyl fluorosulfate (**5a**, 1.5 equiv): Two 4 mL vials, equipped with magnetic stir-bars, were capped with septum-fitted vial caps and connected by a polyethylene tube. Vial A was charged with SDI (1.35 mmol) and anhydrous KF (3.58 mmol), then the system was placed under N_2 atmosphere. To vial B was added dry DMF (0.84 mL), 2,2,2-trifluoroethanol (0.45 mmol) and DIPEA (0.16 mL). The polyethylene tube in vial B was immersed into the solution and then to vial A was added TFA (0.65 mL) in one portion. Vigorous bubbling of SO_2F_2 and fuming were observed in vial B for 1-2 minutes and when the bubbling subsided, vial B was vented via a needle (this triggered more bubbling of SO_2F_2 through the solution). The tube and needle were then removed and the mixture in vial B was allowed to stir at ambient temperature for 5 minutes. The reaction mixture was degassed with N_2 and the amount of reagent was quantified by ^{19}F NMR spectroscopy. To the reaction was added DBU (0.93 mmol), and then a solution of benzyl mercaptan (0.66 equiv) and piperidine (0.66 equiv) in DMF (0.15 mL). The reaction was stirred at ambient temperature and the amount of products formed was determined by ^{19}F NMR spectroscopy using $PhCF_3$ as an internal standard. The calculated percentage of trifluoroethanol (**1a**) was based on the total amount of benzyl mercaptan used.

For studies with bis(trifluoroethyl) sulfate (**6a**, 1.5 equiv): Two 4 mL vials, equipped with magnetic stir-bars, were capped with septum-fitted vial caps and connected by a polyethylene tube. Vial A was charged with SDI (0.87 mmol) and anhydrous KF (2.3 mmol), then the system was placed under N_2 atmosphere. To vial B was added dry DMF (0.84 mL), 2,2,2-trifluoroethanol (1.0 mmol) and DBU (0.30 mL, 2.0 mmol). The polyethylene tube in vial B was immersed into the solution and then to vial A was added TFA (0.50 mL) in one portion. Vigorous bubbling of SO_2F_2 and fuming were observed in vial B for 1-2 minutes and when the bubbling subsided, vial B was vented via a needle (this triggered more bubbling of SO_2F_2 through the solution). The tube and needle were then removed and the mixture in vial B was allowed to stir at ambient temperature for 5 minutes. The reaction mixture was degassed with N_2 and the amount of reagent was quantified by ^{19}F NMR spectroscopy. To the reaction was added a solution of benzyl mercaptan (0.66 equiv) and piperidine (0.66 equiv) in DMF (0.15 mL). The reaction was stirred at ambient temperature and the amount of products formed was determined by ^{19}F NMR spectroscopy using $PhCF_3$ as an internal standard. The calculated percentage of trifluoroethanol (**1a**) was based on the total amount of benzyl mercaptan used.

Scheme S6 - Competition experiments between benzyl mercaptan (**9a**) and piperidine (**12**) with trifluoroethyl fluorosulfate (**5a**) using DIPEA.

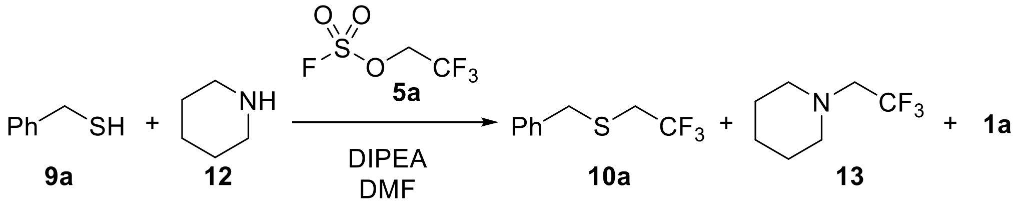

1 equiv **5a**: 37% 30% 5%

For studies with trifluoroethyl fluorosulfate (**5a**, 1.0 equiv): Two 4 mL vials, equipped with magnetic stir-bars, were capped with septum-fitted vial caps and connected by a polyethylene tube. Vial A was charged with SDI (1.35 mmol) and anhydrous KF (3.58 mmol), then the system was placed under N_2 atmosphere. To vial B was added dry DMF (0.84 mL), 2,2,2-trifluoroethanol (0.45 mmol) and DIPEA (0.40 mL, 5.1 equiv). The polyethylene tube in vial B was immersed into the solution

and then to vial A was added TFA (0.65 mL) in one portion. Vigorous bubbling of SO_2F_2 and fuming were observed in vial B for 1-2 minutes and when the bubbling subsided, vial B was vented via a needle (this triggered more bubbling of SO_2F_2 through the solution). The tube and needle were then removed and the mixture in vial B was allowed to stir at ambient temperature for 5 minutes. The reaction mixture was degassed with N_2 and the amount of reagent was quantified by ^{19}F NMR spectroscopy. To the reaction was added a solution of benzyl mercaptan (1 equiv) and piperidine (1 equiv) in DMF (0.15 mL). The reaction was stirred at ambient temperature and the amount of products formed was determined by ^{19}F NMR spectroscopy using $PhCF_3$ as an internal standard. The calculated percentage of trifluoroethanol (**1a**) was based on the total amount of benzyl mercaptan used.

Reactivity studies with benzyl mercaptan (9a) and pyrrolidine (14)

Scheme S7 - Competition experiments between benzyl mercaptan (**9a**) and pyrrolidine (**14**) with bis(trifluoroethyl) sulfate (**6a**) using DBU.

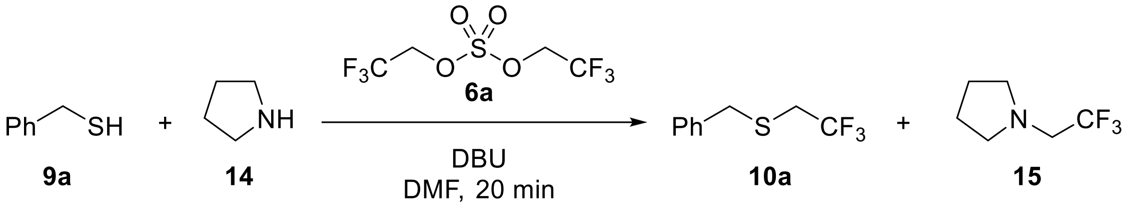

1.0 equiv **6a**: 84% <3%

For studies with bis(trifluoroethyl) sulfate (**6a**, 1.0 equiv): Two 4 mL vials, equipped with magnetic stir-bars, were capped with septum-fitted vial caps and connected by a polyethylene tube. Vial A was charged with SDI (0.87 mmol) and anhydrous KF (2.34 mmol), then the system was placed under N_2 atmosphere. To vial B was added dry DMF (0.80 mL), 2,2,2-trifluoroethanol (1.1 mmol) and DBU (0.26 mL). The polyethylene tube in vial B was immersed into the solution and then to vial A was added TFA (0.48 mL) in one portion. Vigorous bubbling of SO_2F_2 and fuming were observed in vial B for 1-2 minutes and when the bubbling subsided, vial B was vented via a needle (this triggered more bubbling of SO_2F_2 through the solution). The tube and needle were then removed and the mixture in vial B was allowed to stir at ambient temperature for 10 minutes. The amount of reagent was quantified by ^{19}F NMR spectroscopy. To the reaction was added a solution of benzyl mercaptan (1.0 equiv) and pyrrolidine (1.0 equiv) in DMF (0.10 mL). The reaction was stirred at ambient temperature and the amount of products formed was determined by ^{19}F NMR spectroscopy using $PhCF_3$ as an internal standard.

Comments on the reactivity of 5a and 6a

It is important to note that **5a** is much more reactive to amines than **6a**, and both **5a** and **6a** react with thiolates. This suggests that the electrophilic carbon in **5a** is more reactive than the analogous carbon in **6a.**

Reactivity studies with glutathione and trifluoroethyl triflate (S1)

Scheme S8 – Trifluoroethylation of glutathione using trifluoroethyl triflate (**S1)**

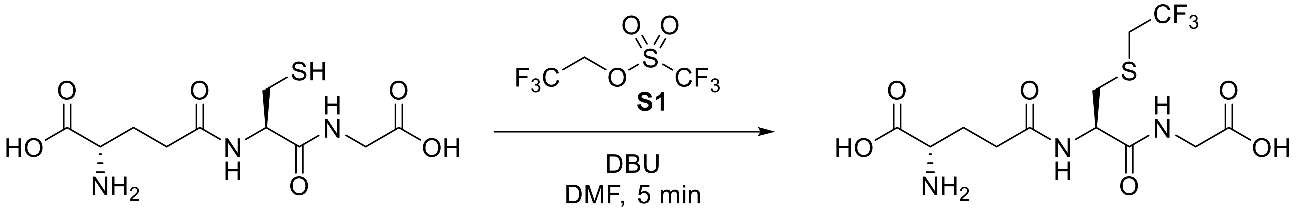

Alkylation using 1.1 equiv of S1:

Trifluoroethyl triflate (76.8 mg, 0.33 mmol, 1.1 equiv) was added to a solution of glutathione (97 mg, 0.3 mmol, 1 equiv), DBU (0.22 mL, 1.5 mmol, 5 equiv), and dimethylformamide (1 mL) at room temperature. α,α,α-Trifluorotoluene (37.6 mg) was added as an internal standard, and the reaction mixture was sampled after five minutes. ^{19}F NMR indicates *S*-alkylation occurred in 66% yield, alongside the formation of unidentified by-products.

^{19}F NMR (282 MHz, $CDCl_3$) δ -67.1 (t, *J* = 10.1 Hz).

Alkylation using 1.5 equiv. of S1

Trifluoroethyl triflate (101 mg, 0.33 mmol, 1.5 equiv) was added to a solution of glutathione (97 mg, 0.3 mmol, 1 equiv), DBU (0.22 mL, 1.5 mmol, 5 equiv), and dimethylformamide (1 mL) at room temperature. α,α,α-Trifluorotoluene (36.2 mg) was added as an internal standard, and the reaction mixture was sampled after five minutes. ^{19}F NMR indicates *S*-alkylation occurred in 50% yield, alongside the formation of unidentified by-products.

^{19}F NMR (282 MHz, $CDCl_3$) δ -67.1 (t, *J* = 10.1 Hz).

General procedure for the one-pot trifluoroethylation of thiols

Scheme S9 – One-pot trifluoroethylation of thiols using sulfuryl fluoride.

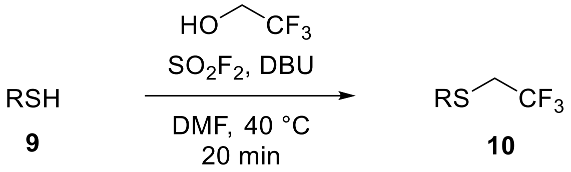

Two 20 mL vials, equipped with magnetic stir-bars, were capped with septum-fitted vial caps and connected by a polyethylene tube. Vial A was charged with SDI (2.89 mmol, 2.89 equiv) and anhydrous KF (7.8 mmol, 7.8 equiv), then the system was placed under N_2 atmosphere. To vial B was added dry DMF (1.47 mL), DBU (5.90 mmol, 5.90 equiv), 2,2,2-trifluoroethanol (TFE; 1.47 mL), and thiol (1.0 mmol, 1 equiv). The reaction vial (B) was heated to 40 °C, the polyethylene tube in vial B was immersed into the solution and then to vial A was added TFA (1.6 mL) in one portion. Vigorous bubbling of SO_2F_2 and fuming were observed in vial B for 2-3 minutes and when the bubbling subsided, vial B was vented via a needle for approximately 1 minute (this triggered more bubbling of SO_2F_2 through the solution). The tube and needle were then removed and the mixture in vial B was allowed to stir at 40 °C for 20 min.

Workup and purification: To the reaction mixture was added aqueous HCl (3M, 10 mL) and the product was extracted with hexanes or pentanes (3 x 10 mL). The combined organic layer was washed sequentially with H_2O (10 mL), NaOH (1M, 10 mL) and then brine (10 mL). The organic layer was dried over anhydrous Na_2SO_4, decanted, and then concentrated *in vacuo*. The resulting residue was purified by silica gel column chromatography. Fractions containing the desired product were combined and concentrated *in vacuo*.

Figure S1 – Setup for the 1,1-dihydrofluoroalkylation using SO_2F_2 generated from SDI, KF, and TFA.

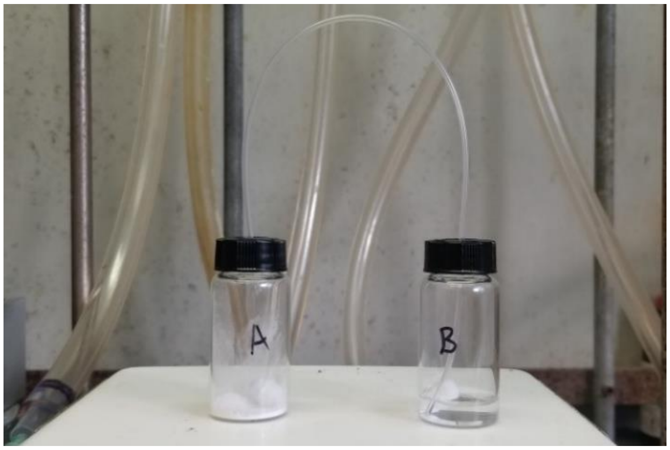

General procedure for the trifluoroethylation of thiols by sequential addition

Shreeve *et al.* have reported that the attack of methanethiol at the sulfur center can presumably form bis(methanethio) sulfate, which can provide the disulfide species by elimination of SO_2.[1] The generation of the bis(trifluoroethyl) sulfate reagent followed by addition of the nucleophile can decrease the amount of disulfide byproduct formed by limiting the interaction of the thiol with more reactive species, such as sulfuryl fluoride.

Scheme S10 – Synthesis of trifluoroethyl sulfides by sequential addition of thiol to bis(trifluoroethyl) sulfate (**6a**).

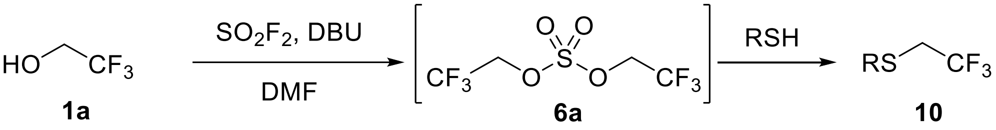

Two 20 mL vials, equipped with magnetic stir-bars, were capped with septum-fitted vial caps and connected by a polyethylene tube. Vial A was charged with SDI (2.89 mmol, 2.89 equiv) and anhydrous KF (7.80 mmol, 7.8 equiv), then the system was placed under N_2 atmosphere. To vial B was added dry DMF (1.47 mL), DBU (5.9 mmol, 5.9 equiv), and 2,2,2-trifluoroethanol (TFE; 1.47 mL). The reaction vial (B) was heated to 40°C, the polyethylene tube in vial B was immersed into the solution and then to vial A was added TFA (1.6 mL) in one portion. Vigorous bubbling of SO_2F_2 and fuming were observed in vial B for 2-3 minutes and when the bubbling subsided, vial B was vented via a needle for approximately 1 minute (this triggered more bubbling of SO_2F_2 through the solution). The tube and needle were then removed and the mixture in vial B was allowed to stir at 40 °C for 5 min. To vial B was added thiol (1.0 mmol, 1 equiv) and the reaction was stirred at 40 °C for 20 min.

General procedure for the synthesis of 2,2,3,3,3-pentafluoropropyl sulfides

Scheme S11 – Synthesis of longer chain 1,1-dihydrofluoroalkyl sulfides using sulfuryl fluoride and 2,2,3,3,3-pentafluoropropanol.

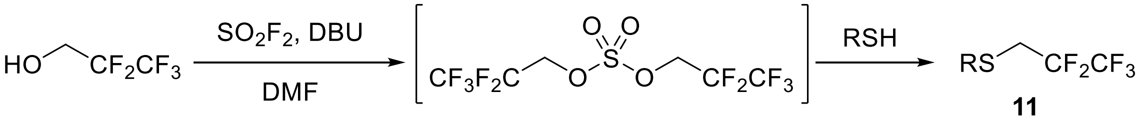

Two 20 mL vials, equipped with magnetic stir-bars, were capped with septum-fitted vial caps and connected by a polyethylene tube. Vial A was charged with SDI (2.89 mmol, 2.89 equiv) and anhydrous KF (7.8 mmol, 7.8 equiv), then the system was placed under N_2 atmosphere. To vial B was added dry DMF (1.90 mL), DBU (5.90 mmol, 5.90 equiv), and 2,2,3,3,3-pentafluoropropanol (PFP; 1.00 mL). The reaction vial (B) was heated to 40°C, the polyethylene tube in vial B was immersed into the solution and then to vial A was added TFA (1.6 mL) in one portion. Vigorous bubbling of SO_2F_2 and fuming were observed in vial B for 2-3 minutes and when the bubbling subsided, vial B was vented via a needle for approximately 1 minute (this triggered more bubbling of SO_2F_2 through the solution). The tube and needle were then removed and the mixture in vial B was allowed to stir at 40 °C for 5 min. To vial B was added thiol (1.0 mmol, 1 equiv) and the reaction was stirred at 40 °C for 30 min.

benzyl(2,2,2-trifluoroethyl)sulfane (10a)

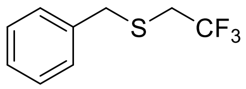

Compound **10a** was prepared on 1.0 mmol scale using the general procedure for one-pot trifluoroethylation of thiols. The general workup procedure followed by silica gel column chromatography with pentanes as eluent afforded the product as a colorless oil (120 mg, 58% yield). The characterization data is consistent with literature values.[2]

^{1}H NMR (300 MHz, $CDCl_3$) δ 7.32 – 7.18 (m, 5H), 3.77 (s, 2H), 2.82 (q, *J* = 10.0 Hz, 2H); **^{13}C{1H} NMR** (75 MHz, $CDCl_3$) δ 136.6, 129.3, 128.9, 127.7, 126.3 (q, *J* = 276.5 Hz), 36.6, 32.6 (q, *J* = 32.7 Hz); **^{19}F NMR** (282 MHz, $CDCl_3$) δ -66.2 (t, *J* = 10.1 Hz).

2-(((2,2,2-trifluoroethyl)thio)methyl)furan (10b)

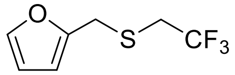

Compound **10b** was prepared on 1.0 mmol scale using the general procedure for one-pot trifluoroethylation of thiols. To the reaction mixture was added aqueous HCl (1M, 5 mL), and the product was extracted with diethyl ether (5 x 7 mL). The combined organics were washed with aqueous NaOH (1M, 3 x 5 mL), dried over anhydrous Na_2SO_4, decanted and concentrated *in vacuo*. The residue was purified by silica gel column chromatography with 3% diethyl ether in pentanes as eluent to afford the product as an oil (128 mg, 60% yield). The product yield has been corrected for 5% disulfide impurities.

^{1}H NMR (300 MHz, $CDCl_3$) δ 7.40 (dd, *J* = 1.9, 0.8 Hz, 1H), 6.33 (dd, *J* = 3.2, 1.9 Hz, 1H), 6.24 (dd, *J* = 3.2, 0.8 Hz, 1H), 3.85 (s, 2H), 3.01 (q, *J* = 9.9 Hz, 2H); **^{13}C{1H} NMR** (75 MHz, $CDCl_3$) δ 150.0, 143.3, 126.4 (q, *J* = 276.4 Hz), 110.9, 109.3, 33.1 (q, *J* = 32.8 Hz), 28.8; **^{19}F NMR** (282 MHz, $CDCl_3$) δ -66.5 (t, *J* = 10.1 Hz); **IR** (cm^{-1}) (neat): 1310, 1237, 1118, 1079, 737; **HRMS-EI** (*m/z*) calcd for $C_7H_7F_3OS$: 196.0170. Found: 196.0169.

decyl(2,2,2-trifluoroethyl)sulfane (10c)

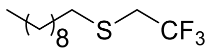

Compound **10c** was prepared on 1.0 mmol scale using the general procedure for one-pot trifluoroethylation of thiols. The general workup procedure followed by flash column chromatography with pentanes as eluent afforded the product as a colorless oil (173 mg, 67% yield).

^{1}H NMR (300 MHz, $CDCl_3$) δ 3.06 (q, *J* = 10.0 Hz, 2H), 2.66 (t, *J* = 7.4 Hz, 2H), 1.64 – 1.54 (m, 2H), 1.40 – 1.34 (m, 2H), 1.31 – 1.26 (m, 12H), 0.88 (t, *J* = 6.9 Hz, 3H); **^{13}C{1H} NMR** (75 MHz, $CDCl_3$) δ 126.1 (q, *J* = 276.2 Hz), 34.5 (q, *J* = 32.7 Hz), 33.4, 32.0, 29.7, 29.6, 29.4, 29.3, 29.2, 28.9, 22.8, 14.3; **^{19}F NMR** (282 MHz, $CDCl_3$) δ -66.8 (t, *J* = 10.2 Hz); **IR** (cm^{-1}) (neat): 2925, 2855, 1309, 1242, 1120, 1078; **HRMS-EI** (*m/z*) calcd for $C_{12}H_{23}F_3S$: 256.1473. Found: 256.1467.

phenethyl(2,2,2-trifluoroethyl)sulfane (10d)

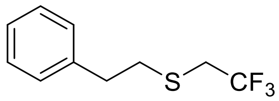

Compound **10d** was prepared on 1.0 mmol scale using the general procedure for one-pot trifluoroethylation of thiols. The general workup procedure followed by silica gel column chromatography with pentanes as eluent afforded the product as a colorless oil (146 mg, 66% yield).

^{1}H NMR (300 MHz, $CDCl_3$) δ 7.38 – 7.22 (m, 5H), 3.07 (q, *J* = 9.9 Hz , 2H), 2.95 (m, 4H); **^{13}C{1H} NMR** (75 MHz, $CDCl_3$) δ 139.8, 128.7, 128.6, 126.7, 126.1 (q, *J* = 276.3 Hz), 35.9, 34.7, 34.6 (q, *J* = 32.6 Hz); **^{19}F NMR** (282 MHz, $CDCl_3$) δ -66.7 (t, *J* = 10.0 Hz); **IR** (cm^{-1}) (neat): 2936, 1116, 1080; **HRMS-EI** (*m/z*) calcd for $C_{10}H_{11}F_3S$: 220.0534. Found: 220.0532.

methyl 2-((2,2,2-trifluoroethyl)thio)acetate (10e)

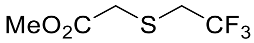

Compound **10e** was prepared on 1.0 mmol scale using the general procedure for one-pot trifluoroethylation of thiols. To the reaction mixture was added aqueous HCl (1M, 10 mL), and the product was extracted with diethyl ether (3 x 10 mL). The combined organics were washed with aqueous HCl (1M, 2 x 10 mL), aqueous saturated $NaHCO_3$ (2 x 10 mL), water (3 x 10 mL), brine (2 x 10 mL), and dried over anhydrous Na_2SO_4, decanted and concentrated *in vacuo*. The residue was purified by silica gel column chromatography with 0 – 5% diethyl ether in pentanes as eluent to afford the product as a colorless oil (116 mg, 62% yield). The product yield has been corrected for 3% solvent impurities.

^{1}H NMR (300 MHz, $CDCl_3$) δ 3.75 (s, 3H), 3.36 (s, 2H), 3.25 (q, *J* = 9.8 Hz, 2H); **^{13}C{1H} NMR** (101 MHz, $CDCl_3$) δ 170.0, 125.9 (q, *J* = 276.4 Hz), 52.7, 34.0 (q, *J* = 33.0 Hz), 33.5; **^{19}F NMR** (282 MHz, $CDCl_3$) δ -66.9 (t, *J* = 9.8 Hz); **IR** (cm^{-1}) (pentanes): 1737, 1261, 1086, 1020; **HRMS-EI** (*m/z*) calcd for $C_5H_7F_3O_2S$: 188.0119. Found: 188.0112.

1,9-bis((2,2,2-trifluoroethyl)thio)nonane (10f)

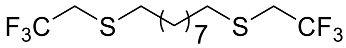

Compound **10f** was prepared on 0.5 mmol scale using the general procedure for one-pot trifluoroethylation of thiols, with 6.90 mmol of DBU. The reaction mixture was added into hexanes and washed with aqueous HCl (1M, 2 x 10 mL). The aqueous layer was back extracted with hexanes (2 x 10 mL). The combined organic layer was washed with saturated aqueous $NaHCO_3$ (10 mL), brine (10 mL) and then dried over anhydrous Na_2SO_4. The solution was decanted and concentrated *in vacuo*. The crude residue was purified by silica gel column chromatography using 0-10% diethyl ether in pentanes. Fractions containing the desired product were combined and concentrated *in vacuo* to afford the product as a colorless oil (130 mg, 73% yield).

^{1}H NMR (300 MHz, $CDCl_3$) δ 3.06 (q, *J* = 10.0 Hz, 4H), 2.66 (t, *J* = 7.4 Hz, 4H), 1.64 – 1.54 (m, 4H), 1.40 – 1.35 (m, 4H), 1.31 – 1.29 (m, 6H); **^{13}C{1H} NMR** (75 MHz, $CDCl_3$) δ 126.1 (q, *J* =

276.3 Hz), 34.5 (q, J = 32.6 Hz), 33.4, 29.4, 29.2, 28.7; **^{19}F NMR** (282 MHz, $CDCl_3$) δ -66.8 (t, J = 9.9 Hz); **IR** (cm^{-1}) (neat): 2929, 2857, 1309, 1240, 1117, 1073; **HRMS-EI** (*m/z*) calcd for $C_{13}H_{22}F_6S_2$: 356.1067. Found: 356.1071.

(2,2,2-trifluoroethyl)(trityl)sulfane (10h)

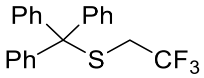

Compound **10h** was prepared on 1.0 mmol scale using the general procedure for one-pot trifluoroethylation of thiols. The reaction was diluted with H_2O (12 mL) and then extracted with diethyl ether (3 x 10 mL). The ether layer was washed with saturated aqueous $NaHCO_3$ (10 mL), dried over anhydrous Na_2SO_4, decanted, and then concentrated *in vacuo*. The crude solid was purified by silica gel column chromatography with toluene in hexanes (10-100% gradient) as eluent. Fractions containing the desired product were combined and concentrated to dryness *in vacuo* to afford the product as a white solid (258 mg, 72% yield).

^{1}H NMR (300 MHz, $CDCl_3$) δ 7.44 – 7.39 (m, 6H), 7.35 – 7.23 (m, 9H), 2.77 (q, J = 10.1 Hz, 2H); **^{13}C{1H} NMR** (75 MHz, $CDCl_3$) δ 143.8, 129.6, 128.3, 127.3, 125.4 (m), 67.6, 34.7 (q, J = 31.5 Hz); **^{19}F NMR** (282 MHz, $CDCl_3$) δ -64.6 (t, J = 9.8 Hz); **IR** (cm^{-1}) (neat): 1594, 1486, 1305, 1224, 1130, 1050; **HRMS-EI** (*m/z*) calcd for $C_{15}H_{12}F_3S$ $[M - Ph]^+$: 281.0612. Found: 281.0604.

phenyl(2,2,2-trifluoroethyl)sulfane (10i)

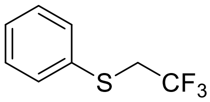

Compound **10i** was prepared on 1.0 mmol scale using the general procedure for one-pot trifluoroethylation of thiols. The general workup procedure followed by flash column chromatography with pentanes as eluent afforded the product as a colorless oil (152 mg, 79% yield). Product ^{1}H NMR data is consistent with literature data.[3]

^{1}H NMR (300 MHz, $CDCl_3$) δ 7.51 – 7.48 (m, 2H), 7.34 – 7.31 (m, 2H), 3.44 (q, J = 9.7 Hz, 2H); **^{13}C{1H} NMR** (75 MHz, $CDCl_3$) δ 133.8, 132.0, 129.4, 128.2, 125.5 (q, J = 276.4 Hz), 38.2 (q, J = 32.6 Hz); **^{19}F NMR** (282 MHz, $CDCl_3$) δ -66.7 (t, J = 9.8 Hz); **IR** (cm^{-1}) (neat): 3066, 2950, 1584, 1240, 1119, 1079, 740; **HRMS-EI** (*m/z*) calcd for $C_8H_7F_3S$: 192.0221. Found: 192.0223.

(4-methoxyphenyl)(2,2,2-trifluoroethyl)sulfane (10j)

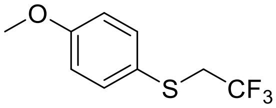

Compound **10j** was prepared on 1.0 mmol scale using the general procedure for one-pot trifluoroethylation of thiols. To the reaction mixture was added aqueous HCl (1M, 7 mL), and the product was extracted with pentanes (6 x 10 mL). The combined organic layer was washed with aqueous HCl (1M, 3 x 10 mL), aqueous NaOH (1M, 3 x 10 mL), brine (10 mL), dried over

anhydrous Na_2SO_4, decanted and concentrated *in vacuo*. The residue was purified by silica gel column chromatography with 10% ethyl acetate in pentanes as eluent to afford the product as an oil (171 mg, 78% yield). The characterization data is consistent with literature values.[2]

^{1}H NMR (300 MHz, $CDCl_3$) δ 7.50 – 7.45 (m, 2H), 6.89 – 6.84 (m, 2H), 3.81 (s, 3H), 3.32 (q, *J* = 9.8 Hz, 2H); **^{13}C{1H} NMR** (75 MHz, $CDCl_3$) δ 160.3, 135.5, 125.7 (q, *J* = 276.5 Hz), 124.2, 114.9, 55.5, 39.7 (q, *J* = 32.0 Hz); **^{19}F NMR** (282 MHz, $CDCl_3$) δ -66.7 (t, *J* = 9.8 Hz).

N-(4-((2,2,2-trifluoroethyl)thio)phenyl)acetamide (10k)

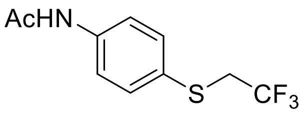

Compound **10k** was prepared on 1.0 mmol scale using the general procedure for one-pot trifluoroethylation of thiols. The general workup procedure, using ethyl acetate instead of hexanes/pentanes, followed by silica gel column chromatography with 50% ethyl acetate in hexanes as the eluent afforded the product as a white solid (150 mg, 60% yield).

^{1}H NMR (300 MHz, CD_2Cl_2) δ 7.52 – 7.44 (m, 5H), 3.42 (q, *J* = 9.8 Hz , 2H), 2.13 (s, 3H); **^{13}C{1H} NMR** (75 MHz, CD_2Cl_2) δ 169.0, 139.1, 134.0, 128.6, 126.2 (q, *J* = 276.3 Hz), 120.8, 39.2 (q, *J* = 32.2 Hz), 24.9; **^{19}F NMR** (282 MHz, CD_2Cl_2) δ -67.2 (t, *J* = 9.8 Hz); **IR** (cm^{-1}) (neat): 3287, 1659, 1541, 1108, 1080; **HRMS-EI** (*m/z*) calcd for $C_{10}H_{10}F_3NOS$: 249.0435. Found: 249.0435.

(4-chlorophenyl)(2,2,2-trifluoroethyl)sulfane (10l)

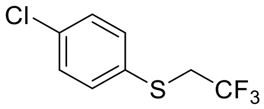

Compound **10l** was prepared on 1.0 mmol scale using the general procedure for one-pot trifluoroethylation of thiols. To the reaction mixture was added aqueous HCl (1M, 10 mL) over ice, and the product was extracted with diethyl ether (3 x 10 mL). The combined organics were washed with aqueous HCl (1M, 2 x 10 mL), aqueous NaOH (1M, 2 x 10 mL), water (3 x 10 mL), brine (2 x 10 mL), and dried over anhydrous Na_2SO_4, decanted and concentrated *in vacuo*. The residue was purified by silica gel column chromatography with pentanes as eluent to afford the product as a colorless oil (133 mg, 57% yield).

^{1}H NMR (300 MHz, $CDCl_3$) δ 7.46 – 7.40 (m, 2H), 7.34 – 7.28 (m, 2H), 3.41 (q, *J* = 9.6 Hz, 2H); **^{13}C{1H} NMR** (101 MHz, $CDCl_3$) δ 134.6, 133.5, 132.2, 129.6, 124.0 (q, *J* = 276.4 Hz), 38.4 (q, *J* = 32.9 Hz).; **^{19}F NMR** (282 MHz, $CDCl_3$) δ -66.7 (t, *J* = 9.4 Hz); **IR** (cm^{-1}) (neat): 1575, 1477, 1309, 1122, 1076, 817; **HRMS-EI** (*m/z*) calcd for $C_8H_6ClF_3S$: 225.9831. Found: 225.9832.

(2,2,2-trifluoroethyl)(4-(trifluoromethyl)phenyl)sulfane (10m)

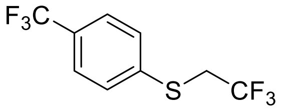

Compound **10m** was prepared on 1.0 mmol scale using the general procedure for one-pot trifluoroethylation of thiols. The reaction mixture was diluted with H_2O (10 mL) and the product was extracted with diethyl ether (3 x 10 mL). The combined organic layer was washed sequentially with aqueous HCl (3M, 10 mL), H_2O (10 mL), aqueous NaOH (1M, 10 mL), and then brine (10 mL). The organic layer was dried over anhydrous Na_2SO_4, decanted, and then concentrated *in vacuo*. The resulting residue was purified by silica gel column chromatography eluting with pentanes. Fractions containing product were combined and concentrated *in vacuo* to afford the product as a colorless oil (217 mg, 80% yield). The product's NMR spectra matches literature spectra.[4]

^{1}H NMR (300 MHz, $CDCl_3$) δ 7.63 – 7.49 (m, 4H), 3.52 (q, *J* = 9.5 Hz, 2H); **^{13}C{1H} NMR** (75 MHz, $CDCl_3$) δ 138.9, 130.4, 129.9 (m), 126.2 (q, *J* = 3.7 Hz), 125.2 (q, *J* = 276.6 Hz), 124.0 (q, *J* = 272.2 Hz), 37.0 (q, *J* = 33.4 Hz); **^{19}F NMR** (282 MHz, $CDCl_3$) δ -63.1 (s, 3F), -66.6 (t, *J* = 9.7 Hz, 3F).

(3-fluorophenyl)(2,2,2-trifluoroethyl)sulfane (10n)

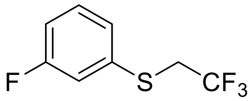

Compound **10n** was prepared on 1.0 mmol scale using the general procedure for one-pot trifluoroethylation of thiols. The general workup procedure followed by silica gel column chromatography with pentanes as eluent afforded the product as a colorless oil (128 mg, 61% yield). The product's NMR spectra matches literature spectra.[4]

^{1}H NMR (300 MHz, $CDCl_3$) δ 7.35 – 7.22 (m, 2H), 7.22 – 7.15 (m, 1H), 7.04 – 6.95 (m, 1H), 3.47 (q, *J* = 9.6 Hz, 2H); **^{13}C{1H} NMR** (75 MHz, $CDCl_3$) δ 162.8 (d, *J* = 249.3 Hz), 136.0 (d, *J* = 7.8 Hz), 130.7 (d, *J* = 8.5 Hz), 126.9 (d, *J* = 3.2 Hz), 125.3 (q, *J* = 276.4 Hz), 118.2 (d, *J* = 22.8 Hz), 115.2 (d, *J* = 21.1 Hz), 37.8 (q, *J* = 33.1 Hz); **^{19}F NMR** (282 MHz, $CDCl_3$) δ -66.7 (t, *J* = 9.7 Hz, 3F), -111.7 – -111.8 (m, 1F).

(3-chlorophenyl)(2,2,2-trifluoroethyl)sulfane (10o)

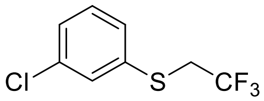

Compound **10o** was prepared on 1.0 mmol scale using the general procedure for one-pot trifluoroethylation of thiols. To the reaction mixture was added aqueous HCl (1M, 10 mL) over ice, and the product was extracted with diethyl ether (3 x 10 mL). The combined organics were washed with aqueous HCl (1M, 2 x 10 mL), aqueous NaOH (1M, 2 x 10 mL), water (3 x 10 mL), brine (2 x 10 mL), and dried over anhydrous Na_2SO_4, decanted and concentrated *in vacuo*. The residue

was purified by silica gel column chromatography with pentanes as eluent to afford the product as a colorless oil (109 mg, 48% yield).

^{1}H NMR (300 MHz, $CDCl_3$) δ 7.47 (dd, *J* = 2.1, 0.8 Hz, 1H), 7.36 (ddd, *J* = 5.7, 3.0, 1.8 Hz, 1H), 7.30 – 7.22 (m, 2H), 3.45 (q, *J* = 9.6 Hz, 2H); **^{13}C{1H} NMR** (101 MHz, $CDCl_3$) δ 135.7, 135.0, 131.2, 130.4, 129.6, 128.3, 125.3 (q, *J* = 276.5 Hz), 37.8 (q, *J* = 33.0 Hz); **^{19}F NMR** (282 MHz, $CDCl_3$) δ -66.7 (t, *J* = 9.7 Hz); **IR** (cm^{-1}) (neat): 1578, 1463, 1309, 1121, 1074; **HRMS-EI** (*m/z*) calcd for $C_8H_6ClF_3S$: 225.9831. Found: 225.9833.

(2-fluorophenyl)(2,2,2-trifluoroethyl)sulfane (10p)

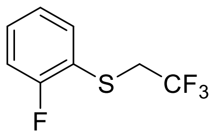

Compound **10p** was prepared on 1.0 mmol scale using the general procedure for one-pot trifluoroethylation of thiols. The general workup procedure followed by silica gel column chromatography with pentanes as eluent afforded the product as a colorless oil (168 mg, 80% yield).

^{1}H NMR (300 MHz, $CDCl_3$) δ 7.54 (td, *J* = 7.7, 1.9 Hz, 1H), 7.38 – 7.30 (m, 1H), 7.15 – 7.08 (m, 2H), 3.43 (q, *J* = 9.6 Hz, 2H); **^{13}C{1H} NMR** (75 MHz, $CDCl_3$) δ 162.5 (d, *J* = 247.1 Hz), 135.2, 131.0 (d, *J* = 8.1 Hz), 125.4 (m), 124.9 (d, *J* = 4.9 Hz), 120.2 (d, *J* = 17.5 Hz), 116.3 (*J* = 22.6 Hz), 36.9 (qd, *J* = 32.8, 3.4 Hz); **^{19}F NMR** (282 MHz, $CDCl_3$) δ -66.9 (t, *J* = 9.5 Hz, 3F), -108.5 (q, *J* = 7.6 Hz, 1F); **IR** (cm^{-1}) (neat): 1596, 1120, 1081, 1029; **HRMS-EI** (*m/z*) calcd for $C_8H_6F_4S$: 210.0126. Found: 210.0126.

o-tolyl(2,2,2-trifluoroethyl)sulfane (10q)

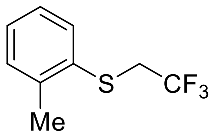

Compound **10q** was prepared on 1.0 mmol scale using the general procedure for one-pot trifluoroethylation of thiols. The general workup procedure followed by silica gel column chromatography with pentanes as eluent afforded the product as a colorless oil (180 mg, 83% yield). The yield has been corrected for 4% disulfide impurities.

^{1}H NMR (300 MHz, $CDCl_3$) δ 7.54 – 7.52 (m, 1H), 7.29 – 7.25 (m, 2H), 7.24 – 7.19 (m, 1H), 3.43 (q, *J* = 9.7 Hz , 2H), 2.52 (s, 3H); **^{13}C{1H} NMR** (75 MHz, $CDCl_3$) δ 140.2, 132.9, 132.5, 130.7, 128.4, 127.0, 125.6 (q, *J* = 276.5 Hz), 37.6 (q, *J* = 32.6 Hz), 20.7; **^{19}F NMR** (282 MHz, $CDCl_3$) δ -66.6 (t, *J* = 9.7 Hz); **IR** (cm^{-1}) (neat): 2939, 1118, 1078; **HRMS-EI** (*m/z*) calcd for $C_9H_9F_3S$: 206.0377. Found: 206.0380.

6-((2,2,2-trifluoroethyl)thio)hexan-1-ol (10r)

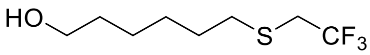

Compound **10r** was prepared on 1.0 mmol scale using the general procedure for one-pot trifluoroethylation of thiols. The reaction mixture was added to aqueous HCl (3M, 10 mL) in a separatory funnel and the product was extracted with diethyl ether (3 x 10 mL). The combined organic layer was washed sequentially with H_2O (5 mL), aqueous NaOH (1M, 10 mL), and the brine (10 mL). The organic layer was dried over anhydrous Na_2SO_4, decanted, and concentrated *in vacuo*. The crude residue was purified by silica gel column chromatography eluting with diethyl ether in pentanes (60-80% gradient). Fractions containing the desired product were combined and concentrated *in vacuo* to afford the desired product as colorless oil (170 mg, 76%).

^{1}H NMR (300 MHz, $CDCl_3$) δ 3.64 (t, *J* = 6.5 Hz, 2H), 3.06 (q, *J* = 10.0 Hz, 2H), 2.66 (t, *J* = 7.3 Hz, 2H), 1.66 – 1.52 (m, 4H), 1.43 – 1.37 (m, 4H); **^{13}C{1H} NMR** (75 MHz, $CDCl_3$) δ 126.1 (q, *J* = 276.2 Hz), 62.9, 34.5 (q, *J* = 32.6 Hz), 33.3, 32.7, 29.1, 28.5, 25.4; **^{19}F NMR** (282 MHz, $CDCl_3$) δ -66.8 (t, *J* = 9.8 Hz); **IR** (cm^{-1}) (neat): 3346, 2933, 2860, 1310, 1241, 1118, 1068; **HRMS-EI** (*m/z*) calcd for $C_8H_{15}F_3OS$: 216.0796. Found: 216.0792.

2-((2,2,2-trifluoroethyl)thio)acetic acid (10s)

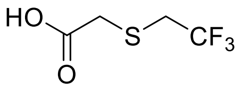

Compound **10s** was prepared on 1.0 mmol scale using the general procedure for trifluoroethylation of thiols by sequential addition, with 6.70 mmol of DBU. The reaction mixture was diluted with H_2O (10 mL) and washed with diethyl ether:hexanes (1:1 v/v, 2 x 10 mL). The aqueous layer was acidified with aqueous HCl (1M) to a pH ~1 and the product was extracted with ethyl acetate (3 x 10 mL). The organic later was dried over anhydrous Na_2SO_4, decanted, and concentrated *in vacuo*. The crude residue was purified by silica gel column chromatography using 5% methanol in dichloromethane with 0.1% formic acid as eluent. Fractions containing the desired product were concentrated *in vacuo*. To remove some of the residual solvent present, the product was taken up in ethyl acetate and washed with aqueous HCl (0.1M, 10 mL), H_2O (2 x 10 mL), and then 50% brine (10 mL). The organic layer was dried over anhydrous Na_2SO_4, filtered, and then concentrated *in vacuo* to afford the product as a clear, slightly yellow oil (131 mg, 75% yield).

^{1}H NMR (300 MHz, $CDCl_3$) δ 3.42 (s, 2H), 3.28 (q, *J* = 9.7 Hz, 2H); **^{13}C{1H} NMR** (75 MHz, $CDCl_3$) δ 176.0, 125.8 (q, *J* = 276.3 Hz), 34.0 (q, *J* = 33.1 Hz), 33.5; **^{19}F NMR** (282 MHz, $CDCl_3$) δ -66.7 (t, *J* = 9.8 Hz); **IR** (cm^{-1}) (neat): 2986 (br), 1710, 1413, 1313, 1245, 1120, 1076; **HRMS-ESI** (*m/z*) calcd for $C_4H_4F_3O_2S$ $[M – H]^-$: 172.9884. Found: 172.9885.

4-((2,2,2-trifluoroethyl)thio)aniline (10t)

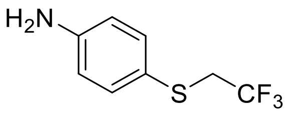

Compound **10t** was prepared on 1.0 mmol scale using the general procedure for one-pot trifluoroethylation of thiols. The reaction mixture was then acidified with aqueous HCl (3M, 10 mL) and washed with hexanes (2 x 5 mL). The aqueous layer was basified with saturated aqueous $NaHCO_3$, and extracted with Et_2O (3 x 10 mL). The combined organic layer was washed with H_2O (10 mL), brine (10 mL), and then dried over anhydrous Na_2SO_4, decanted, and concentrated *in vacuo* to a brown oil. The crude residue was purified by silica gel column chromatography using ethyl acetate in hexanes with triethylamine (2.1:7:0.9). Fractions containing the desired product were combined and concentrated *in vacuo* to afford the product as a yellow-brown oil (152 mg, 73% yield).

^{1}H NMR (300 MHz, $CDCl_3$) δ 7.37 – 7.32 (m, 2H), 6.65 – 6.60 (m, 2H), 3.75 (br s, 2H), 3.27 (q, *J* = 9.9 Hz, 2H); **^{13}C{1H} NMR** (75 MHz, $CDCl_3$) δ 147.4, 135.9, 125.7 (q, *J* = 276.4 Hz), 121.0, 115.7, 40.1 (q, *J* = 31.6 Hz); **^{19}F NMR** (282 MHz, $CDCl_3$) δ -66.7 (t, *J* = 10.1 Hz); **IR** (cm^{-1}) (neat): 3475, 3377, 1621, 1597, 1496, 1305, 1237, 1114, 1072; **HRMS-ESI** (*m/z*) calcd for $C_8H_9F_3NS$ $[M + H]^+$: 208.0408. Found: 208.0409.

4-((2,2,2-trifluoroethyl)thio)pyridine (10u)

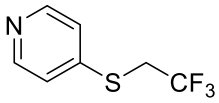

Compound **10u** was prepared on 1.0 mmol scale using the general procedure for one-pot trifluoroethylation of thiols stirring for 2 hours instead of 20 minutes. The reaction mixture was diluted with DCM (5 mL) and the product was extracted with aqueous HCl (3M, 3 x 5 mL). The aqueous layer was basified with aqueous NaOH (1M) until pH ~8. The product was extracted with ethyl acetate (5 x 5 mL). The combined organic layer was washed with brine (3 x 10 mL) and then concentrated *in vacuo*. The residue was purified by silica gel column chromatography using 50% ethyl acetate in pentanes with 1% triethylamine. Fractions containing the product were combined and concentrated *in vacuo* to afford the product as a yellow oil (121 mg, 60% yield).

^{1}H NMR (300 MHz, $CDCl_3$) δ 8.48 – 8.45 (m, 2H), 7.22 – 7.19 (m, 2H), 3.57 (q, *J* = 9.4 Hz, 2H); **^{13}C{1H} NMR** (75 MHz, $CDCl_3$) δ 149.9, 145.7, 125.0 (q, *J* = 276.6 Hz), 121.8, 34.2 (q, *J* = 34.1 Hz); **^{19}F NMR** (282 MHz, $CDCl_3$) δ -66.5 (t, *J* = 9.4 Hz); **IR** (cm^{-1}) (neat): 2942, 1240, 1125, 1076; **HRMS-ESI** (*m/z*) calcd for $C_7H_7F_3NS$ $[M + H]^+$: 194.0251. Found: 194.0249.

ethyl (*S*)-2-amino-2-((2,2,2-trifluoroethyl)thio)acetate (10v)

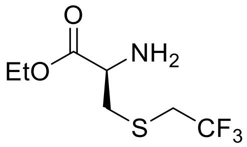

Compound **10v** was prepared on 1.0 mmol scale using the general procedure for trifluoroethylation of thiols by sequential addition, starting from the L-cysteine ethyl ester hydrochloride salt. The reaction mixture was diluted in H_2O (20 mL), and then extracted with ethyl acetate (5 x10 mL). The combined organic layers were washed with H_2O (5 x 10 mL), brine (10 mL), and then dried over anhydrous $MgSO_4$, decanted, and concentrated *in vacuo*. The crude residue was taken up in diethyl ether (1 mL), and then acidified using HCl in diethyl ether (2 M, 2.0 mL). The solution was sparged with argon gas, concentrated *in vacuo*, and the resulting solid was filtered and washed with cold diethyl ether. The salt was then free-based by using K_2CO_3 in ethanol, and then stirred for 10 minutes at ambient temperature. The neutralized product was flushed through a pad of basic alumina using ethyl acetate, and then concentrated *in vacuo* to afford the product as a slight yellow oil (152 mg, 63% yield).

^{1}H NMR (300 MHz, $CDCl_3$) δ 4.21 (q, *J* = 7.1 Hz, 2H), 3.68 (dd, *J* = 7.2, 4.8 Hz, 1H), 3.19 (q, *J* = 9.9 Hz, 2H), 3.03 (dd, *J* = 13.5, 4.8 Hz, 1H), 2.93 (dd, *J* = 13.5, 7.2 Hz, 1H), 1.70 (s, 2H), 1.29 (t, *J* = 7.1 Hz, 3H); **^{13}C{1H} NMR** (75 MHz, $CDCl_3$) δ 173.7, 125.9 (q, *J* = 276.3 Hz), 61.6, 54.7, 37.9, 35.0 (q, *J* = 32.8 Hz), 14.3; **^{19}F NMR** (282 MHz, $CDCl_3$) δ -67.0 (t, *J* = 9.8 Hz); **IR** (cm^{-1}) (neat): 3313, 2939, 1732, 1309, 1239, 1121, 1076; **HRMS-EI** (*m/z*) calcd for $C_7H_{11}NO_2F_3S$ $[M - H]^+$: 230.0463. Found: 230.0451.

***N*^{5}-((*R*)-1-((carboxymethyl)amino)-1-oxo-3-((2,2,2-trifluoroethyl)thio)propan-2-yl)-*L*-glutamine (10w)**

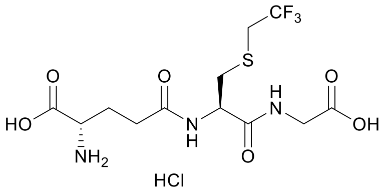

Compound **10w** was prepared on 1.0 mmol scale using the general procedure for one-pot trifluoroethylation of thiols by sequential addition. Two extra equivalents of DBU (0.30 mL) were added to the reaction mixture before the commercially available glutathione (reduced) was added. The reaction was stirred at 40 °C for 20 minutes and then diluted in H_2O (15 mL) and washed with diethyl ether (2 x 10 mL). The aqueous layer was acidified to pH 2-3 using aqueous HCl (3M) and then aqueous NaOH (1M) was added until a pH ~6. The aqueous layer was concentrated *in vacuo* to a white residue and placed under high vacuum for 12 hours. The crude residue was triturated in acetonitrile (40 mL) and then the solid was collected and triturated in 2-propanol (40 mL). The sticky solid was collected and placed under high vacuum for 1 hour. The solid was taken up in ethanol (30 mL) and then acidified to pH ~1 using aqueous HCl (3M). The solvent was removed *in vacuo* and the residue was taken up in ethanol (70 mL) and then filtered. The filtrate was concentrated *in vacuo* to give the product as a white sticky solid (252 mg, 59% yield). The product yield has been corrected for 12% ethanol impurities.

^{1}**H NMR** (300 MHz, D_2O) δ 4.62 (dd, *J* = 8.6, 5.3 Hz , 1H), 4.09 (t, *J* = 6.6 Hz, 1H), 4.01 (s, 2H), 3.35 (qd, *J* = 10.2, 2.5 Hz, 2H), 3.20 (dd, *J* = 14.0, 5.2 Hz, 1H), 3.02 (dd, *J* = 14.0, 8.6 Hz, 1H), 2.69 – 2.51 (m, 2H), 2.35 – 2.13 (m, 2H); ^{13}**C{1H} NMR** (75 MHz, D_2O) δ 174.6, 173.0, 172.6, 171.7, 126.1 (q, *J* = 275.4 Hz), 53.1, 52.4, 41.3, 34.0, 33.7 (m), 31.1, 25.6; ^{19}**F NMR** (282 MHz, D_2O) δ -66.9 (t, *J* = 9.9 Hz); **IR** (cm^{-1}) (neat): 3360, 3035 (br), 2967, 1677, 1643, 1311, 1115, 1077; **HRMS-ESI** (*m/z*) calcd for $C_{12}H_{19}F_3O_6N_3S$ $[M + H]^+$: 390.0947. Found: 390.0945.

benzyl(2,2,3,3,3-pentafluoropropyl)sulfane (11a)

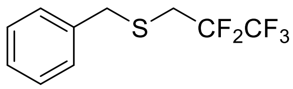

Compound **11a** was prepared on 1.0 mmol scale using the general procedure for the synthesis of 2,2,3,3,3-pentafluoropropyl sulfides. The reaction mixture was diluted with H_2O (12 mL) and then extracted with pentanes (3 x 10 mL) and diethyl ether (1 x 10 mL). The combined organic layer was washed with aqueous HCl (1M, 2 x 10 mL), aqueous NaOH (1M, 10 mL), H_2O (10 mL), brine (10 mL), and then dried over anhydrous Na_2SO_4, decanted, and then concentrated *in vacuo*. The residue was purified by silica gel flash column chromatography with pentanes as eluent. Fractions containing the product were combined and concentrated *in vacuo* to afford the product as a colorless oil (231 mg, 90% yield).

^{1}**H NMR** (300 MHz, $CDCl_3$) δ 7.40 – 7.28 (m, 5H), 3.86 (s, 2H), 2.89 (t, *J* = 17.1 Hz, 2H); ^{13}**C{1H} NMR** (75 MHz, $CDCl_3$) δ 136.5, 129.4, 128.9, 127.8, 119.1 (qt, *J* = 286.4, 36.4 Hz), 114.9 (tq, *J* = 251.8, 37.0 Hz), 37.1 (t, *J* = 1.6 Hz), 30.3 (t, *J* = 24.1 Hz); ^{19}**F NMR** (282 MHz, $CDCl_3$) δ -84.9 (s, 3F), -116.8 (t, *J* = 17.2 Hz, 2F); **IR** (cm^{-1}) (neat): 3033, 1495, 1189, 1078, 1016; **HRMS-EI** (*m/z*) calcd for $C_{10}H_9F_5S$: 256.0345. Found: 256.0348.

decyl(2,2,3,3,3-pentafluoropropyl)sulfane (11c)

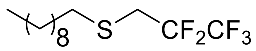

Compound **11c** was prepared on 1.0 mmol scale using the general procedure for the synthesis of 2,2,3,3,3-pentafluoropropyl sulfides. The reaction mixture was diluted with H_2O (15 mL) and the product was extracted with hexanes (3 x 10 mL). The combined organic layer was washed with aqueous HCl (1M, 2 x 10 mL), aqueous NaOH (1M, 1 x 10 mL), H_2O (10 mL), brine (10 mL) and then dried over anhydrous Na_2SO_4. The solution was decanted and then concentrated *in vacuo* to a yellow oil. The residue was purified by silica gel column chromatography with pentanes as eluent to afford the product as a colorless oil (273 mg, 89% yield).

^{1}**H NMR** (300 MHz, $CDCl_3$) δ 3.12 – 3.00 (m, 2H), 2.67 (t, *J* = 7.4 Hz, 2H), 1.65 – 1.57 (m, 2H), 1.46 – 1.33 (m, 2H), 1.27 (br s, 12H), 0.91 – 0.85 (m, 3H); ^{13}**C{1H} NMR** (75 MHz, $CDCl_3$) δ 119.2 (qt, *J* = 286.5, 36.6 Hz), 114.6 (tq, *J* = 251.4, 36.8 Hz), 34.0, 32.4 (t, *J* = 24.2 Hz), 32.1, 29.7, 29.7, 29.5, 29.3, 28.8, 22.9, 14.1; ^{19}**F NMR** (282 MHz, $CDCl_3$) δ -84.9 (s, 3F), -117.3 (t, *J* = 17.0 Hz, 2F); **IR** (cm^{-1}) (neat): 2926, 2856, 1191, 1079, 1018; **HRMS-EI** (*m/z*) calcd for $C_{13}H_{23}F_5S$: 306.1441. Found: 306.1436.

6-((2,2,3,3,3-pentafluoropropyl)thio)hexan-1-ol (11r)

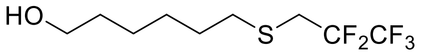

Compound **11r** was prepared on 1.05 mmol scale using the general procedure for one-pot trifluoroethylation of thiols, 2,2,3,3,3-pentafluoropropanol was used instead of 2,2,2-trifluoroethanol and the reaction stirred for 2 hours. The reaction mixture was diluted with aqueous HCl (3M, 10 mL) and the product was extracted with diethyl ether (3 x 10 mL). The combined organic layer was washed with aqueous HCl (1M, 10 mL) and H_2O (10 mL). The aqueous layer was back extracted with diethyl ether (10 mL). The combined organic layer was washed with aqueous NaOH (1M, 2 x 10 mL), brine (10 mL), and then dried over anhydrous Na_2SO_4. The solution was decanted and then concentrated *in vacuo.* The residue was purified by silica gel column chromatography with 60-80% diethyl ether in pentanes as eluent. Fractions containing the product were combined and concentrated *in vacuo* to afford the product as a colorless oil (186 mg, 70% yield).

^{1}H NMR (300 MHz, $CDCl_3$) δ 3.64 (t, *J* = 6.5 Hz, 2H), 3.06 (t, *J* = 17.0 Hz, 2H), 2.68 (t, *J* = 7.3, Hz, 2H) 1.67 – 1.53 (m, 4H), 1.44 – 1.38 (m, 4H); **^{13}C{1H} NMR** (75 MHz, $CDCl_3$) δ 119.1 (qt, *J* = 286.4, 36.3 Hz), 114.5 (tq, *J* = 251.6, 36.8 Hz), 62.9, 33.8 (t, *J* = 1.3 Hz), 32.7, 32.4 (t, J = 24.2 Hz), 29.2, 28.5, 25.4; **^{19}F NMR** (282 MHz, $CDCl_3$) δ -84.9 (s, 3F), -117.4 (t, *J* = 17.0 Hz, 2F); **IR** (cm^{-1}) (neat): 3346, 2934, 2861, 1187, 1073, 1017; **HRMS-EI** (*m/z*) calcd for $C_9H_{13}F_5S$ $[M - H_2O]^+$: 248.0658. Found: 248.0663.

4-((2,2,3,3,3-pentafluoropropyl)thio)aniline (11t)

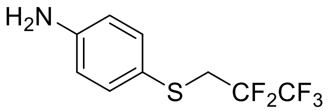

Compound **11t** was prepared on 1.0 mmol scale using the general procedure for the synthesis of 2,2,3,3,3-pentafluoropropyl sulfides. The reaction mixture was diluted with aqueous HCl (3M, 10 mL) and washed with hexanes (3 x 10 mL). The aqueous layer was basified with saturated aqueous $NaHCO_3$ and extracted with diethyl ether (4 x 20 mL). The combined organic layer was washed with aqueous NaOH (1M, 3 x 20 mL), H_2O (3 x 15 mL), brine (2 x 15 mL), and then dried over anhydrous Na_2SO_4. The solution was decanted and concentrated *in vacuo*. The residue was purified by silica gel column chromatography using 30% ethyl acetate in hexanes with 2% Et_3N as eluent to afford the product as a yellow oil (162 mg, 63% yield).

^{1}H NMR (300 MHz, $CDCl_3$) δ 7.38 – 7.33 (m, 2H), 6.64 – 6.59 (m, 2H), 3.27 (t, *J* = 17.0 Hz, 2H); **^{13}C{1H} NMR** (75 MHz, $CDCl_3$) δ 147.5, 135.8, 120.9, 118.8 (m), 115.6, 113.1 (m), 38.0 (q, *J* = 23.7 Hz); **^{19}F NMR** (282 MHz, $CDCl_3$) δ -84.9 (s, 3F), -117.4 (t, *J* = 17.1 Hz, 2F); **IR** (cm^{-1}) (neat): 3478, 3380, 1622, 1597, 1496, 1183, 1017; **HRMS-ESI** (*m/z*) calcd for $C_9H_9F_5NS$ $[M + H]^+$: 258.0376. Found: 258.0377.

Thiobenzoic acid experiments

Scheme S12 – Trifluoroethylation of thiobenzoic acid by one-pot sequential protocol.

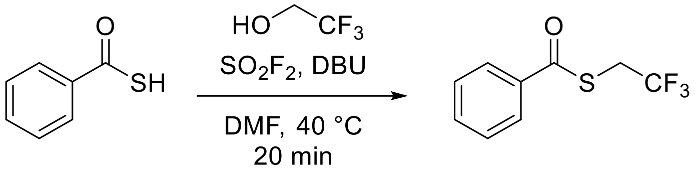

Thiobenzoic acid was subjected to the general procedure for one-pot trifluoroethylation of thiols on a 1 mmol scale. The reaction was monitored by quantitative ^{19}F NMR, using $PhCF_3$ (100 µL, 0.815 mmol) as an internal standard. The ^{19}F NMR showed that sulfur was alkylated in high yields. The reaction mixture was worked up by diluting in water (15 mL), and then extracted with diethyl ether (5 x 5 mL). The combined organics were washed with water (5 x 5 mL) and brine (2 x 5 mL), and then dried over anhydrous Na_2SO_4, decanted and concentrated *in vacuo*. Fractional distillation has been attempted to purify the residue, however, it was unsuccessful in obtaining the desired product. It is presumed that the trifluoroethyl thioester hydrolyzed or underwent a trans-esterification type reaction with the excess trifluoroethanol and DBU in the reaction mixture.

Figure S2 – ^{19}F NMR spectrum of the thiobenzoic acid trifluoroethylation reaction.

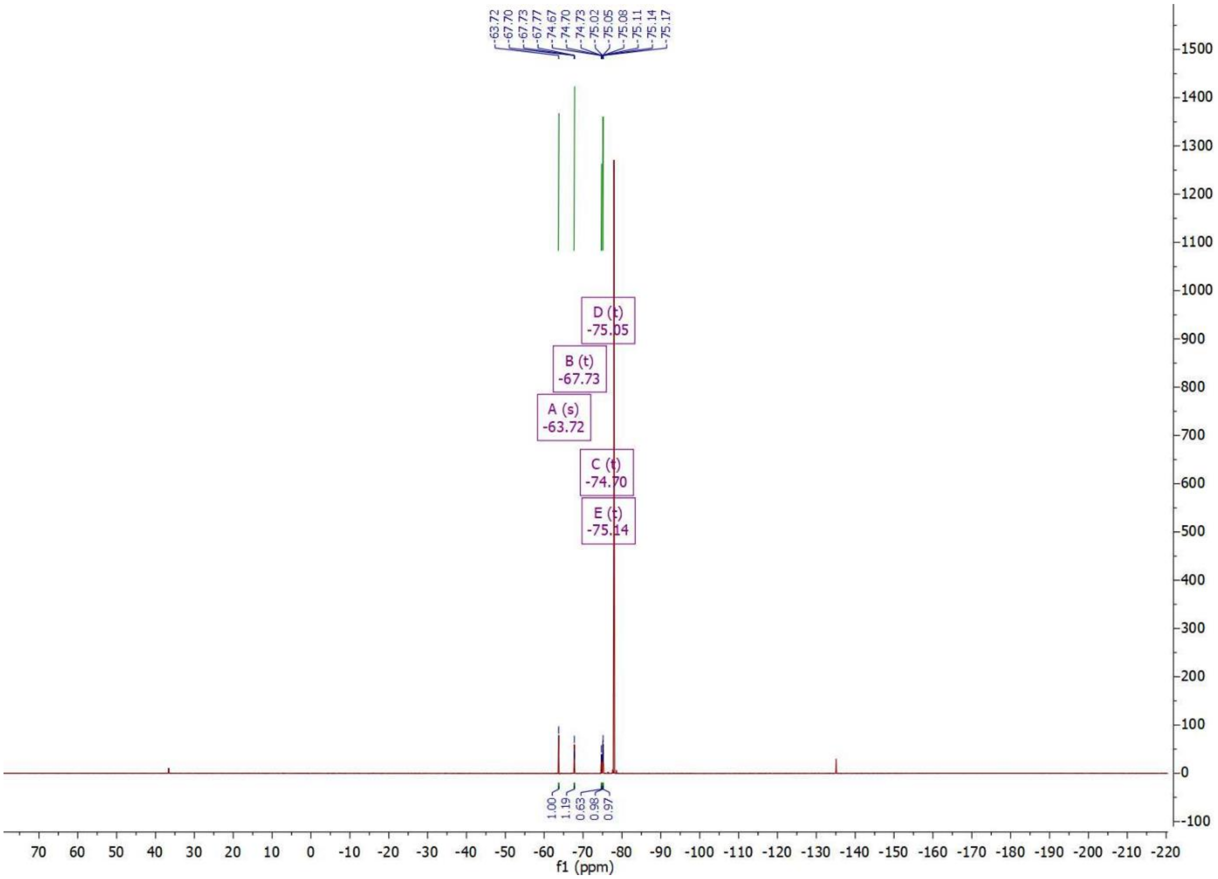

References

1) S. A. Kinkead, R. C. Kumar, J. M. Shreeve, *J. Am. Chem. Soc.* 1984, **106**, 7496–7500.
2) S. Hyde, J. Veliks, B. Liegault, D. Grassi, M. Taillefer, V. Gouverneur, *Angew. Chem. Int. Ed.* 2016, **55**, 3784-3789.
3) A. Toutchkine, D. Aebisher, E. L. Clennan, *J. Am. Chem. Soc.* 2001, **123**, 4966-4973.
4) S. Chen, M. Zhang, X. Liao, Z. Weng, *J. Org. Chem.* 2016, **81**, 7993-8000.

6a (^{1}H NMR in CD_2Cl_2) (300 MHz)

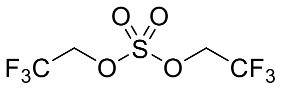

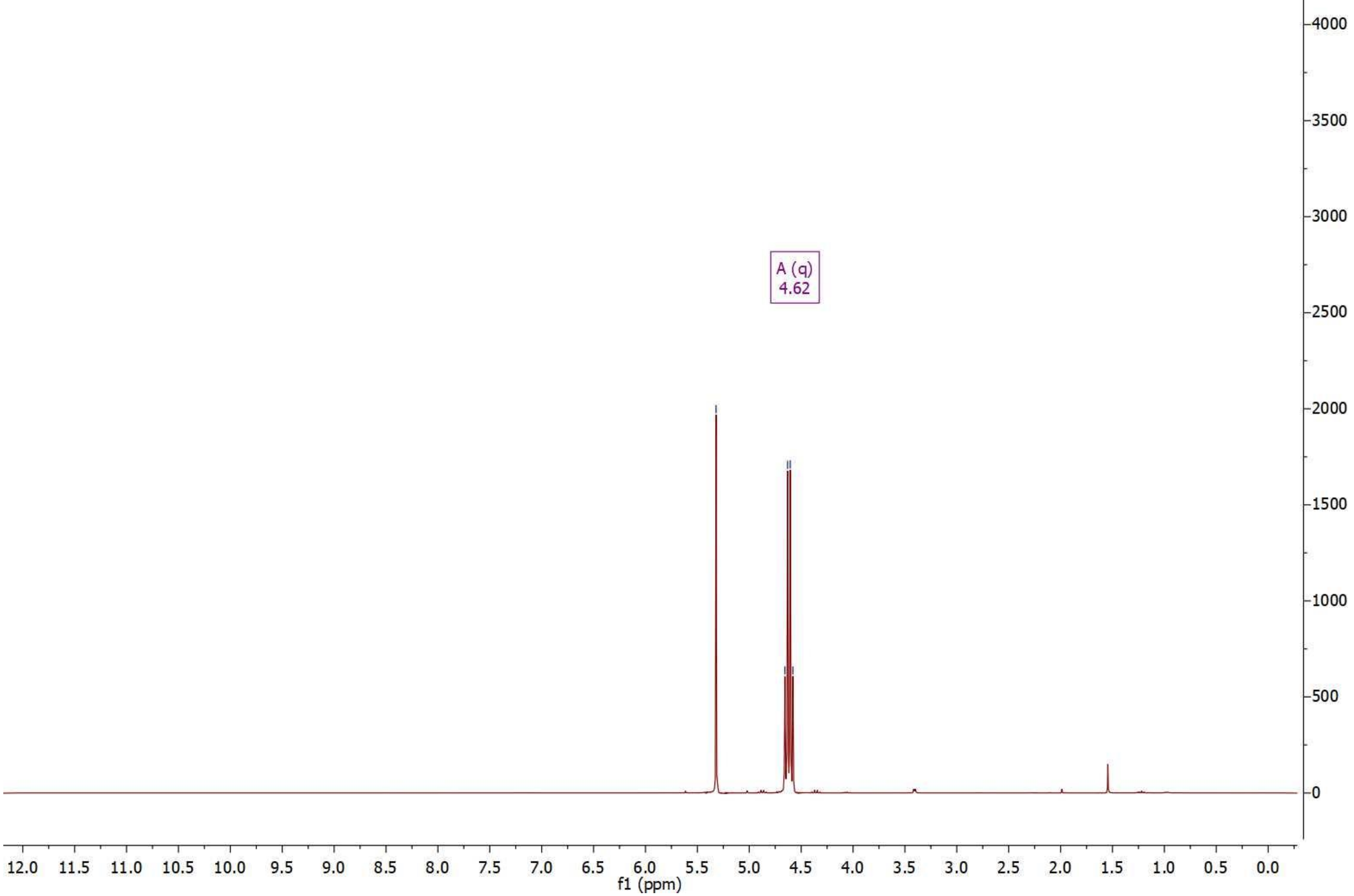

6a (^{13}C NMR in CD_2Cl_2) (75 MHz)

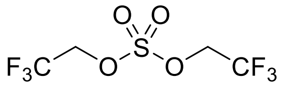

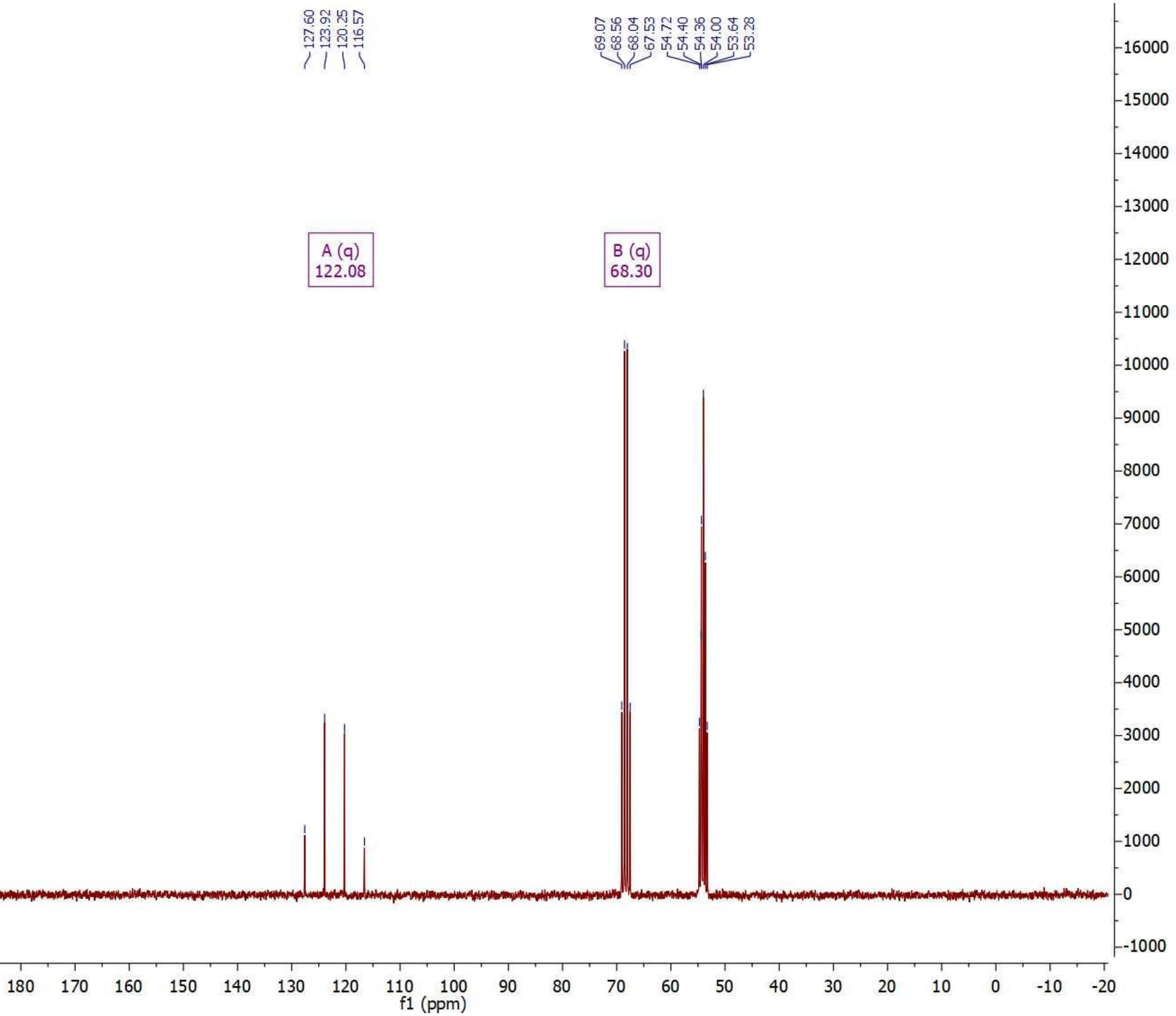

6a (^{19}F NMR in CD_2Cl_2) (282 MHz)

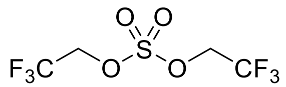

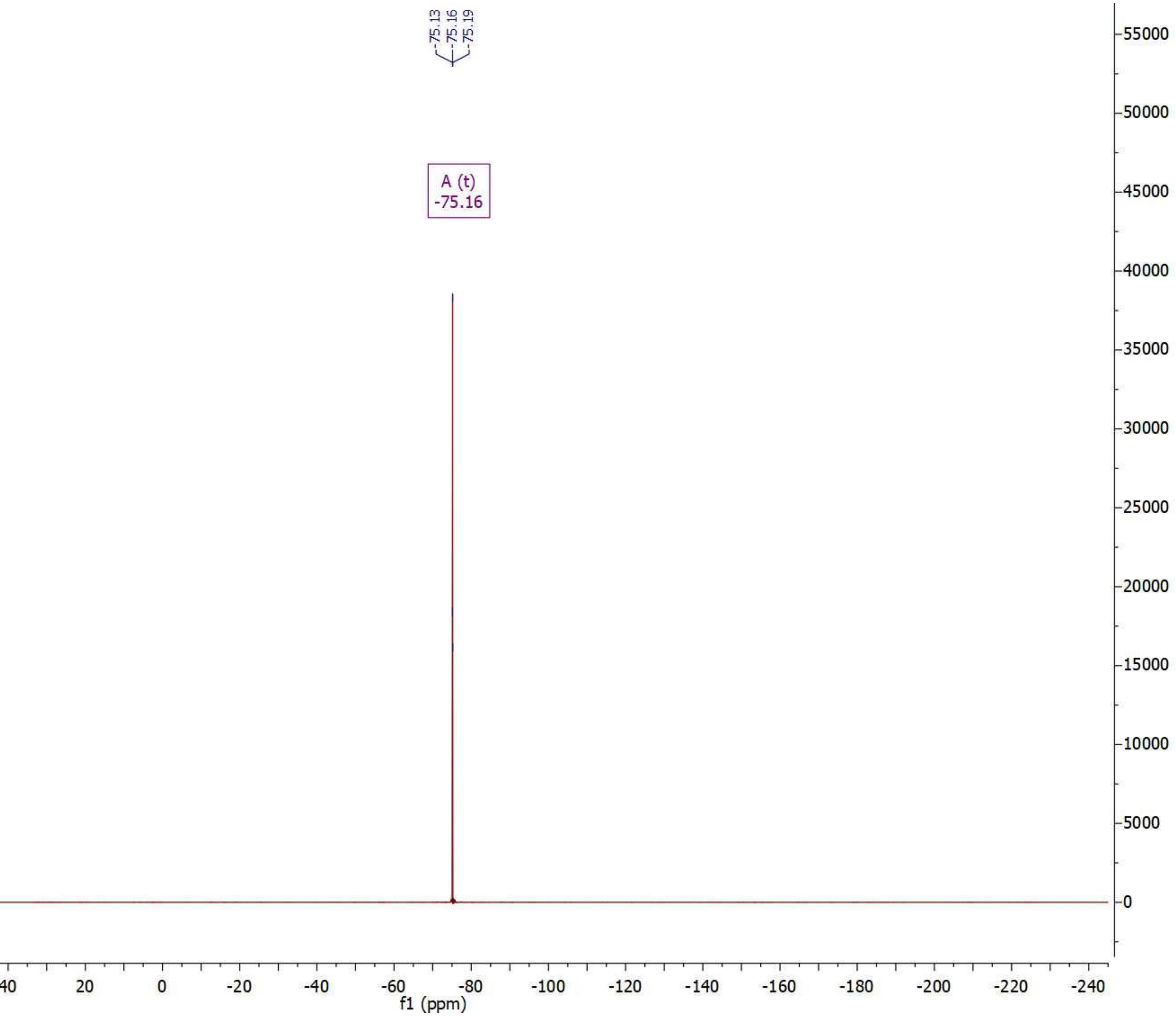

6b (^{1}H NMR in CD_2Cl_2) (300 MHz)

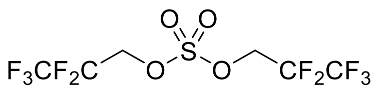

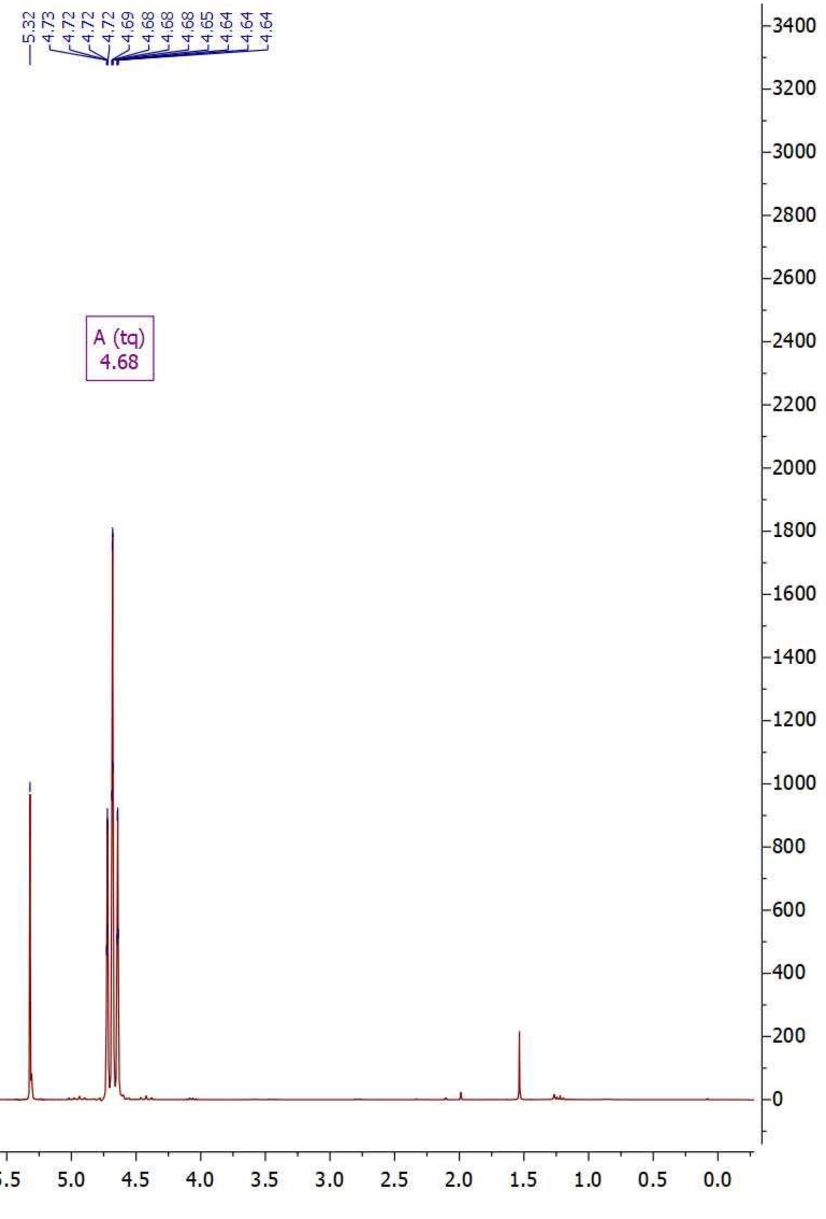

6b (^{13}C NMR in CD_2Cl_2) (75 MHz)

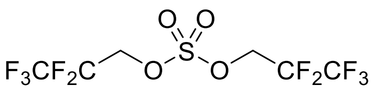

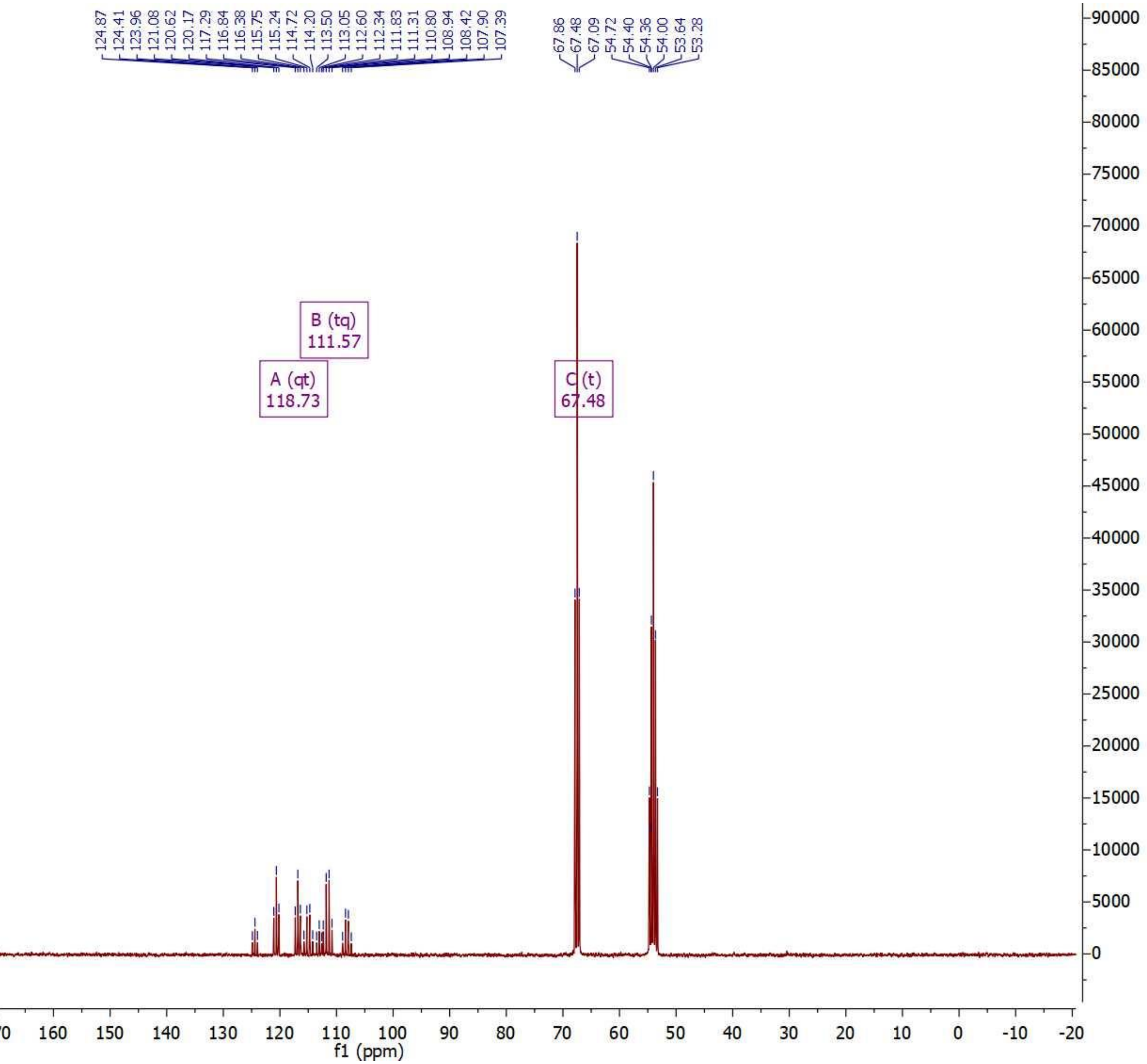

6b (^{19}F NMR in CD_2Cl_2) (282 MHz)

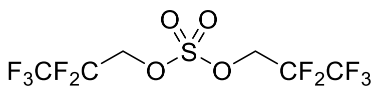

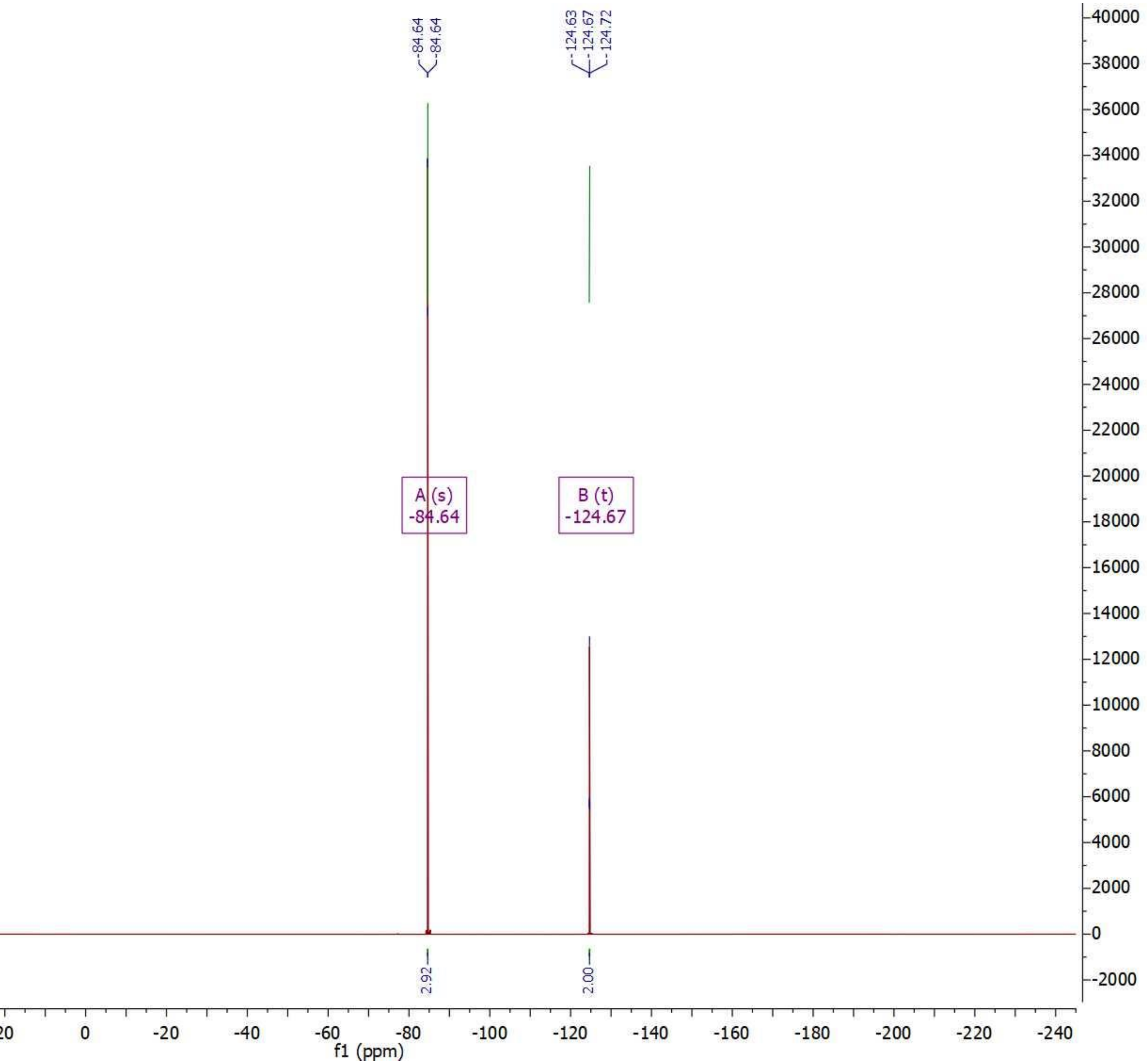

10a (^{1}H NMR in $CDCl_3$) (300 MHz)

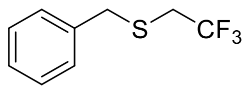

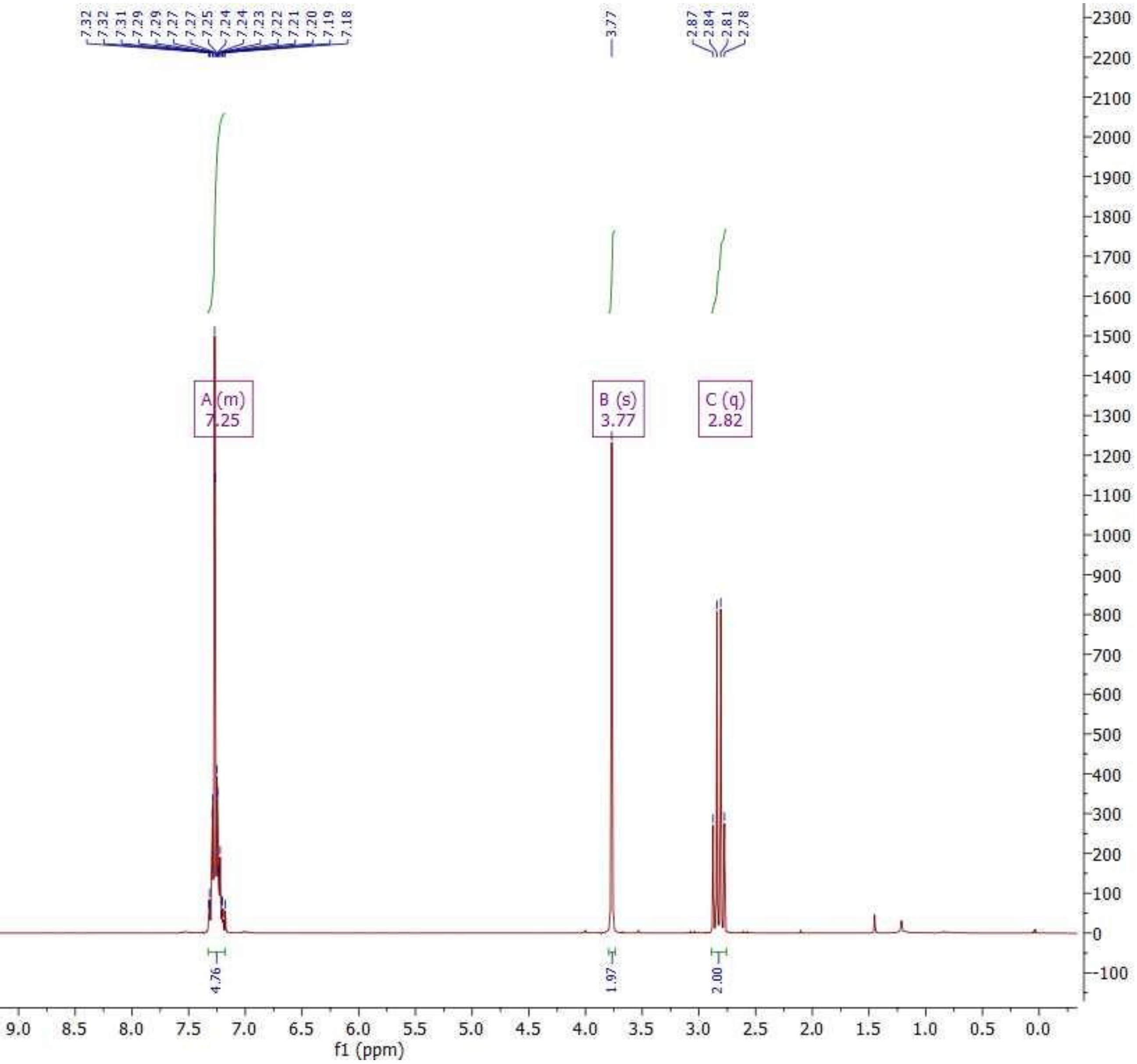

10a (^{13}C NMR in $CDCl_3$) (75 MHz)

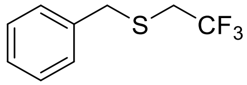

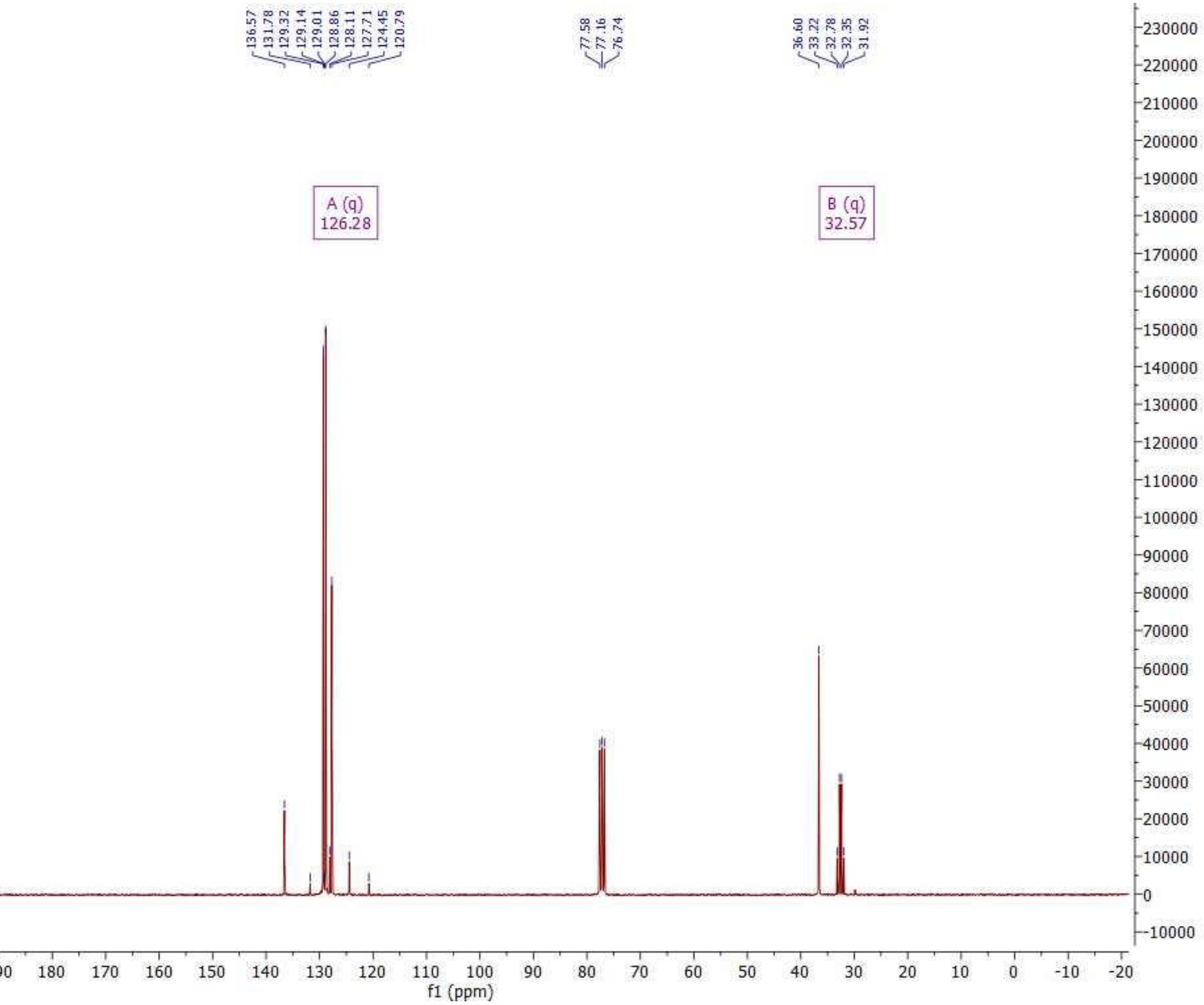

10a (^{19}F NMR in $CDCl_3$) (282 MHz)

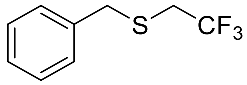

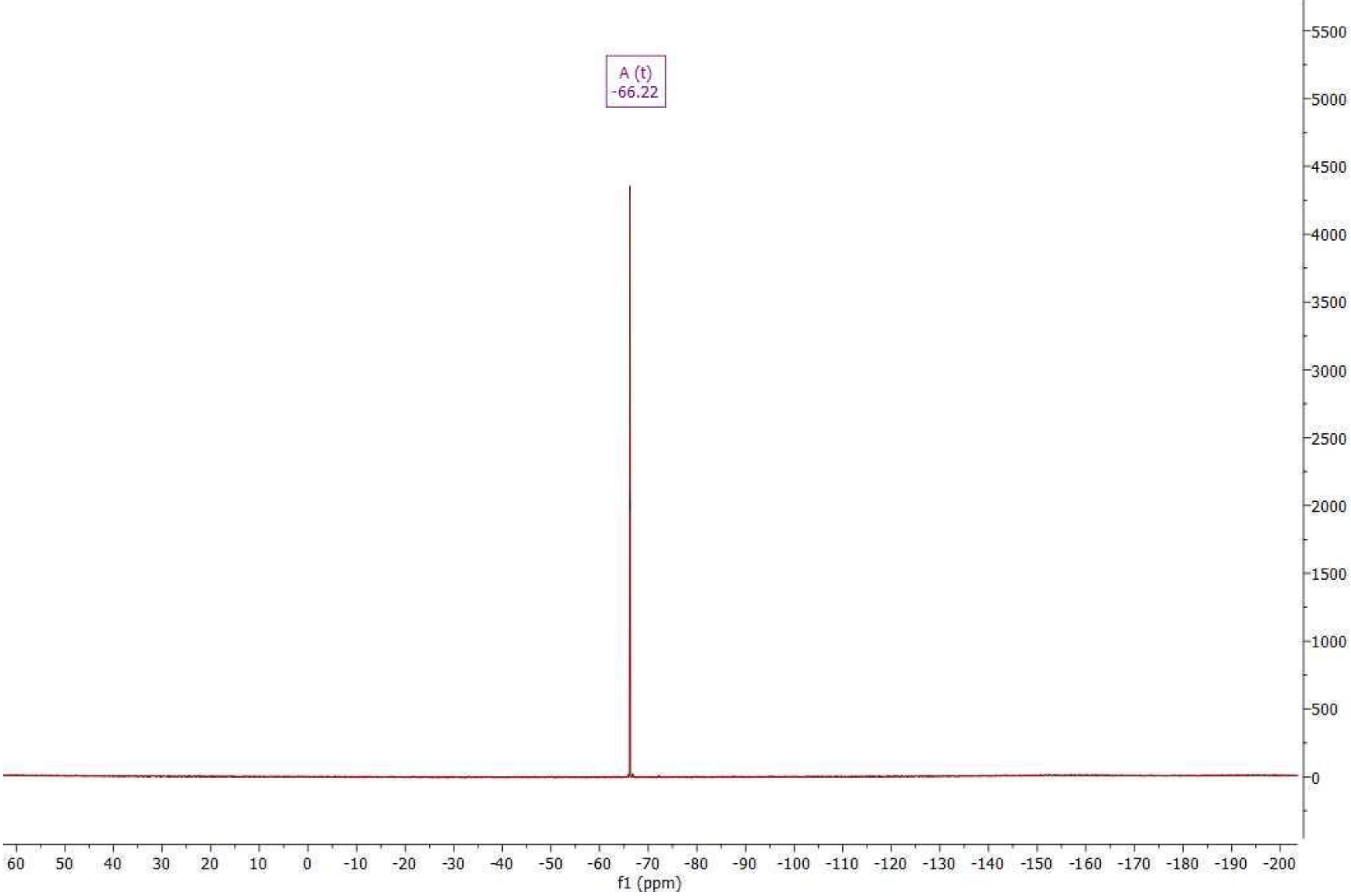

10b (^{1}H NMR in $CDCl_3$) (300 MHz)

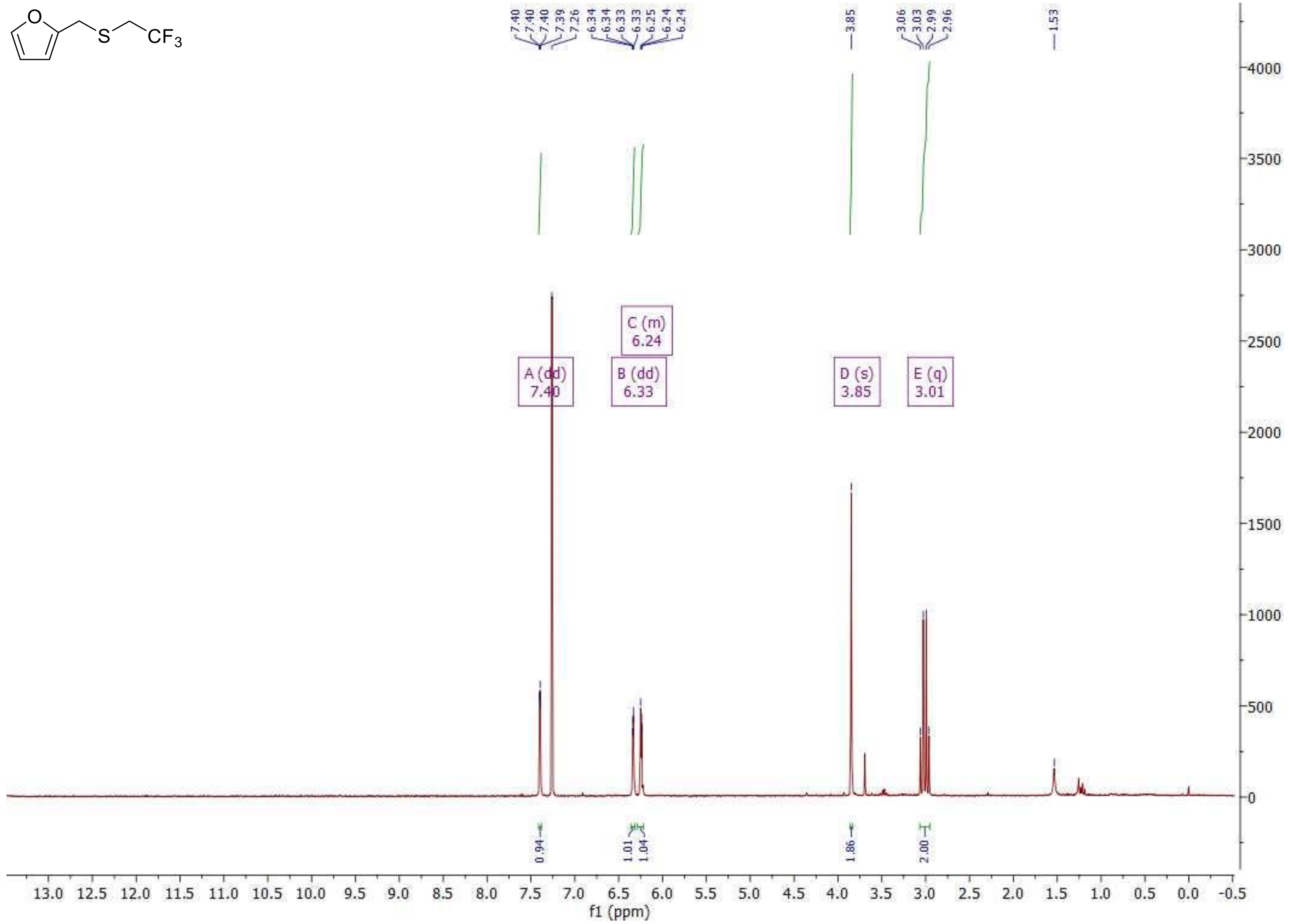

10b (^{13}C NMR in $CDCl_3$) (75 MHz)

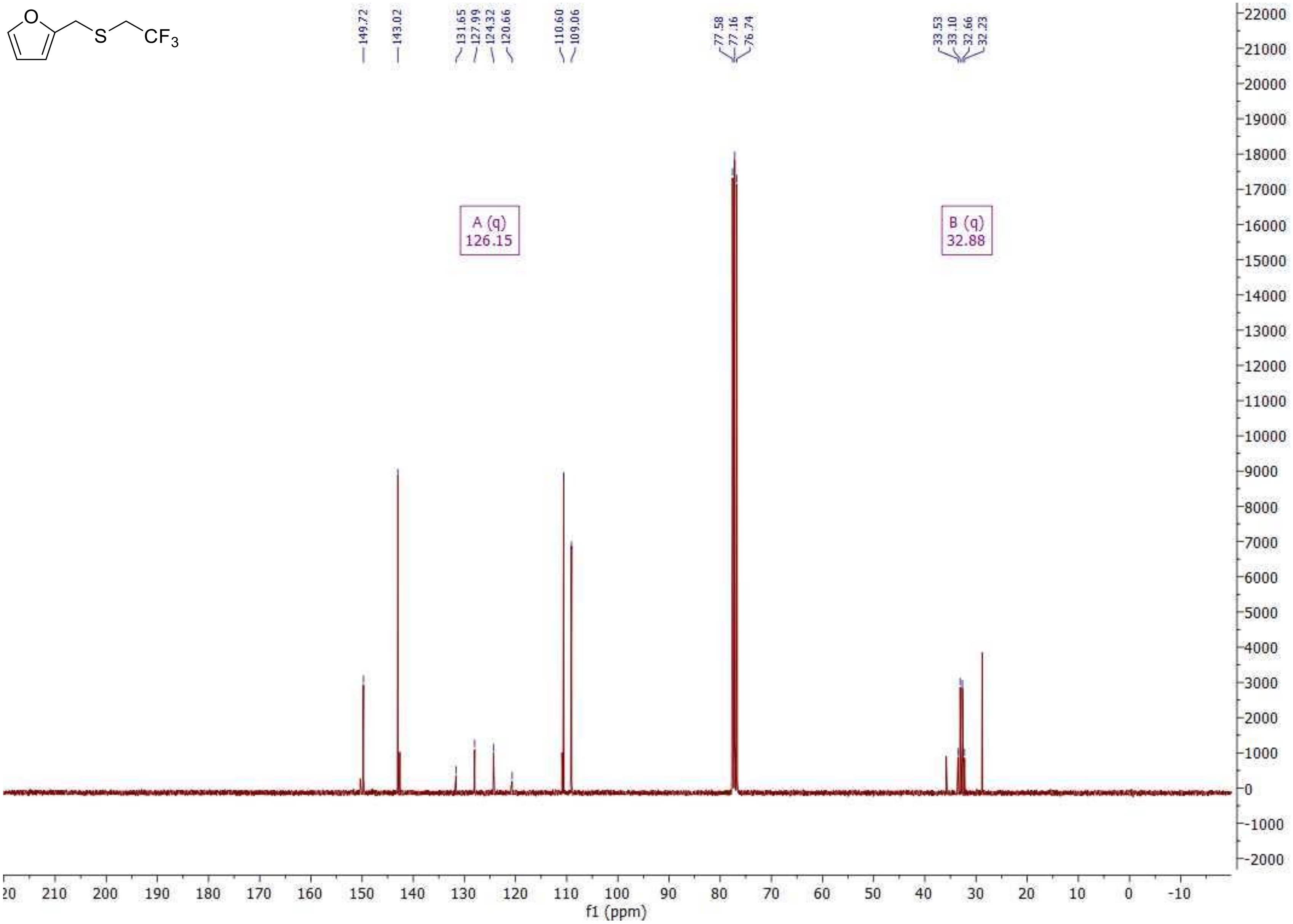

10b (^{19}F NMR in $CDCl_3$) (282 MHz)

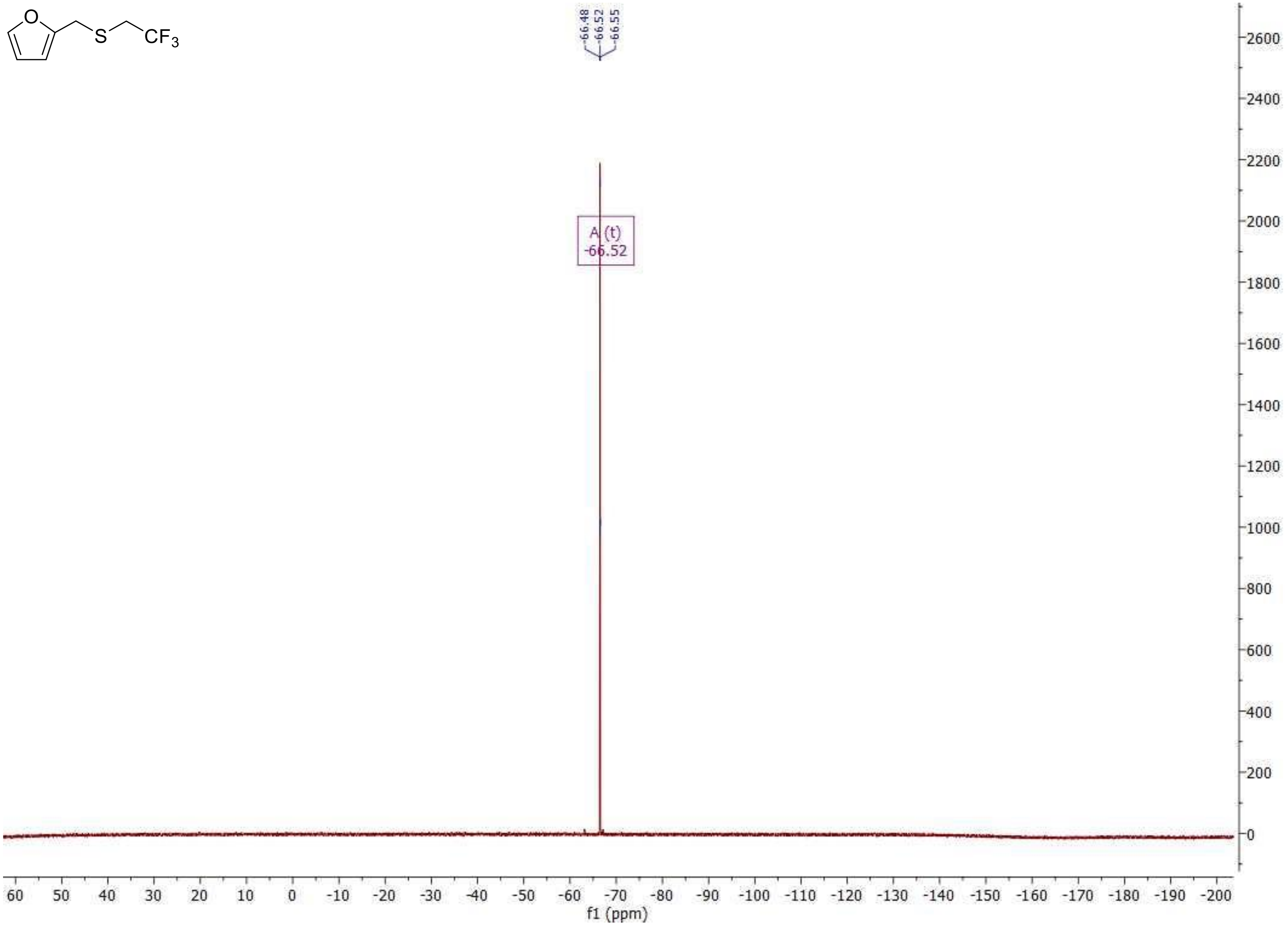

10c (^{1}H NMR in $CDCl_3$) (300 MHz)

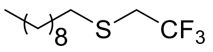

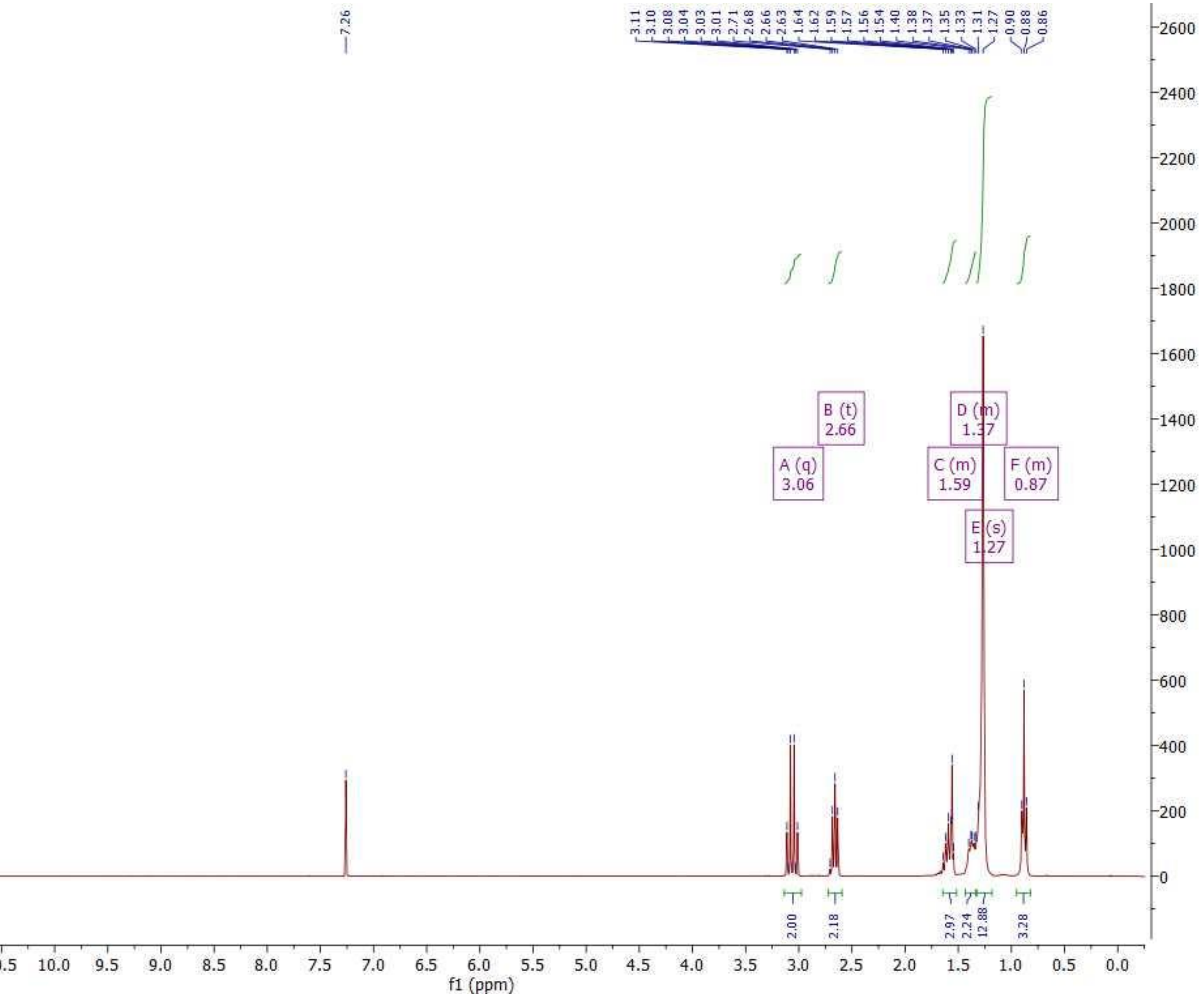

10c (^{13}C NMR in $CDCl_3$) (75 MHz)

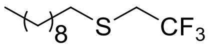

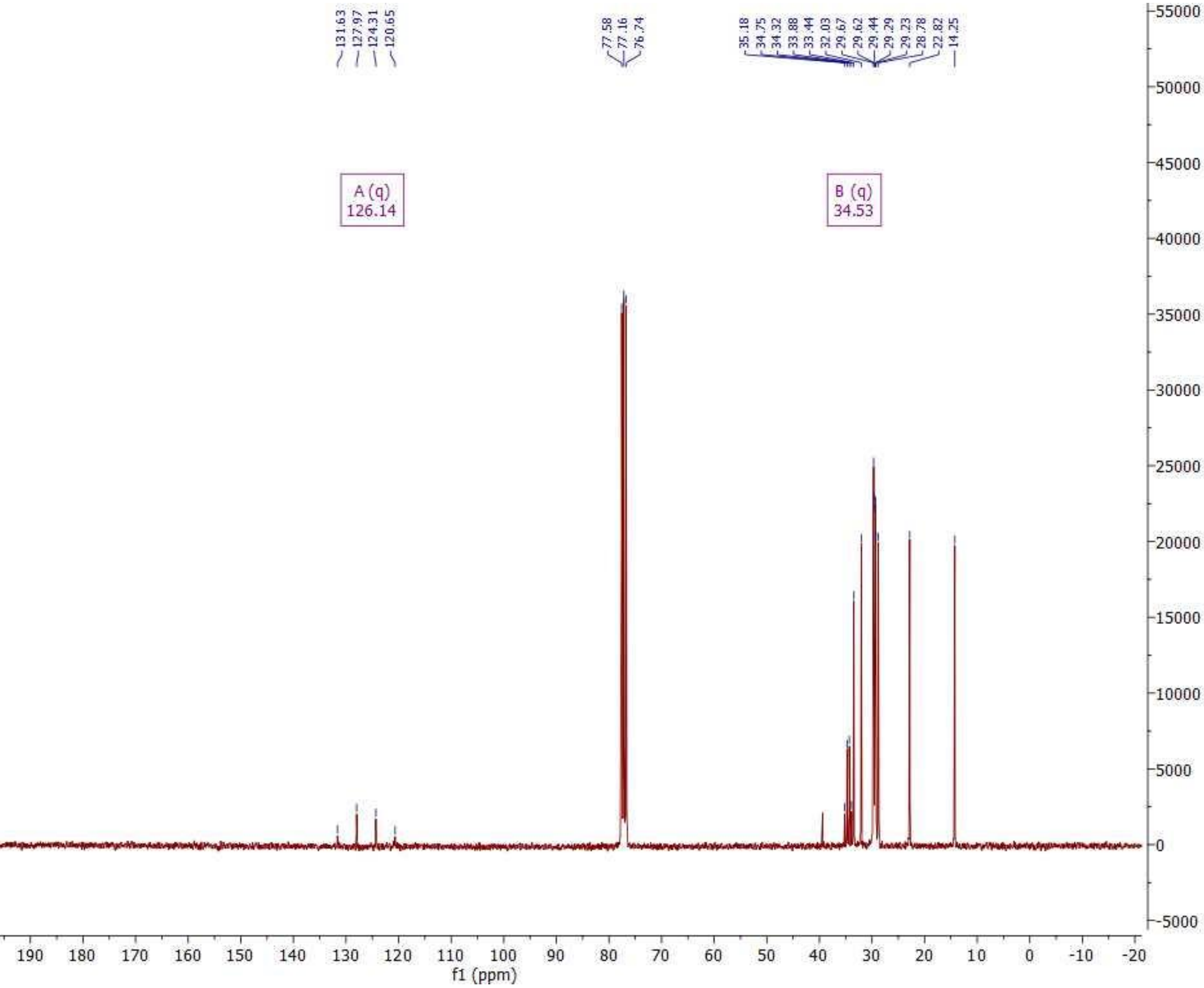

10c (^{19}F NMR in $CDCl_3$) (282 MHz)

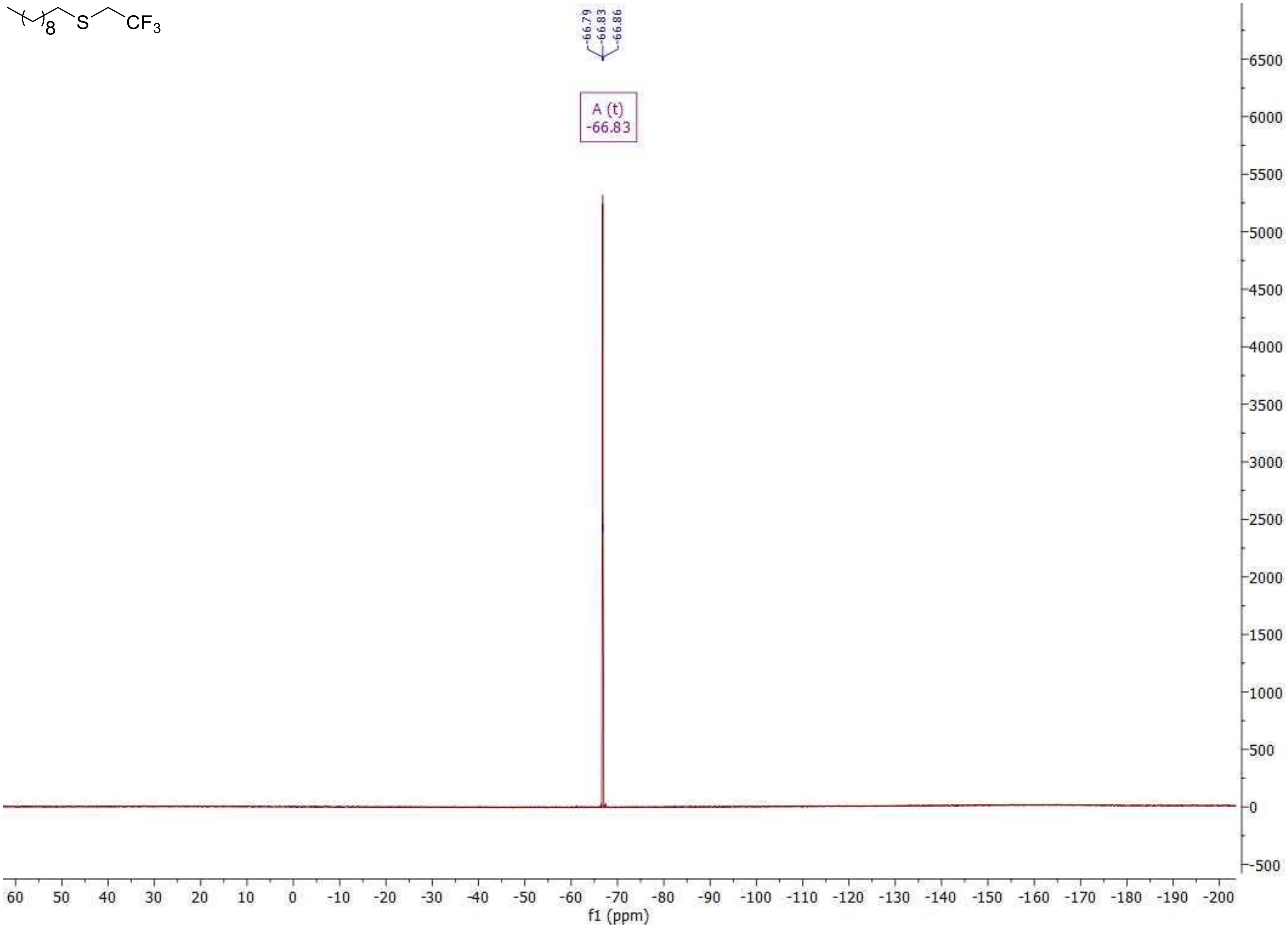

10d (^{1}H NMR in $CDCl_3$) (300 MHz)

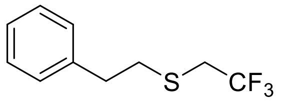

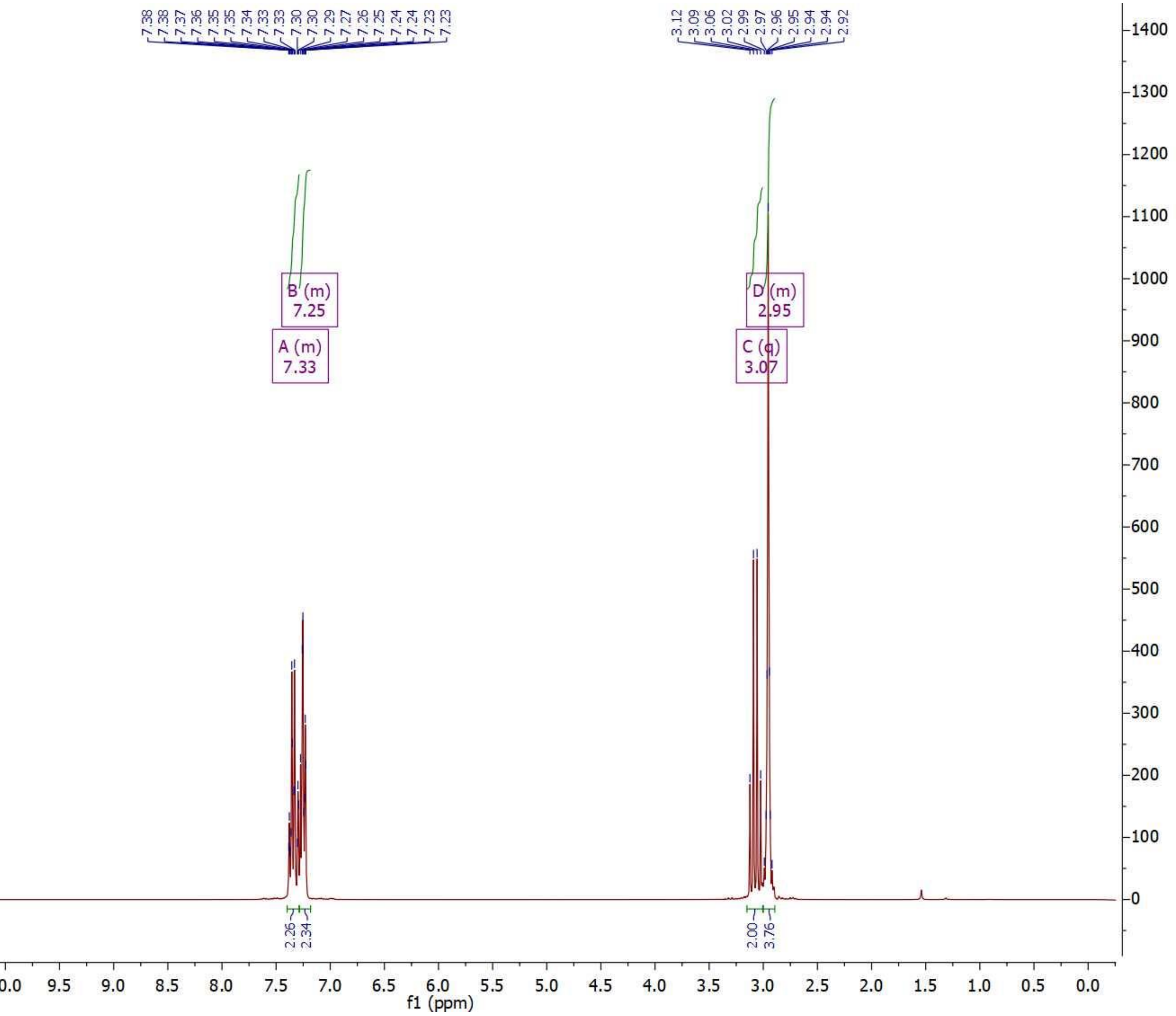

10d (^{13}C NMR in $CDCl_3$) (75 MHz)

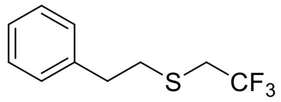

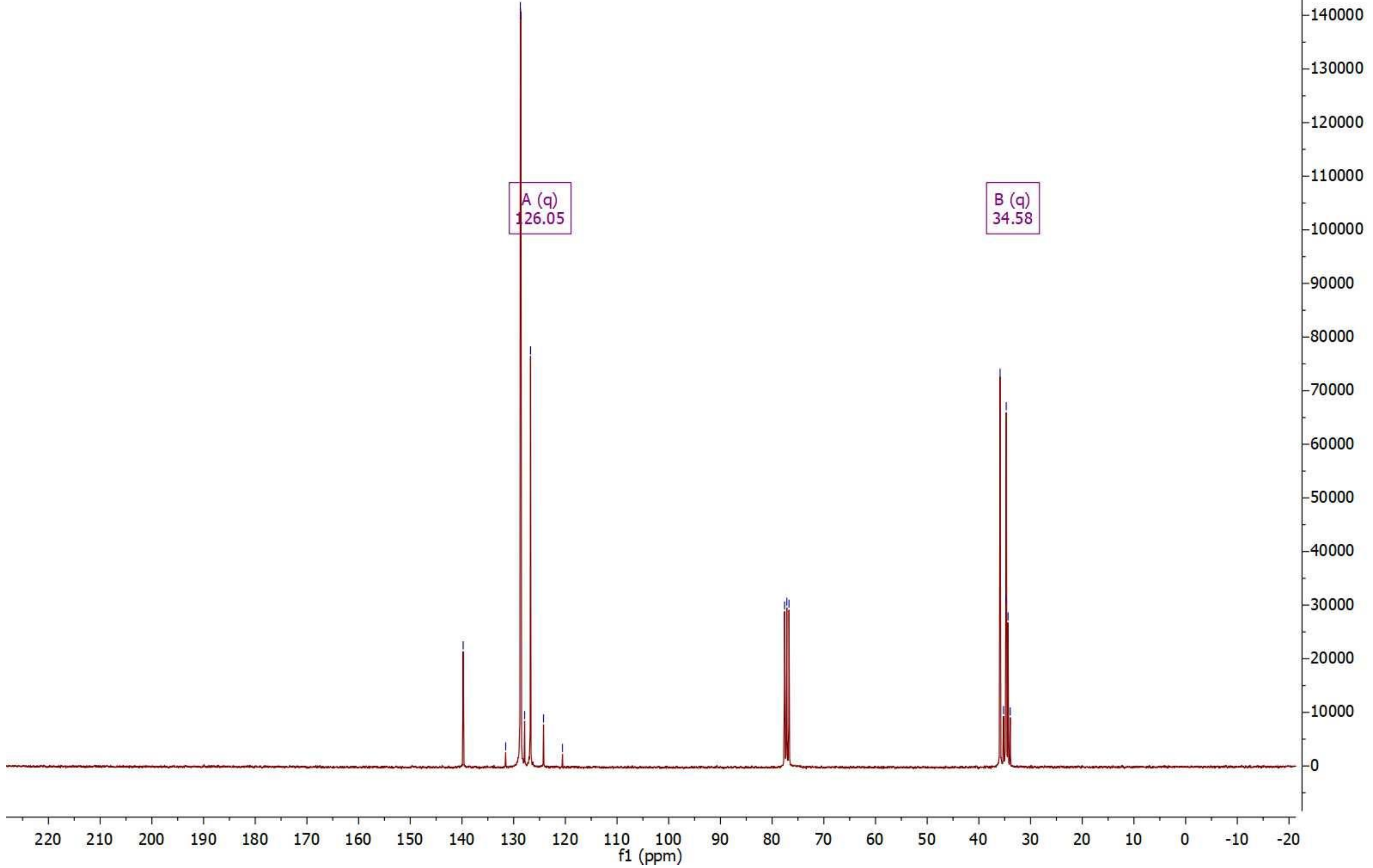

10d (^{19}F NMR in $CDCl_3$) (282 MHz)

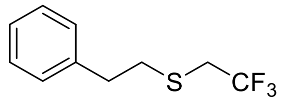

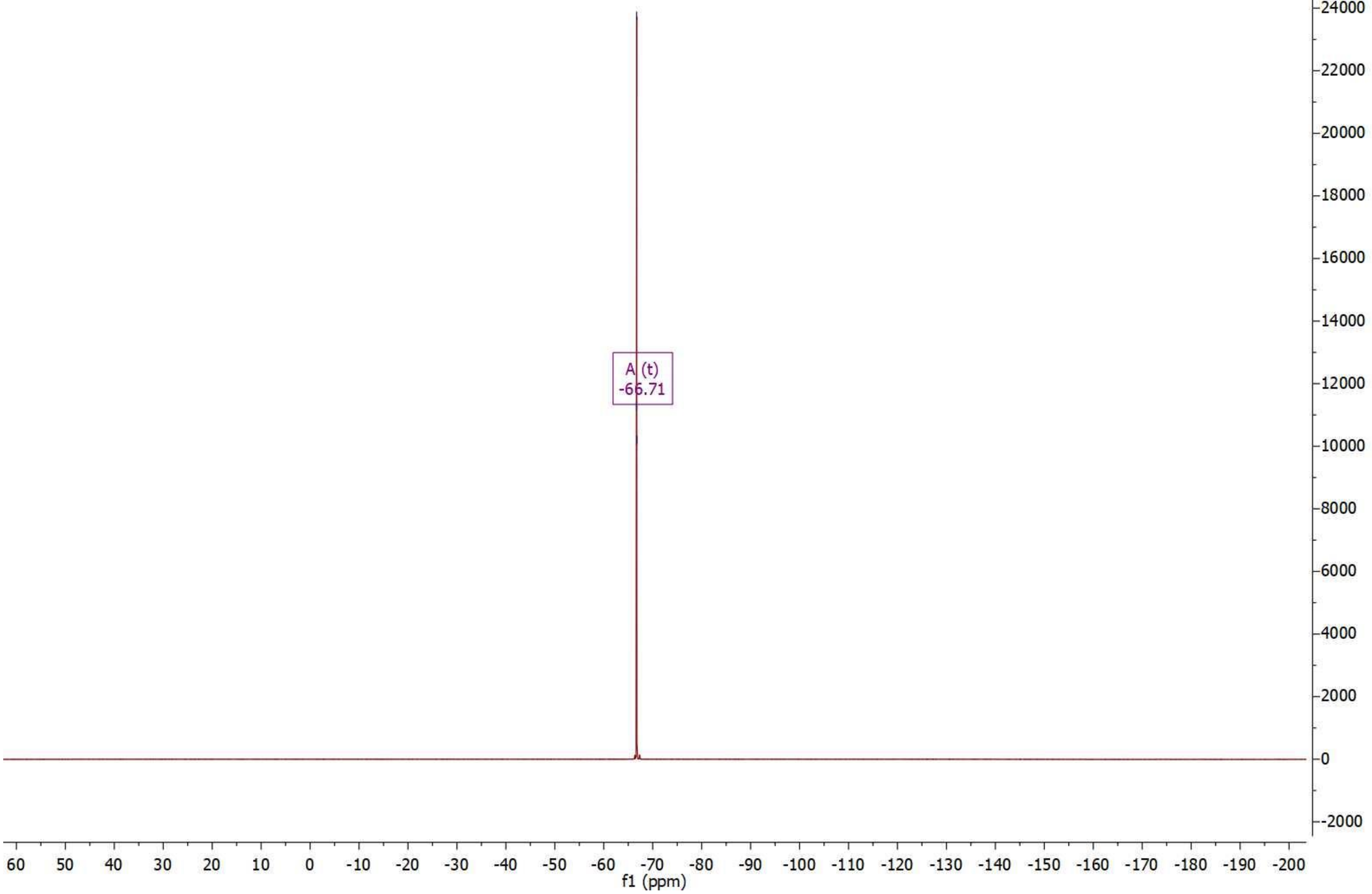

10e (^{1}H NMR in $CDCl_3$) (300 MHz)

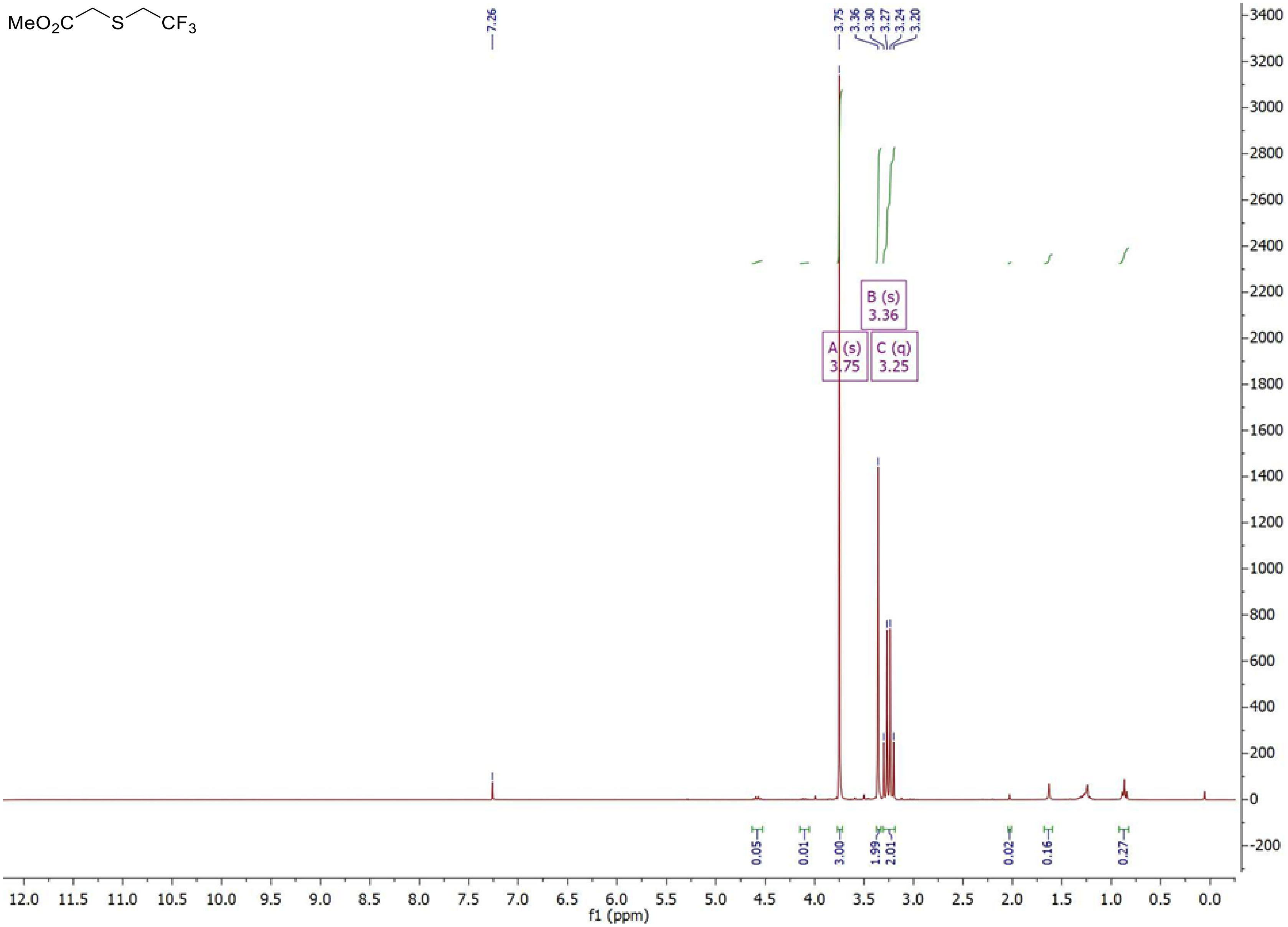

10e (^{13}C NMR in $CDCl_3$) (101 MHz)

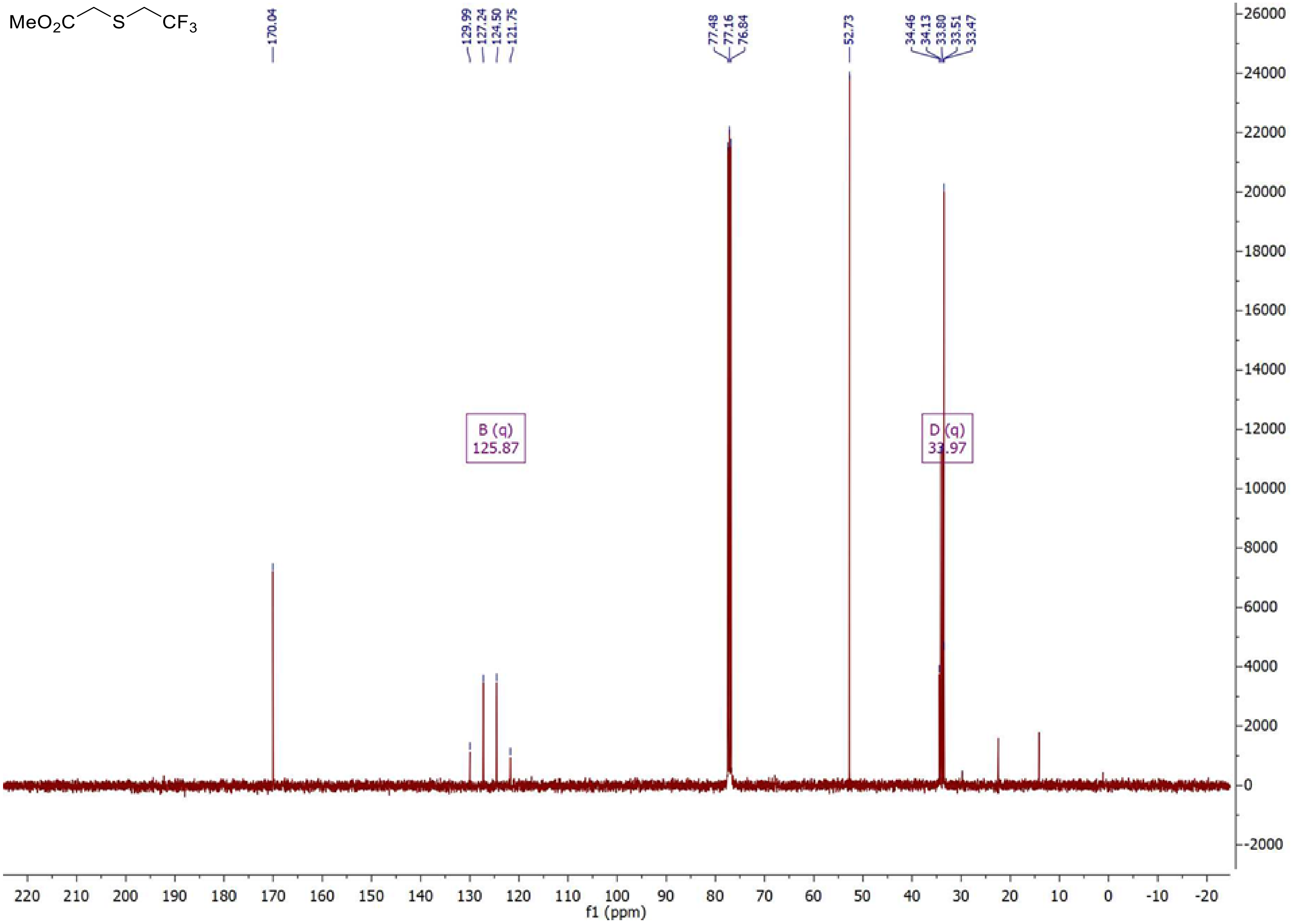

10e (^{19}F NMR in $CDCl_3$) (282 MHz)

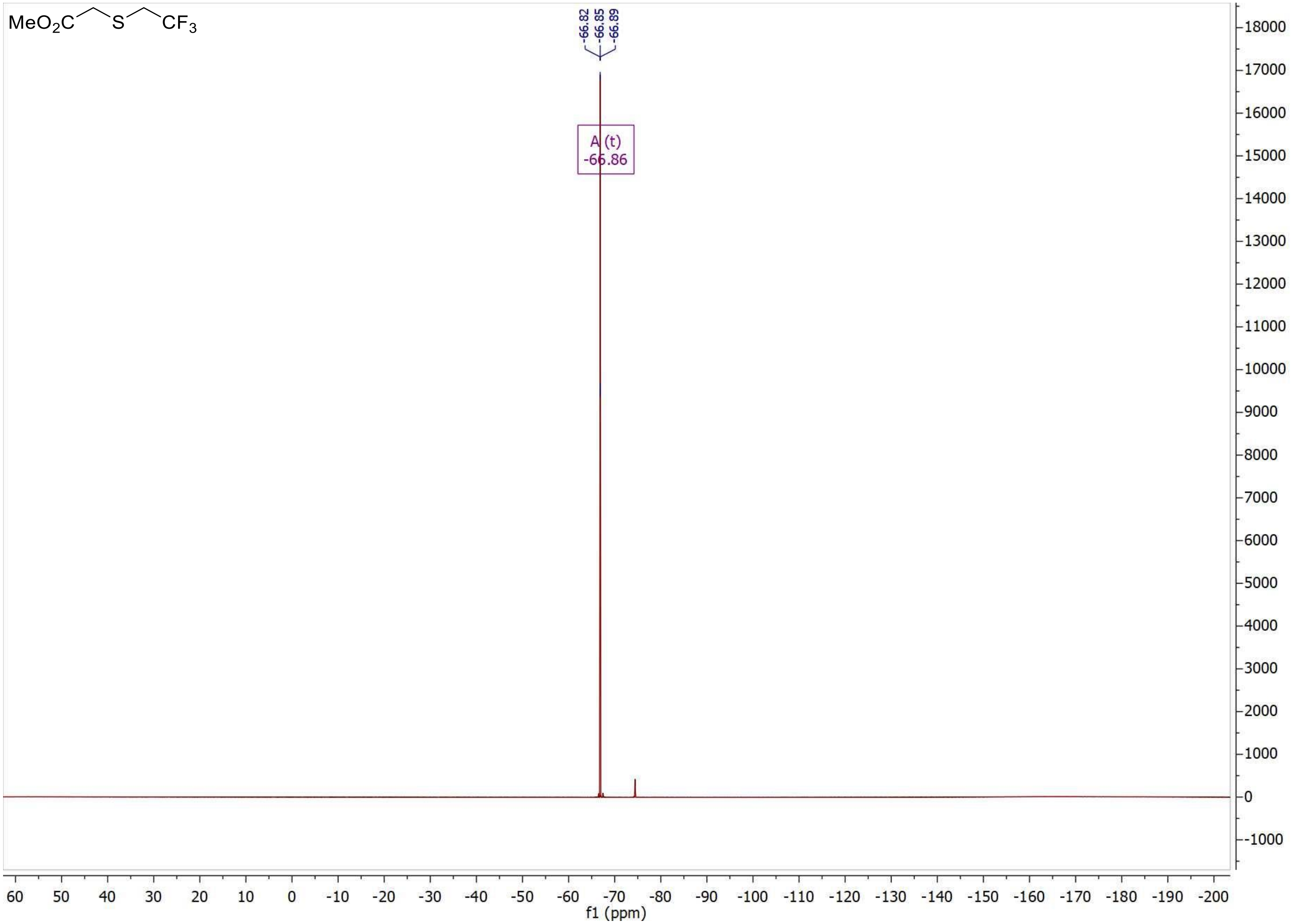

10f (^{1}H NMR in $CDCl_3$) (300 MHz)

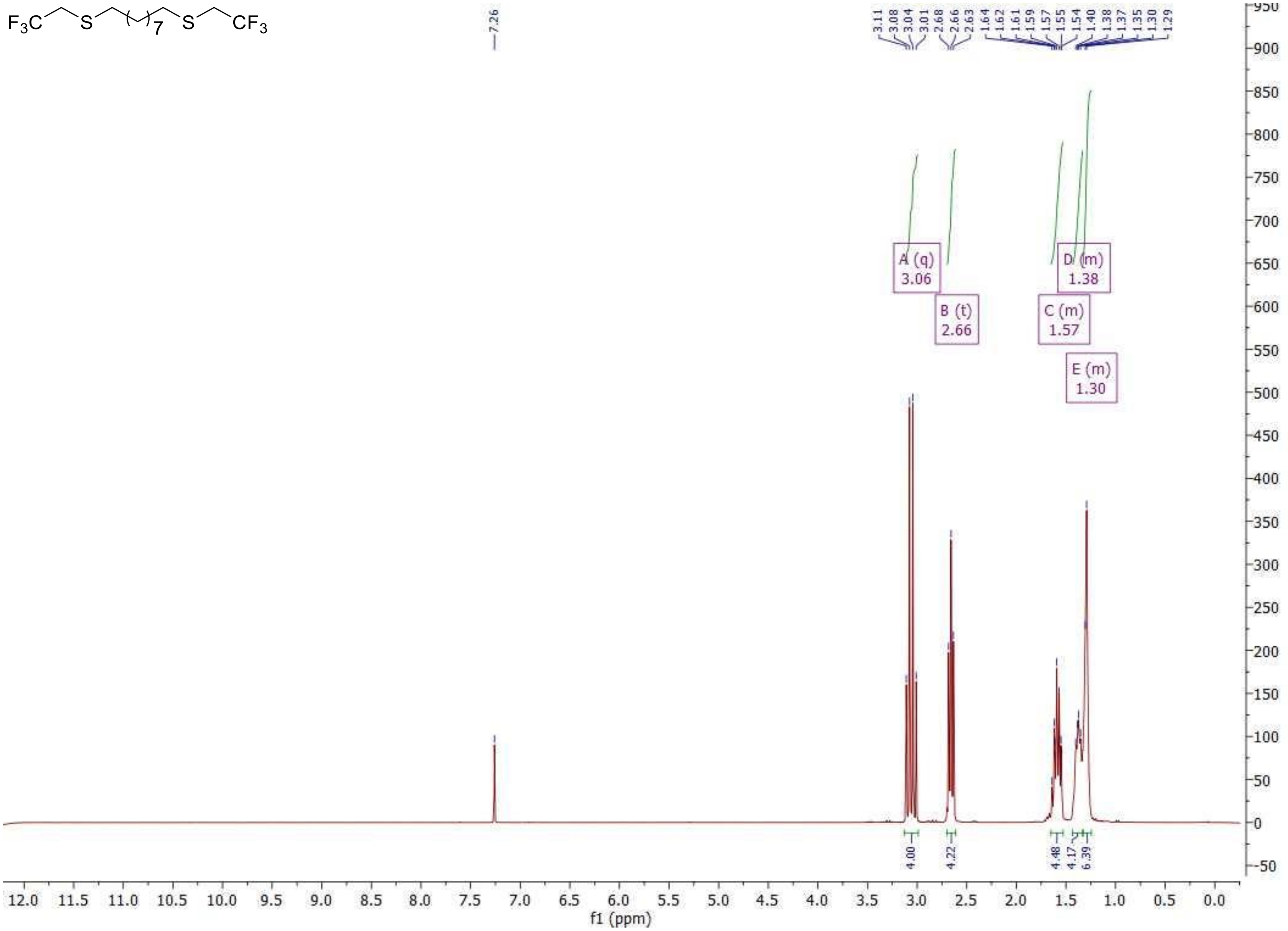

10f (^{13}C NMR in $CDCl_3$) (75 MHz)

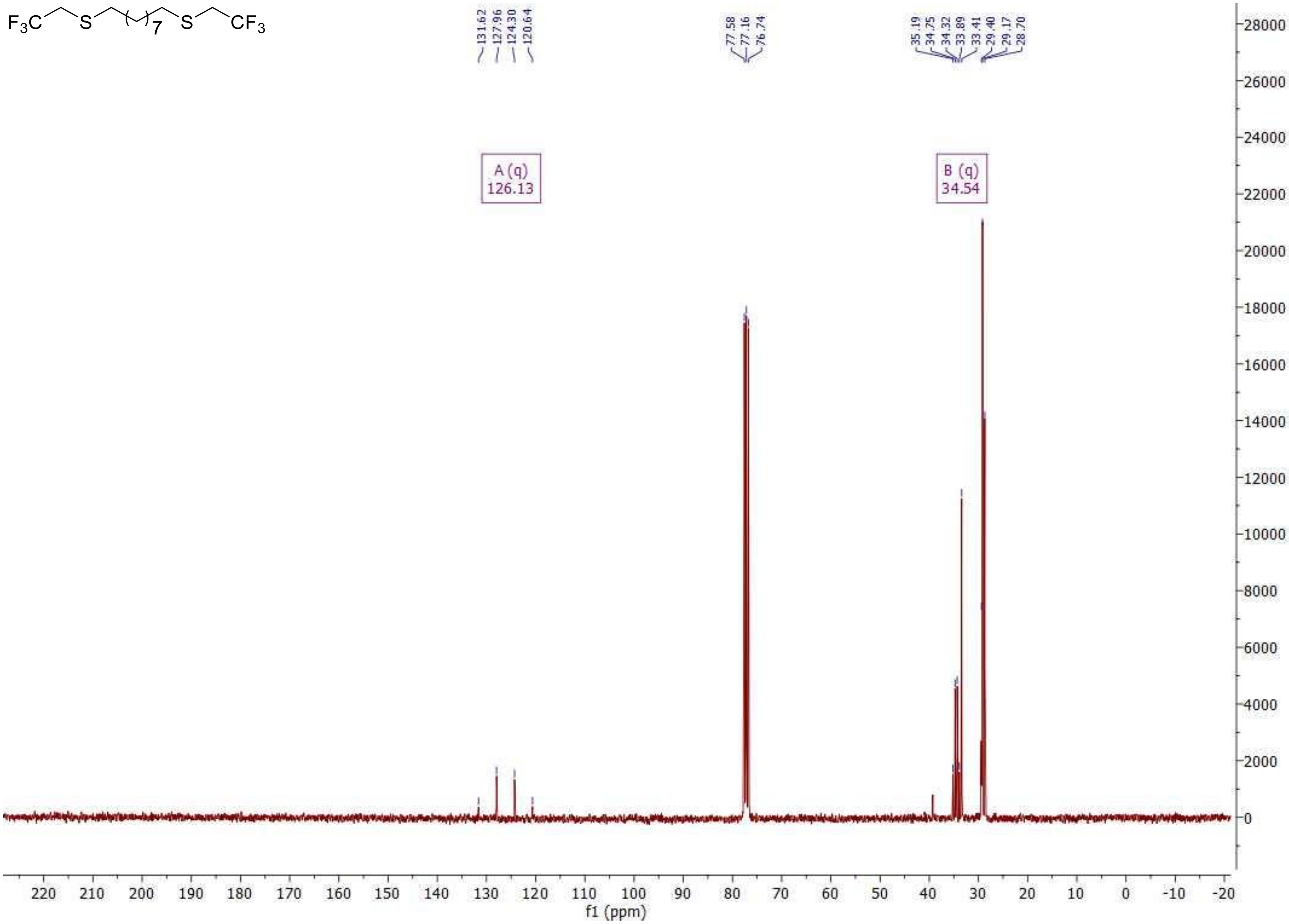

10f (^{19}F NMR in $CDCl_3$) (282 MHz)

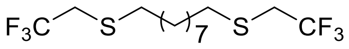

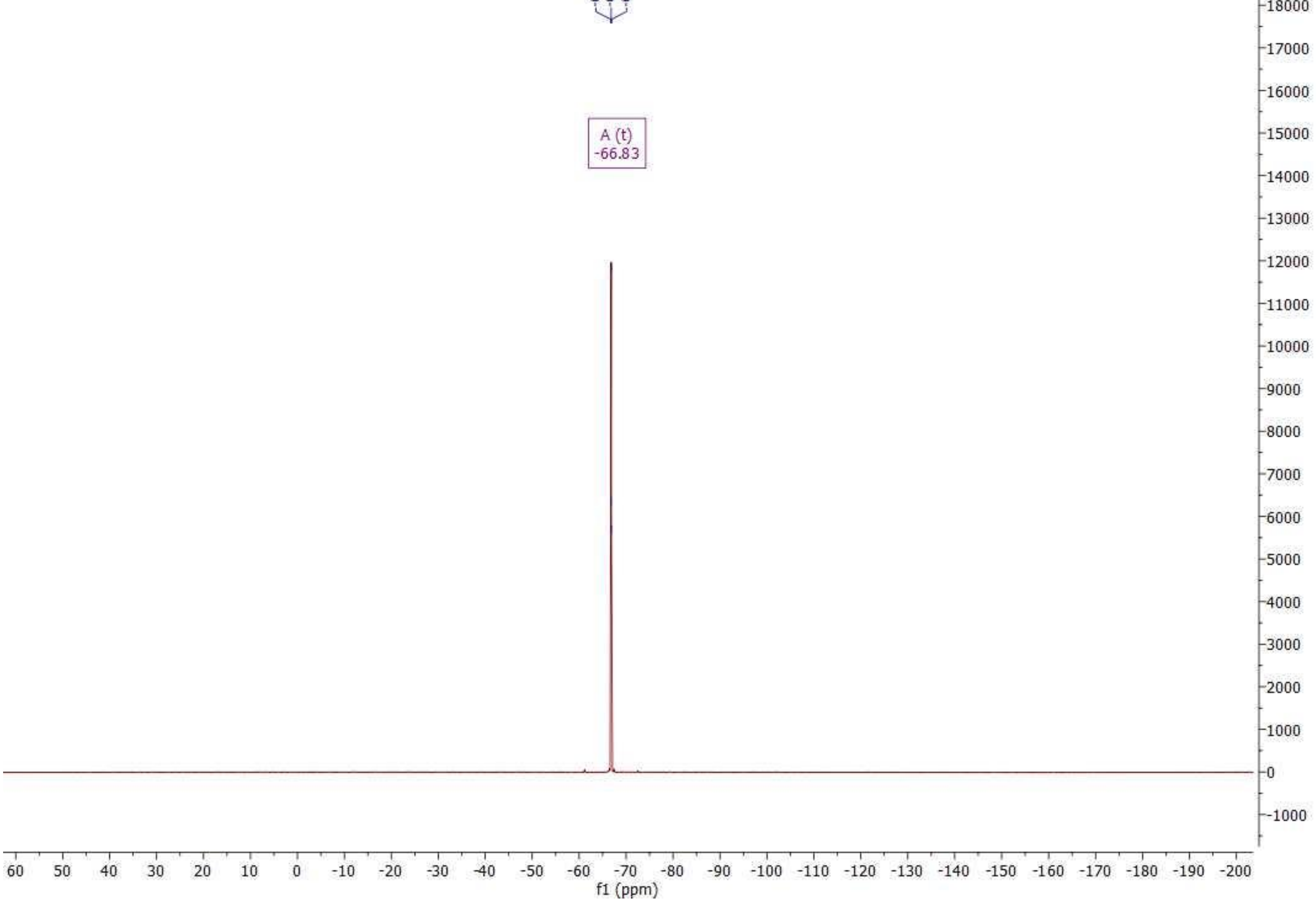

10h (^{1}H NMR in $CDCl_3$) (300 MHz)

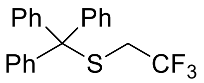

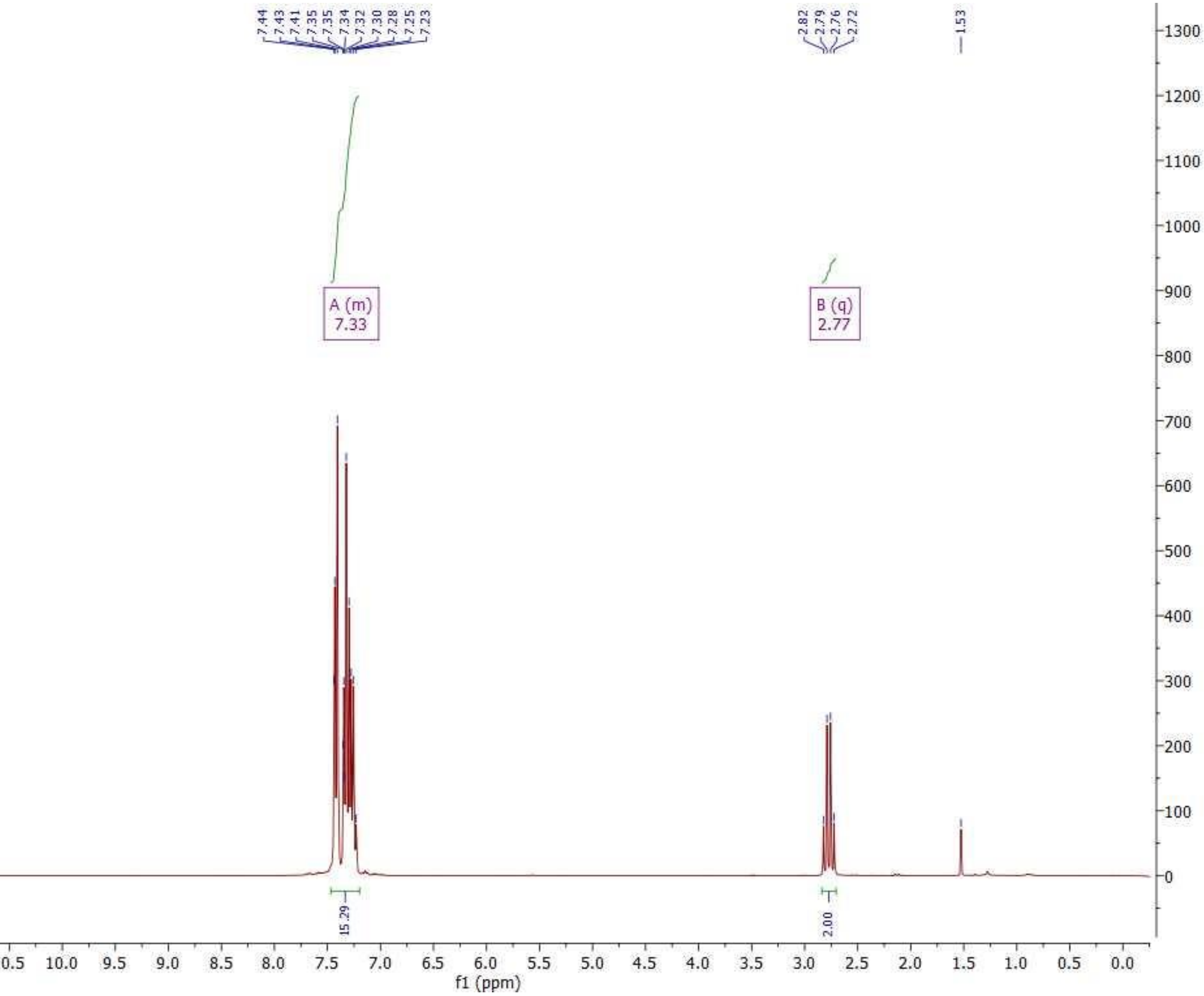

10h (^{13}C NMR in $CDCl_3$) (75 MHz)

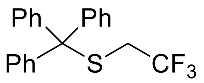

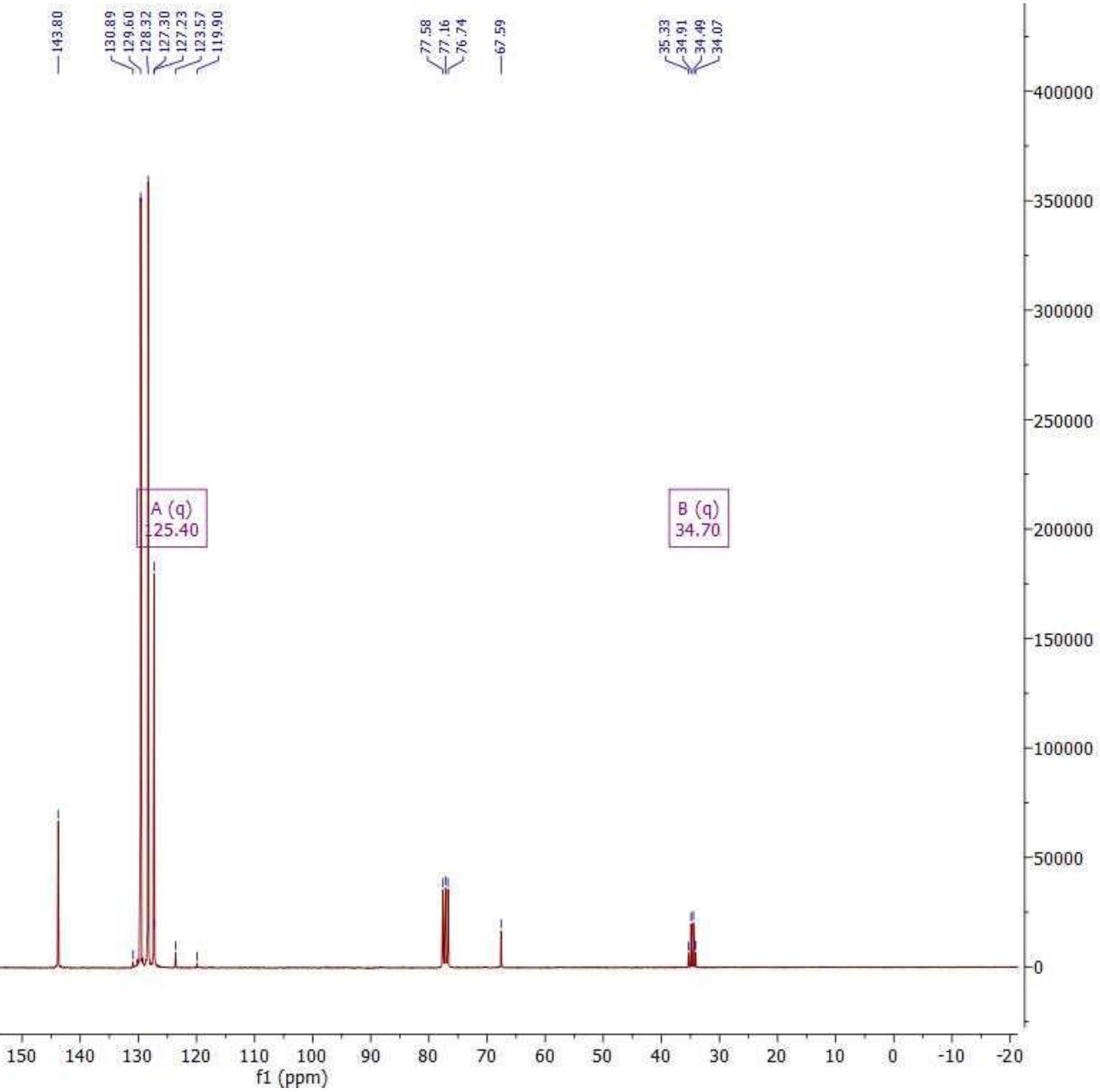

10h (^{19}F NMR in $CDCl_3$) (282 MHz)

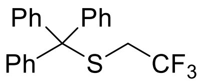

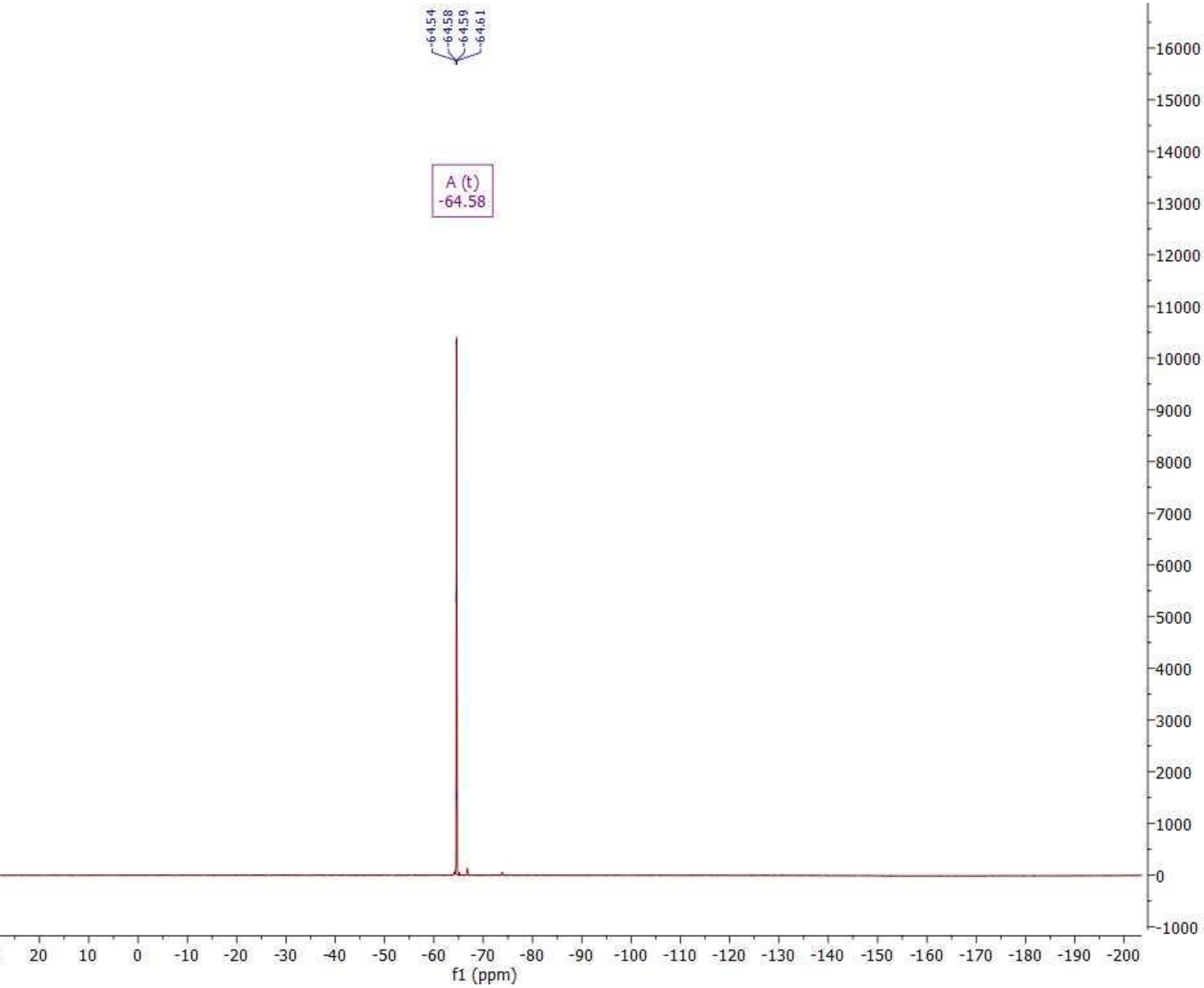

10i (^{1}H NMR in $CDCl_3$) (300 MHz)

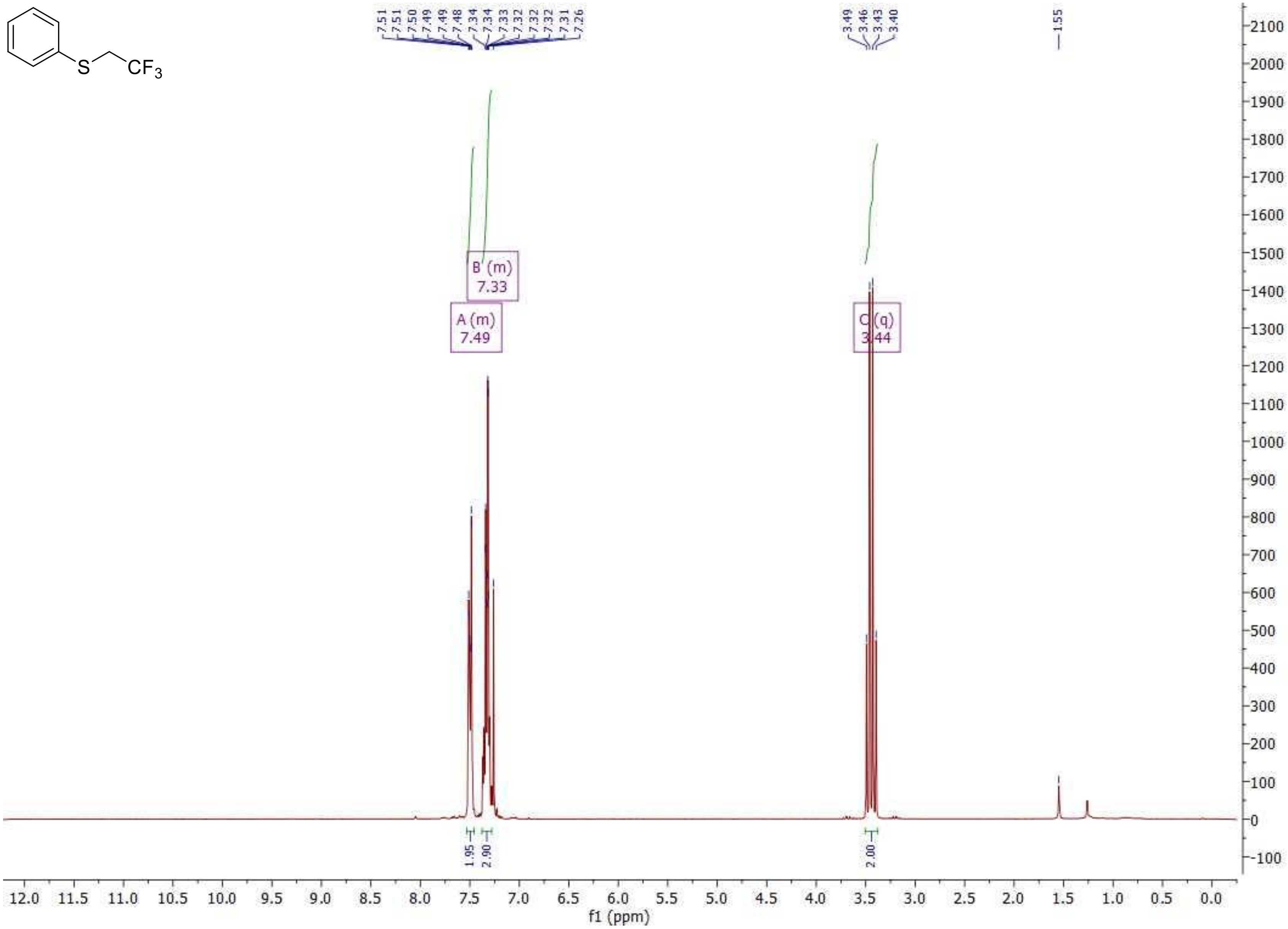

10i (^{13}C NMR in $CDCl_3$) (75 MHz)

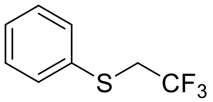

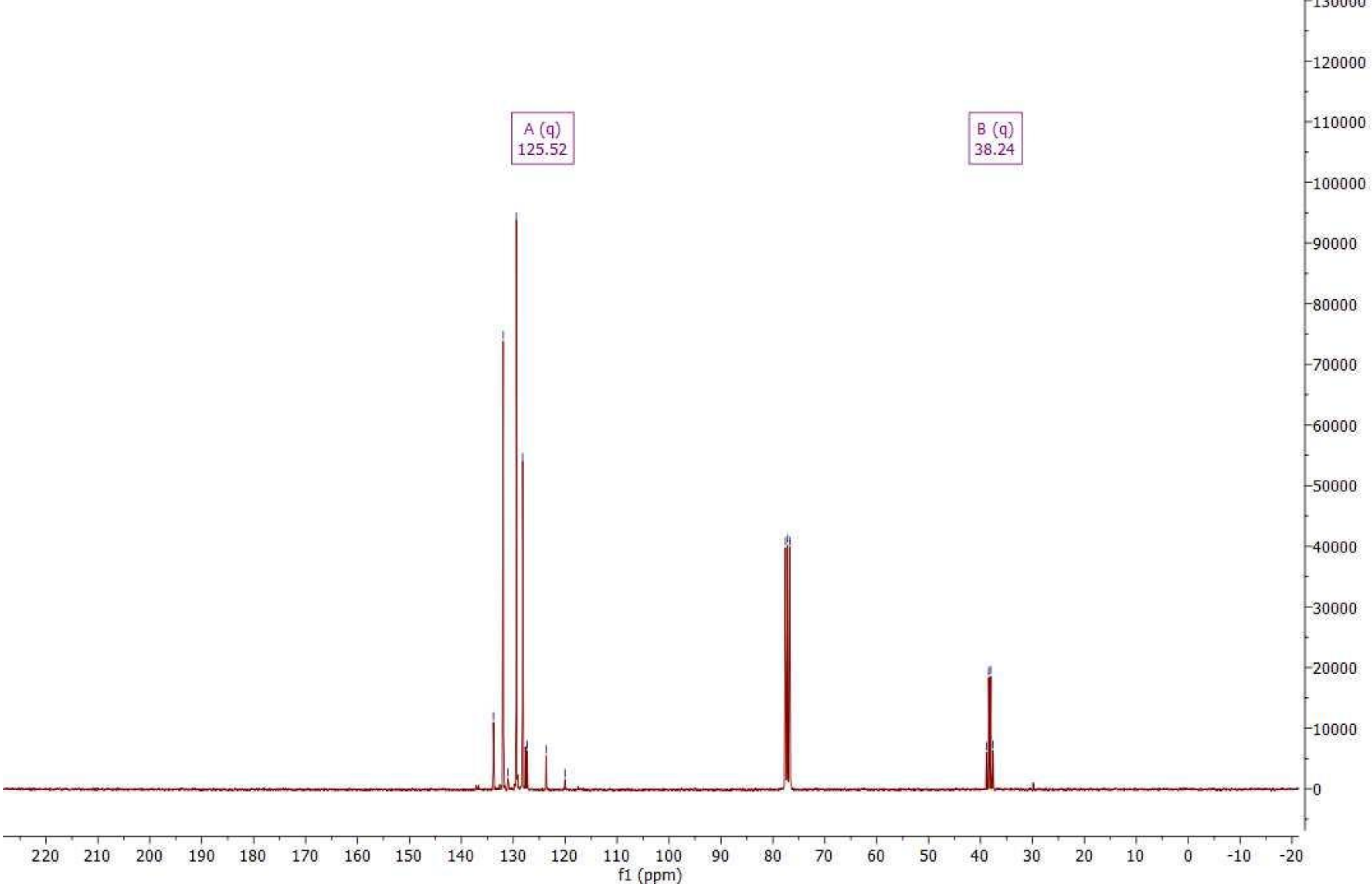

10i (^{19}F NMR in CDCl$_3$) (282 MHz)

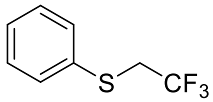

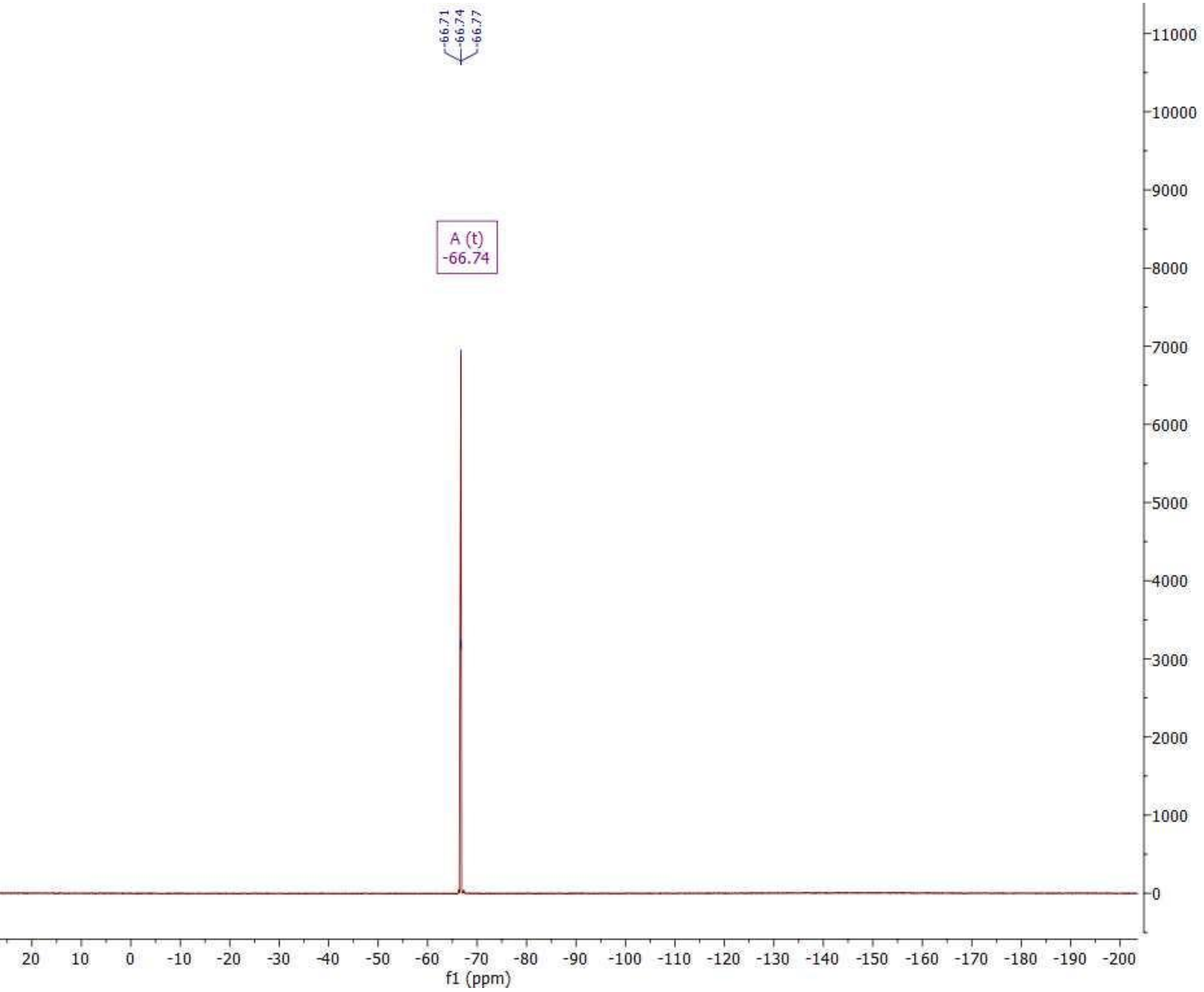

10j (^{1}H NMR in $CDCl_3$) (300 MHz)

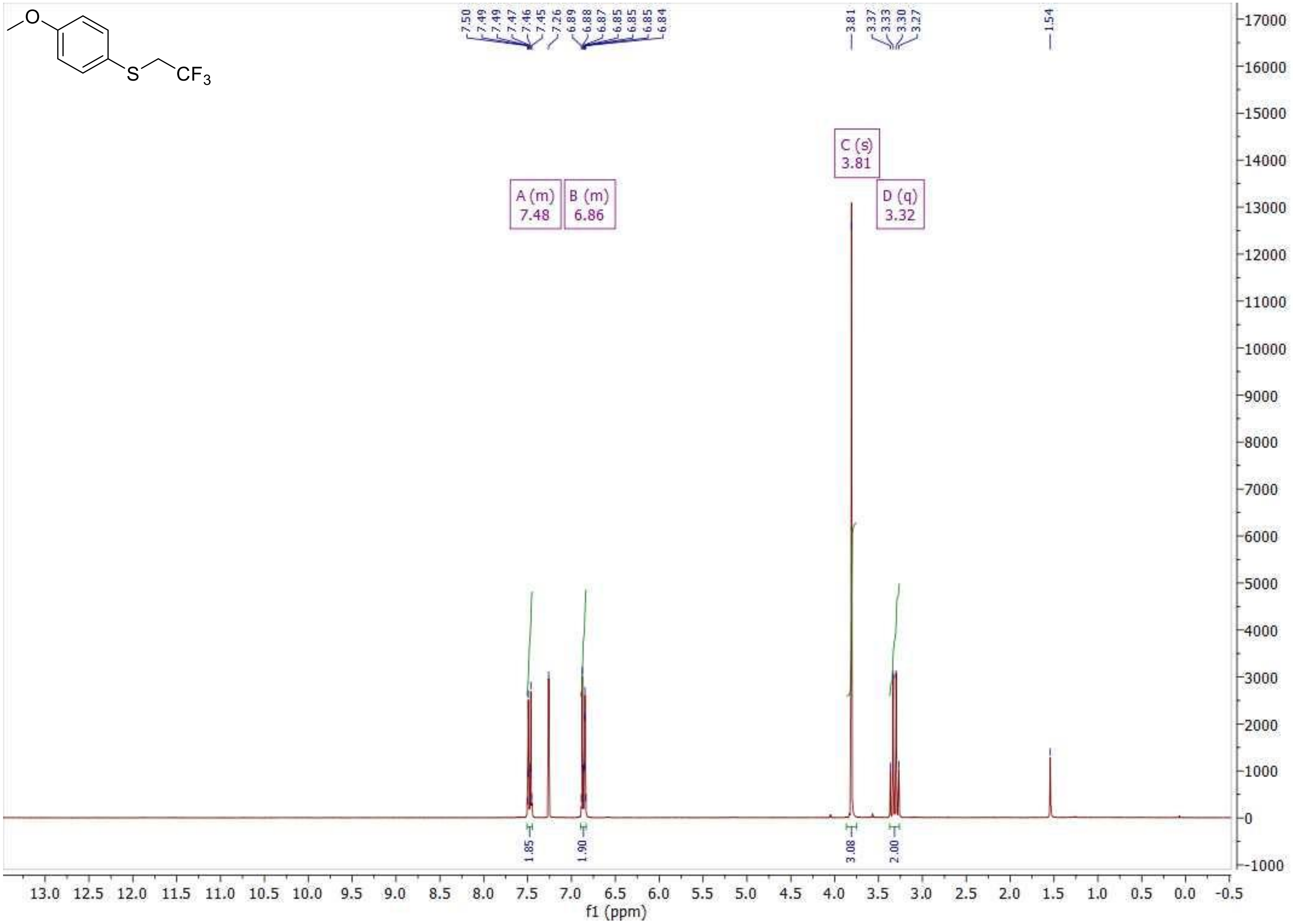

10j (^{13}C NMR in $CDCl_3$) (75 MHz)

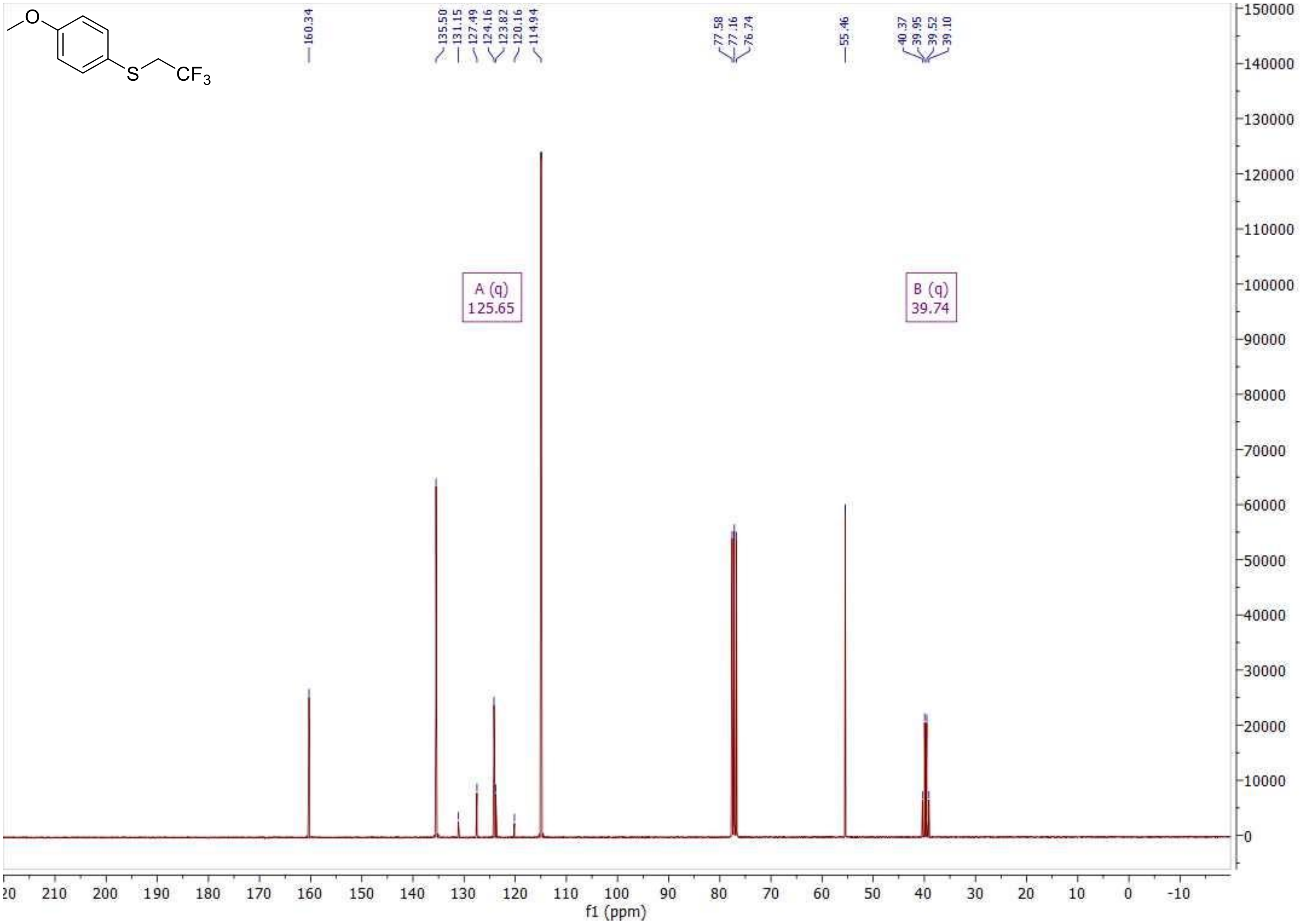

10j (^{19}F NMR in $CDCl_3$) (282 MHz)

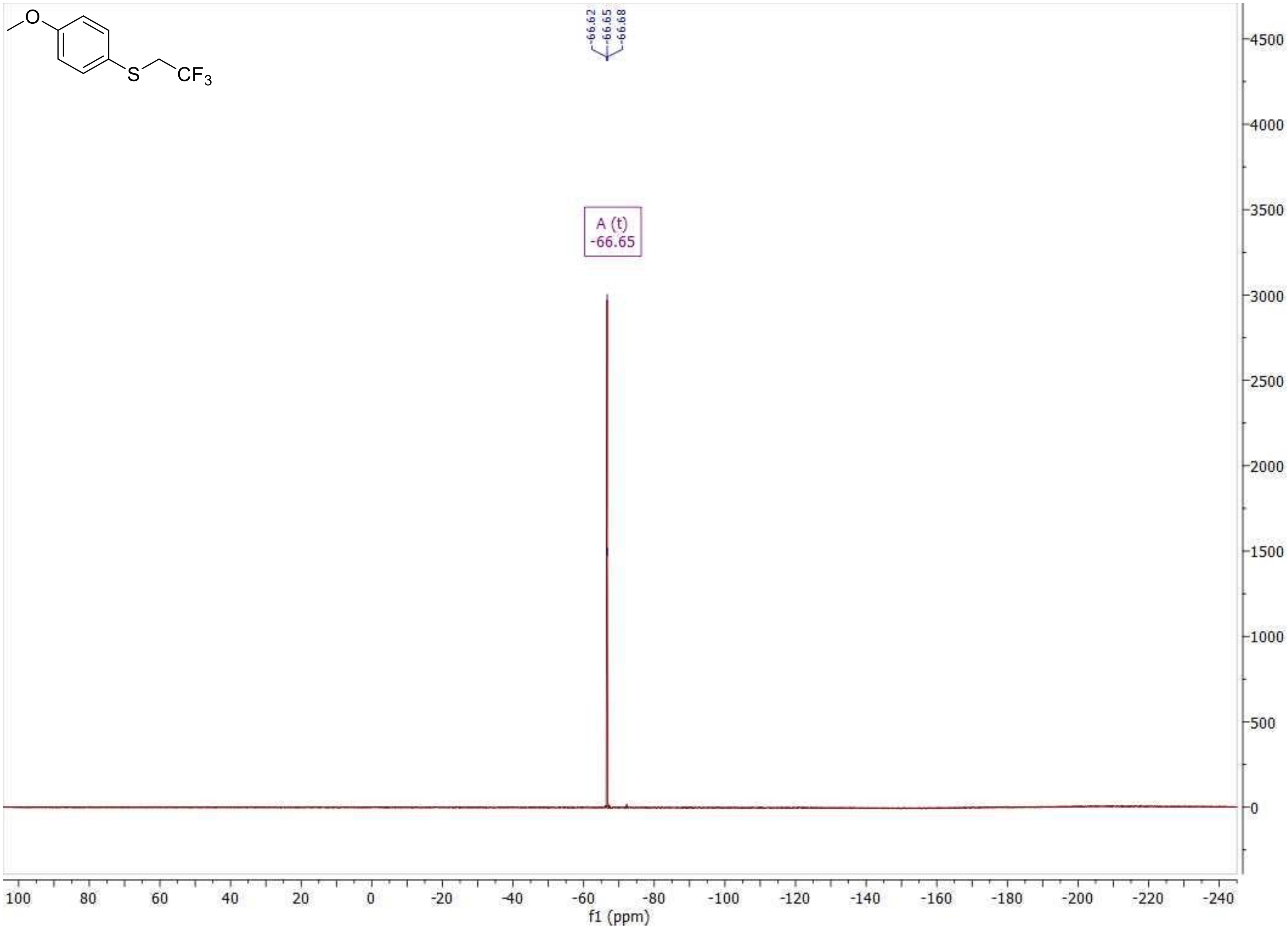

10k (^{1}H NMR in CD_2Cl_2) (300 MHz)

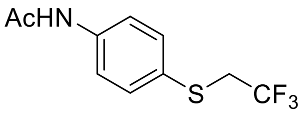

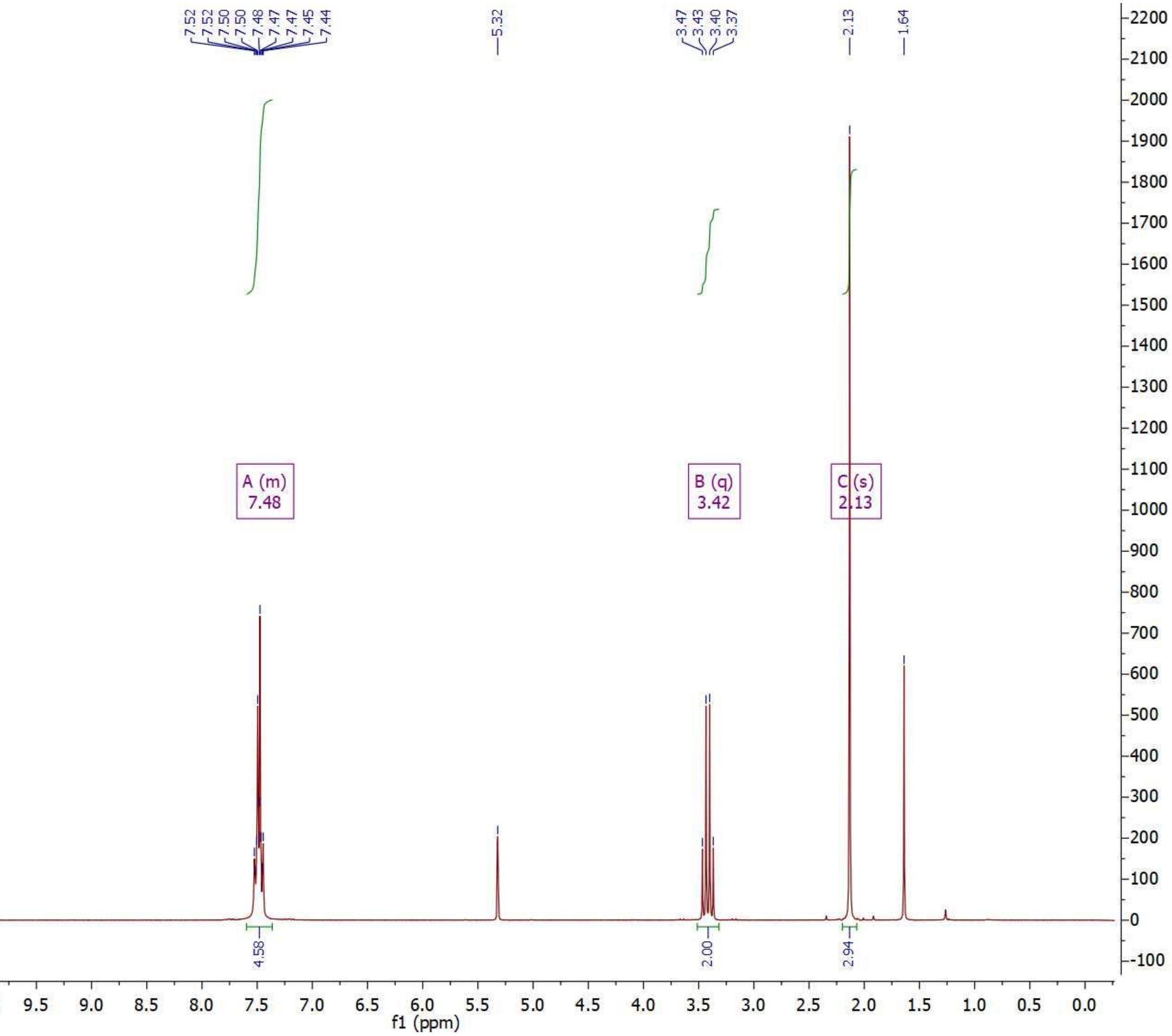

10k (^{13}C NMR in CD_2Cl_2) (75 MHz)

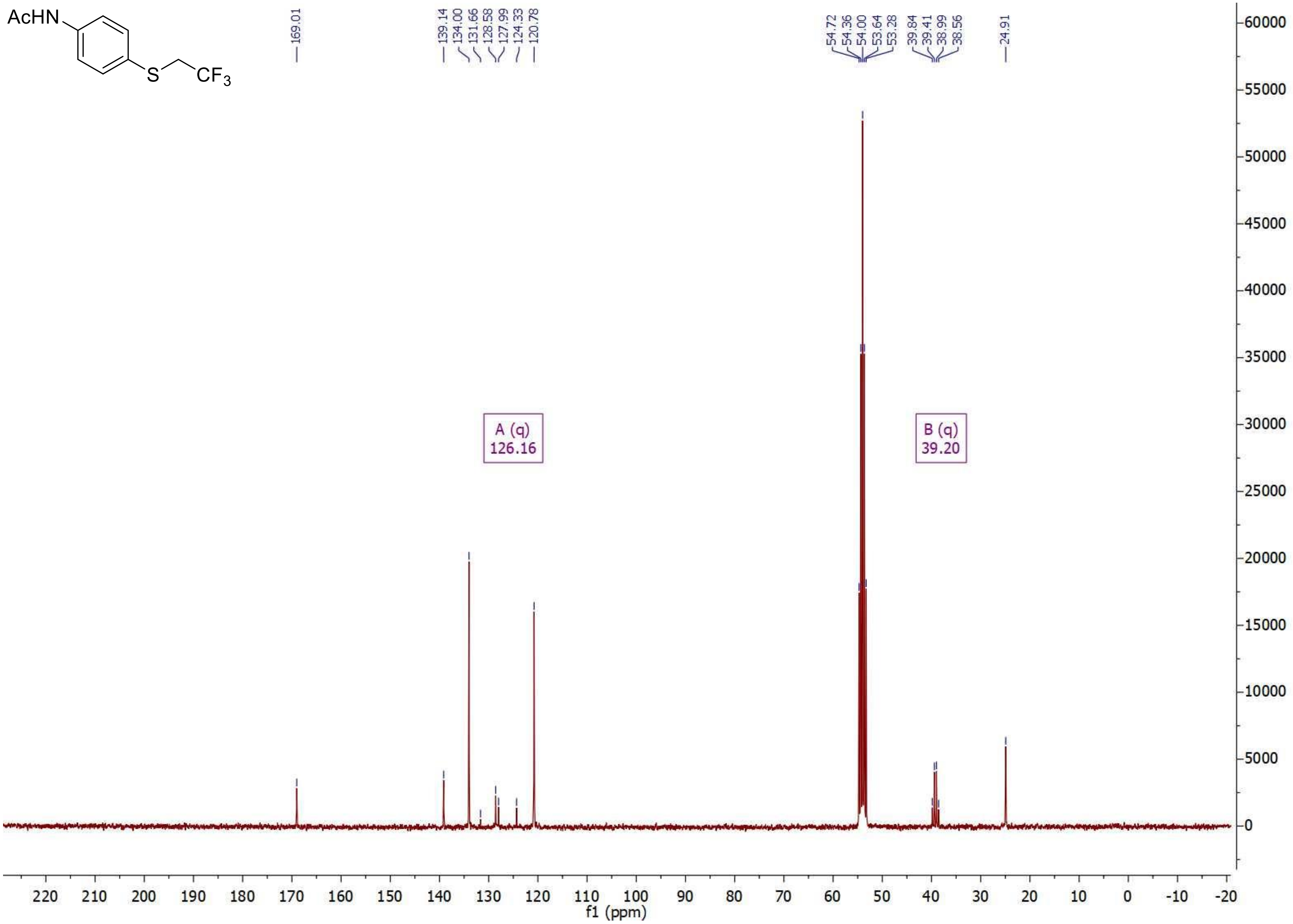

10k (^{19}F NMR in CD_2Cl_2) (282 MHz)

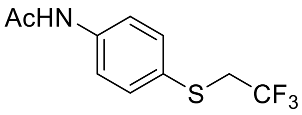

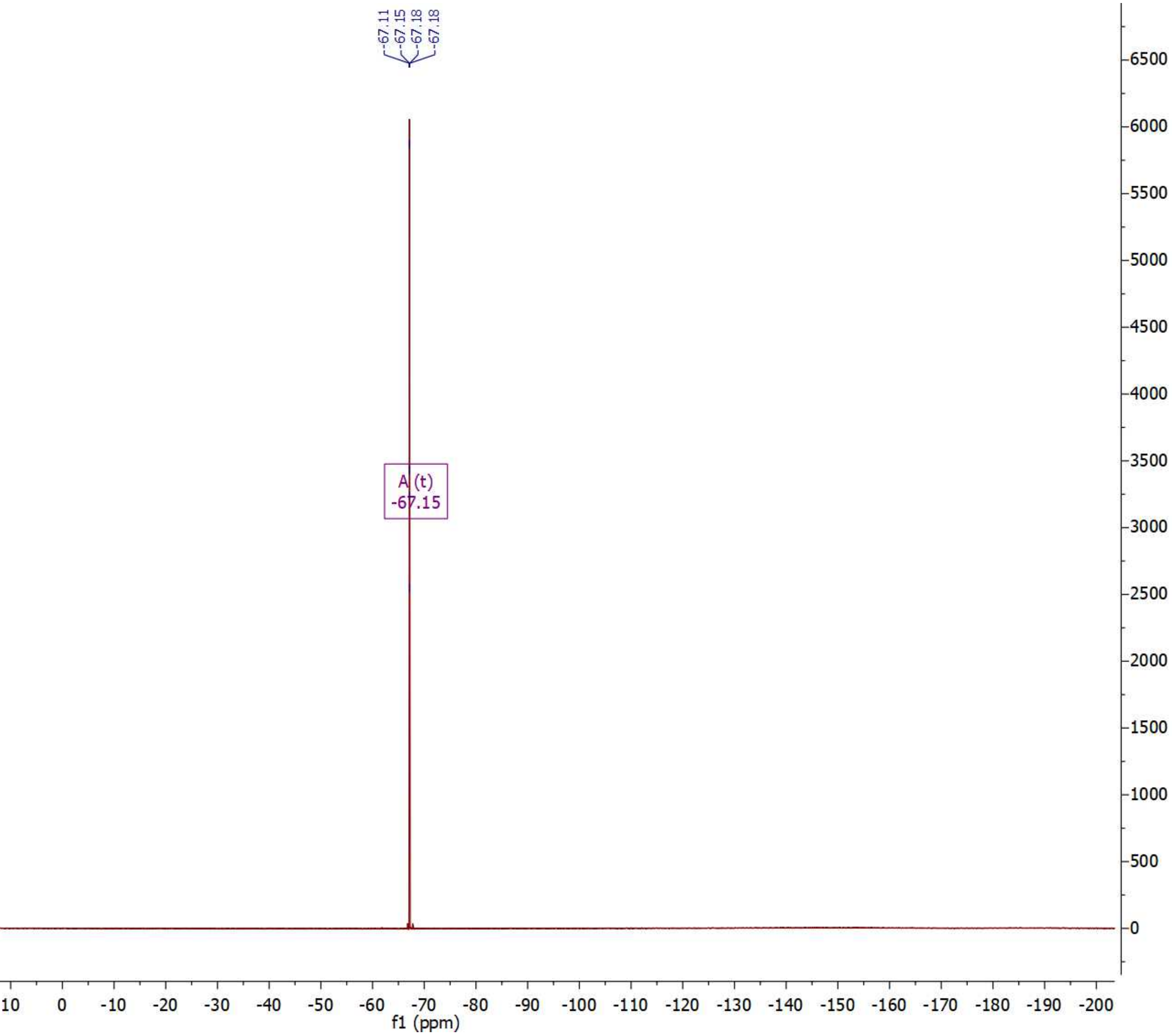

10l (^{1}H NMR in $CDCl_3$) (300 MHz)

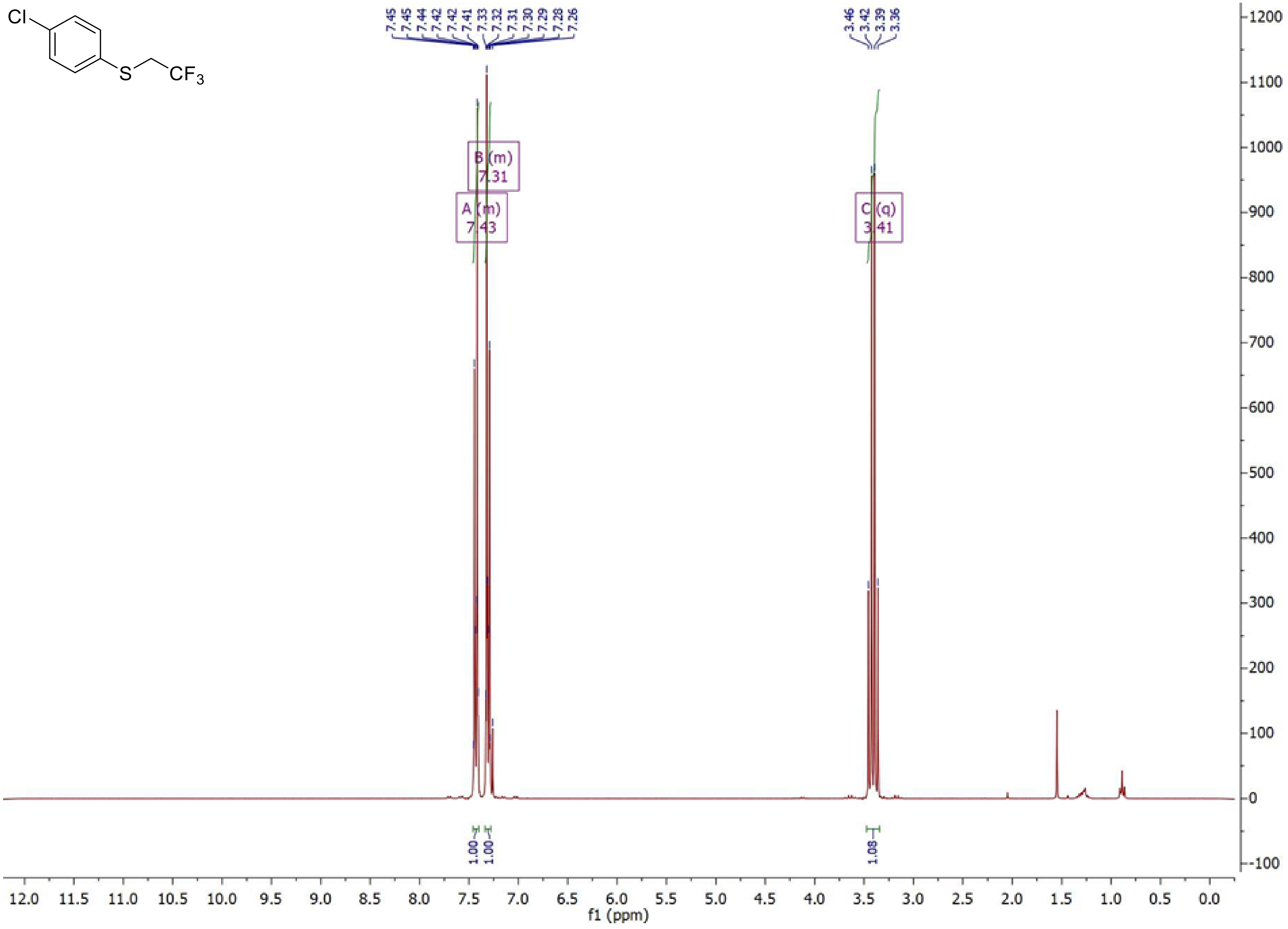

10l (^{13}C NMR in $CDCl_3$) (101 MHz)

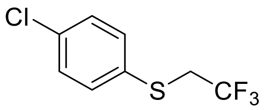

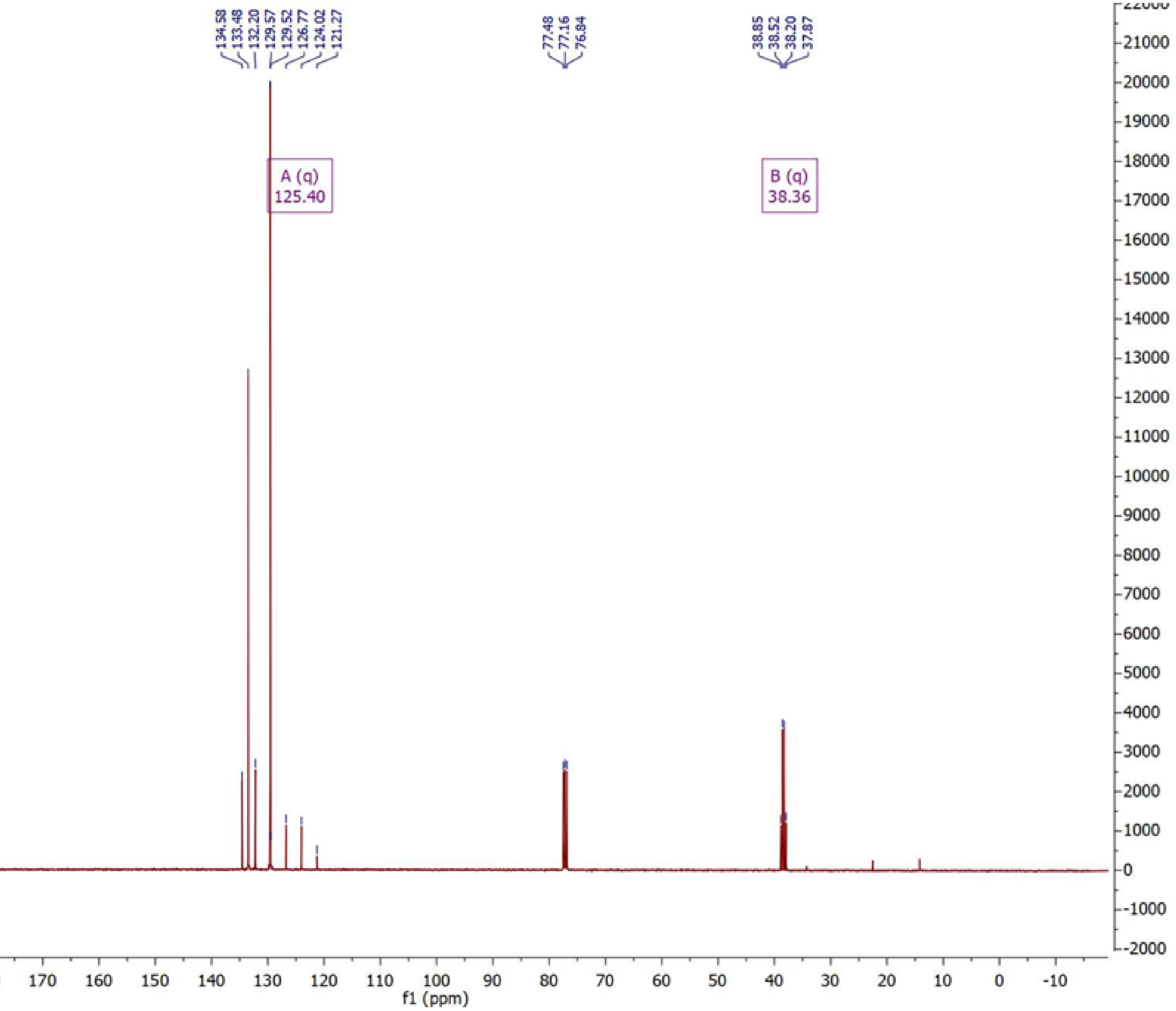

10l (^{19}F NMR in $CDCl_3$) (282 MHz)

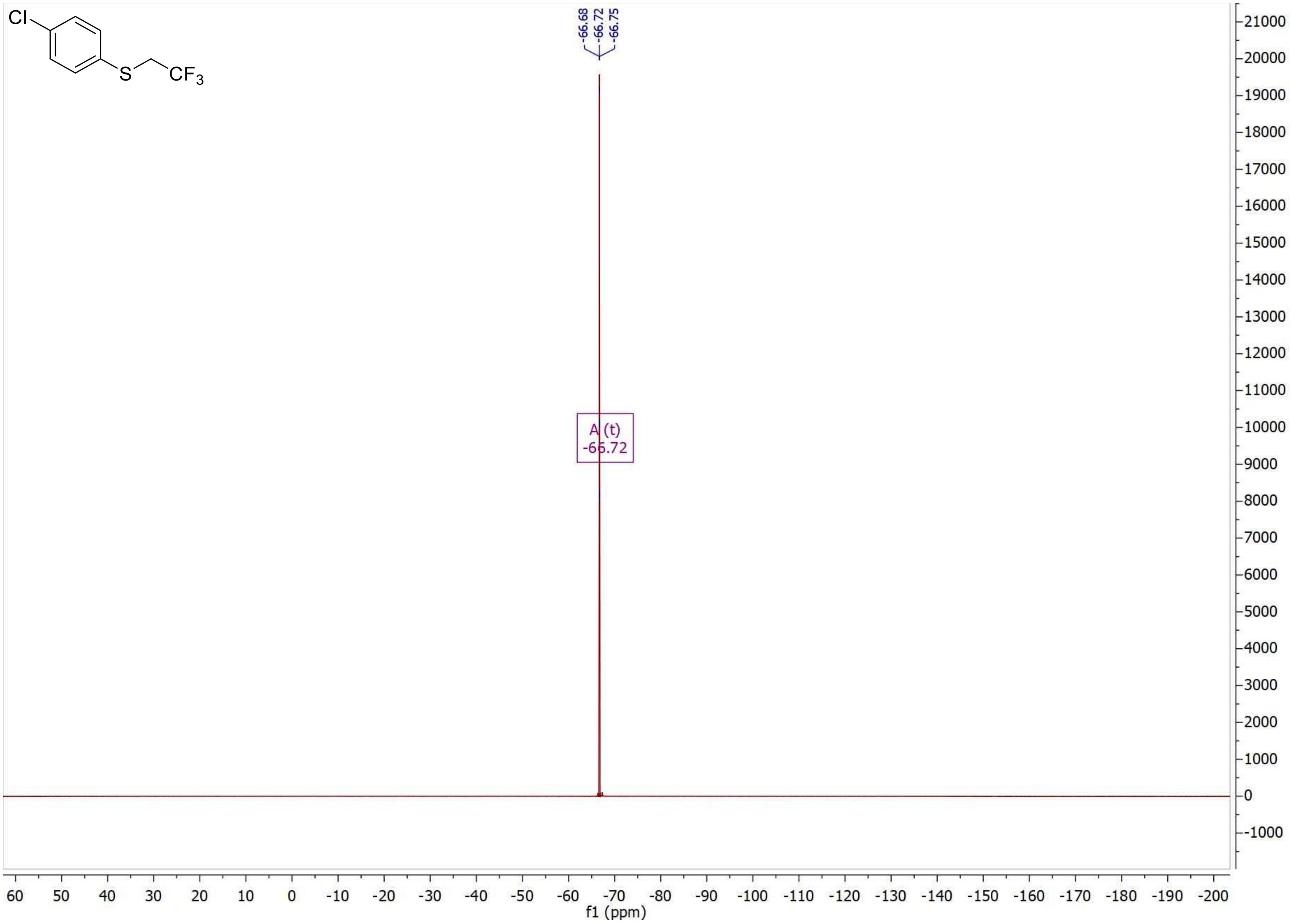

10m (^{1}H NMR in $CDCl_3$) (300 MHz)

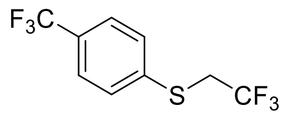

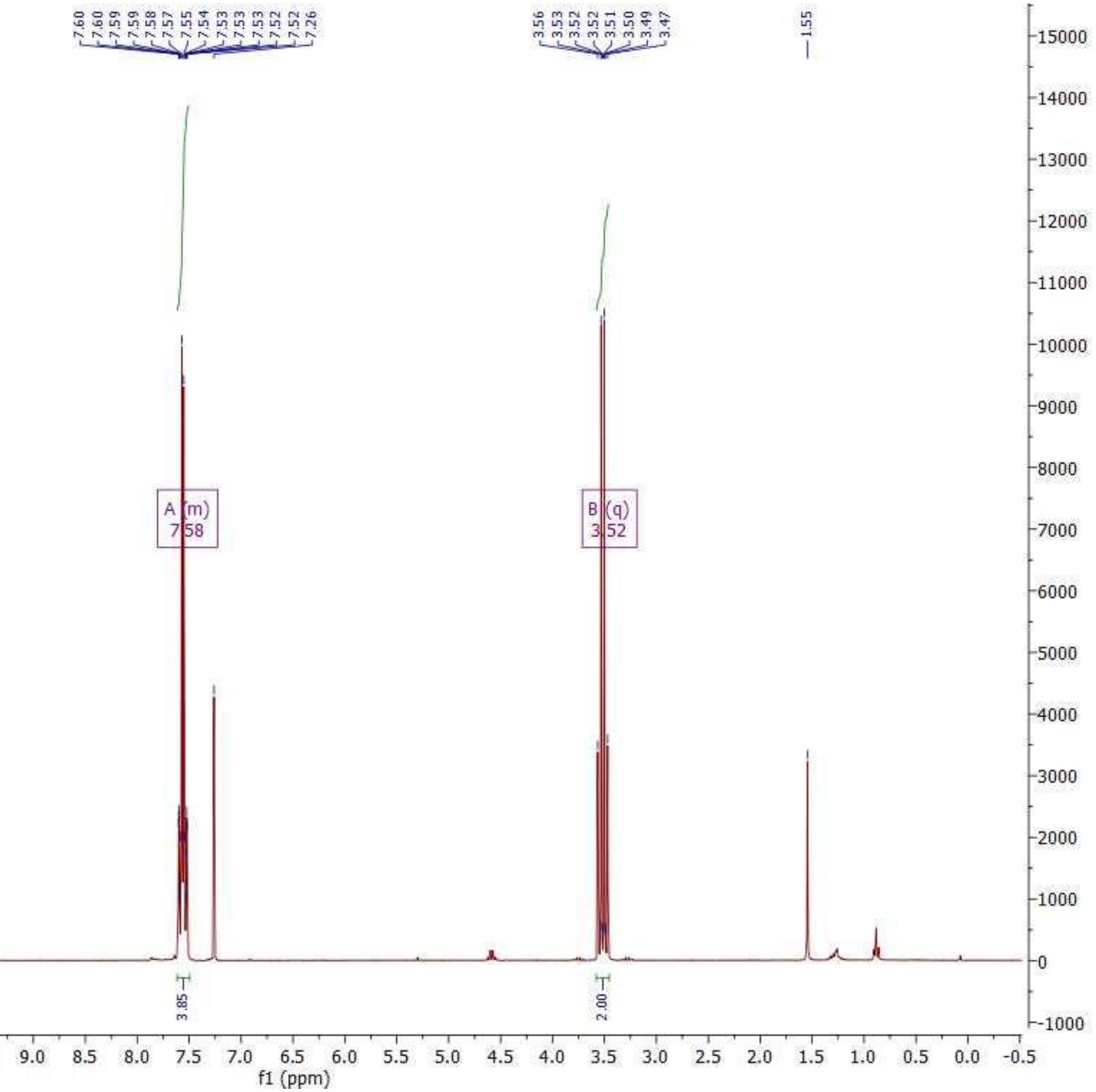

10m (^{13}C NMR in $CDCl_3$) (75 MHz)

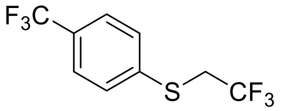

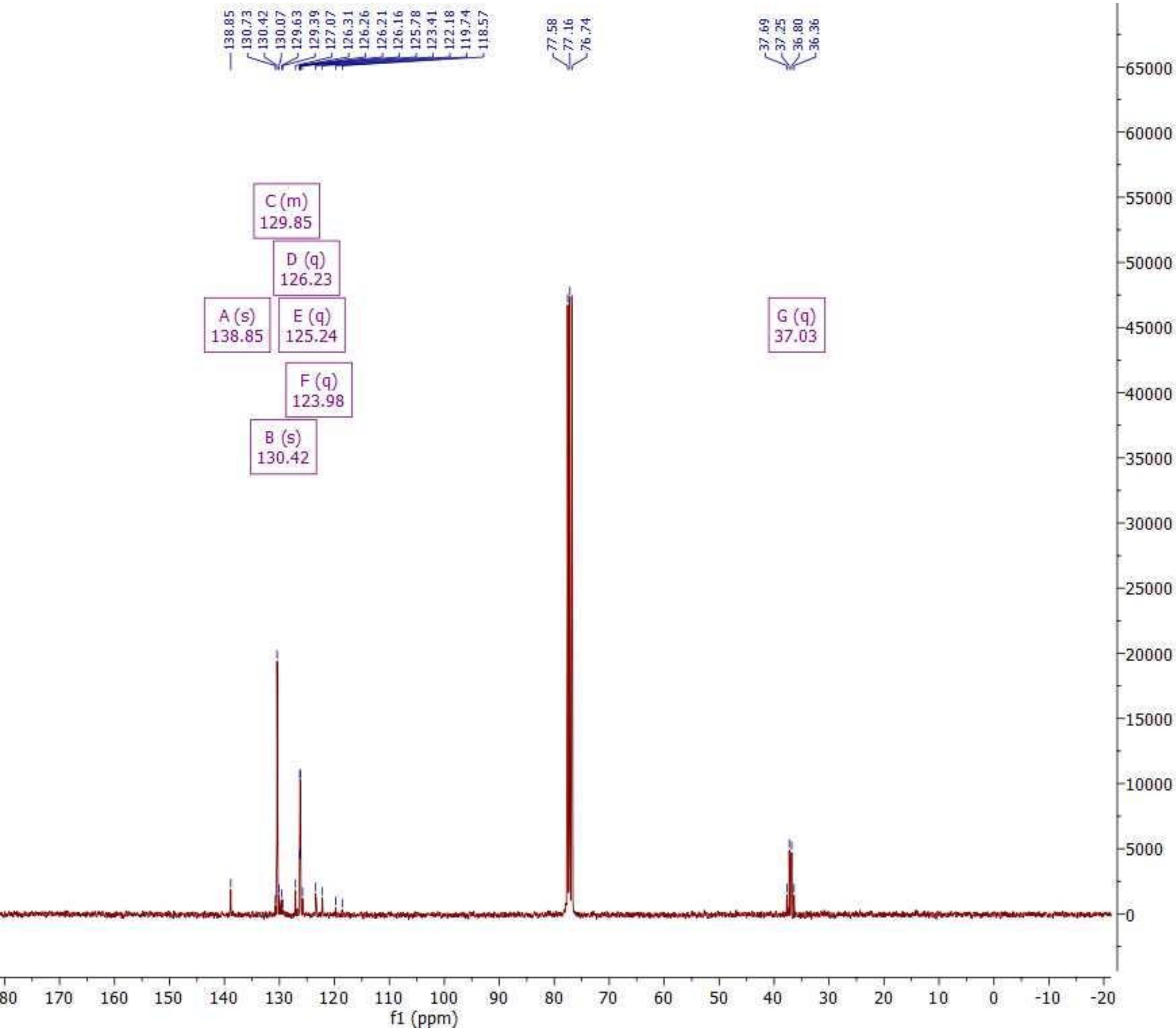

10m (^{19}F NMR in $CDCl_3$) (282 MHz)

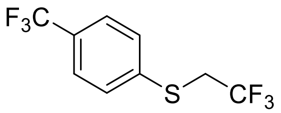

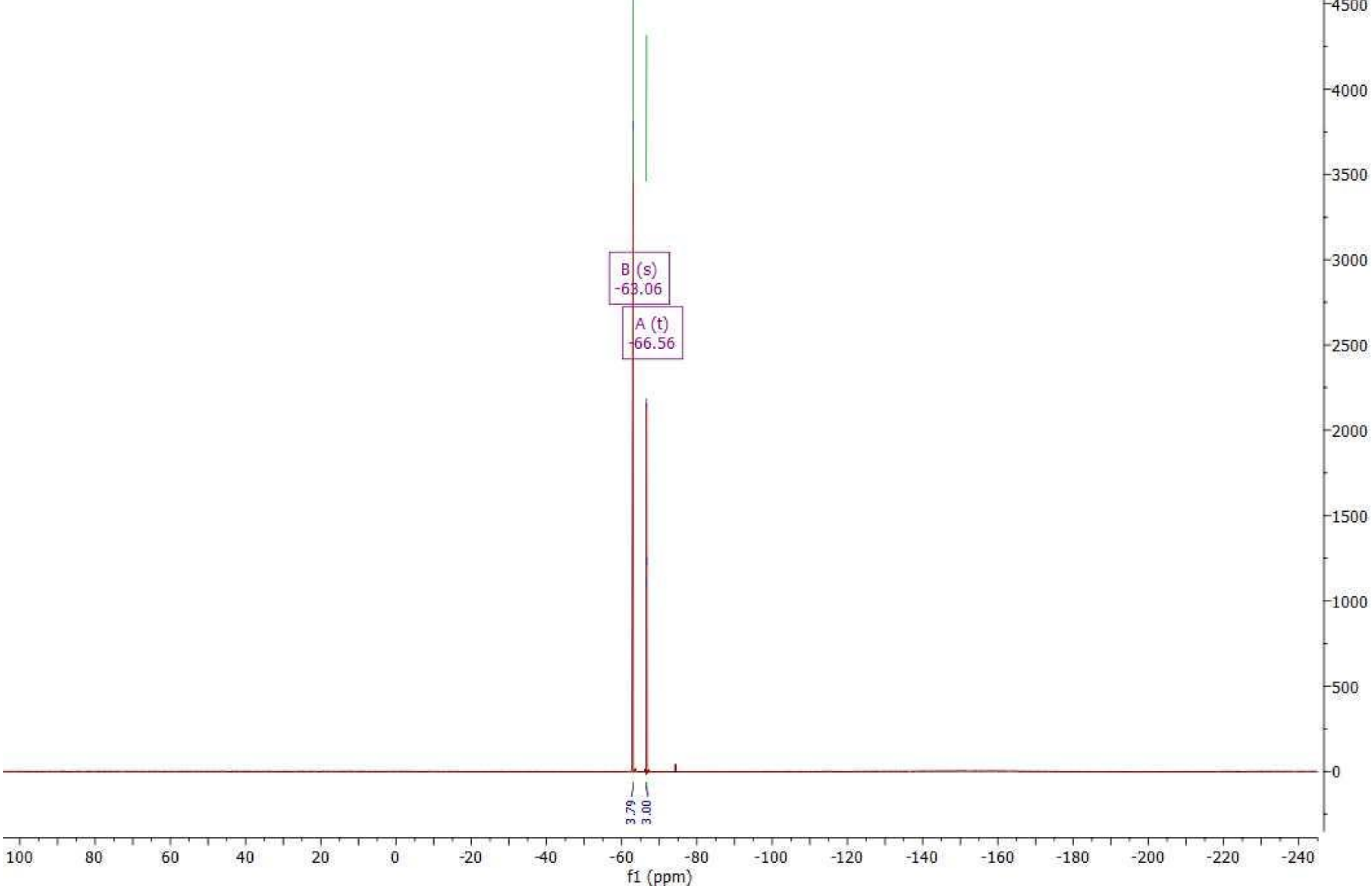

10n (^1H NMR in $CDCl_3$) (300 MHz)

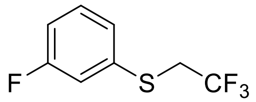

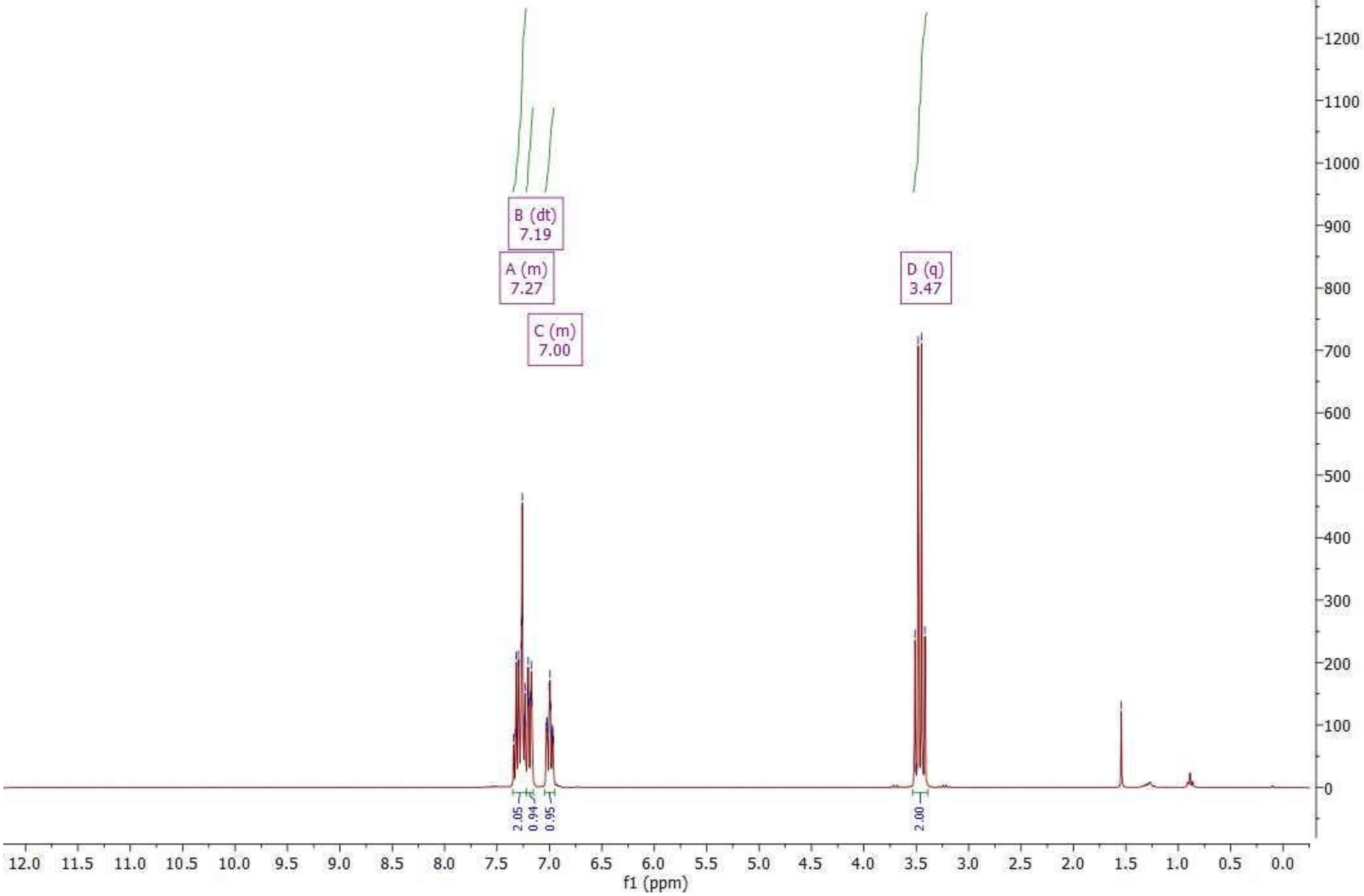

10n (^{13}C NMR in $CDCl_3$) (75 MHz)

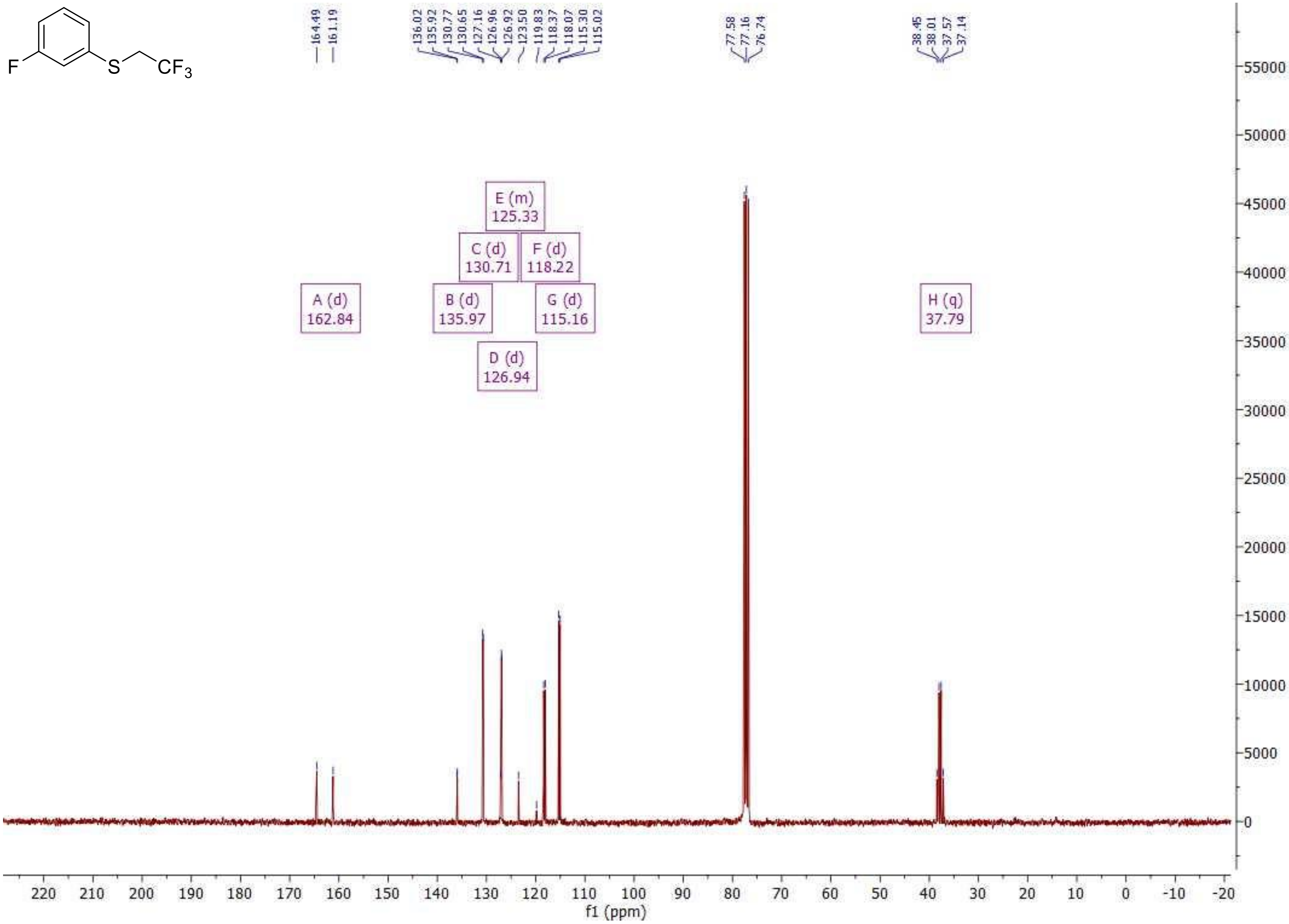

10n (^{19}F NMR in $CDCl_3$) (282 MHz)

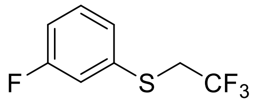

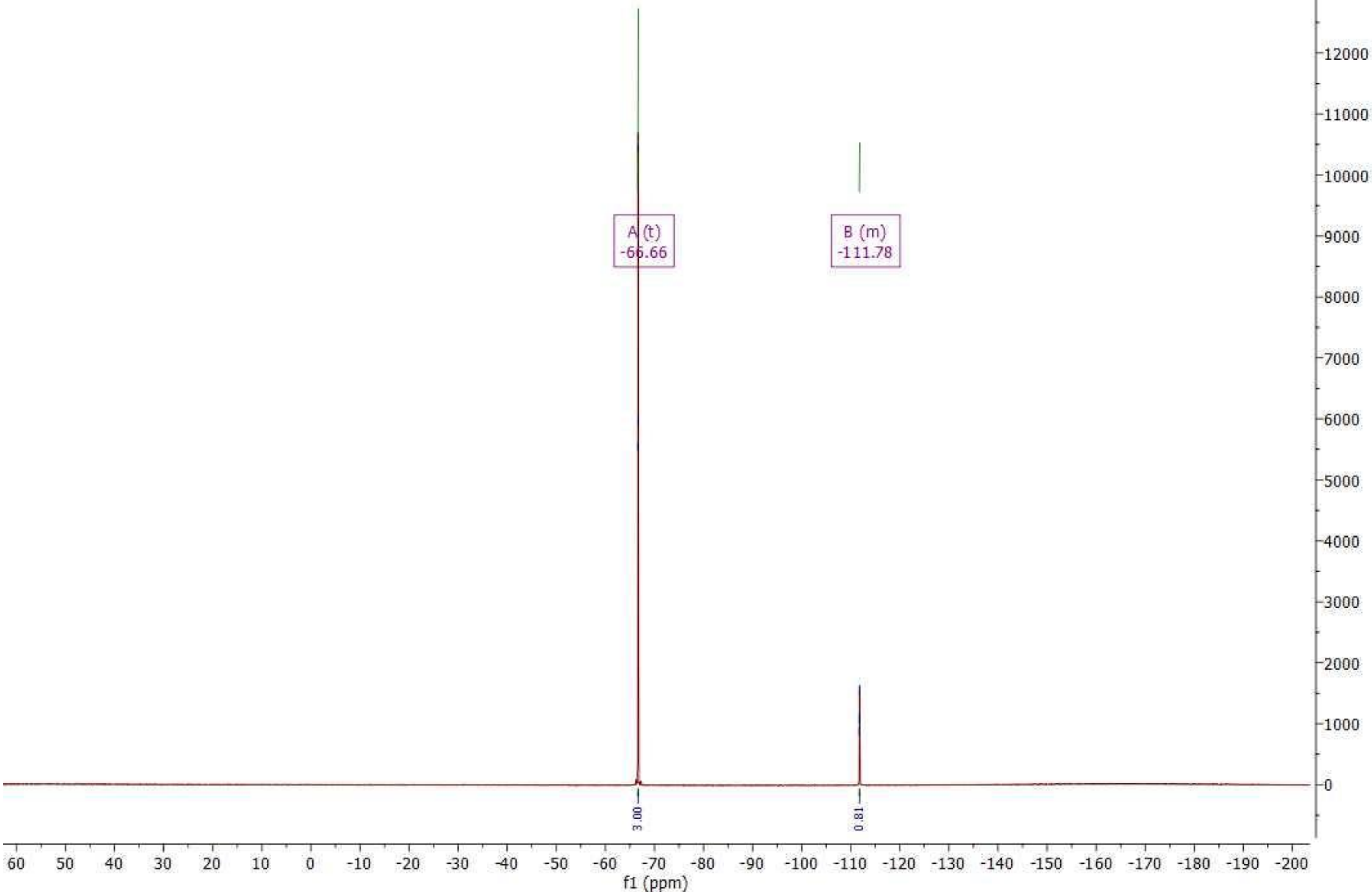

10o (^{1}H NMR in $CDCl_3$) (300 MHz)

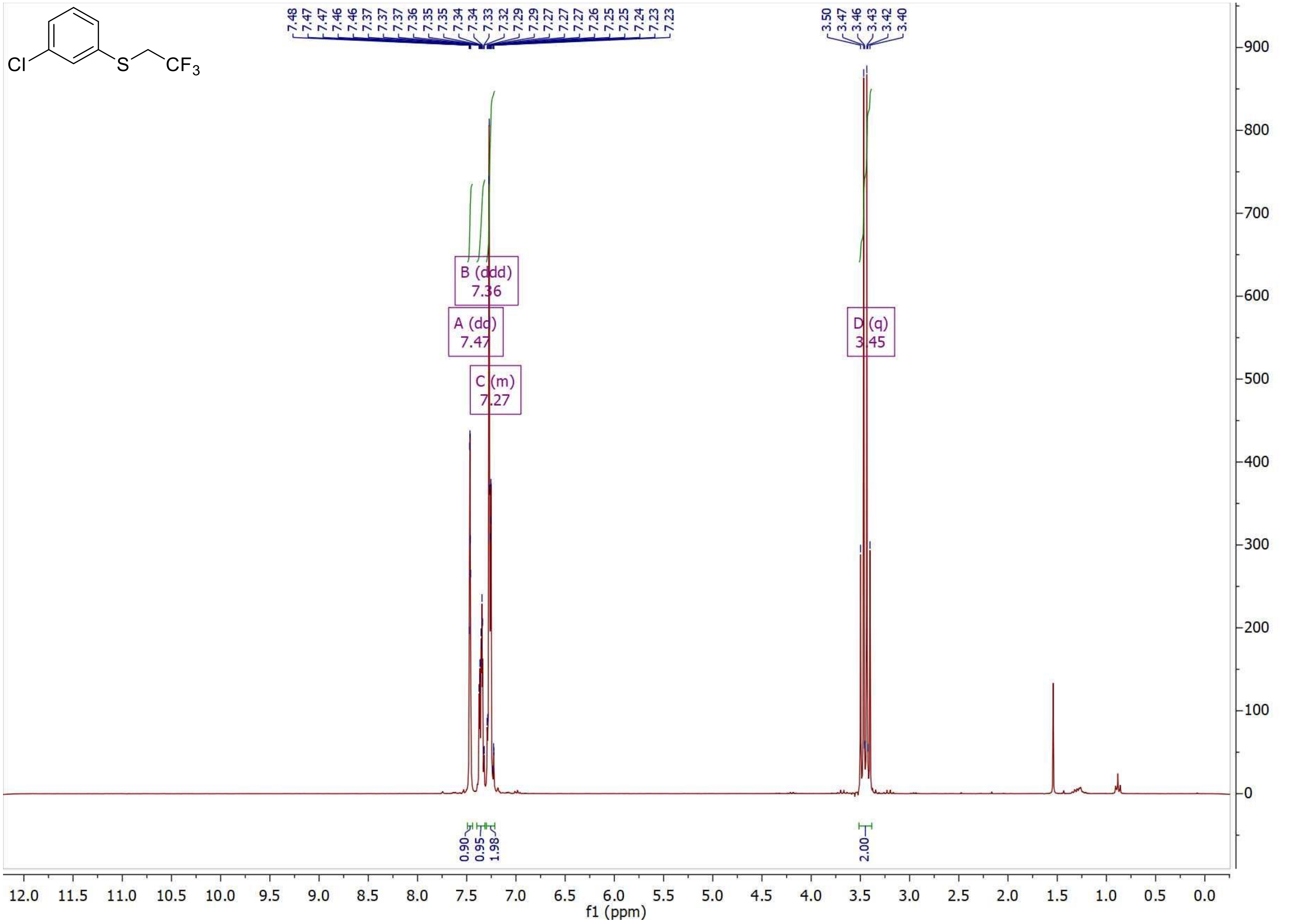

10o (^{13}C NMR in $CDCl_3$) (101 MHz)

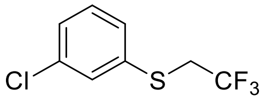

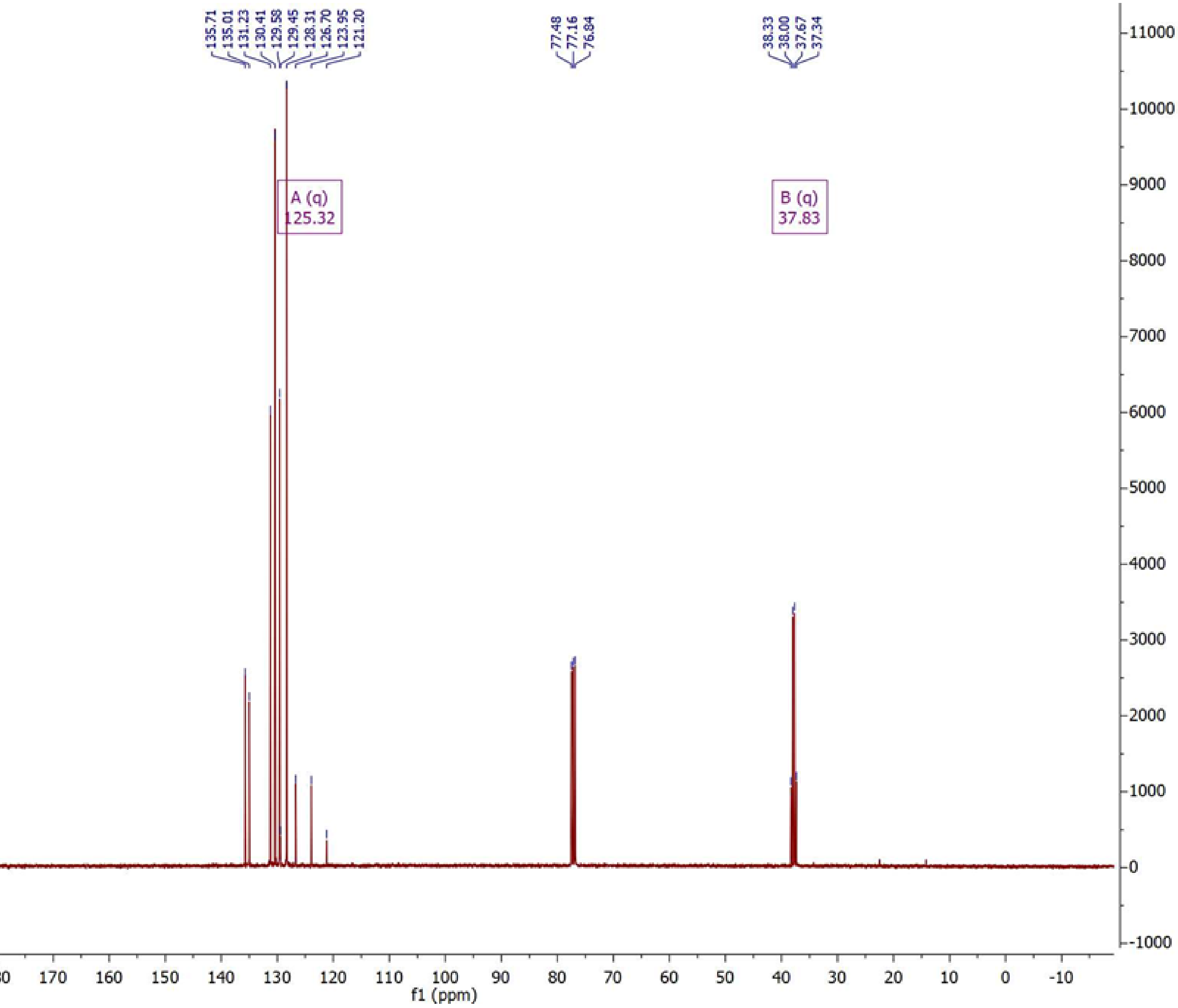

10o (^{19}F NMR in $CDCl_3$) (282 MHz)

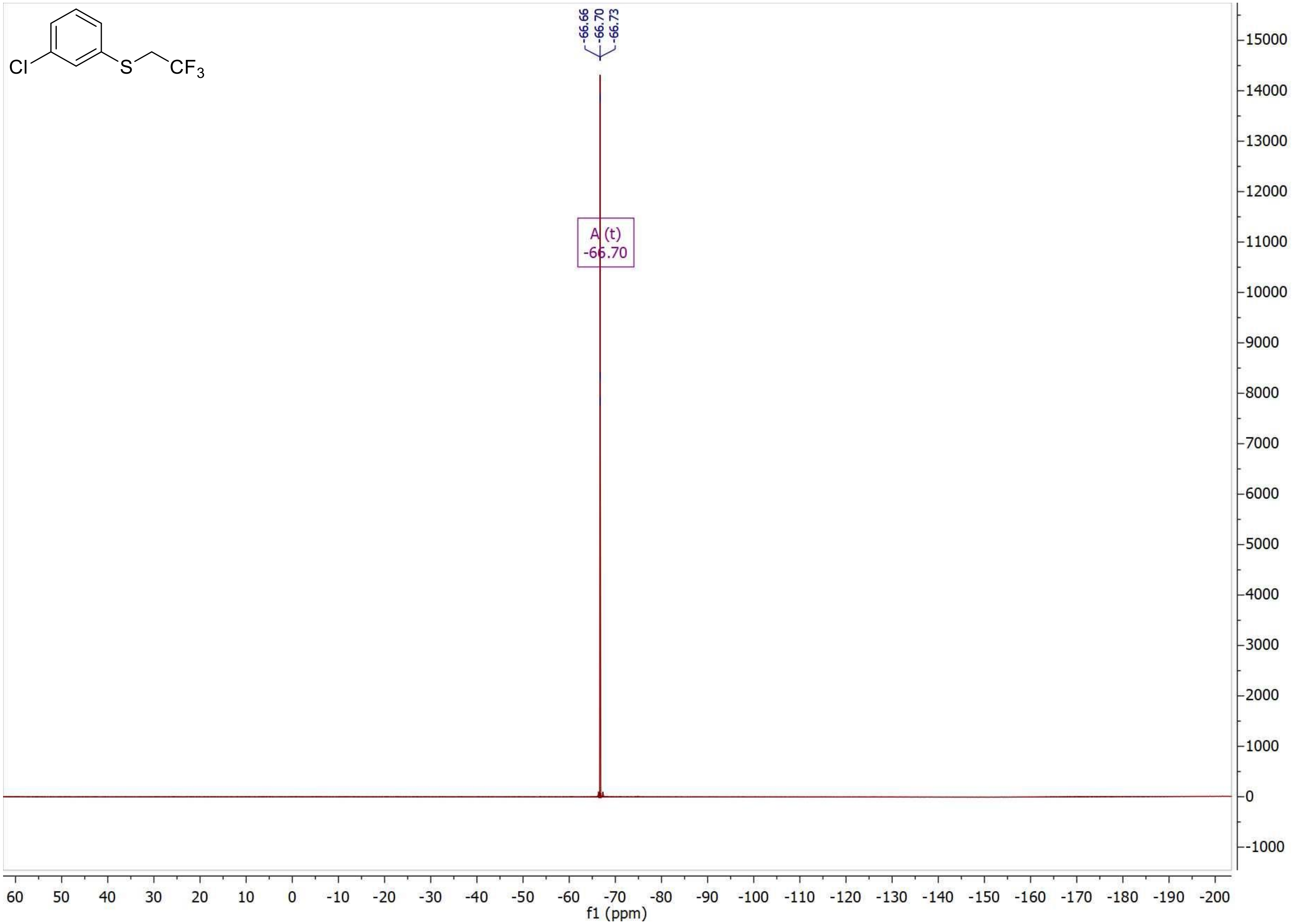

10p (^{1}H NMR in $CDCl_3$) (300 MHz)

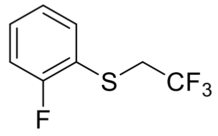

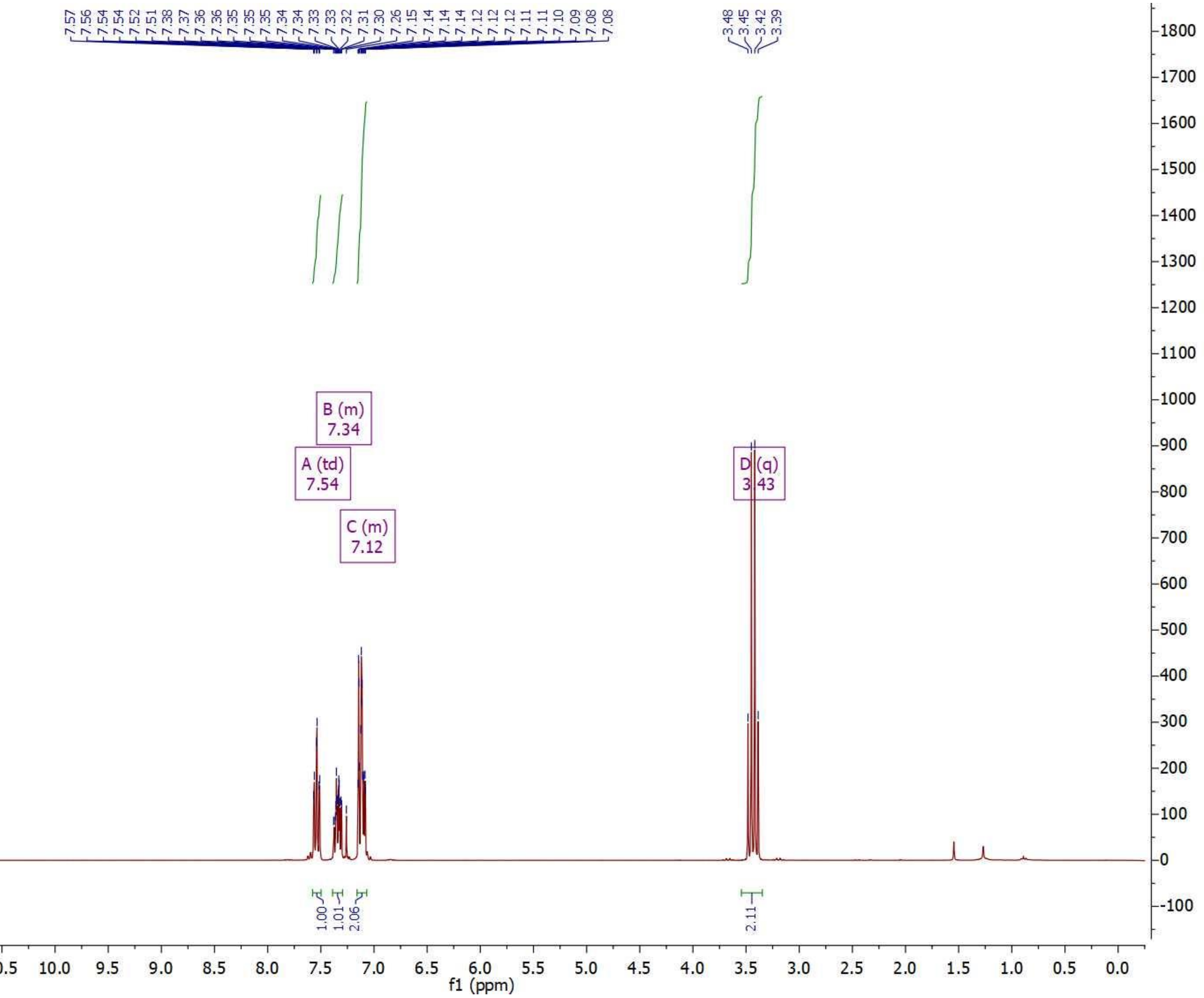

10p (^{13}C NMR in $CDCl_3$) (75 MHz)

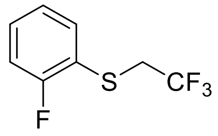

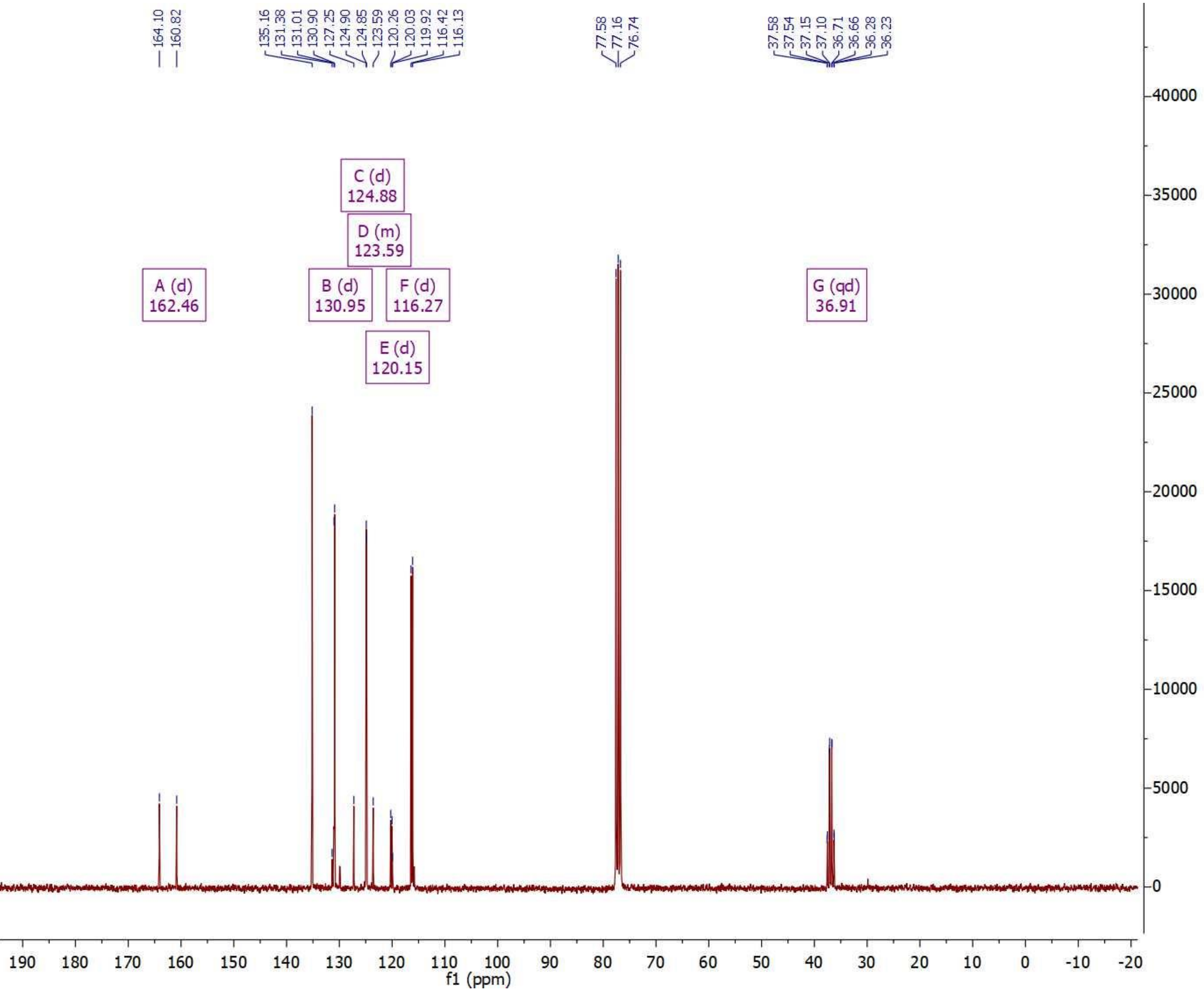

10p (^{19}F NMR in $CDCl_3$) (282 MHz)

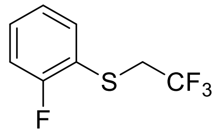

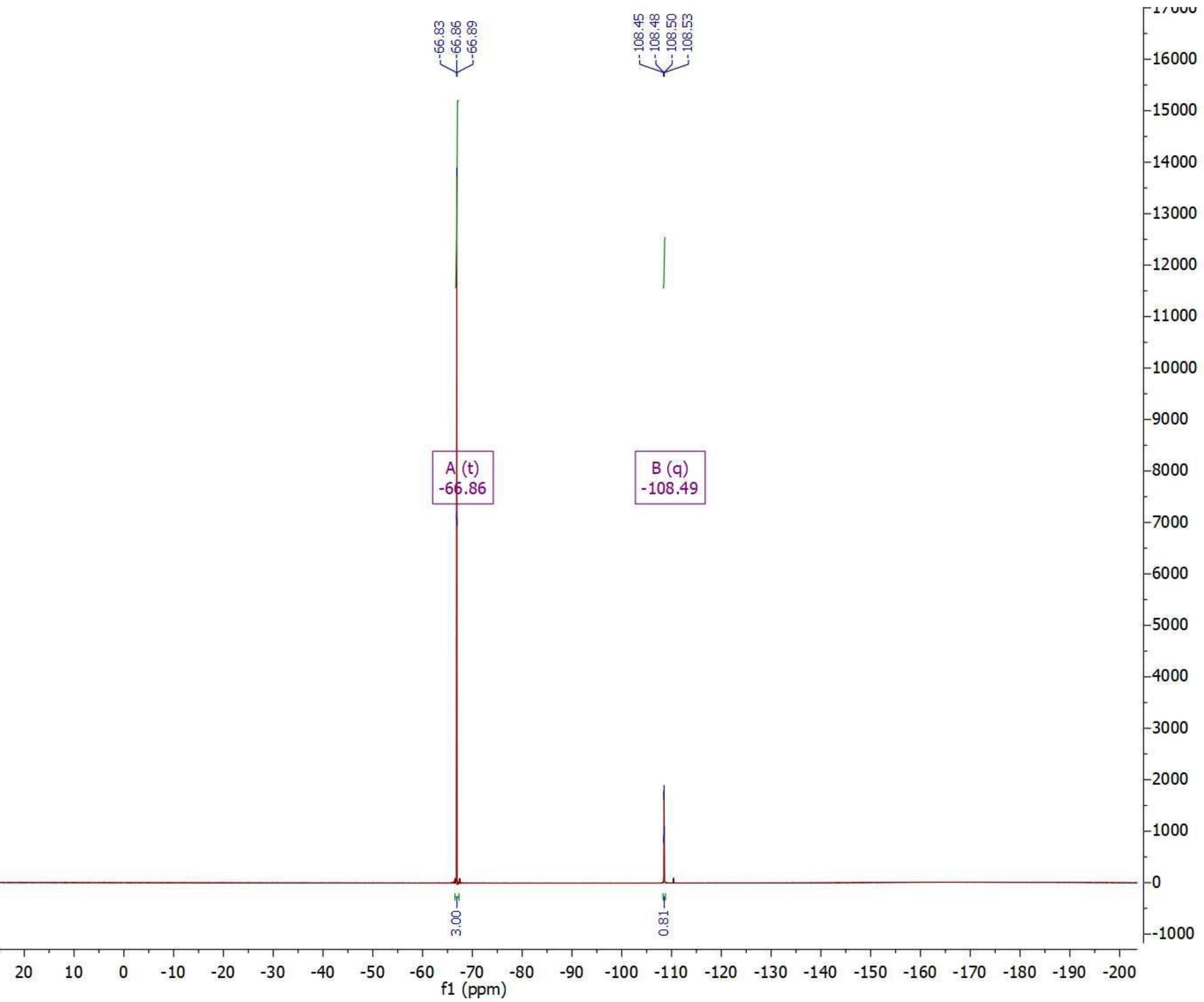

10q (^{1}H NMR in $CDCl_3$) (300 MHz)

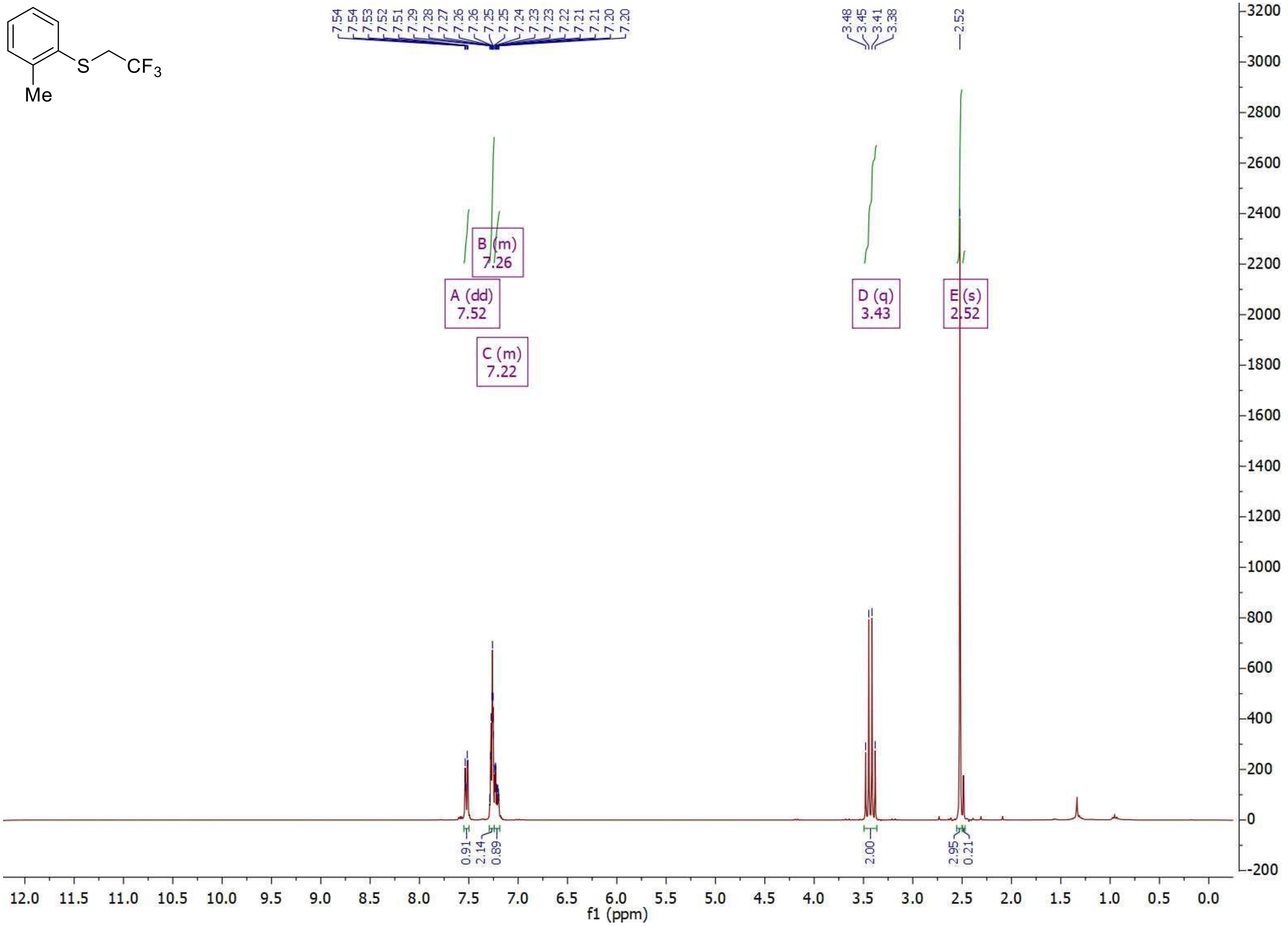

10q (^{13}C NMR in $CDCl_3$) (75 MHz)

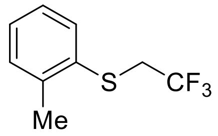

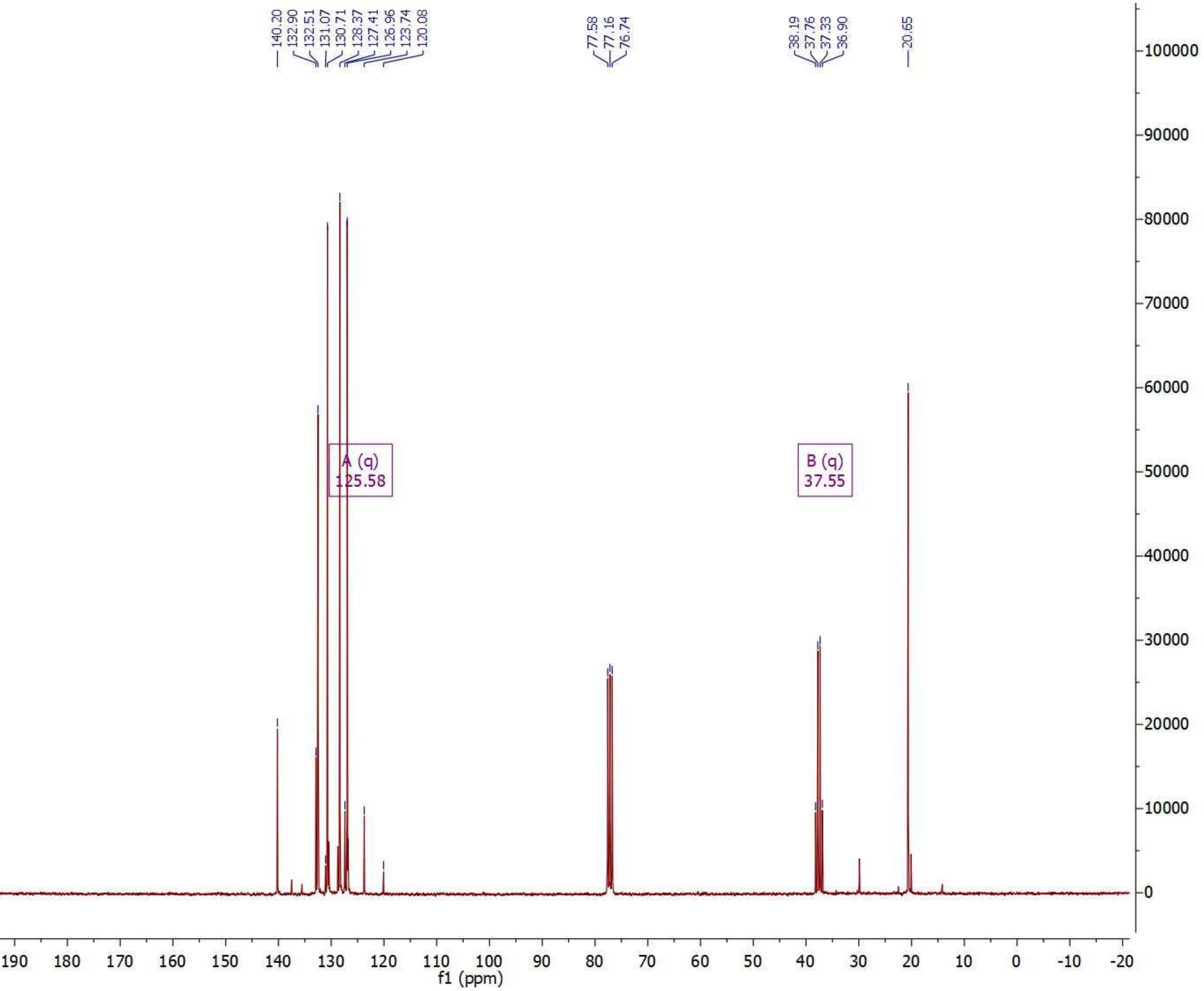

10q (^{19}F NMR in $CDCl_3$) (282 MHz)

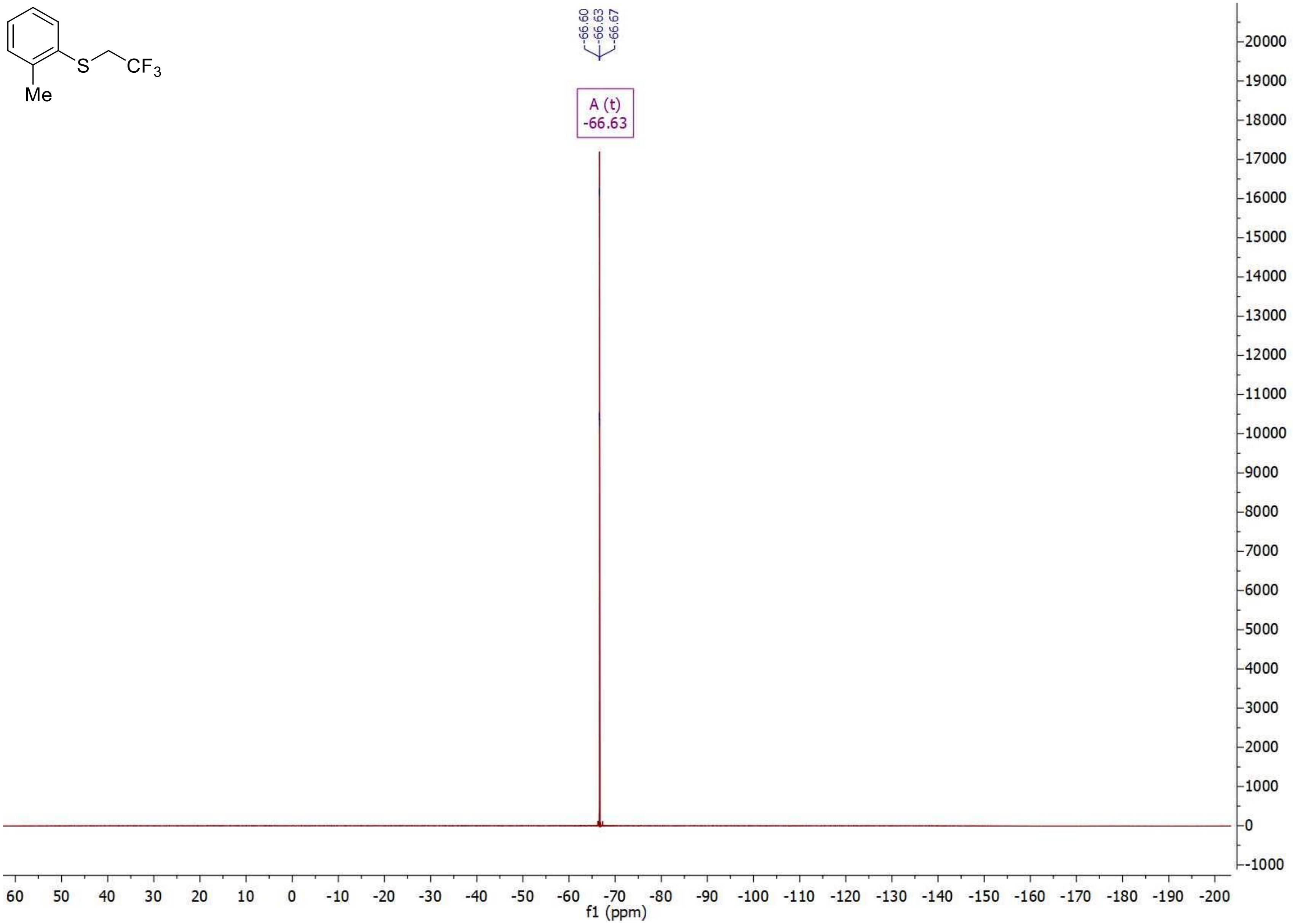

10r (^{1}H NMR in $CDCl_3$) (300 MHz)

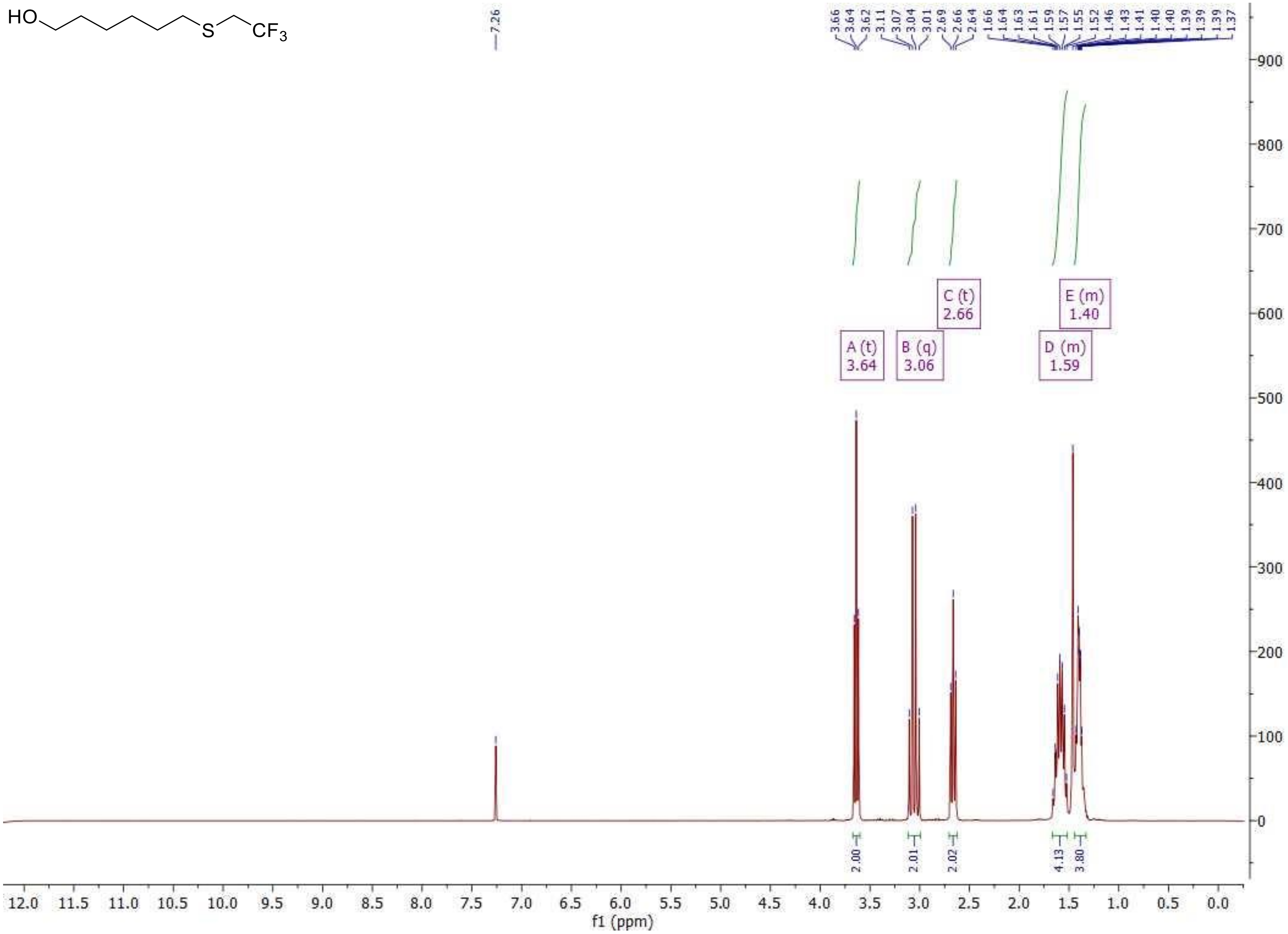

10r (^{13}C NMR in $CDCl_3$) (75 MHz)

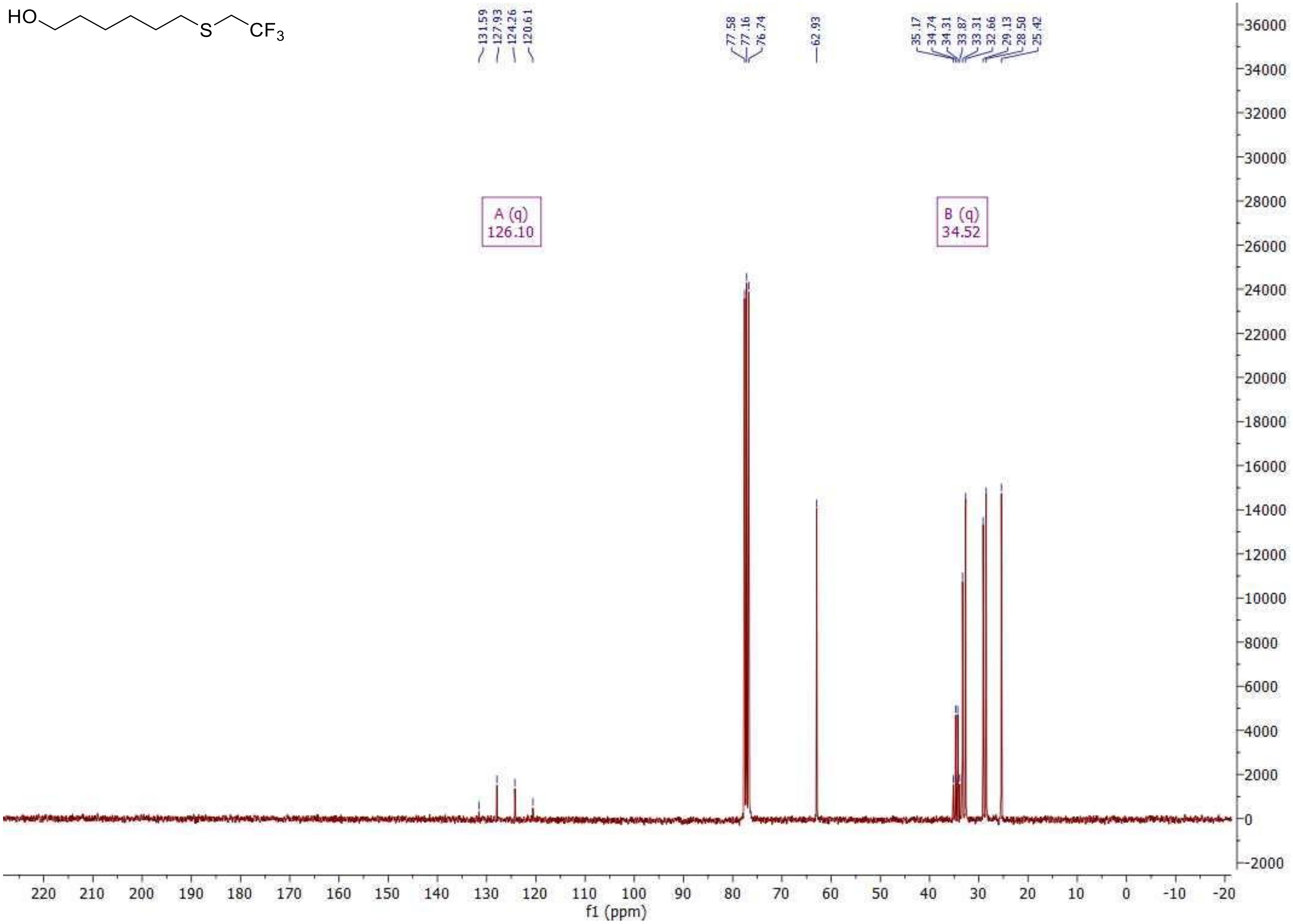

10r (^{19}F NMR in $CDCl_3$) (282 MHz)

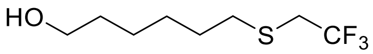

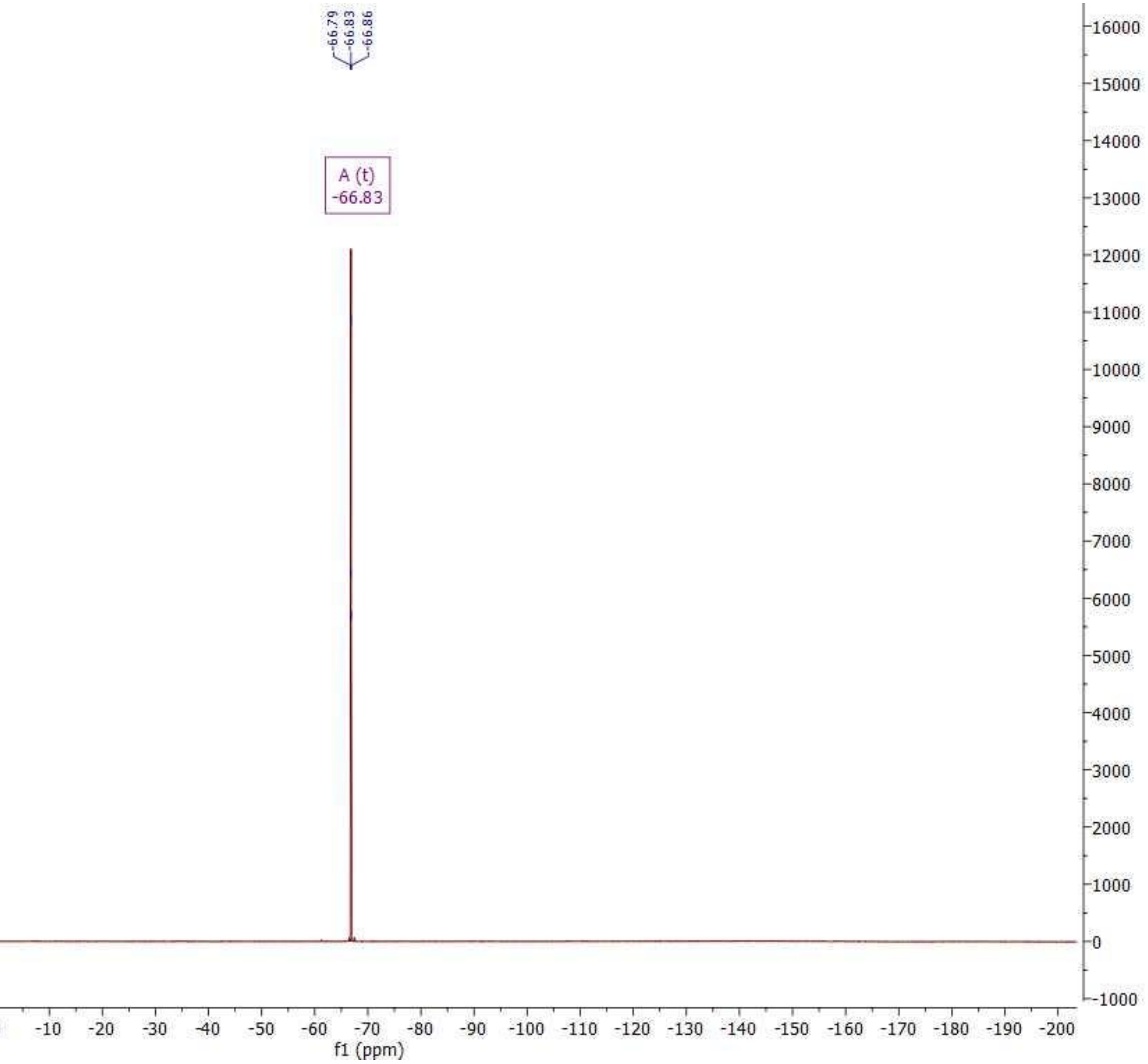

10s (^{1}H NMR in $CDCl_3$) (300 MHz)

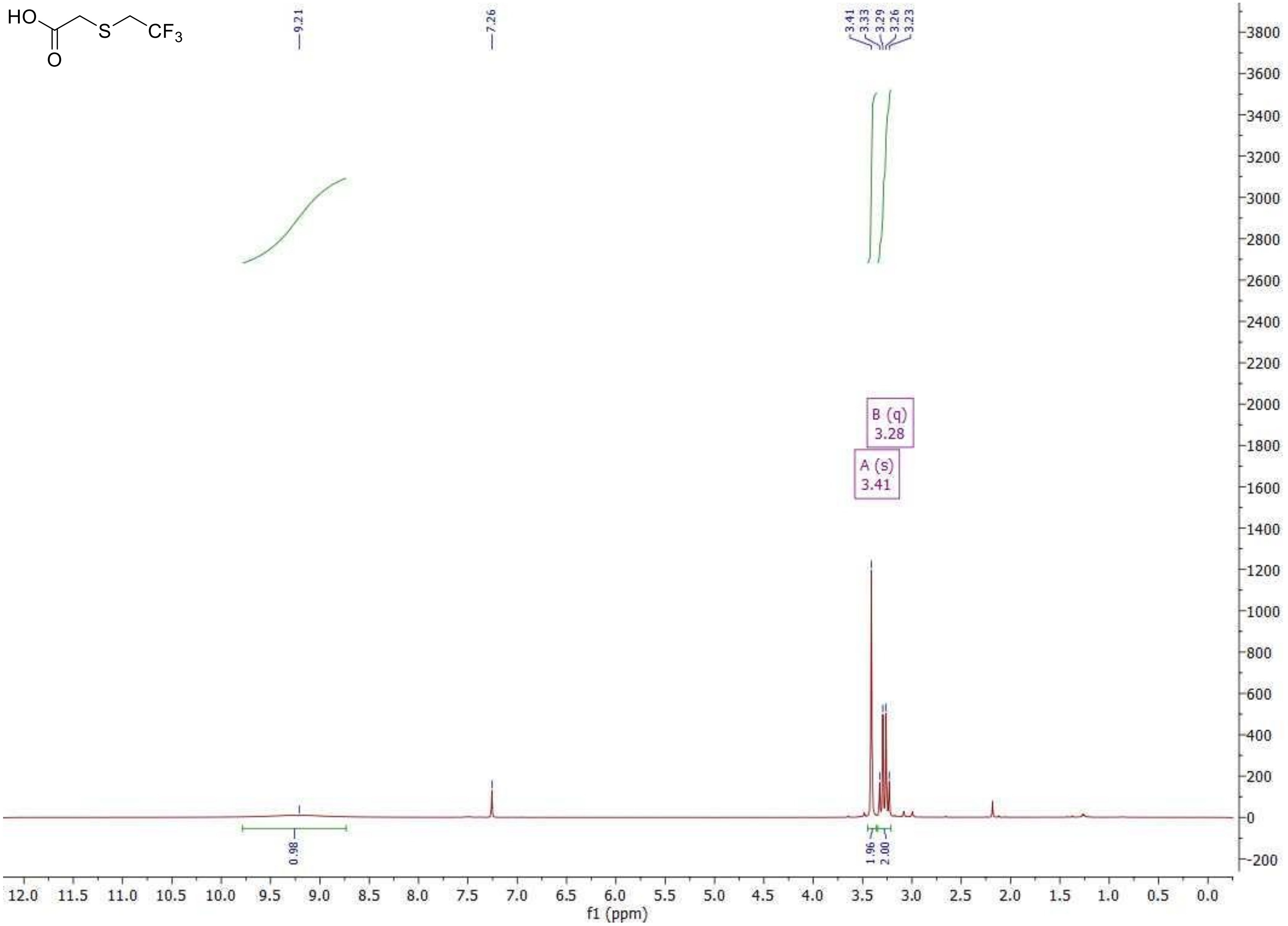

10s (^{13}C NMR in $CDCl_3$) (75 MHz)

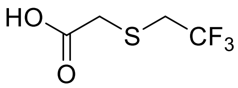

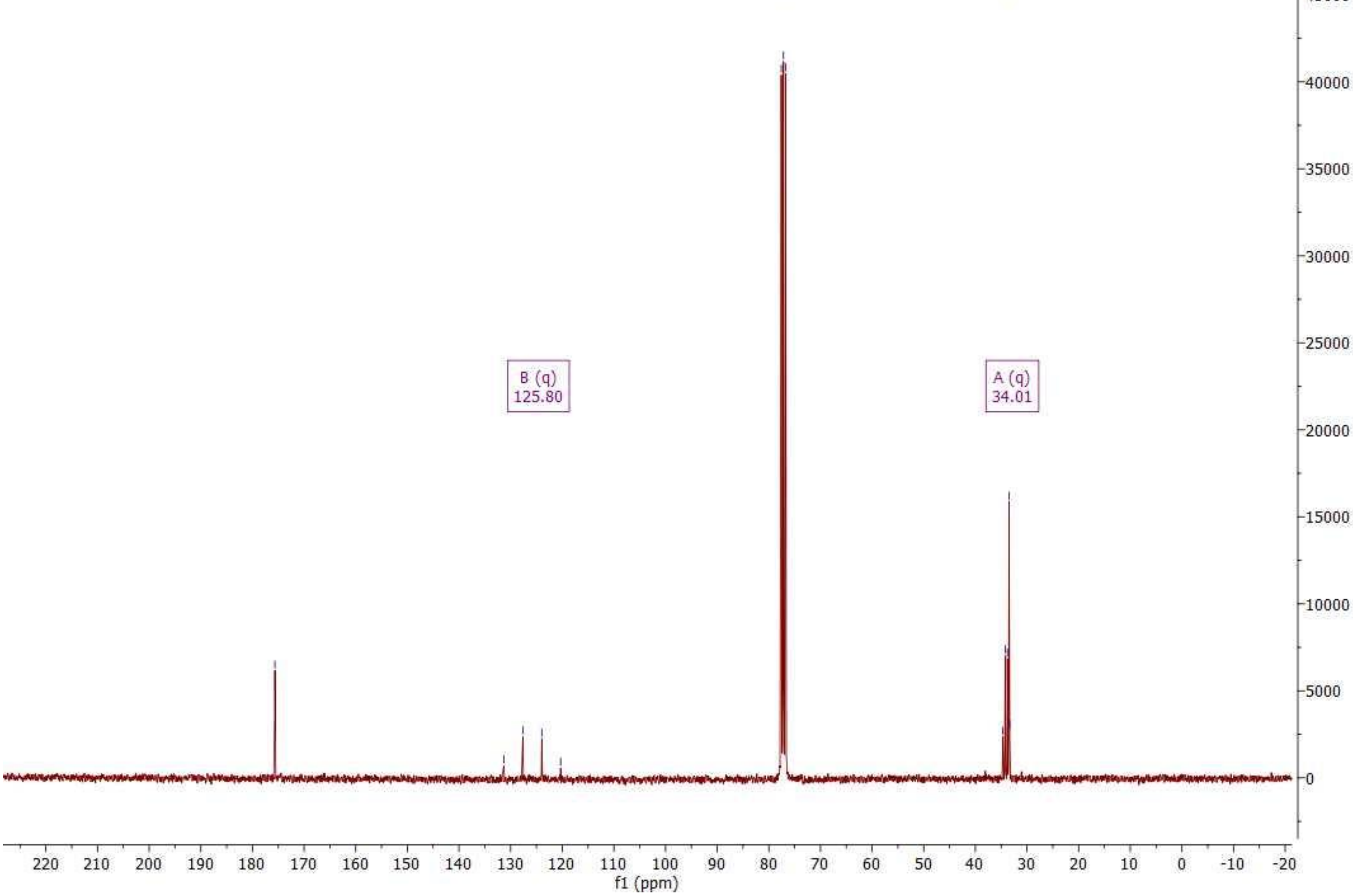

10s (^{19}F NMR in $CDCl_3$) (282 MHz)

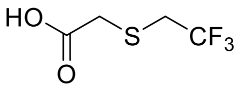

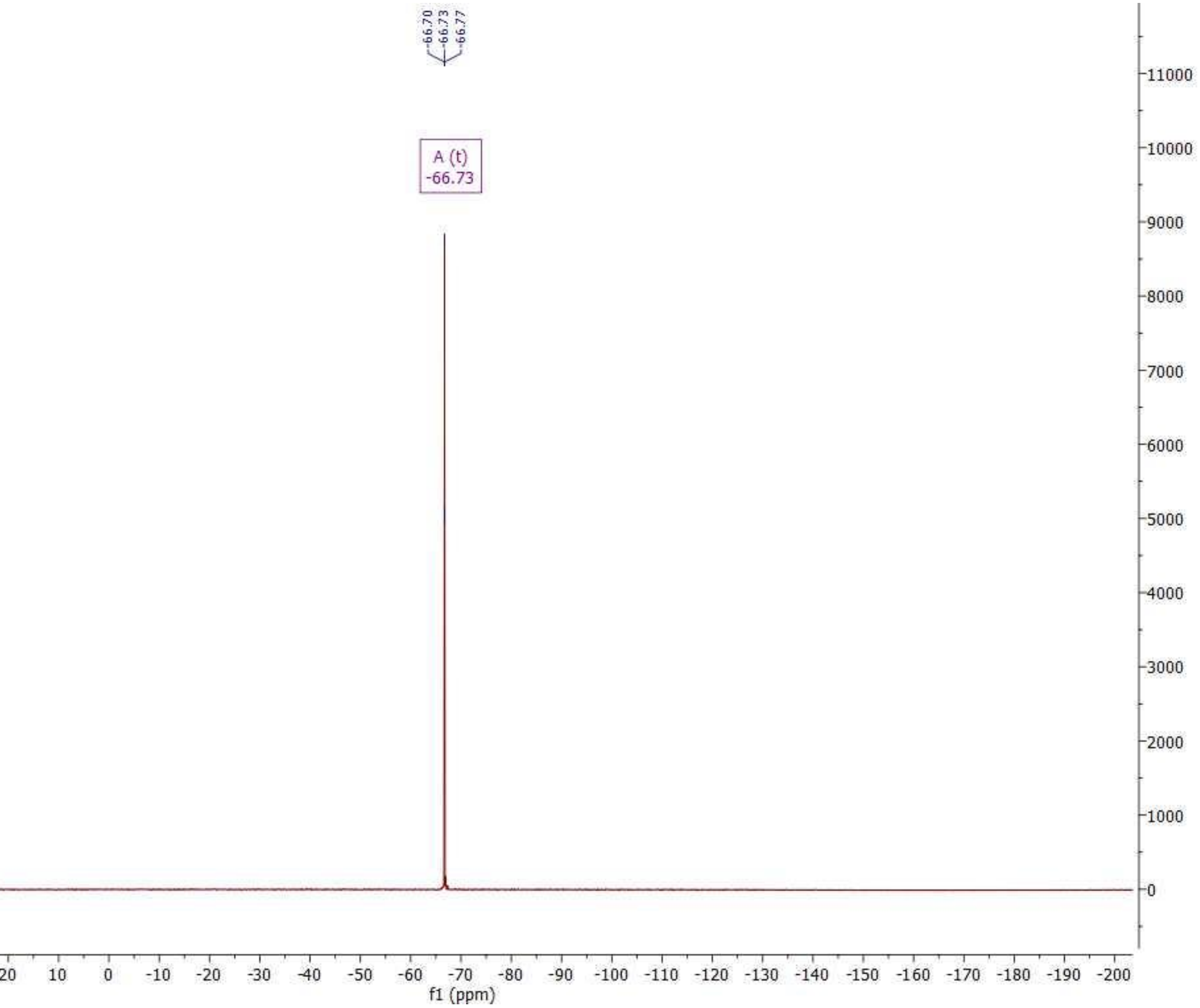

10t (^{1}H NMR in $CDCl_3$) (300 MHz)

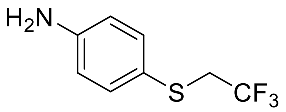

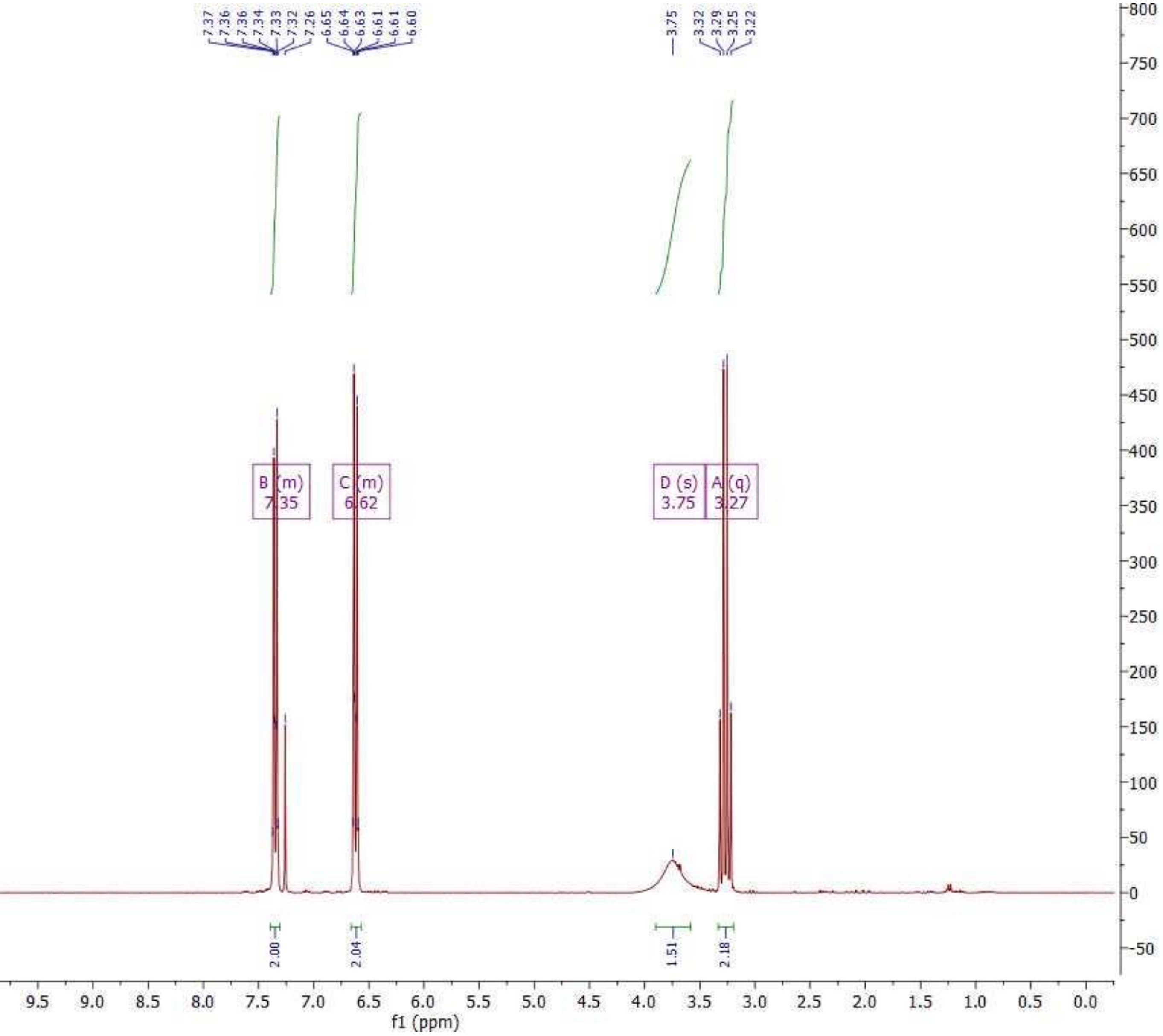

10t (^{13}C NMR in $CDCl_3$) (75 MHz)

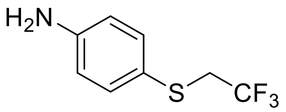

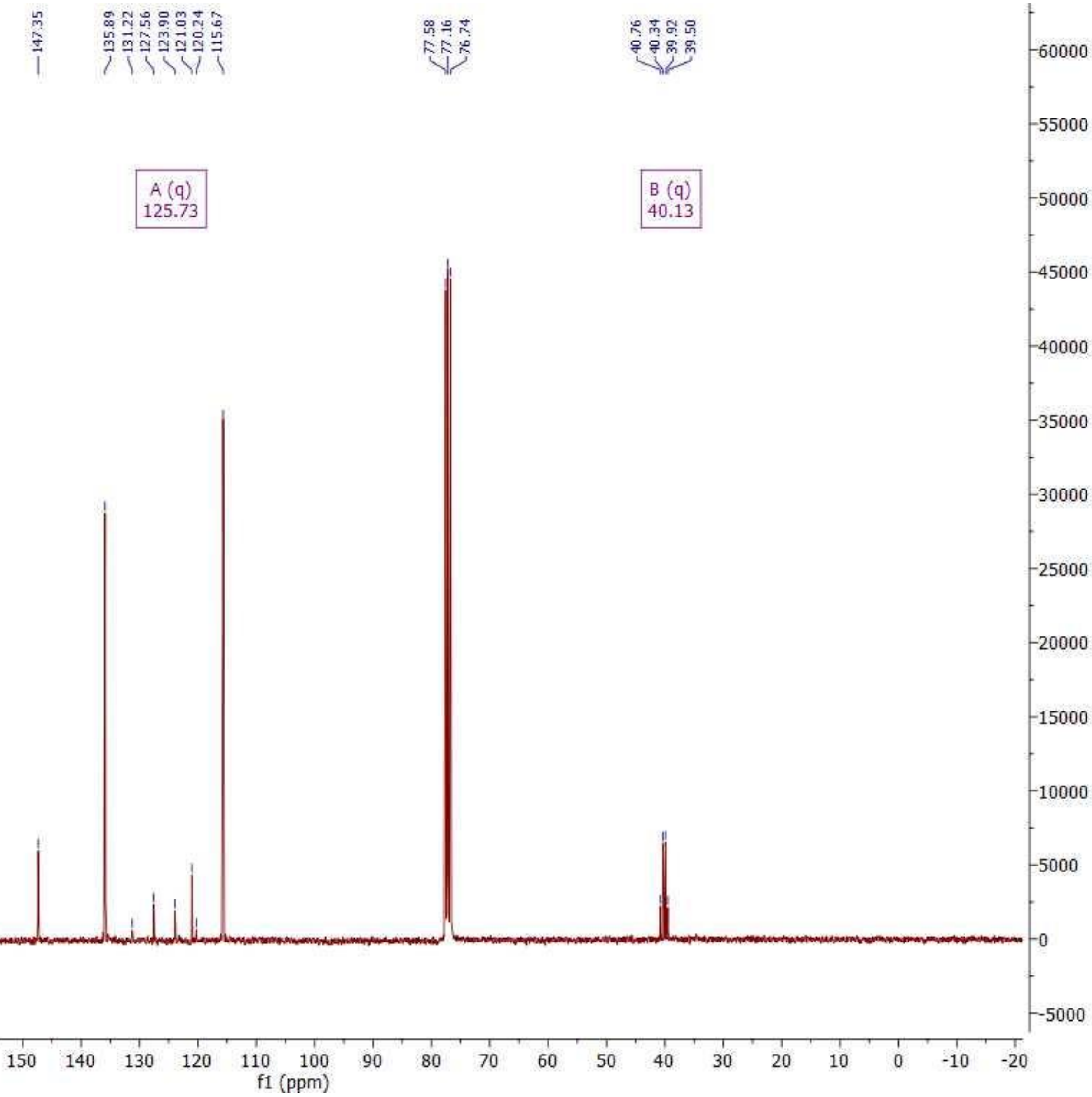

10t (^{19}F NMR in $CDCl_3$) (282 MHz)

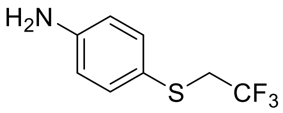

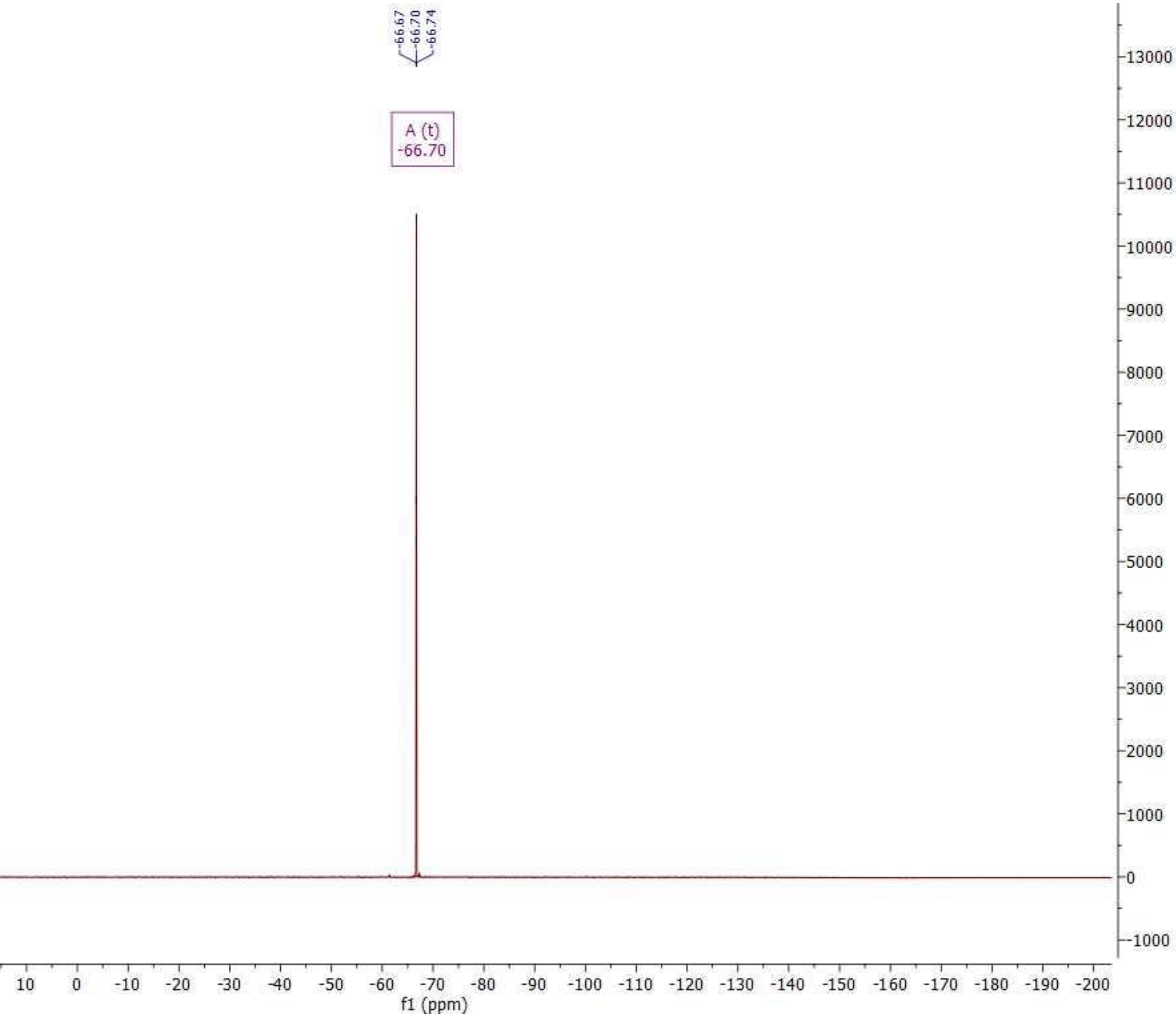

10u (^{1}H NMR in $CDCl_3$) (300 MHz)

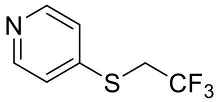

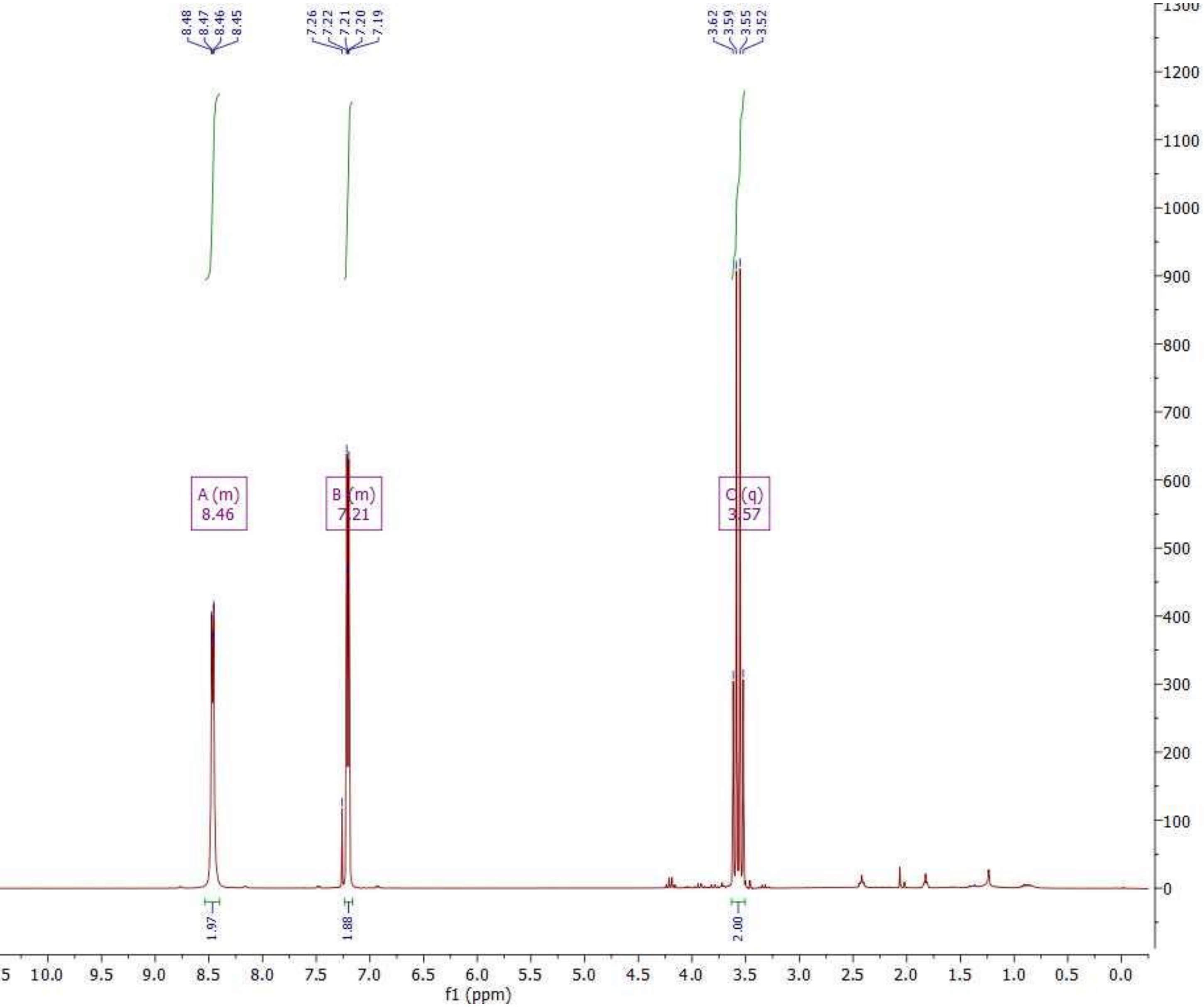

10u (^{13}C NMR in $CDCl_3$) (75 MHz)

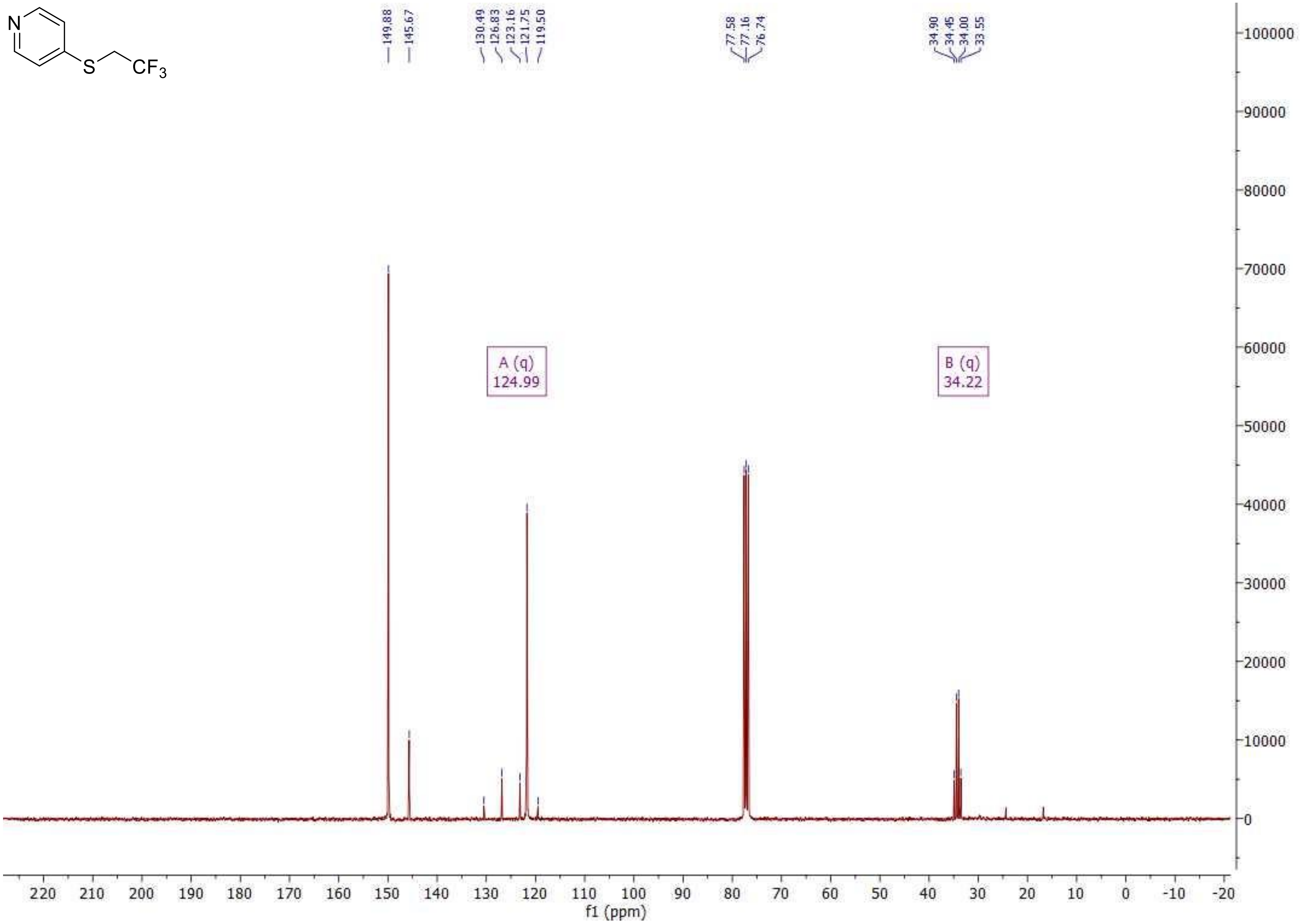

10u (^{19}F NMR in $CDCl_3$) (282 MHz)

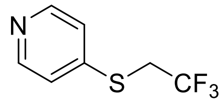

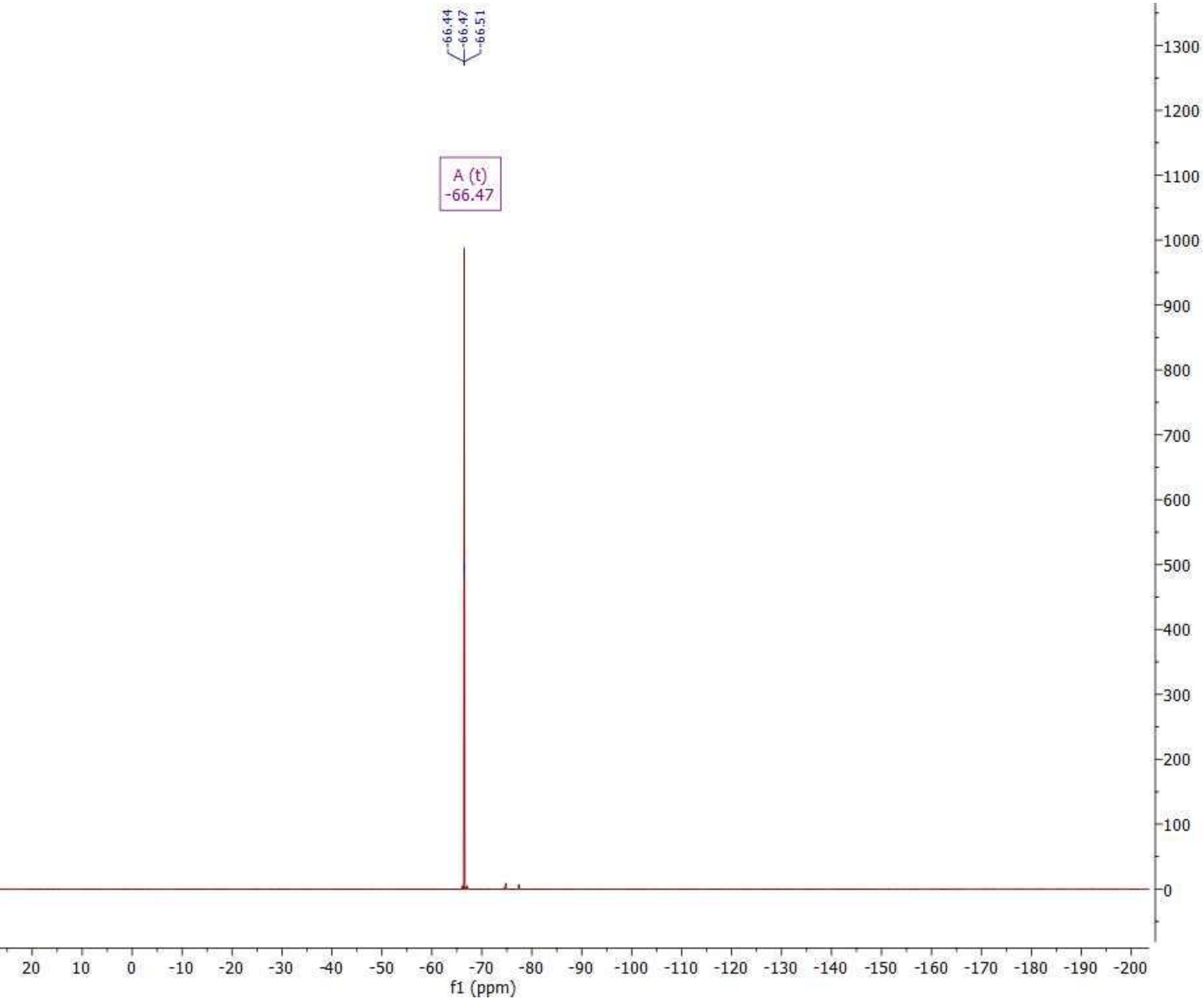

10v (^{1}H NMR in $CDCl_3$) (300 MHz)

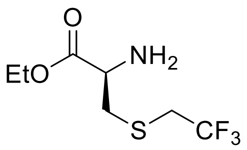

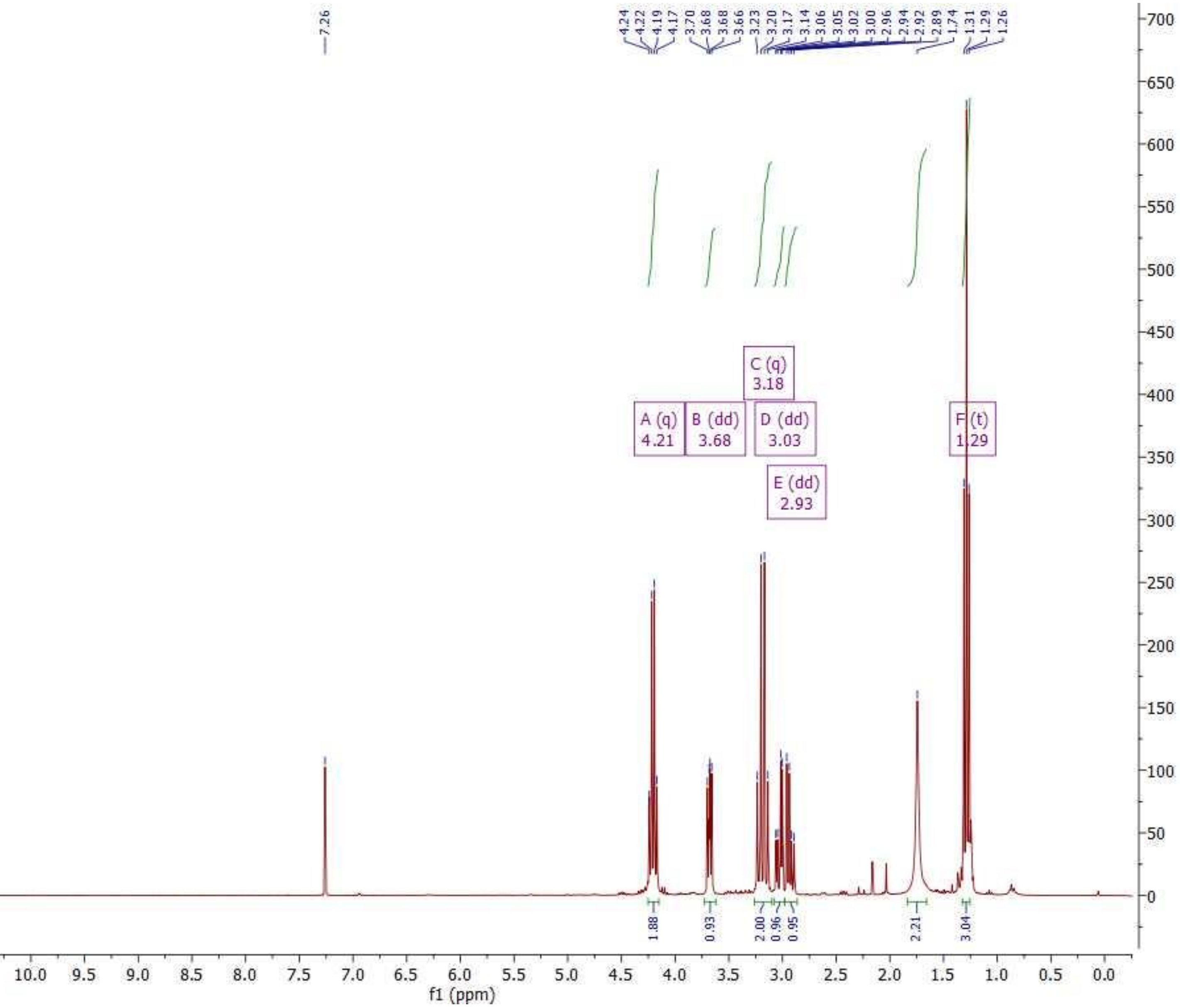

10v (^{13}C NMR in $CDCl_3$) (75 MHz)

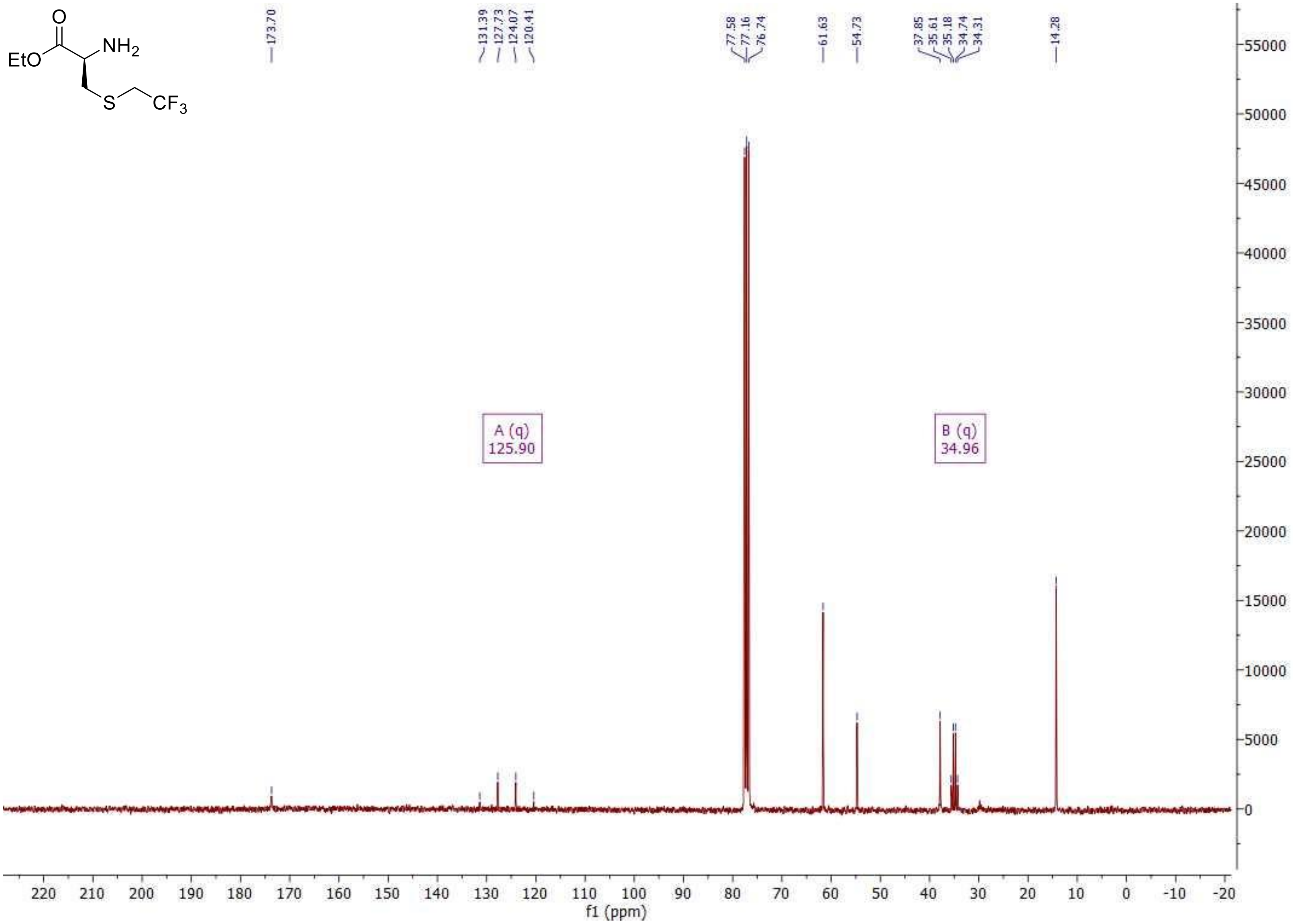

10v (^{19}F NMR in $CDCl_3$) (282 MHz)

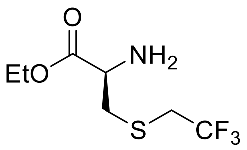

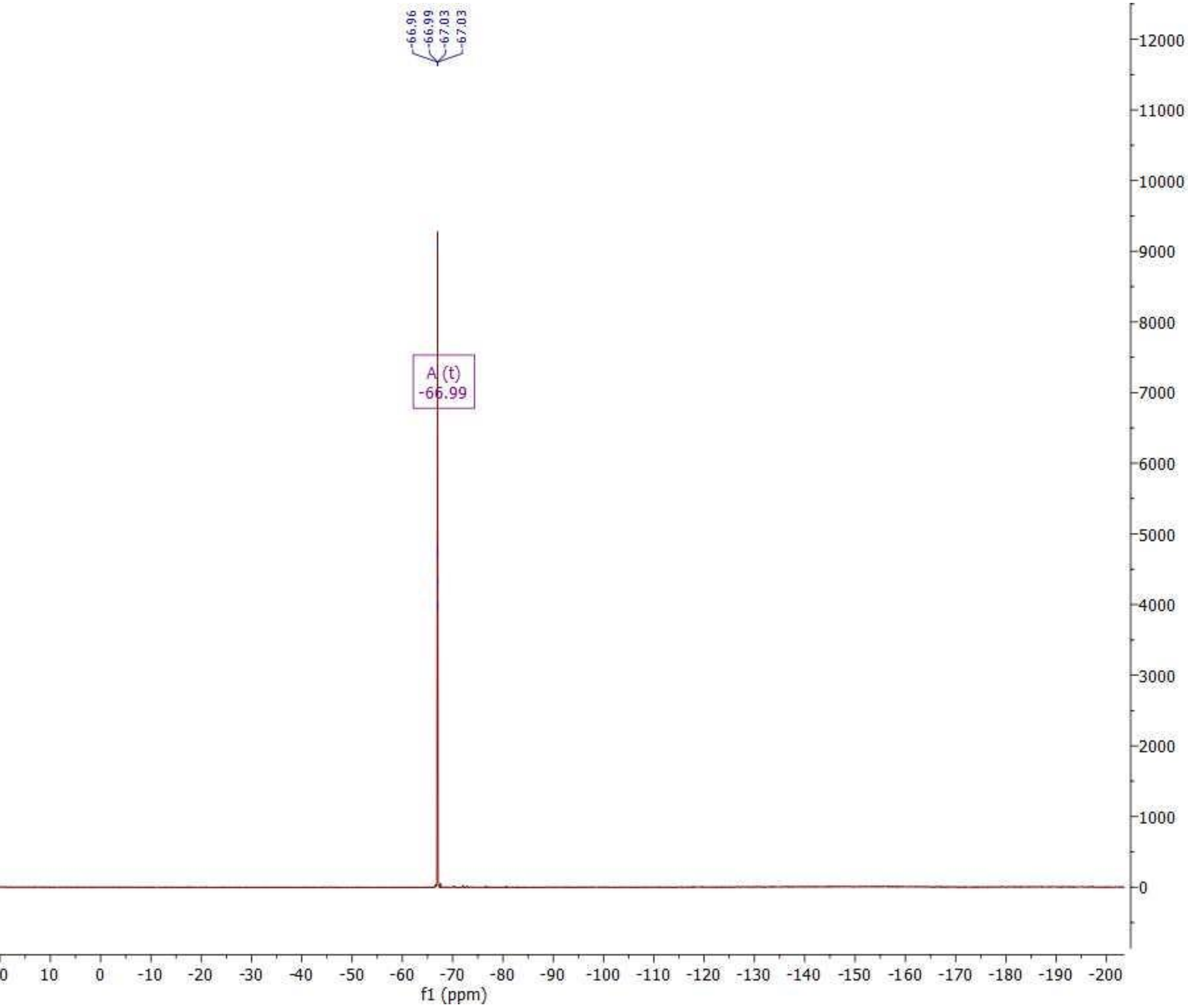

10w (^{1}H NMR in D_2O) (300 MHz)[a]

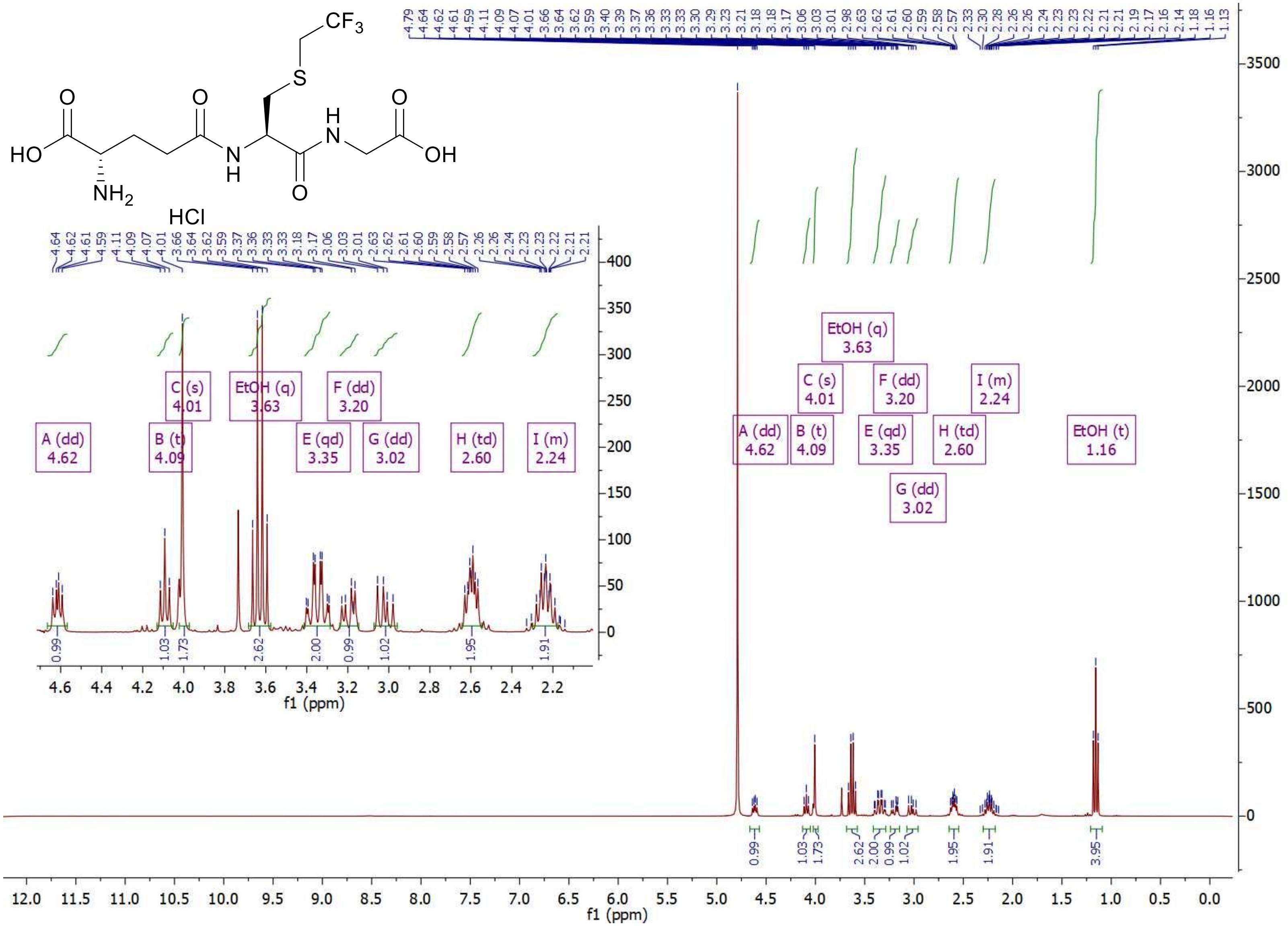

[a]*The product is contaminated with calcd. 12% ethanol (labeled above). See the experimental section for details.*

10w (^{13}C NMR in D_2O) (75 MHz)[a]

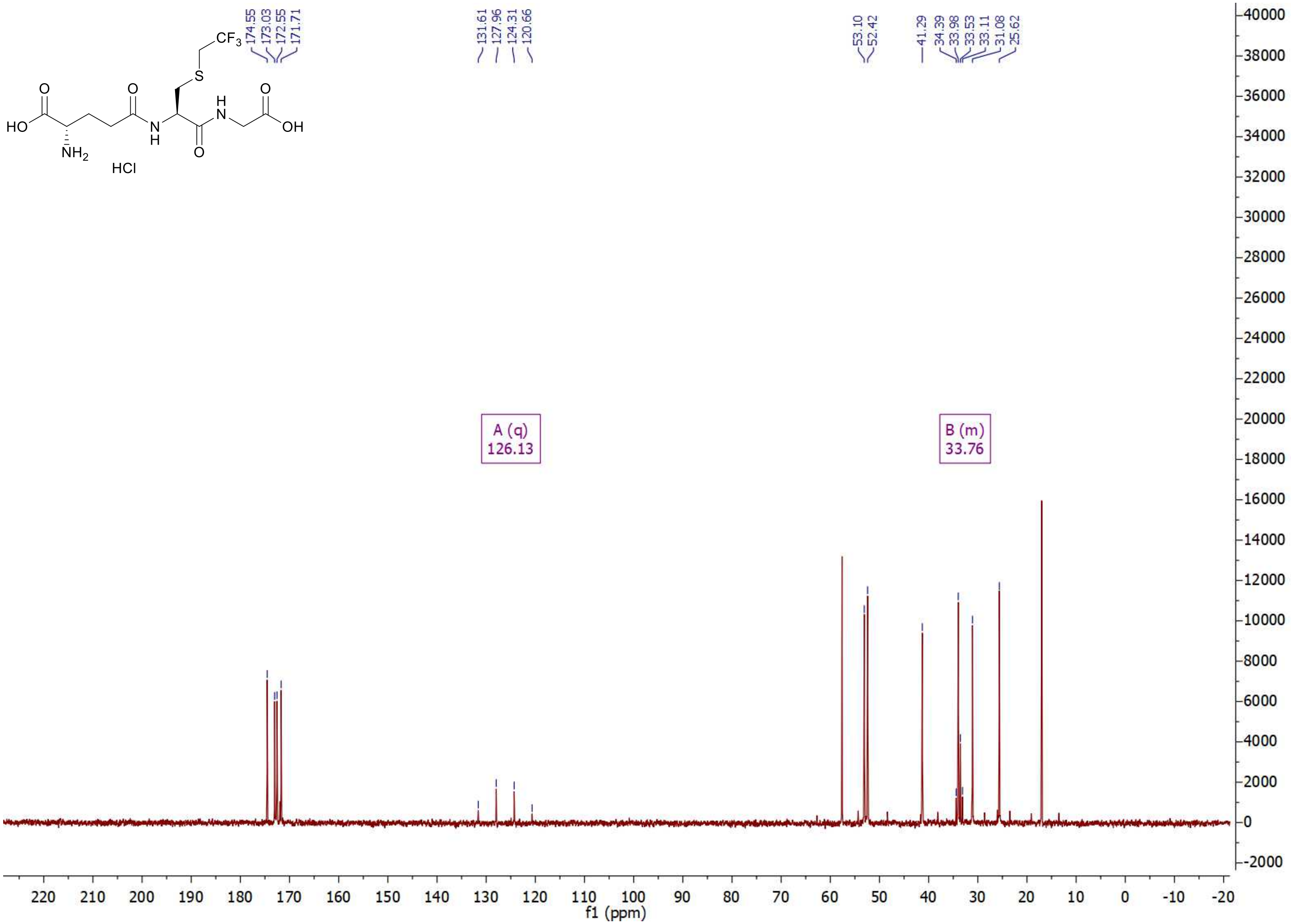

[a]*The product is contaminated with calcd. 12% ethanol. See the experimental section for details.*

10w (^{19}F NMR in D_2O) (282 MHz)

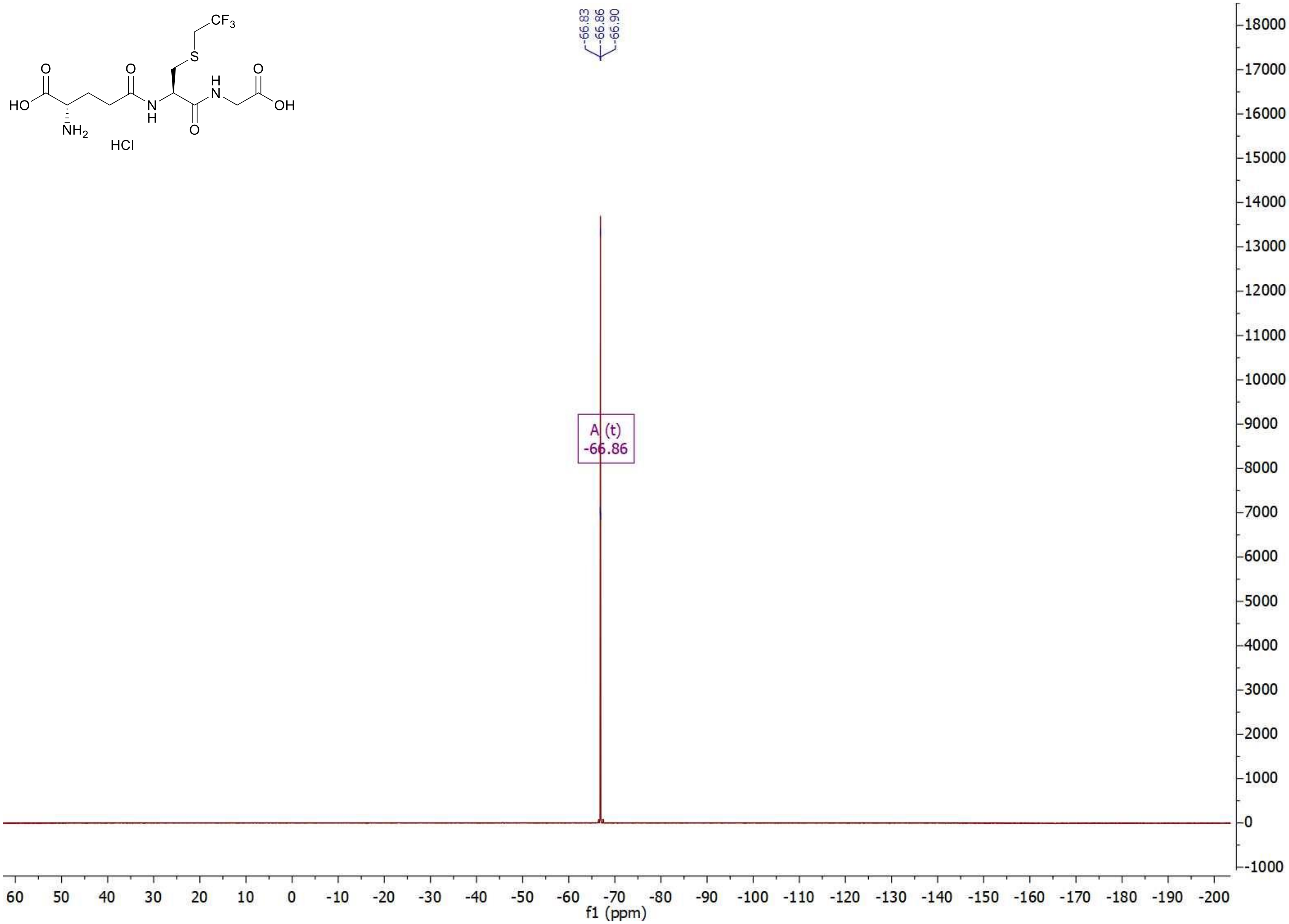

11a (^{1}H NMR in $CDCl_3$) (300 MHz)

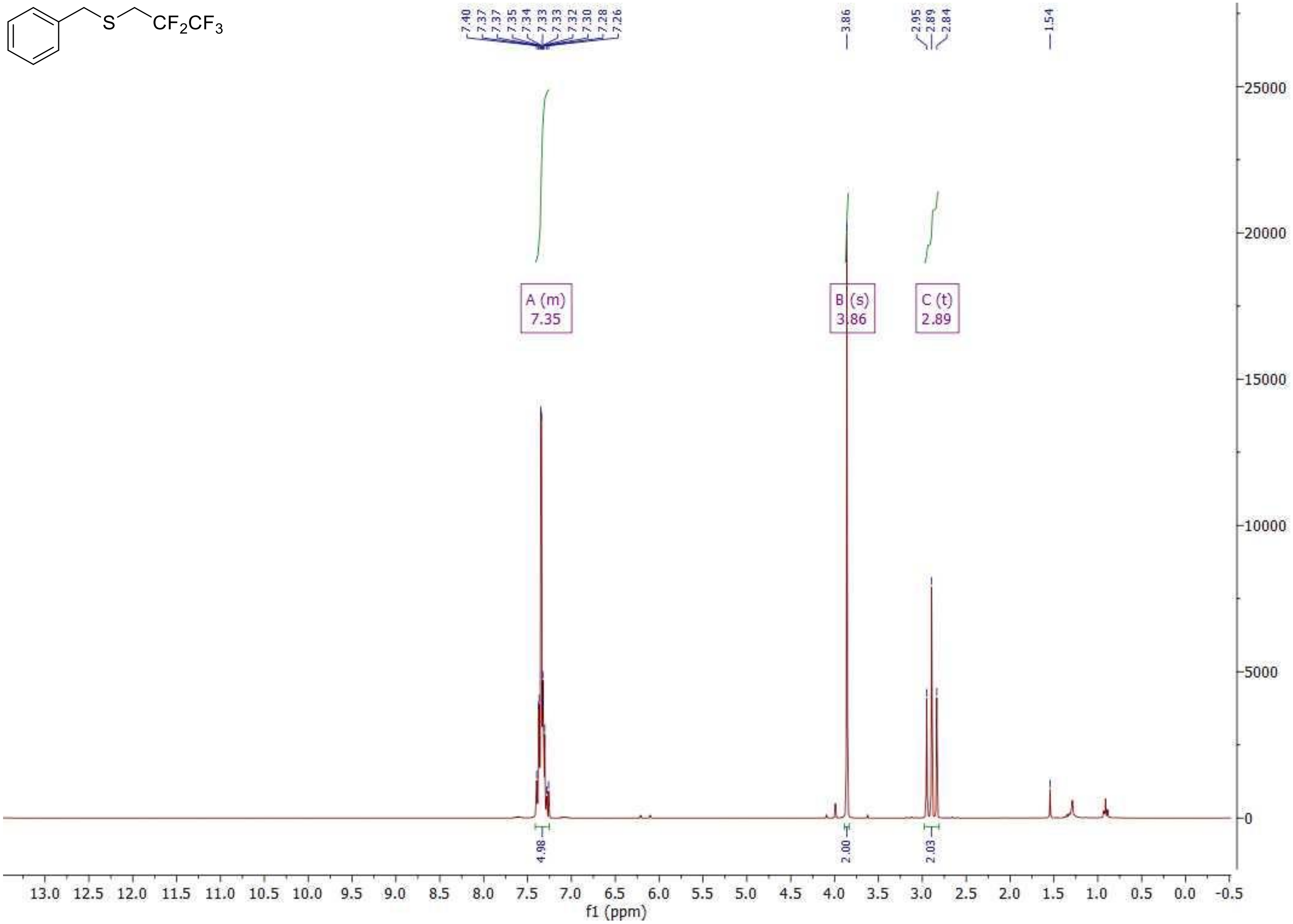

11a (^{13}C NMR in $CDCl_3$) (75 MHz)

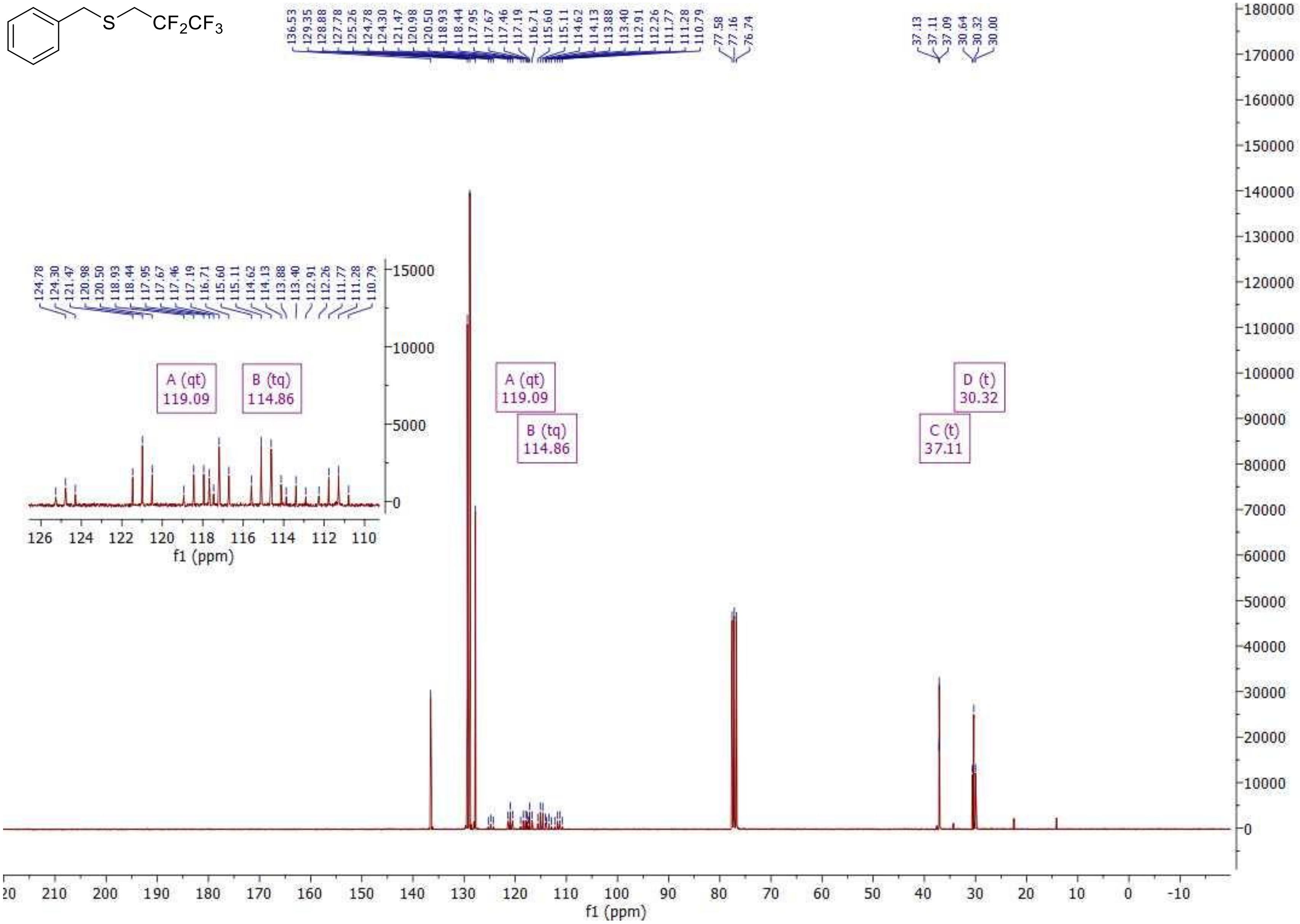

11a (^{19}F NMR in $CDCl_3$) (282 MHz)

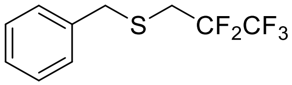

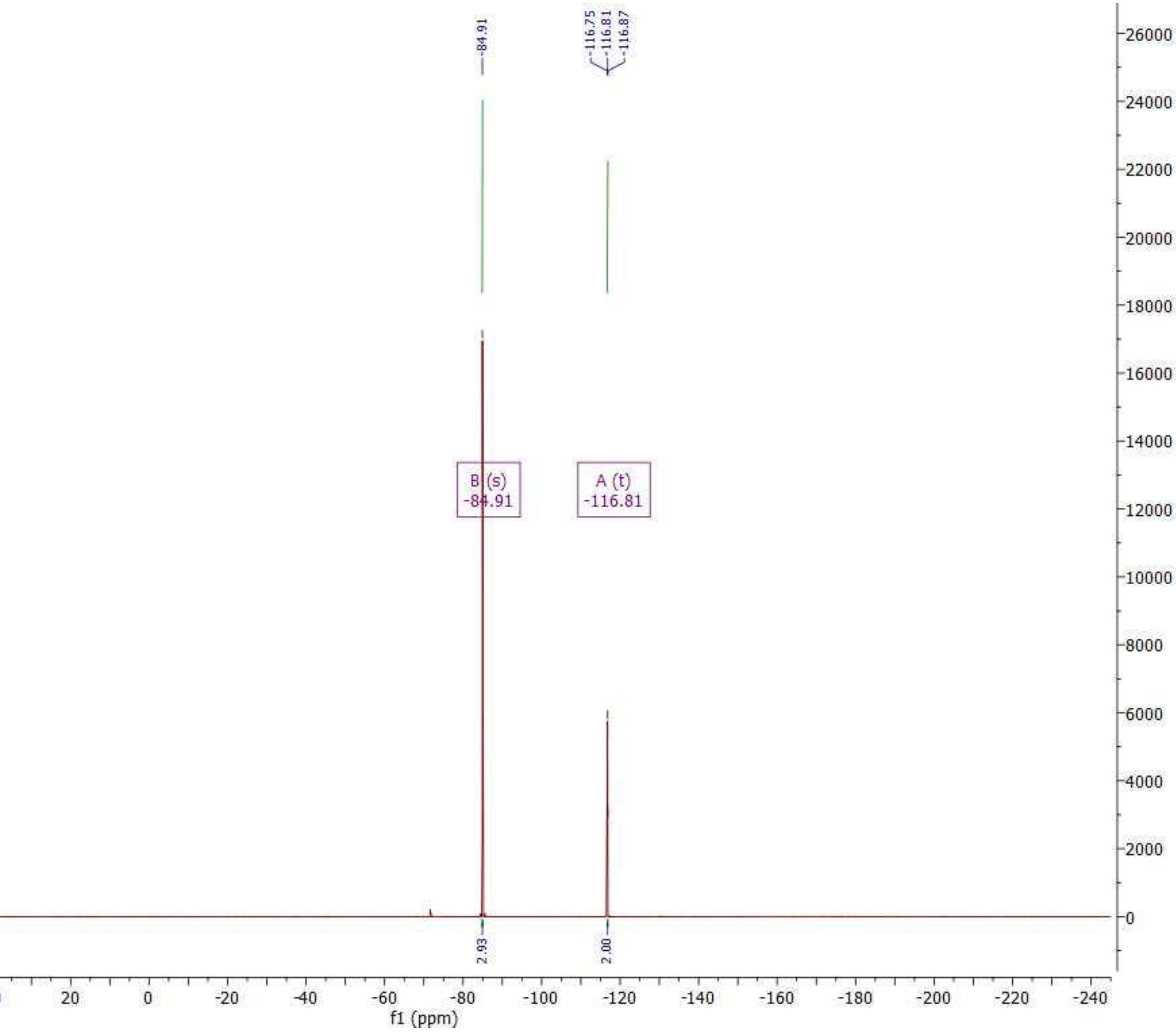

11c (^1H NMR in $CDCl_3$) (300 MHz)

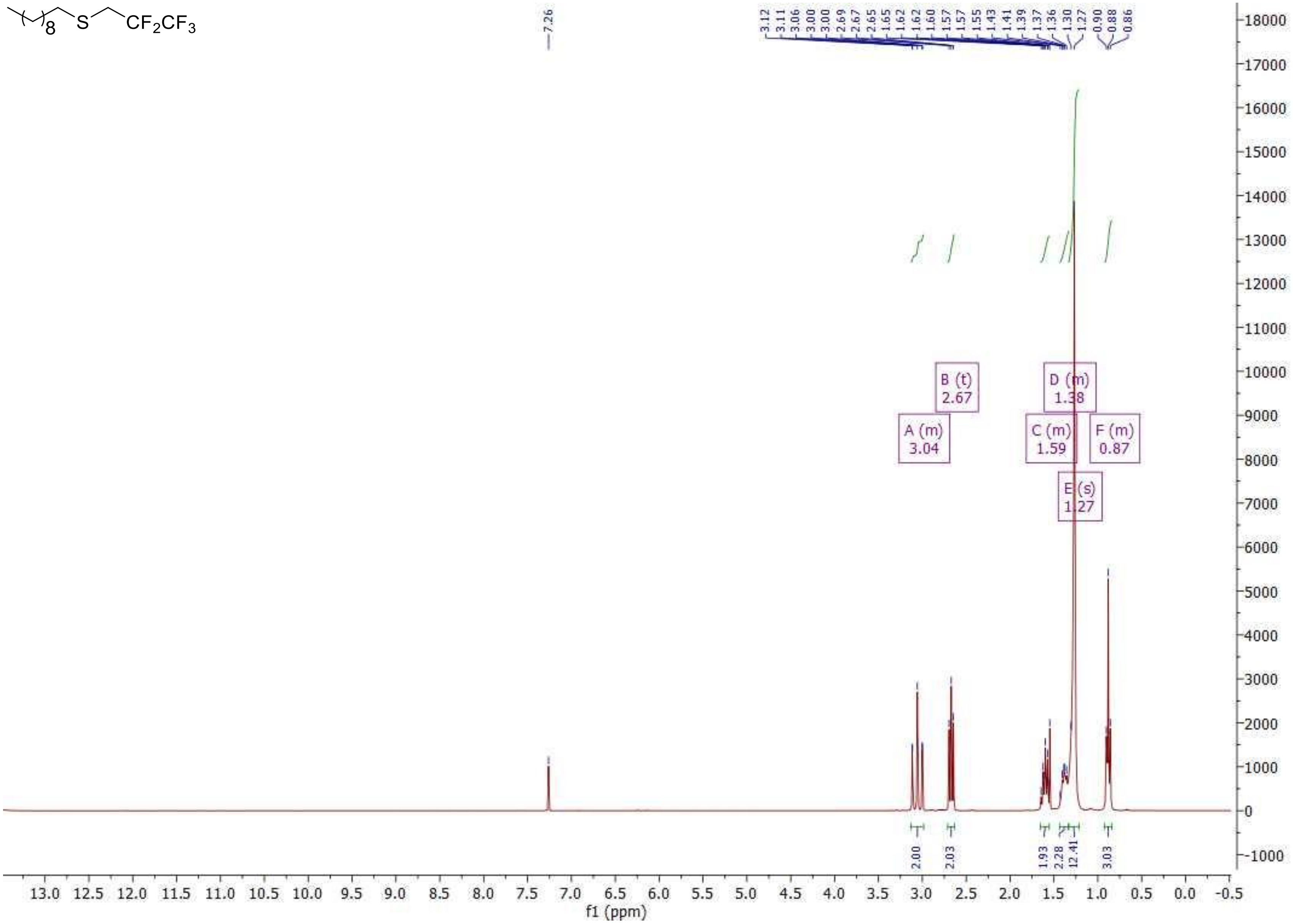

11c (^{13}C NMR in $CDCl_3$) (75 MHz)

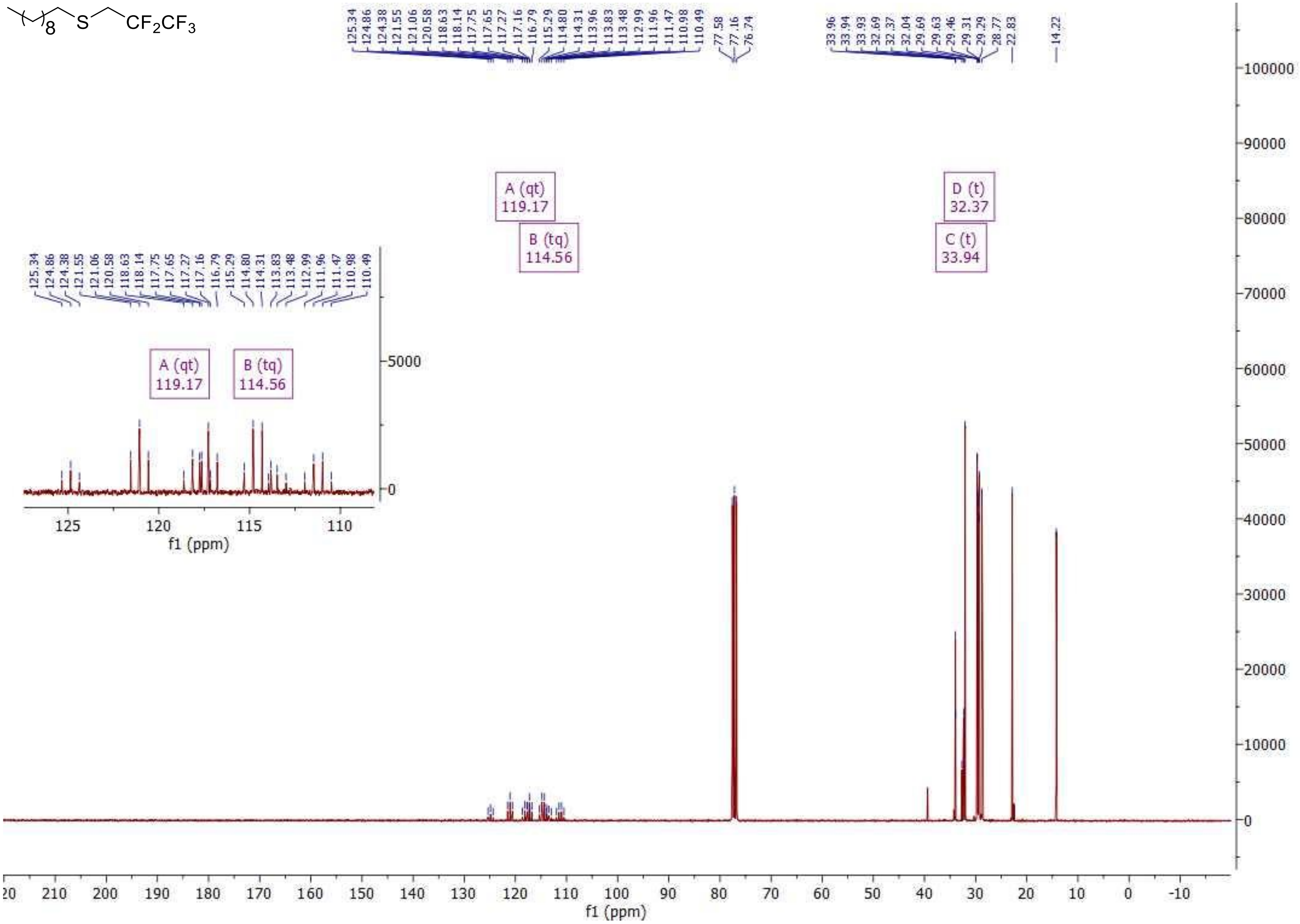

11c (^{19}F NMR in $CDCl_3$) (282 MHz)

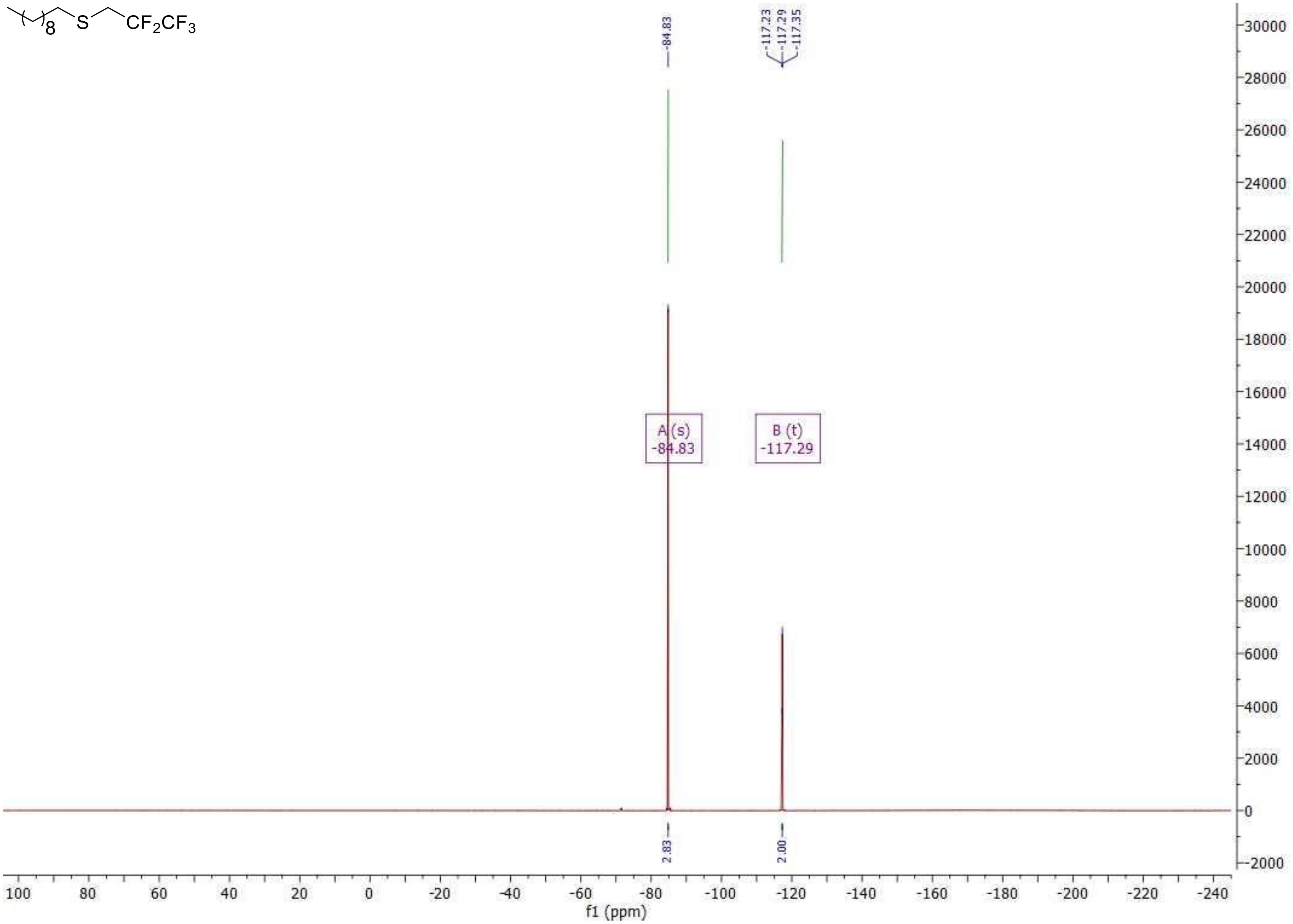

11r (^1H NMR in $CDCl_3$) (300 MHz)

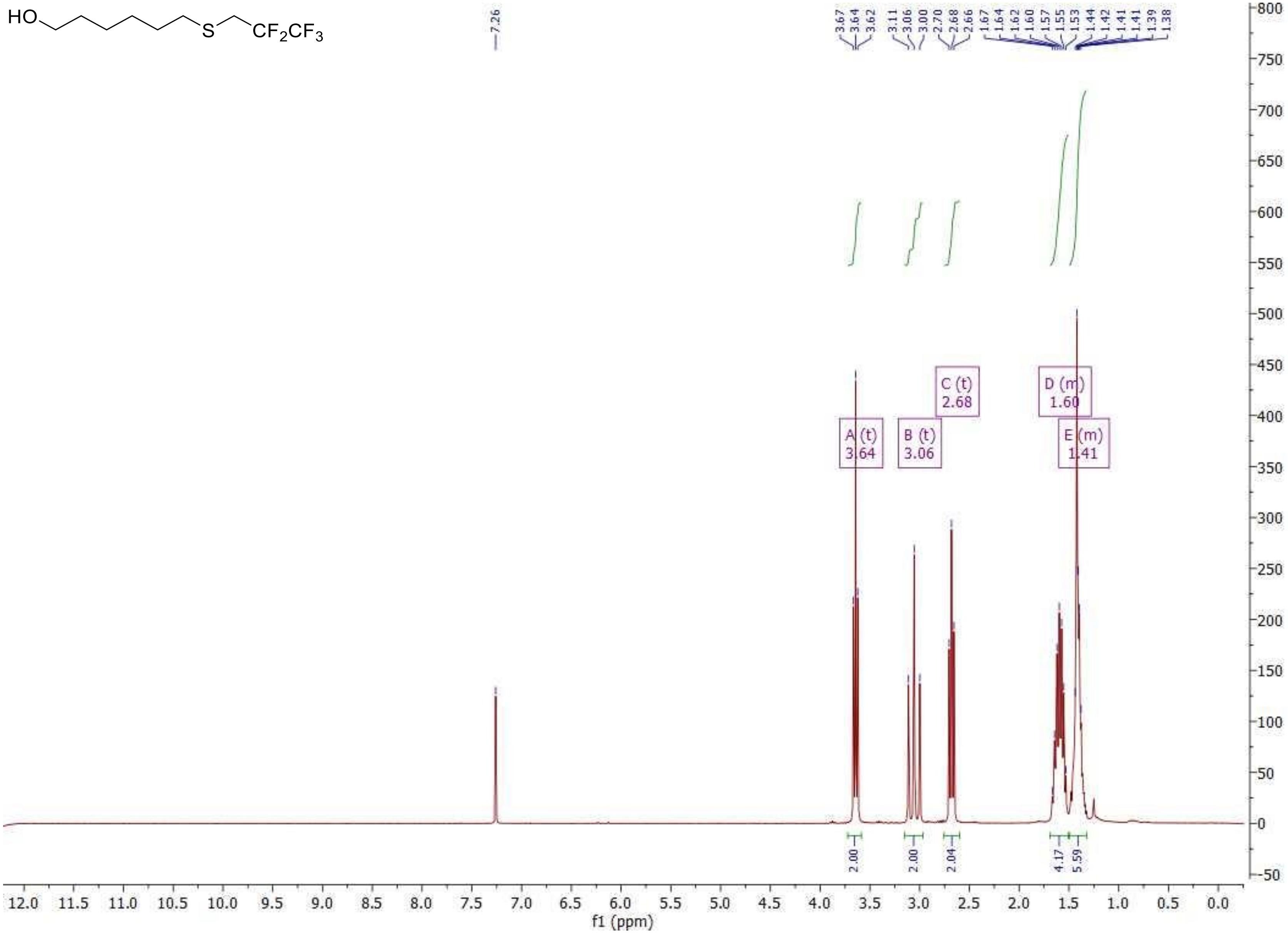

11r (^{13}C NMR in $CDCl_3$) (75 MHz)

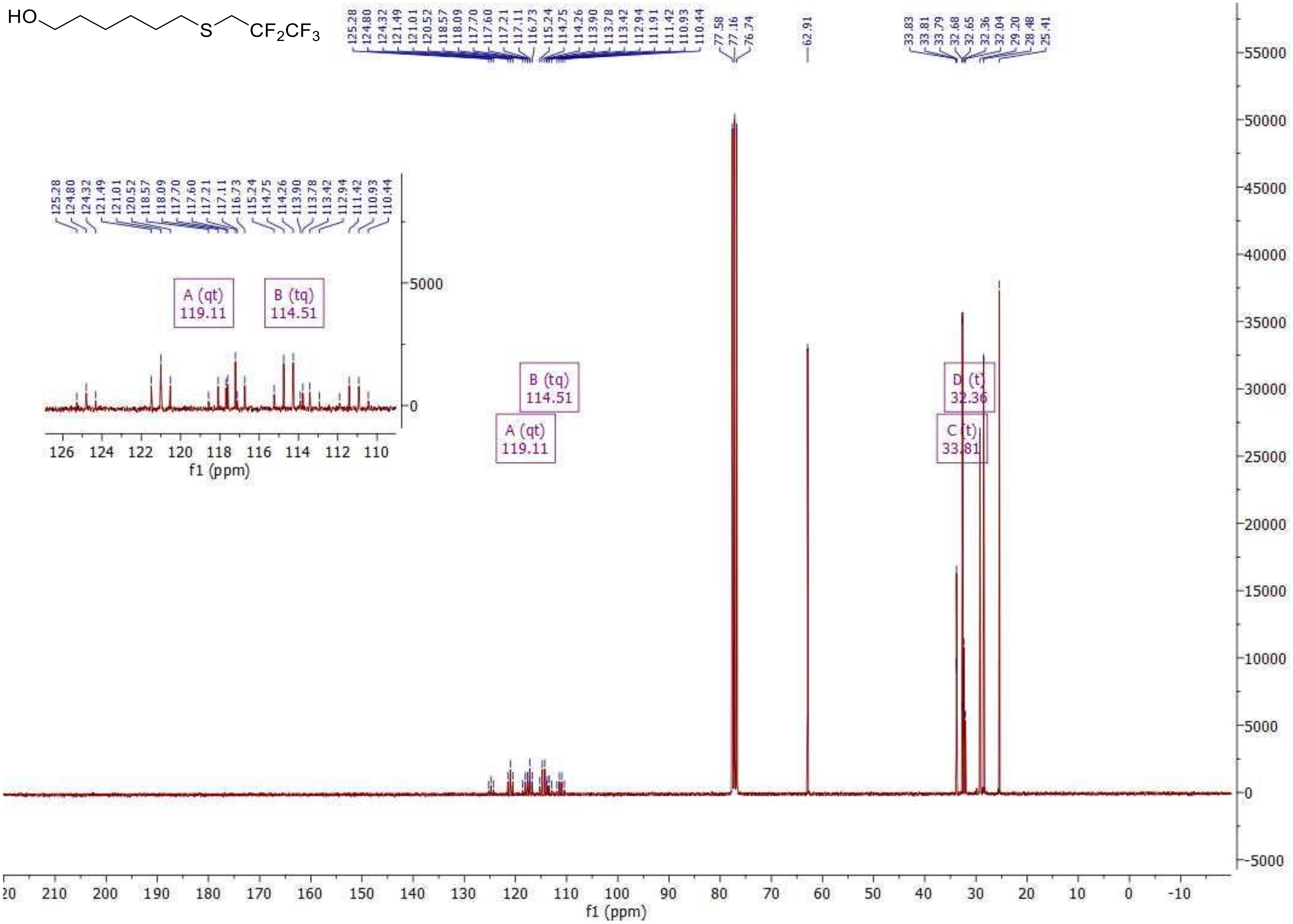

11r (^{19}F NMR in $CDCl_3$) (282 MHz)

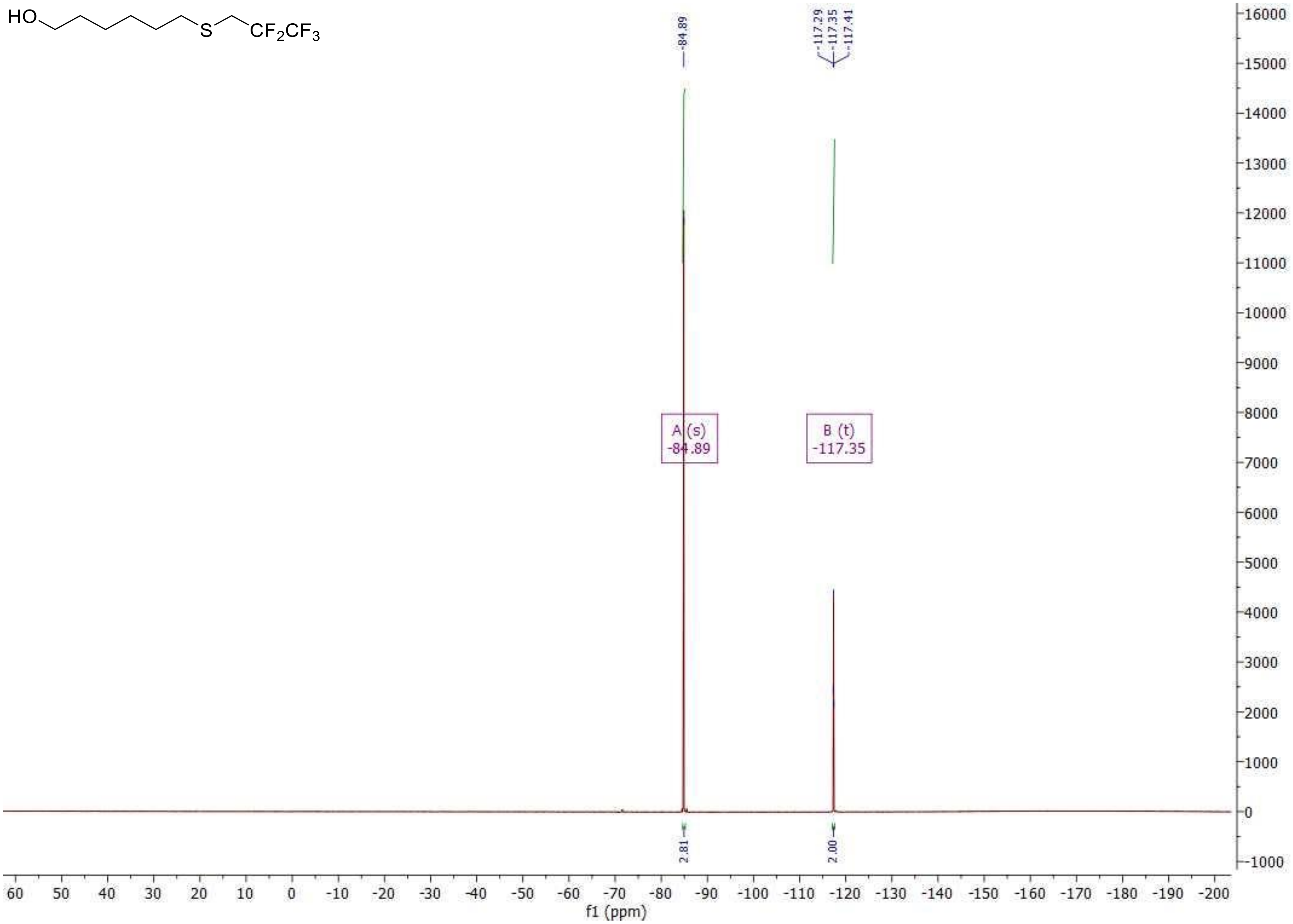

11t (^1H NMR in $CDCl_3$) (300 MHz)

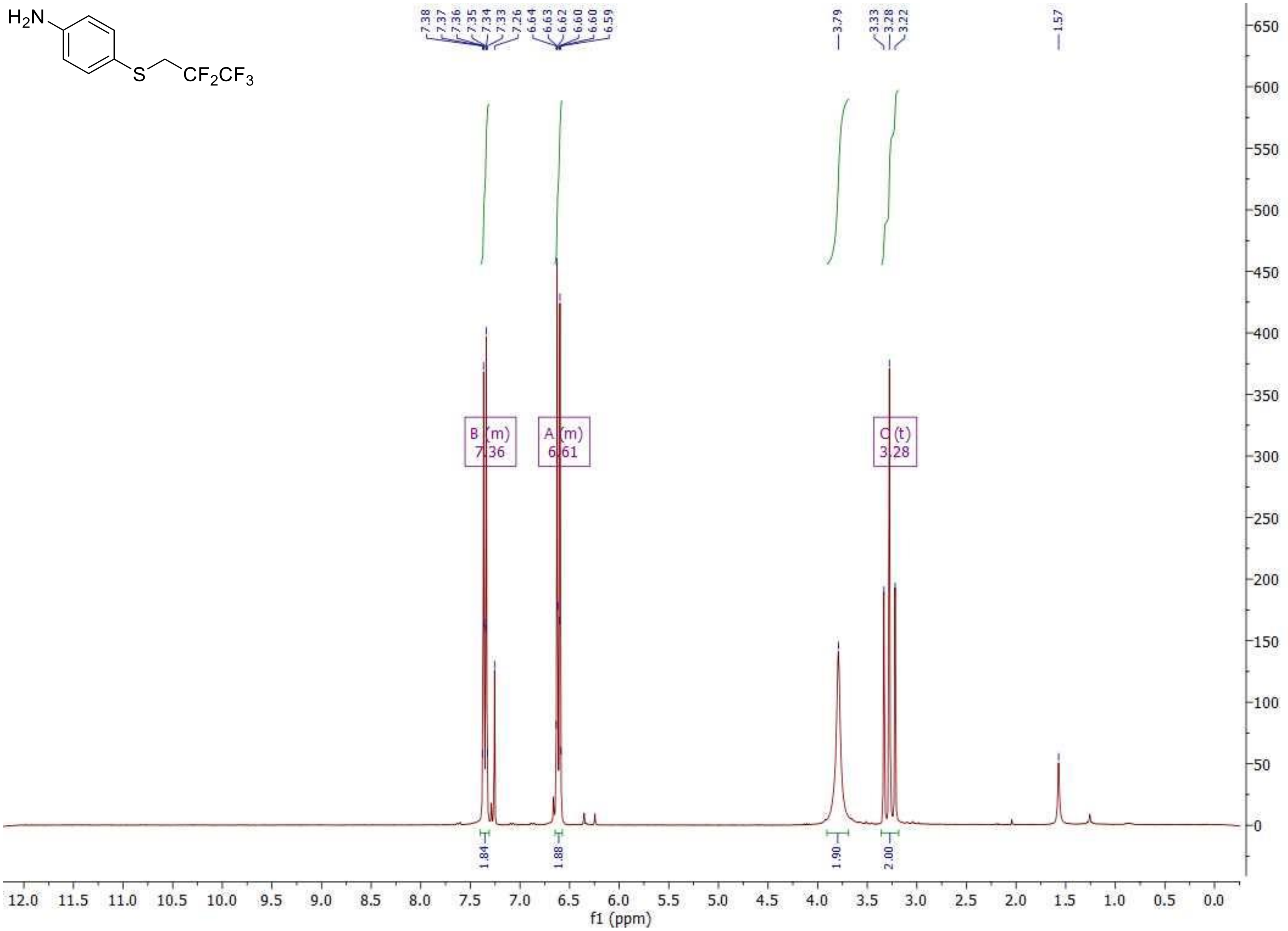

11t (^{13}C NMR in $CDCl_3$) (75 MHz)

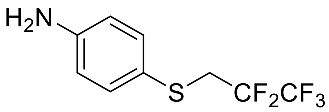

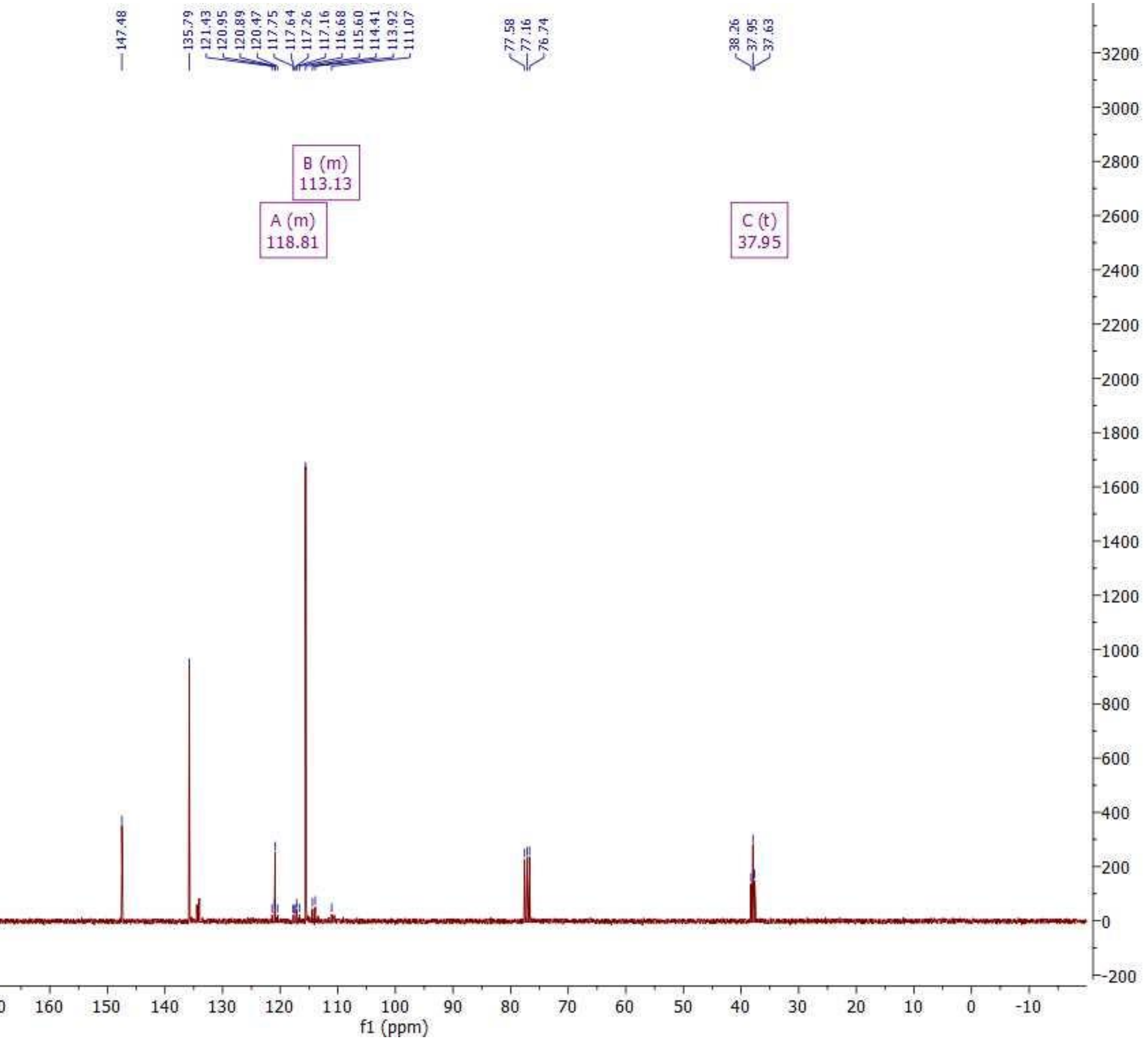

11t (^{19}F NMR in $CDCl_3$) (282 MHz)

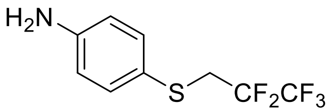

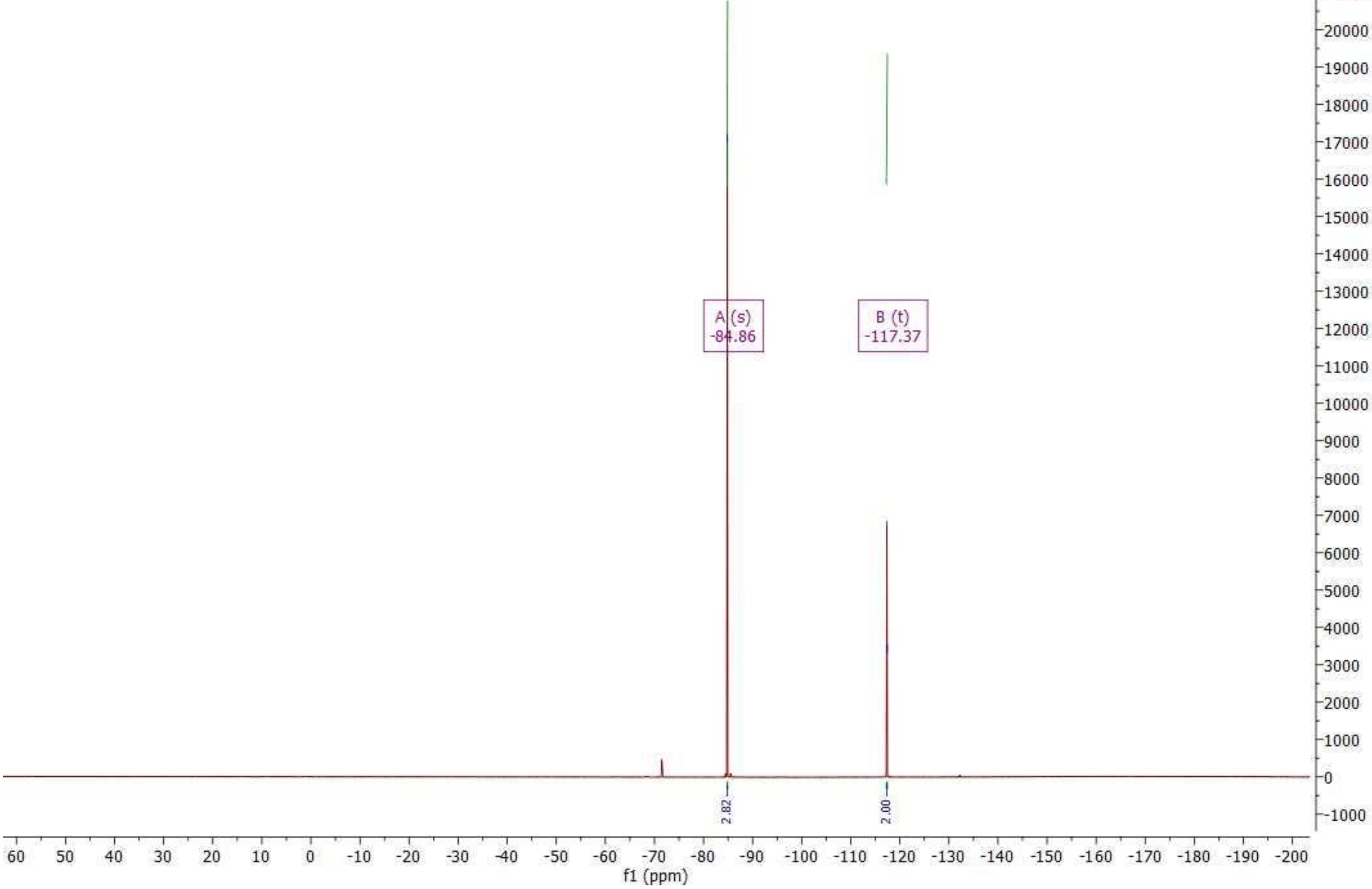

Supplement: Supplementary file 1 [file SC-010-C9SC03570B-s001.pdf]
